# Novel chlorinated phospholipids—possible biomarkers of chlorine gas exposure

**DOI:** 10.1007/s00216-025-05864-6

**Published:** 2025-04-11

**Authors:** Noora-Kaisa Rantanen, Nurhazlina Hamzah, Matti A. Kjellberg, Solja Säde, Paula Vanninen, Hanna Hakulinen

**Affiliations:** 1 Finnish Institute for Verification of the Chemical Weapons Convention (VERIFIN), Department of Chemistry, University of Helsinki, P.O. Box 55, 00014 Helsinki, Finland; 2 Toxicology Division, Forensic Science Analysis Centre, Department of Chemistry Malaysia, 46661 Petaling Jaya, Selangor Malaysia; 3 Department of Chemistry, University of Helsinki, P.O. Box 55, 00014 Helsinki, Finland

**Keywords:** Chlorine gas, Chlorinated phospholipids, Peroxy-diphospholipids, Chemical weapons, Liquid chromatography, Mass spectrometry

## Abstract

Biomarkers are needed to verify suspected exposure to chlorine gas in chemical attacks. Here, we aimed to expand upon the array of known biomarkers of chlorine gas exposure. 1-Palmitoyl- 2-oleoyl-*sn*-glycero- 3-phosphocholine (POPC), a phospholipid belonging to the phosphatidylcholine (PC) class, was chosen as the study material. The lipids were chlorinated using chlorine gas, followed by analysis for chlorinated PCs using liquid chromatography (LC) coupled with mass spectrometry (MS), using unit- (MS, MS/MS) and high-resolution (HRMS, MS/HRMS). By interpreting accurate masses, isotopic patterns, and fragmentation patterns, PC chlorohydrin, PC dichloride, the chlorinated PC with *m*/*z* 794.54611 (here denoted as PC A) and 10 novel chlorinated PCs were identified: four PCs chlorinated at the glycerol backbone and six chlorinated peroxy-diphospholipids. Similar experiments with 1-palmitoyl-2-oleoyl-*sn*-glycero-3-phosphoethanolamine (POPE) resulted in the formation of the corresponding chlorohydrin and dichloride, as well as five chlorinated phosphatidylethanolamines (PEs) with chlorine in the glycerol backbone. Surprisingly, no chlorinated forms of 1-palmitoyl-2-stearoyl-*sn*-glycero-3-phosphocholine (PSPC) were observed in similar experiments. In total, 15 novel chlorinated lipids were found. Furthermore, to determine the relevance of the novel lipids as biomarkers, a pig lung tissue sample was chlorinated in vitro. Two monomeric chlorinated PCs and two chlorinated peroxy-diphospholipids were identified from the chlorine-exposed lung sample, showing that the novel lipid compounds are potential biomarkers for chlorine gas exposure.

**Graphical Abstract:**

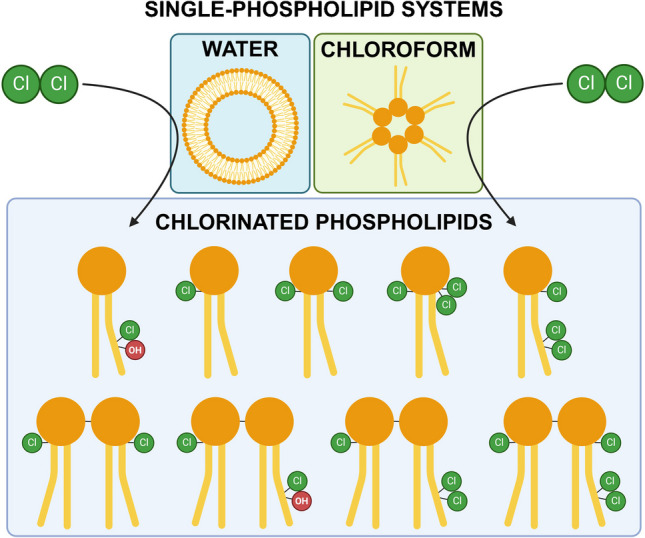

**Supplementary Information:**

The online version contains supplementary material available at 10.1007/s00216-025-05864-6.

## Introduction

Chlorine (Cl_2_) is a gaseous industrial chemical that is widely used in multiple areas of chemistry, such as in water treatment and in the manufacturing of polyvinyl chloride. In addition to its civil applications, chlorine also has military applications. Chlorine is a choking agent that has been used as a chemical weapon (CW) since World War I (WWI). Although the weaponized use of chlorine is prohibited by the General Purpose Criteria (Articla II.2) of the Chemical Weapons Convention (CWC) [1], it continues to be used as a CW, accounting for 89% of the chemicals used in the Syrian civil war between 2013 and 2018 [2].

Chlorine is extremely reactive and readily forms reaction products when it comes into contact with other substances. It is broken down by sunlight within several minutes, and when it dissolves in water, it converts to chloride (Cl^−^), and hydrochloric (HCl) and hypochlorous (HOCl) acids [3]. Victims can be exposed to chlorine by inhalation, as well as skin and eye contact during incidents, such as chlorine tank leaks, improper use of chlorine-containing household chemicals, and chlorine attacks [3].

Methods are needed to detect chlorine gas exposure in biomedical samples of exposed individuals. However, finding unambiguous biomarkers for chlorine gas exposure is difficult, due to the innate immune system producing reactive chlorine species in the body, e.g. as a result of infections or chronic illnesses [4–6]. Mono- and dichlorotyrosine are two of the proposed markers for chlorine gas exposure, since both are stable and can be found in the tyrosine residues of proteins. Methods have been developed for detection of chlorotyrosines in blood and plasma [7, 8], hair [9, 10], and tissue samples [10, 11]. Chlorotyrosines, chlorodopamines, 2-amino- 6-chloropurine (DNA-adduct), and multiple other chlorine-containing biomolecules have also been detected in plants exposed to chlorination agents. Of these, chlorodopamines were identified as chlorine gas–specific biomarkers [12]. Recently, the effect of chlorine gas exposure on volatile organic compounds (VOCs) in exhaled air was studied using mice. The authors observed several chlorinated chemicals and other changes in the VOC composition of the exhaled air of the chlorine-exposed individuals [13]. Though interesting, one should keep in mind that the results obtained with plants and mice may not be translatable for humans.

Notably, chlorine biomarkers can also be found in lipids. Thus far, the primary species of chlorinated lipids studied include chlorinated sterols and fatty acids, phospholipid chlorohydrins, α-chloro fatty aldehydes, and the oxidation products of chlorinated fatty acids and aldehydes [8, 12, 14–19]. Chlorinated lipids have been detected in human [15, 16] and animal [8, 18, 19] lung samples, plasma [8, 18], and abscessed post-mortem human tissue [17]. Just like other published biomarkers for chlorine gas exposure, chlorinated lipids and fatty acids are also produced by e.g. medical conditions and in exposure to chlorine containing household chemicals. Though, the timescale in which the chlorinated lipids become detectable may vary for the chlorination sources [16].

Since chlorine is absorbed into the body via inhalation, it is beneficial to collect samples from the upper and lower airways, particularly from the nose to the bronchial level [3]. Two suitable biomedical specimens for this are bronchoalveolar lavage fluid (BALF) and nasal lavage fluid (NLF). Collection of BALF samples involves the insertion of a bronchoscope through the mouth or nose into the lungs and is rarely performed on healthy humans. Sampling of NFL is a more realistic option; it is obtained by squirting fluid into the patient’s nostrils, and the collected fluid is used for analysis [20]. Both BALF and NLF contain lipids making them suitable samples for the analysis of chlorinated lipid biomarkers of chlorine exposure. Phospholipid chlorohydrins such as the chlorohydrin of 1-palmitoyl- 2-oleoyl-*sn*-glycero- 3-phosphocholine (POPC) have been detected in BALF [15, 19] and NLF [16]. Chlorinated fatty acids such as α-chlorofatty acid are also found in BALF [15]. Other chlorinated lipids, namely 2-chloropalmitaldehyde, 2-chloro-stearaldehyde, and their oxidized products (free and esterified 2-chloropalmitic acid, and 2-chlorostearic acid) have been detected in the lungs of chlorine-exposed mice and rats [18].

Phospholipids are the most prevalent lipid component in the exoplasmic (inner) and cytosolic (outer) leaflets of the plasma membrane in eukaryotic cells [21]. Typically, phosphatidylcholines (PCs) comprise 40–50% of the total phospholipids in the plasma membrane and are the only phospholipids present in both leaflets. In the cytosolic leaflet, the PCs are generally fully saturated, while the exoplasmic leaflet is dominated by polyunsaturated PC species [22]. PCs are a major component of the pulmonary surfactant of the lung, which comprises of 70–80% phospholipids [23, 24]. The presence of double bonds in unsaturated fatty acyl chains has a significant effect on the membrane structure fluidity [21]. PCs and phosphatidylethanolamines (PEs) are the most abundant phospholipids in human nasal mucosa [16]. It has been proposed that the formation of chlorinated lipids occurs when HOCl reacts with lipids via electrophilic attack on the double bonds in the unsaturated fatty acyl chains and via N-halogenation of the lipid headgroups including PC and PE, resulting in chlorohydrins and peroxidation products [14].

**Fig. 1:**
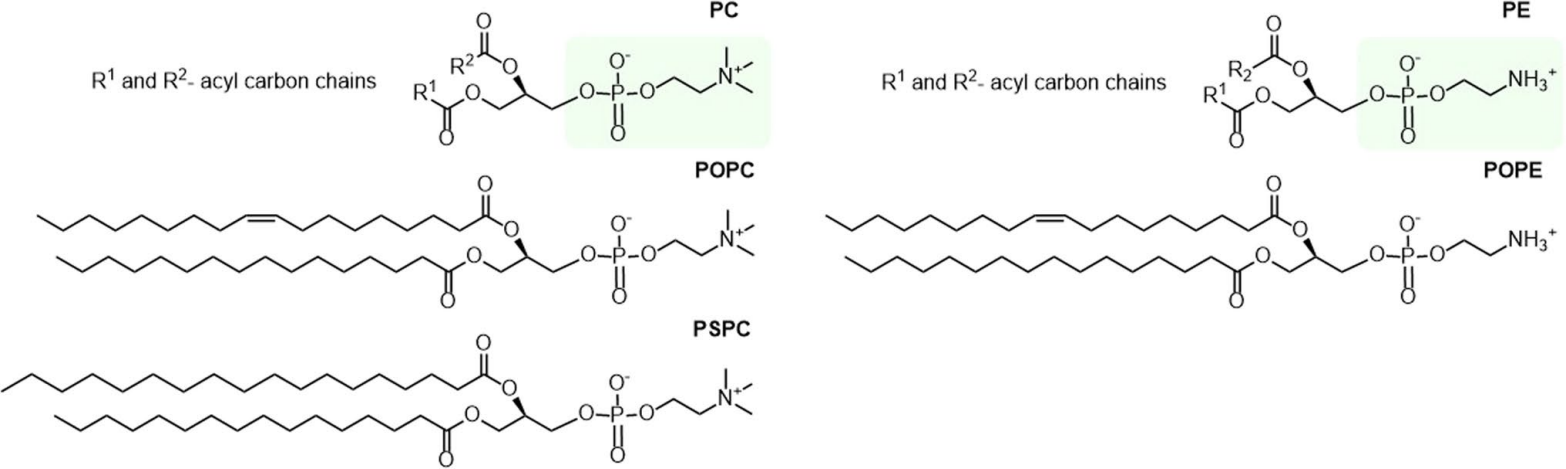
Structures of lipid classes PC and PE, and the lipids POPC, PSPC, and POPE. The headgroups of PC and PE lipids are marked with a green box

Here, POPC, a ubiquitous phospholipid belonging to the PC class, was chosen as the main target molecule for study. In addition, the corresponding saturated PC (1-palmitoyl- 2-stearoyl-*sn*-glycero- 3-phosphocholine, PSPC) and the monounsaturated PE (1-palmitoyl- 2-oleoyl-*sn*-glycero- 3-phosphoethanolamine, POPE) were studied. POPC, PSPC, and POPE are commercially available and naturally found in eukaryotic cell membranes. The general structure of PC and PE lipids and the structures of the lipids of interest in this work are presented in Fig. 1. The aim of this work was to identify novel chlorinated phospholipids, using liquid chromatography (LC) coupled to mass spectrometry (MS), employing unit- (MS, MS/MS) and high-resolution (HRMS, MS/HRMS) tandem MS. The experiments were carried out using single-lipid model systems and pig lung samples.

## Experimental section

All described experiments were repeated at least once, with comparable or practically identical results.

### Materials and reagents

POPC (> 99%), PSPC (> 99%), and POPE (> 99%) were obtained from Avanti Polar Lipids (Alabaster, AL, USA); chlorine gas (500 parts per million (ppm)) from Messer Austria GMH (Gumpoldskirchen, Austria); and formic acid (FA) for LC–MS (98–100%) and phospholipase C (PLC) from *Clostridium perfringens* from Merck (Burlington, MA, USA). Acetonitrile (ACN), dichloromethane (DCM), isopropanol (IPA), and methanol (MeOH) were obtained from Honeywell Fluka (Loughborough, Leicestershire, UK). Sodium hydroxide (NaOH) and chloroform were obtained from Thermo Fisher Scientific (Loughborough, Leicestershire, UK). Ammonium formate (NH_4_HCO_2_) was purchased from Sigma-Aldrich (St. Louis, MO, USA). Concentrated HCl, sodium chloride (NaCl), calcium chloride (CaCl_2_), Tris-base, and bovine serum albumin were purchased from VWR International (Radnor, PA, USA). Deuterated MeOH (d_4_-MeOH) and chloroform (d_1_-chloroform) were obtained from Eurisotop (Saint-Aubin, France). All chemicals and reagents were of analytical grades or higher. The water used in this study was purified using a Direct-Q^®^ 3 UV system from Millipore (Darmstadt, Germany). A lung from a freshly slaughtered pig was obtained from a local butcher (Tilateurastamo Kiven Säästöpossu, Karkkila, Finland), cut into smaller pieces, and stored at − 80 °C until analysis.

### Instrumentation

The LC–MS and LC–MS/MS analyses were performed with an Acquity I-Class UPLC^®^ (Waters™; Milford, MA, USA) coupled to a Xevo^®^ TQ-XS triple quadrupole mass spectrometer (Waters™). LC-HRMS and LC–MS/HRMS analyses were performed with a Thermo Scientific Dionex Ultimate 3000 UHPLC (Germering, Germany), coupled to a Thermo Scientific Orbitrap Fusion™ mass spectrometer (San Jose, CA, USA). LC separation was achieved using ACQUITY UPLC^®^ BEH HILIC (2.1 × 100 mm, 1.7 µm; Waters™) and XBridge^®^ BEH C18 (2.1 × 100 mm, 2.5 µm; Waters™) columns. For hydrophilic interaction liquid chromatography (HILIC) analysis, we used the following eluents: ACN (A), and 55 mM NH_4_HCO_2_ in 50% ACN in ultrapure water (B). The gradient was run from the starting point of 2% B for 1 min to 20% B at 10 min. The B eluent was linearly increased to 40% over 2 min and held for 3 min. The B ratio was linearly reduced to 2% B within 1 min, and the column was equilibrated for 4 min at 2% B, for a total run time of 20 min. The column oven was set to 45 °C. The flow rate was 0.5 mL/min and the injection volume was 5 µL. For analysis with the reverse-phase C18 column, these LC eluents were used: 0.1% FA and 5 mM of NH_4_HCO_2_ in 60% ACN (A); 0.1% FA and 5 mM of NH_4_HCO_2_ in 80% IPA and 20% ACN (B). The column oven was set to 60 °C. The chromatographic gradients consisted of initial conditions of 5% B at 0.6 min before linearly increasing to 100% B over 13 min. It was then held for 4 min before the B ratio was reduced to 5% B within 1 min, and the column was equilibrated for 2 min at 5% B. The flow rate was 0.6 mL/min, and the injection volume was 5 µL.

For the MS and MS/MS analyses, electrospray ionization (ESI) in positive mode was used. The MS parameters were set as follows: capillary voltage 3 kV, cone voltage 20 V, source temperature 150 °C, desolvation temperature 500 °C, cone gas flow 150 L/h, desolvation gas (nitrogen, N_2_) flow 1000 L/h, collision gas (argon, Ar) flow 0.15 mL/min. MassLynx (Waters™, version 4.2) was used for data acquisition and analysis. For the precursor ion-scanning analyses (parent ion scanning of the mass-to-charge ratio (*m*/*z*) 184), a collision energy of 33 V was used. The collision gas was Ar. For the HRMS and MS/HRMS analyses, ionization was performed using heated electrospray ionization (HESI) in the positive ion mode, with N_2_ as the spray gas and helium (He) as the collision gas. The instrument parameters were set as follows: spray voltage 4500 V, source temperature 350 °C, ion transfer tube temperature 230 °C, sheath gas 40 arbitrary units (Arb), auxiliary gas 15 Arb, and sweep gas 0 Arb. Exact mass measurements were carried out in the *m*/*z* range of 600–1000 with RF lens at 60% and a resolution of 60,000 in the full scan method and 120,000 in the product ion scan method. The instrument was calibrated to ≤ 5 ppm mass accuracy using external calibration. Stepped high-energy collisional dissociation (HCD) with 15%, 30%, and 45% energies was used to fragment the ions. Xcalibur™ (Thermo Scientific, version 4.5) was applied for data acquisition and analysis.

### Chlorination of phospholipids

POPC (7.6 mg) in chloroform was transferred to a glass tube with a screw cap (Kimax^®^; Merck) and concentrated to dryness under a stream of N_2_ in a TurboVap^®^ LV II (Caliper Life Sciences, Hopkinton, MA, USA), at 7.5 psi and 30 °C. Next, 1 mL of water was added to the dried POPC, resulting in a 10 mM sample. The aqueous 10 mM sample was bath-sonicated (Branson 3200; Branson Ultrasonics; Brookfield, CT, USA) for 1 h at room temperature to form a POPC vesicle solution. The vesicle solution was exposed to chlorine gas, with a 500 ppm concentration, for 5 min by submerging the gas stream (approximately 1 L/min) in the liquid to produce bubbling. The chlorinated POPC was extracted using a modified Folch’s extraction method: 1.5 mL of MeOH and 3 mL of DCM were added, and the sample was vortexed for 10 s. The tube was then shaken for 1 min before being centrifuged for 5 min at 1000 revolutions per minute (rpm). The DCM (lower) layer was collected and dried in a TurboVap^®^ LV II (as above). The dry extract was reconstituted with 1 mL of ACN. An aliquot of the reconstituted sample was further diluted with ACN to a concentration equivalent of 50 µM POPC starting material for analysis by LC–MS-based techniques. Unit-resolution LC–MS was used to screen for novel chlorinated PCs. LC-HRMS and LC–MS/HRMS were used to measure the accurate masses and fragmentation patterns of the novel compounds. For comparison, a chlorination of 10 mM POPC in chloroform was similarly performed. After the chlorination, the chloroform was evaporated (TurboVap^®^ LV II, as above), and the sample was subsequently reconstituted in 1 mL of ACN. An aliquot of the sample was diluted and analyzed as described above. The chlorination experiments were repeated using PSPC (7.6 mg) and POPE (7.2 mg) dissolved in chloroform and in water. All prepared samples were stored at − 20 °C until analysis. Untreated control samples were prepared similarly.

### Pig lung tissue chlorination

A surface piece of approximately 0.5 g was cut from pig lung, which had previously been frozen and stored at − 80 °C. The thawed lung sample was placed in a 10-mL borosilicate glass tube with a screw cap (Kimax^®^). The lung sample was exposed to 500 ppm chlorine gas flow for 5 min, shaken, and then exposed for an additional 5 min. The chlorine gas flow in the exposure was approximately 1 L/min. The surface lipids of the exposed lung tissue sample were collected by submerging the sample in 1 mL of water, followed by a modified Folch’s extraction protocol (see the “Chlorination of phospholipids” section). The dried extract was reconstituted in 400 µL of ACN for analysis by LC-HRMS and LC–MS/HRMS. An un-exposed control lung sample was identically prepared.

### Mild alkaline hydrolysis

A 0.1 mL sample of a previously chlorinated 10 mM POPC solution in ACN (chlorinated in water, see the “Chlorination of phospholipids” section) was evaporated to dryness in a glass tube (Kimax^®^) under N_2_ flow (TurboVap^®^ LV II, see the “Chlorination of phospholipids” section). 0.5 mL MeOH and 0.5 mL chloroform were added to the evaporation residue before the tube was shaken. One hundred sixty-seven microliters of 0.3 M NaOH in MeOH/water (freshly prepared as 96:4 by volume) was added before vortexing thoroughly and incubating in the dark for 4 h. The mixture was neutralized by adding 100 µL of 0.3 M HCl in MeOH (prepared from concentrated HCl) before vortexing. Following this, 1.5 mL chloroform, 0.16 mL MeOH, and 0.75 mL of water were added to the mixture before vortexing and centrifuging at 2000 rpm for 10 min. The upper phase was collected and analyzed directly by LC–MS. The lower phase was washed once with 2 mL of Folch’s theoretical upper phase containing chloroform, MeOH and water (3:48:47 by volume). The mixture was shaken and centrifuged again before the upper phase was discarded. The lower phase was dried in a TurboVap^®^ LV II (see the “Chlorination of phospholipids” section) and reconstituted with 1 mL of ACN. An aliquot of the reconstituted sample was further diluted with ACN to a concentration equivalent of 50 µM POPC starting material, and analyzed by LC–MS.

### Enzymatic cleavage by phospholipase C

For PLC treatment, 0.1 mL of a previously chlorinated 10 mM POPC solution in ACN (chlorinated in water, see the “Chlorination of phospholipids” section) was evaporated to dryness in a glass tube (Kimax^®^) under N_2_ flow (TurboVap^®^ LV II, see the “Chlorination of phospholipids” section), and 0.5 mL of PLC buffer (140 mM NaCl, 10 mM Tris–HCl, 10 mM CaCl_2_, 0.1% bovine serum albumin, pH 7.2) was added to form an approximate concentration of 2 mM chlorinated POPC solution. The lipids were emulsified by vortexing and PLC (solubilized in PLC buffer) was added to an approximate concentration of 5 units/mL. The sample was incubated overnight while shaking at 37 °C. A concentration matched, PLC-untreated sample was used as a control. The samples were extracted using a modified Folch’s extraction protocol (see the “Chlorination of phospholipids” section). The dry extracts were reconstituted in 1 mL of ACN. An aliquot was further diluted with ACN to a concentration equivalent of 100 µM POPC starting material, prior to analysis by C18 LC–MS and LC–MS/MS.

## Results and discussion

### POPC vesicles chlorinated in water

**Fig. 2:**
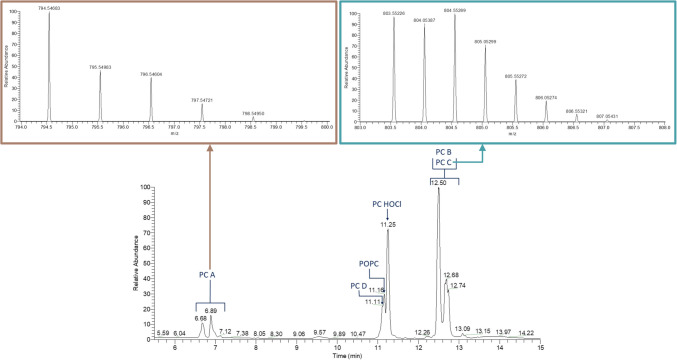
TIC of a POPC sample chlorinated in water separated by HILIC and the isotopic patterns of PC A (left) and PC C (right). The chlorinated PCs elute in three structure-dependent groups: PCs chlorinated in the glycerol backbone (e.g., PC A), PCs chlorinated in the acyl chains (e.g., PC D), and dimeric chlorinated PCs (e.g., PC C). The isotope peaks in the isotopic pattern of PC C are 0.5 units apart, which is typical for doubly charged ions

The POPC samples chlorinated as vesicles in water were first analyzed with LC–MS and LC-HRMS using a C18 column in positive polarity, before further analysis with HILIC separation. Chlorinated PCs were not detected in the untreated control sample. Five chlorinated PCs were found in the chlorine-exposed sample: PC chlorohydrin (PC HOCl), PC dichloride (PC D), and analytes with theoretical accurate *m*/*z* values of 794.54611 (PC A), 777.56560 (PC B), and 803.55139 (PC C) (Table 1). Evaluation of the isotopic patterns of PC A–C revealed that PC A and B contain one chlorine atom and PC C two chlorine atoms (Fig. S10–S12). The ^13^C peaks of PC B and C differed by approximately 0.5 mass units from the monoisotopic ion peak (Fig. 2, and Fig. S11 and Fig. S12 in the Electronic Supplementary Material (SM)), indicating that the analytes are doubly charged.

**Table 1:** Abbreviations of the chlorinated lipids, their predicted formula, and theoretical and measured *m*/*z*. Masses corresponding to the lipids marked with an asterisk (*) were detected in the chlorinated lung sample

**Lipid**	**Predicted formula**	***z***	**Theoretical *m***/***z***	**Measured *m***/***z***	**Error (ppm)**
Chlorinated PCs					
PC HOCl*	C_42_H_84_ClNO_9_P^+^	1	812.55667	812.55767	1.22240
PC A*	C_42_H_82_ClNO_8_P^+^	1	794.54611	794.54683	0.90686
PC B	C_84_H_165_ClN_2_O_16_P_2_^2+^	2	777.56560	777.56672	1.44039
PC C*	C_84_H_166_Cl_2_N_2_O_17_P_2_^2+^	2	803.55139	803.55226	1.08539
PC D*	C_42_H_83_Cl_2_NO_8_P^+^	1	830.52279	830.52391	1.34962
PC E	C_42_H_82_Cl_3_NO_8_P^+^	1	864.48381	864.48357	− 0.27991
PC F*	C_84_H_165_Cl_3_N_2_O_16_P_2_^2+^	2	812.53445	812.53392	1.81570
PC G	C_84_H_164_Cl_4_N_2_O_16_P_2_^2+^	2	829.51496	829.51399	− 1.17469
PC H	C_84_H_163_Cl_5_N_2_O_16_P_2_^2+^	2	846.49548	846.49431	− 1.37940
PC I	C_84_H_162_Cl_6_N_2_O_16_P_2_^2+^	2	863.47599	863.47391	− 2.40630
PC J*	C_42_H_81_Cl_4_NO_8_P^+^	1	898.44484	898.44468	− 0.18223
PC K	C_42_H_81_Cl_2_NO_8_P^+^	1	828.50714	828.50808	1.13393
PC L	C_42_H_80_Cl_3_NO_8_P^+^	1	862.46816	862.46855	0.44985
Chlorinated PEs					
PE HOCl	C_39_H_78_ClNO_9_P^+^	1	770.50972	770.51004	0.41349
PE A	C_39_H_77_Cl_2_NO_8_P^+^	1	788.47584	788.47738	1.95313
PE B	C_39_H_77_Cl_2_NO_9_P^+^	1	804.47075	804.47082	0.08136
PE C	C_39_H_76_Cl_3_NO_8_P^+^	1	822.43686	822.43597	− 1.08806
PE D	C_39_H_76_ClNO_8_P^+^	1	752.49916	752.49996	1.06970
PE E	C_39_H_75_Cl_2_NO_8_P^+^	1	786.46019	786.46131	1.42531
PE F	C_39_H_74_Cl_3_NO_8_P^+^	1	820.42121	820.42299	2.16989

**Fig. 3:**
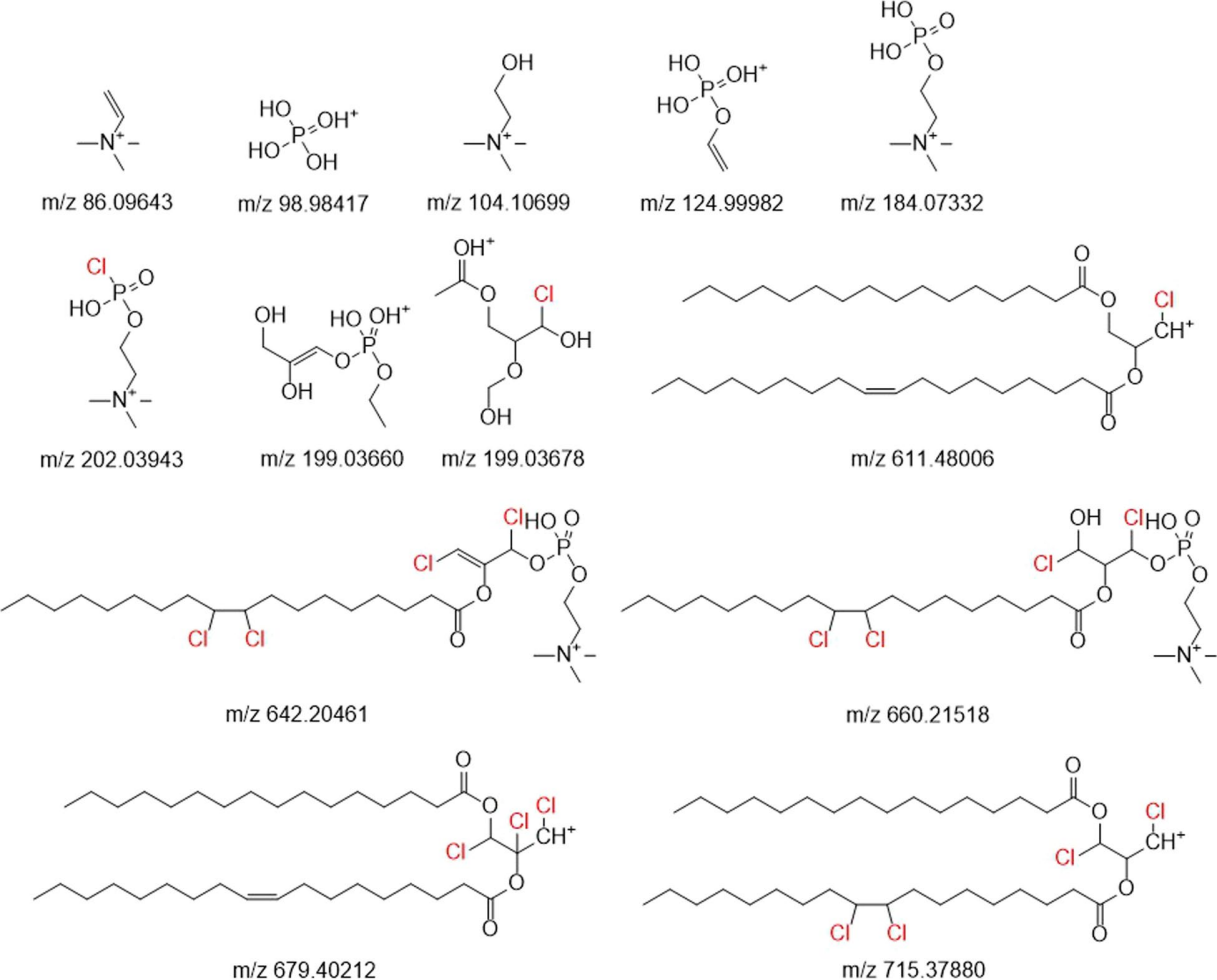
Proposed structures of selected fragments of the chlorinated PCs

A chlorinated PC with an accurate mass of 794.54611 has previously been reported in POPC samples incubated with HOCl [17]. Based on the fragmentation pattern, the authors suggested that the analyte was a POPC chlorinated in one of the acyl chains without opening the double bond. Here, LC–MS/HRMS with stepped HCD fragmentation was used to fragment PC A–C to obtain structural information. All three lipids produced typical phosphocholine fragments (*m*/*z* 86.09643, 98.98417, 104.10699, 124.99982, and 184.07332, proposed structures in Fig. 3) [25], indicating that the chlorine substituent is not located in the headgroup. This is also supported by the chlorinated fragments consisting of the glycerol backbone and one or two acyl chains (e.g., *m*/*z* 977.61184, 794.54611, 611.48006, 556.31644, and 373.25040) (Tables S3, S5, and S6; Fig. S21–S23 in the SM). Interestingly, PC A and PC C produced a fragment ion with an *m*/*z* of 202.03943 (Fig. 3), corresponding to the phosphocholine headgroup in which one of the hydroxyls in the phosphate has been replaced with a chlorine atom (Tables S3 and S6; Fig. S31 and S33 in the SM). This fragment is presumably produced by re-arrangement reactions during the collisional fragmentation. For the re-arrangement reaction to occur, the chlorine atom must be in close proximity to the phosphate, i.e., either in the headgroup or in the glycerol backbone. Since PC A and C produce the aforementioned phosphocholine fragments, it is unlikely that the chlorine substituent is in the headgroup. PC A also produced fragment ions with *m*/*z* 239.23694, 263.23696, and 199.03678. The first two mentioned are ions produced in the fragmentation of the un-chlorinated acyl chains. The last ion mentioned could potentially have the molecular formula C_6_H_12_ClO_5_^+^, which corresponds the chlorinated glycerol backbone (Fig. 3).

Based on the aforementioned and other fragments of PC A–C (and the other detected chlorinated PCs discussed in the “POPC chlorinated in chloroform” section), we propose that the chlorine substituent is located in the glycerol backbone, which differs from previously made conclusions on the structure of the chlorinated PC with the *m*/*z* value of 794.54611 [17]. This hypothesis is supported by previous studies where chlorination of ethers resulted in the addition of chlorine in the α- and β-positions. While the α-carbon of the carbonyl can also be chlorinated [26], the glycerol backbone might be a more available reaction site, hence leading to the observed products. Selected fragments of the chlorinated PCs are presented in Fig. 3. The exact position of the chlorine atom(s) in the glycerol backbone cannot be concluded from the data available. However, multiple peaks were detected for many of the analytes, suggesting that multiple structural isomers of the chlorinated PCs were formed during the chlorination.

Based on the two charges and the fragmentation (e.g., *m*/*z* 489.30972 = PC A with an additional phosphocholine headgroup), we propose that PC B and C are chlorinated, dimeric peroxy-diphospholipids in which one of the lipid monomers is PC A. The second lipid in the dimers is POPC or PC HOCl in PC B and PC C, respectively.

**Fig. 4:**
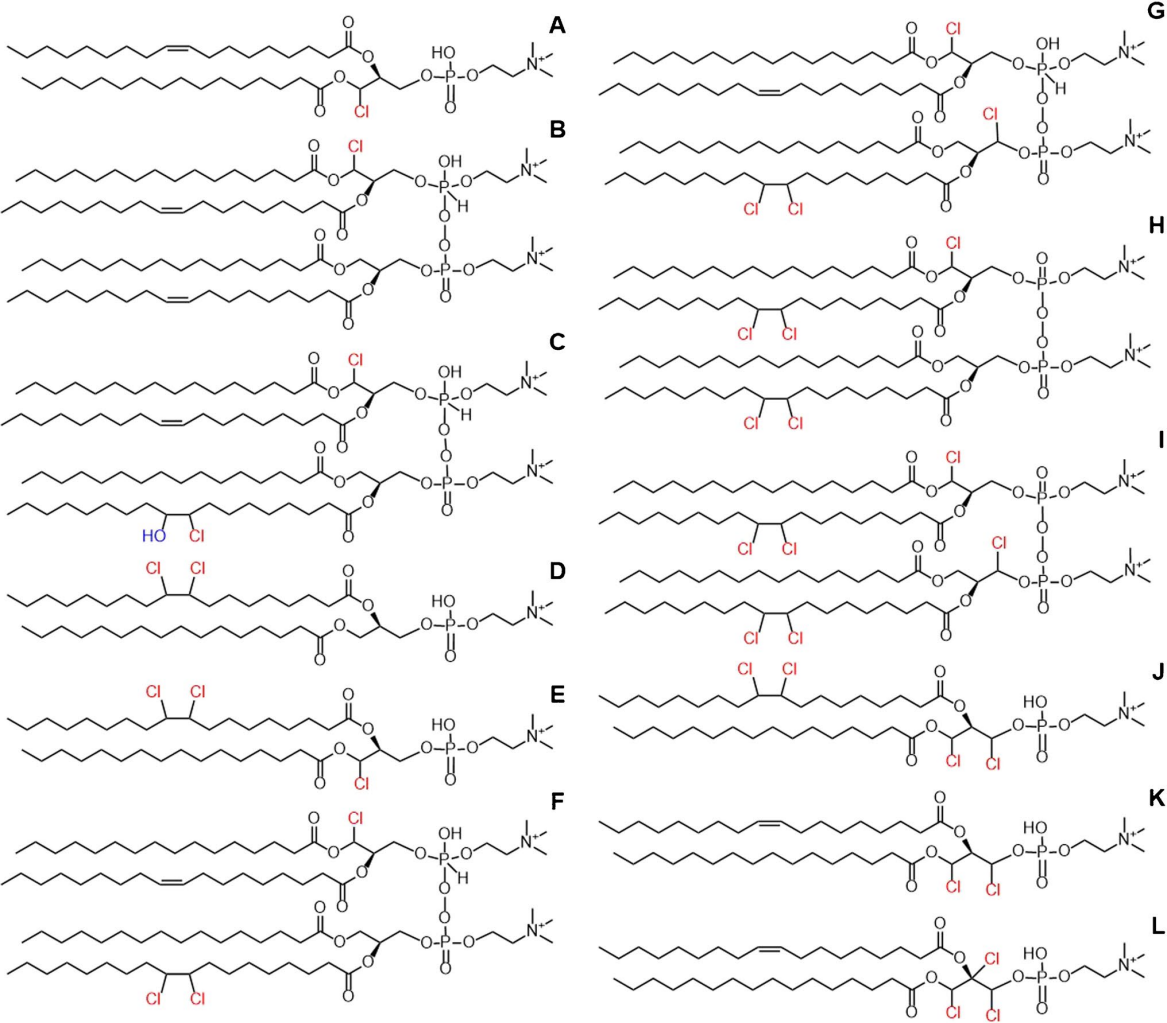
Proposed structures of PC HOCl and PC A–L, as detected by LC-HRMS. The chlorine substituent(s) may be located in any of the carbons in the glycerol backbone

The structures of PC HOCl and PC A–D (and PC E–L, which are discussed in the “POPC chlorinated in chloroform” section) are presented in Fig. 4. The isotopic patterns of the analytes are presented in Fig. S9–S13, and the fragmentation patterns of the analytes can be found in Tables S1, S3, S5, S6, and S8 in the SM. The proposed structures for the detected fragments are presented in Fig. S30–S34 in the SM.

To obtain further structural information, the samples were also analyzed with HILIC LC-HRMS. In C18, retention is mainly based on hydrophobicity; hydrophobic analytes are retained more strongly than hydrophilic analytes. The hydrophobicity of PCs is determined by the lengths and degrees of unsaturation of the alkyl chains [27]. The retention of analytes in HILIC columns is more complicated, but generally polar and ionic compounds are retained more effectively [28]. The polarity of PCs is mainly determined by the phosphocholine headgroup [27]. PC B and C show the strongest retention on HILIC, presumably because they both have two charged cholines which like to interact with the hydrated zone and charges of the stationary phase. The retention times suggests that PC A is the least polar/ionic of the four chlorinated PCs. An electronegative chlorine atom in the glycerol backbone might decrease the net-polarity of PC A, e.g., by attracting the quaternary amine or by changing the charge distribution in the headgroup. This can, in turn, result in changes in the PCs’ interactions with solvent molecules and the stationary phase and shifting of the retention times. Further research is needed to determine how the chlorine modification in the glycerol backbone affects the structure and properties of PCs. Nevertheless, the charge and chlorine substituent position–dependent retention in HILIC separation were useful in determining the structure of the chlorinated PCs and PEs discussed in the “POPC chlorinated in chloroform” and “Alkaline and enzymatic degradation of chlorinated POPC” sections.

Experiments using ESI negative polarity scanning were also performed with C18 LC–MS. Of the chlorinated analytes, only PC HOCl and PC D were detected as negative ions ([M-CH_3_]^−^, *m/z* 796.5 and *m/z* 814.5, respectively, data not shown). In the case of the glycerol-chlorinated lipids, the inability to produce negatively charged ions may have been due to changes in ionizability, caused by the very electronegative chlorine atom near the headgroup. Similarly to un-chlorinated PCs, PC HOCl and PC D (which contain chlorine atoms only in their acyl chains, see Fig. 4) were negatively ionized, through a loss of a methyl group and subsequent neutralization of the quaternary ammonium choline [25].

For further structural investigation of the position of the chlorine substituents and the dimeric lipids, a sample was prepared for ^1^H nuclear magnetic resonance (NMR) analysis. A 1 mL sample containing 10 mM of POPC in water was chlorinated, after which the solvent was changed to a mixture of d_4_-MeOH and d_1_-chloroform (1:5, 600 µL) and analyzed by ^1^H NMR. An untreated control sample of POPC was prepared and analyzed similarly. The results of the analysis were, however, inconclusive due to the complexity of the chlorinated sample. For easier interpretation, the various chlorinated molecular species would need to be isolated, which is planned for future experiments.

### POPC chlorinated in chloroform

Samples prepared by chlorination of POPC dissolved in chloroform and untreated control samples were also analyzed with LC-HRMS. The samples were prepared in an attempt to determine whether an aqueous environment is necessary for the production of the novel lipids, as well as to study whether a water-free environment produces additional chlorinated PCs that could give more structural insight into the location of the chlorine substituents in the novel lipids.

No chlorinated lipids were detected in the control sample. Most of the chlorinated lipids observed in the samples chlorinated in water were detected in the chloroform samples as well; however, eight additional chlorinated PCs were also identified (Table 1, Fig. 4). The isotopic patterns and fragmentation measured by LC-HRMS and LC–MS/HRMS indicated that four of the eight additional compounds were peroxy-diphospholipids (PC F, G, H, and I) (Fig. S15–S18 in the SM). The other four had chlorine atoms attached to their glycerol backbone (PC K and L), or to both the glycerol backbone and the acyl chain (PC E and J). They produce fragments containing the glycerol backbone with one or both acyl chains, which support the proposed chlorination site being located in the glycerol backbone (selected structures in Fig. 3). For example PC E and J produce fragments corresponding to ions resulting from the loss of the saturated acyl chain and the loss of the phosphocholine (*m*/*z* 681.41777, 626.25415, 715.37880, and 660.21518) (selected structures in Fig. 3). These fragments show that the chlorine atoms must reside in the oleoyl-chain and/or the glycerol backbone. Since the double bonds in both molecules have been saturated with chlorine, the only logical location for the remaining chlorine atoms is the glycerol backbone. The corresponding fragments (*m*/*z* 645.44109, 590.27747, 643.42544, and 624.23850) and the retention time of PC K and L support the conclusion. The fragmentation patterns and isotopic patterns of the analytes can be found in Tables S10, S11, S13–S16, S18, and S19 and Fig. S14, S19–21, and S35–S42 in the SM. The proposed structures for all chlorinated PCs are presented in Fig. 4, and their masses are listed in Table 1. The measured accurate masses of all chlorinated PCs did not differ more than ± 2.5 ppm from the theoretical value.

Chlorination of POPC dissolved in chloroform resulted in the production of individual lipids with higher degrees of chlorination than obtained in the chlorination of an aqueous POPC-sample. In water, the differences in hydrophobicity of the headgroup and the alkyl chains cause the lipids to arrange in bilayer vesicles, especially when energy is introduced into the system e.g. through sonication. Although chlorine is a small molecule, the ordered structure of the bilayer vesicles may limit access to the unsaturated sites, and electrostatic and other physical interactions with headgroups nearby may restrict reactivity at the glycerol backbone. In addition, the reaction of chlorine with water (producing HOCl and HCl [29]) competes with the chlorination of the lipids. In chloroform, the lipids are ordered as inverted micelles, in which the acyl chains are oriented towards the solvent. There are fewer competing reactions with the solvent, resulting in more polychlorinated mono- and dimeric-PCs. Due to the differing behavior of lipids and reactions of chlorine in chloroform and in aqueous systems, the chloroform experiments are poor simulations of chlorination reactions in biological systems. Therefore, the suitability of the polychlorinated PCs identified in the chloroform experiments as biomarkers should be critically evaluated. Some polychlorinated PCs may also be formed in tissues (aqueous systems) from chlorine produced in the spontaneous degradation of externally introduced or metabolically produced HOCl. Dichlorination of unsaturated sites through natural metabolism has been demonstrated by the detection of dichlorinated cholesterol in tissue [4, 14]. However, when chlorine is used as a CW, the concentrations are very high. Therefore, it is feasible that polychlorinated lipids, such as the PCs identified in the chloroform experiments, would be more prominent in biomedical samples of chlorine attack victims, than in samples from patients suffering from immunological stress conditions.

Chromatograms of the POPC sample chlorinated in chloroform and analyzed with the different separation methods (C18 and HILIC) are presented in Fig. S1 and Fig. S2 in the SM. From the chromatograms, one can note that the chlorinated PCs elute in three groups on both stationary phases. The first eluting group consists of the PCs chlorinated only at the glycerol backbone (PC A, K, and L). The POPCs chlorinated at the acyl chain double bond (PC D, E, and J) are eluted next. With the HILIC column, PC HOCl elutes in the second group. With the C18 column, the increased interactions with the mobile phase cause the PC HOCl to elute before the first group of chlorinated lipids. The peroxy-diphosphocholines (PC C, F, G, H, and I) are eluted last on both stationary phases. This elution behavior taking place in groups is useful when determining the possible location of the chlorine atom(s) in the lipids. Multiple chromatographic peaks can be detected for most of the chlorinated PCs. This may have resulted from the analytes having several isomers. No conclusions from the structural differences could be drawn from the mass-spectrometric fragmentation patterns of the isomers.

### Mechanism of dimerization

As can be seen from the structures presented in Fig. 4, all chlorinated peroxy-diphosphocholines (PC B, C, F, G, H, and I) have at least one chlorine substituent in the glycerol backbone. Our hypothesis is that the dimerization is initiated by the formation of a PC chlorinated at the glycerol, e.g., PC A. The chlorine substituent changes the three-dimensional structure and electron distribution in the headgroup, allowing dimerization with other lipids in its proximity. After dimerization, the lipids can be further chlorinated at the glycerol and remaining unsaturated sites in the acyl chains. The literature describes the production of peroxy-diphosphates by electrolysis of phosphate solutions containing added halide or pseudohalide anions [30]. However, the exact mechanism of the chlorination and subsequent dimerization of phospholipids cannot be determined from our data. Further experiments are needed to elucidate the mechanisms involved.

### Alkaline and enzymatic degradation of chlorinated POPC

In an attempt to verify the position of the chlorine substituent in PC A, chlorinated aqueous POPC samples were treated with mild alkaline hydrolysis or with PLC and analyzed with positive full-scan LC–MS and positive precursor ion scanning of *m*/*z* 184 (LC–MS/MS). Positive precursor ion scanning of *m*/*z* 184 is a PC-specific MS/MS-scanning mode [31]. The rationale was to investigate whether alkaline or enzymatic degradation would result in degradation products, that would confirm the position of the chlorine in PC A (e.g. a chlorinated glycerophosphocholine or a chlorinated diacylglycerol (DAG)). Mild alkaline hydrolysis degrades PCs to fatty acids and a glycerophosphocholine [32]. The free fatty acids were detected in alkaline-treated chlorinated POPC samples; however, the chlorinated glycerophosphocholine was not detected (data not shown). No chlorinated species were found, which might be a result of the alkaline conditions causing elimination of the chlorine substituent.

PLC is an enzyme that is capable of releasing the phosphocholine headgroup of PCs, yielding a corresponding diacylglycerol lipid [33, 34]. PLC-treatment was able to liberate phosphocholine from POPC, PC HOCl, and PC D, i.e., monomeric PCs without a chlorine substituent in the glycerol backbone (Fig. 1 and Fig. 4). The liberation was evidenced by the loss of respective PC signals (Fig. S50 in the SM) and the appearance of the corresponding DAG in the organic phase (Fig. S52 in the SM). Interestingly, PC A, which putatively contains a chlorine substituent in the glycerol backbone, was seemingly resistant to PLC degradation, as evidenced by apparently no loss of signal intensity between PLC-treated and -untreated samples (Fig. S50 and Fig. S51 in the SM). Masses corresponding to a DAG released from PC A could not be observed in any of the PLC-treated samples; however, the signal may have been below the limit of detection of the instrument.

The signals of PC B and PC C also disappeared after PLC-treatment (Fig. S53 in SM), but no logical degradation products could be identified for these compounds. It is unlikely that the PLC-enzyme was able to cleave the dimers (PC B and C) back into individual lipids, because the signal for PC A in the sample did not increase compared to that of an untreated sample (Fig. S51 in the SM). Nevertheless, the resistance of PC A to degradation by PLC further suggests that the chlorine is located in a position close to the lipid headgroup, possibly hindering PLC from accommodating the lipid headgroup at its active site. The resistance to degradation could also have biological implications. Hindering cellular PC catabolism could affect the generation of lipid signaling molecules (e.g., phosphatidic acid and DAG) that are involved in a range of cellular processes [35].

### Chlorinated PSPC and POPE

To determine whether other phospholipids in addition to POPC react similarly to chlorination, PSPC and POPE were evaluated. They were dissolved in chloroform or sonicated in water, and subsequently chlorinated and analyzed with LC-HRMS and LC–MS/HRMS, using HILIC separation. Chlorinated lipids were not detected in the control samples that had not been exposed to chlorine gas. Interestingly, no chlorinated PSPC-related lipids were found in any of the samples. The saturated acyl chains of PSPC may have formed more rigid membrane structures, sterically obstructing chlorine from reaching the glycerol backbone. The presence of unsaturated acyl chains (such as the oleoyl in POPC) is seemingly required for sufficient micellar and vesicle bilayer fluidity, and subsequent chlorination of the glycerol backbone. Further experiments are needed to verify this assumption.

**Fig. 5:**
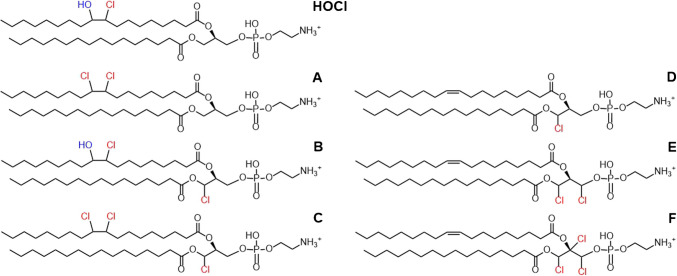
Proposed structures of PE HOCl and PE (**A**–**F)**, as detected by LC-HRMS. The chlorine(s) may be located in any of the carbons in the glycerol backbone

Seven chlorinated PEs were detected in the chlorine-exposed POPE samples (Table 1, Fig. 5). The isotopic patterns and fragmentation patterns of the chlorinated PEs are presented in Fig.S23–S29 and S43–S48 and Tables S20–S28 in the SM. The seven chlorinated PEs included the chlorohydrin (PE HOCl), the chlorine saturated PE (PE A), and five PEs chlorinated at the glycerol backbone (PE B–F). The proposed structures of PE A–F are presented in Fig. 5. All seven chlorinated PEs were detected in the POPE sample chlorinated in chloroform. PE F was not produced, and only small amounts of PE D was formed when POPE was chlorinated in water. This difference is presumably caused by the solvent effects described in the “POPC chlorinated in chloroform” section. The accurate masses of some dimeric chlorinated PEs were detected, but their low quantity did not allow reliable identification using isotopic patterns and LC–MS/HRMS analysis. The experiment with POPE showed that other unsaturated phospholipid classes can also be chlorinated in the glycerol backbone, but the reactivity of different lipid classes varies.

The chromatograms of the chlorinated POPE samples are presented in Fig. S5–S7 in the SM. The retention of the chlorinated PEs follows the trends observed for the chlorinated PCs (section “POPC vesicles chlorinated in water”). However, there is a notable difference in the retention of the backbone-chlorinated PEs (PE D–F) and the PCs (PC A, K, and L) using HILIC separation (Fig. S2, S4, S6, and S7 in the SM). The backbone-chlorinated PCs eluted near 7 min, while their PE analogues eluted at 1.6 min (PE D) and 0.6 min (PE E and F). This demonstrates that the addition of chlorine atoms to the backbone of POPE reduces the polarity of the resulting PE more compared to the corresponding PCs. The methyl substituents on the amine make the headgroup of PCs bulkier, which may hinder their interaction with the chlorine substituents in the glycerol backbone and the 3D-structure of the chlorinated PCs. The much smaller unsubstituted amine in PEs may allow the headgroup to interact more efficiently with the chlorine atoms in the glycerol backbone, making the molecule less polar (almost non-polar in the case of PE E and F).

### Chlorinated pig lung sample

A sample of pig lung was chlorinated in vitro to determine whether the novel chlorinated lipids could be used as biomarkers for chlorine gas exposure. Lung tissue was selected as a chlorination target because inhalation is one of the main exposure routes for chlorine gas. The extracts of the chlorinated and untreated pig lung samples were analyzed using LC-HRMS for chlorinated PCs. Since both POPC and POPE were naturally present in the lung samples, we searched for the accurate mass of PC HOCl, PC A–L, PE HOCl, and PE A–F in the total ion chromatograms (TICs) (Table 1). The TICs were also evaluated for the presence of other chlorinated PCs. The chlorinated PCs were distinguished from the unmodified PCs based on the accurate masses and their isotopic patterns; the patterns had to indicate that ^37^Cl is present in the molecule, and the monoisotopic peak and the ^13^C-peak of the dimeric compounds had to be approximately 0.5 mass units apart.

No chlorinated lipids were detected in the untreated control sample. As expected based on previous studies [14–18], PC HOCl and PC D were found in the chlorinated lung sample (Tables S2 and S9 in the SM). PC A and three of the novel chlorinated lipids (PC C, F, and J) were also found in the chlorinated lung sample. The fragmentation patterns of the analytes measured with LC–MS/HRMS matched with the fragmentation of the reference chemicals (Tables S4, S7, S12, and S17 in the SM), suggesting that the detected analytes are chlorinated PCs derived from POPC or structural isomers of POPC. Other chlorine-containing doubly charged PCs (based on the isotopic pattern) were also observed in the chlorinated lung sample at 12.4–12.8 min, which is a typical retention time for chlorinated dimeric PCs using HILIC separation. This indicates that chlorine exposure causes dimerization of other PC molecular species as well. Logically, the structures of the chlorinated mono-PCs and peroxy-diphospholipids formed are dependent on the lipids present in the tissue upon chlorine exposure. Identification of the exact structure of all the various mono- and dimeric chlorinated lipids detected in the lung sample is difficult, due to the vast number of structurally distinct PCs (and other lipids) originally present in the sample.

We propose that all chlorinated lipids identified by us here could potentially be used as biomarkers for chlorine gas exposure, alongside with the previously reported chlorohydrins and dichlorides. However, the compounds with more than two chlorine substituents may only be found in victims exposed to high levels of chlorine gas. The chlorinated derivatives of POPC may be particularly suitable as biomarkers of chlorine gas exposure, due to the ubiquitous nature of this lipid. Nevertheless, since the overall lipid composition is tissue-dependent, other suitable lipid alternatives should be explored. Further effort should be directed towards determining whether glycerol-chlorinated lipids and chlorinated peroxy-diphospholipids can be detected in biomedical samples (e.g., BALF and NLF) in concentrations that allow verification of chlorine gas exposure. The risks of false positives due to natural chlorination by metabolically produced HOCl should also be assessed.

## Conclusion

Several biomarkers of chlorine gas exposure have previously been reported, including chlorinated tyrosine and phospholipid chlorohydrins, among others. These biomarkers may be also produced as a response to inflammation caused by disease and other stressful stimuli [4–6], making unambiguous verification of chlorine exposure problematic. As such, a more holistic approach to verification should be studied, where examination of the production of various biomarkers in response to chlorine gas and other stressful stimuli, such as disease, should be considered. As demonstrated by de Bruin-Hoegée et al. [12] and Jonasson et al. [13], machine learning would be a powerful tool for distinguishing chlorine gas exposure–specific lipid biomarkers from chlorinated species produced by other sources. In this work, we aimed on expanding upon the array of known biomarkers, in hopes that the combined knowledge may eventually lead to methods that can unambiguously prove cases of chlorine gas exposure in victims.

Here, we aimed at finding novel potential biomarkers for chlorine gas exposure, using POPC, PSPC, and POPE as study material. With LC-HRMS and LC–MS/HRMS techniques, 15 novel chlorinated lipids were detected and a new structure for the previously reported [17] chlorinated PC detected as *m*/*z* 794.54611 (PC A) was proposed. Five chlorinated PCs that had at least one chlorine atom attached to the glycerol backbone, and six peroxy-diphospholipids generated from the chlorinated PCs were found. Five PEs chlorinated at the glycerol backbone were also detected. No chlorinated PSPC-related PCs were detected, suggesting that at least one unsaturated site at the acyl chain of the phospholipid is needed for chlorination to occur. The comparatively smaller number of observed chlorinated PEs indicates that while other phospholipids can be chlorinated in a manner similar to that of POPC, the reactivity of various phospholipid classes may vary widely. To determine the relevance of the novel chlorinated lipids as biomarkers, chlorination experiments using pig lung were performed in vitro. In addition to the previously reported POPC chlorohydrin and dichloride, PC A and three of the novel chlorinated PCs (PC C, F, and J), or their structural isomers, were found in the chlorinated lung sample, showing that the chlorinated lipids discovered here by us are potential biomarkers for chlorine gas exposure.

To the best of our knowledge, this is the first time putative peroxy-diphospholipids and lipids chlorinated at the glycerol backbone are described. In addition to being novel potential biomarkers for chlorine gas exposure, the compounds might have a negative impact on biological functions and produce a toxic response in organisms. Although we were able to verify their formation in lung tissue upon chlorination in vitro, further effort is needed to verify their suitability for analysis from samples obtained by convenient lung sampling methods, such as nasal lavage. The risk of false positives caused by metabolically produced HOCl must also be assessed.

## Supplementary Information

Below is the link to the electronic supplementary material.

ESM 1 (PDF 5.41 MB)

## Data Availability

The datasets generated and analyzed during the current study are available from the corresponding author upon reasonable request.

## Appendix

Chromatograms and interpretations of the fragmentation patterns are presented in the Electronic Supplementary Material. The Electronic Supplementary Material is available online.

## Abbreviations

(H)ESI: (Heated) electrospray ionization
(HR)MS: (High-resolution) mass spectrometry
ACN: Acetonitrile
BALF: Bronchoalveolar lavage fluid
CW: Chemical weapon
CWC: Chemical Weapons Convention
DAG: Diacylglycerol
DCM: Dichloromethane
FA: Formic acid
HCD: High-energy dissociation
HILIC: Hydrophilic interaction liquid chromatography
IPA: Isopropanol
LC: Liquid chromatography
MeOH: Methanol
MS/(HR)MS: (High-resolution) tandem mass spectrometry
NLF: Nasal lavage fluid
PC: Phosphatidylcholine
PE: Phosphatidylethanolamine
PLC: Phospholipase C
POPC: 1-Palmitoyl- 2-oleoyl-*sn*-glycero- 3-phosphocholine
POPE: 1-Palmitoyl- 2-oleoyl-*sn*-glycero- 3-phosphoethanolamine
PSPC: 1-Palmitoyl- 2-strearoyl-*sn*-glycero- 3-phosphocholine
TIC: Total ion chromatogram
UHPLC: Ultrahigh performance liquid chromatography
UPLC: Ultra-performance liquid chromatography
WWI: World War I
