# Supplementary material for: Novel chlorinated phospholipids—possible biomarkers of chlorine gas exposure
Source: Anal Bioanal Chem. 2025 Apr 11;417(15):3285–98. doi: 10.1007/s00216-025-05864-6 (PMC12122651; doi:10.1007/s00216-025-05864-6)
Supplement: Supplementary file 1 — (PDF 5.41 MB) [file 216_2025_5864_MOESM1_ESM.pdf]

Novel chlorinated phospholipids – possible biomarkers of chlorine gas exposure

Journal:	*Analytical and Bioanalytical Chemistry*
Manuscript ID	ABC-00281-2025.R1
Type of Paper:	Research Paper
Date Submitted by the Author:	21-Mar-2025
Complete List of Authors:	Rantanen, Noora-Kaisa; University of Helsinki, Finnish Institute for Verification of the Chemical Weapons Convention (VERIFIN)<br>Hamzah, Nurhazlina; Department of Chemistry Malaysia, Toxicology Division, Forensic Science Analysis Centre<br>Kjellberg, Matti; University of Helsinki, Finnish Institute for Verification of the Chemical Weapons Convention (VERIFIN)<br>Säde, Solja; University of Helsinki, Finnish Institute for Verification of the Chemical Weapons Convention (VERIFIN)<br>Vanninen, Paula; University of Helsinki, Department of Chemistry<br>Hakulinen, Hanna; University of Helsinki, Finnish Institute for Verification of the Chemical Weapons Convention (VERIFIN)
Keywords:	chlorinated phospholipids, peroxy-diphospholipids, chemical weapons, liquid chromatography, mass spectrometry, chlorine gas

SCHOLARONE™
Manuscripts

Novel chlorinated phospholipids – possible biomarkers of chlorine gas exposure

Noora-Kaisa Rantanen[a], Nurhazlina Hamzah[b], Matti A. Kjellberg[a,*], Solja Säde[a], Paula Vanninen[c] and Hanna Hakulinen[a]

a Finnish Institute for Verification of the Chemical Weapons Convention (VERIFIN), Department of Chemistry, University of Helsinki, P.O. Box 55, FI-00014 Helsinki, Finland.

b Toxicology Division, Forensic Science Analysis Centre, Department of Chemistry Malaysia, 46661 Petaling Jaya, Selangor, Malaysia

c Department of Chemistry, University of Helsinki, P.O. Box 55, FI-00014 Helsinki, Finland.

Corresponding Author: M. Kjellberg (matti.kjellberg@helsinki.fi), Finnish Institute for Verification of the Chemical Weapons Convention (VERIFIN), Department of Chemistry, University of Helsinki, P.O. Box 55, FI-00014 Helsinki, Finland

For Peer Review

ABSTRACT

Biomarkers are needed to verify suspected exposure to chlorine gas in chemical attacks. Here, we aimed to expand upon the array of known biomarkers of chlorine gas exposure. 1-Palmitoyl-2-oleoyl-sn-glycero-3-phosphocholine (POPC), a phospholipid belonging to the phosphatidylcholine (PC) class, was chosen as study material. The lipids were chlorinated using chlorine gas, followed by analysis for chlorinated PCs using liquid chromatography (LC) coupled with mass spectrometry (MS), using unit- (MS, MS/MS) and high-resolution (HRMS, MS/HRMS). By interpreting accurate masses, isotopic patterns, and fragmentation patterns, PC chlorohydrin, PC dichloride, the chlorinated PC with *m/z* 794.54611 (here denoted as PC A) and 10 novel chlorinated PCs were identified: four PCs chlorinated at the glycerol backbone and six chlorinated peroxy-diphospholipids. Similar experiments with 1-palmitoyl-2-oleoyl-sn-glycero-3-phosphoethanolamine (POPE) resulted in formation of the corresponding chlorohydrin and dichloride, as well as five chlorinated phosphatidylethanolamines (PEs) with chlorine in the glycerol backbone. Surprisingly, no chlorinated forms of 1-palmitoyl-2-stearoyl-sn-glycero-3-phosphocholine

(PSPC) were observed in similar experiments. In total, 15 novel chlorinated lipids were found. Furthermore, to determine the relevance of the novel lipids as biomarkers, a pig lung tissue sample was chlorinated *in vitro*. Two monomeric chlorinated PCs and two chlorinated peroxy-diphospholipids were identified from the chlorine-exposed lung sample, showing that the novel lipid compounds are potential biomarkers for chlorine gas exposure.

KEYWORDS

Chlorine gas, chlorinated phospholipids, peroxy-diphospholipids, chemical weapons, liquid chromatography, mass spectrometry

For Peer Review

1 INTRODUCTION

Chlorine (Cl_2) is a gaseous industrial chemical that is widely used in multiple areas of chemistry, such as in water treatment and in the manufacturing of polyvinyl chloride. In addition to its civil applications, chlorine also has military applications. Chlorine is a choking agent that has been used as a chemical weapon (CW) since World War I (WWI). Although the weaponized use of chlorine is prohibited by the General Purpose Criteria (Articla II.2) of the Chemical Weapons Convention (CWC) (1), it continues to be used as a CW, accounting for 89% of the chemicals used in the Syrian civil war between 2013 and 2018 (2).

Chlorine is extremely reactive and readily forms reaction products when it comes into contact with other substances. It is broken down by sunlight within several minutes, and when it dissolves in water, it converts to chloride (Cl^-), and hydrochloric (HCl) and hypochlorous (HOCl) acids (3). Victims can be exposed to chlorine by inhalation, as well as skin and eye contact during incidents, such as chlorine tank leaks, improper use of chlorine-containing household chemicals, and chlorine attacks (3).

Methods are needed to detect chlorine gas exposure in biomedical samples of exposed individuals. However, finding unambiguous biomarkers for chlorine gas exposure is difficult, due to the innate immune system producing reactive chlorine species in the body, e.g. as a result of infections or chronic illnesses (4–6). Mono- and dichlorotyrosine are two of the proposed markers for chlorine gas exposure, since both are stable and can be found in the tyrosine residues of proteins. Methods have been developed for detection

of chlorotyrosines in blood and plasma (7,8), hair (9,10), and tissue samples (10,11). Chlorotyrosines, chlorodopamines, 2-amino-6-chloropurine (DNA-adduct), and multiple other chlorine-containing biomolecules have also been detected in plants exposed to chlorination agents. Of these, chlorodopamines were identified as chlorine gas specific biomarkers (12). Recently, the effect of chlorine gas exposure on volatile organic compounds (VOCs) in exhaled air was studied using mice. The authors observed several chlorinated chemicals and other changes in the VOC composition of the exhaled air of the clorine exposed individuals (13). Though interesting, one should keep in mind that the results obtained with plants and mice may not be translatable for humans.

Notably, chlorine biomarkers can also be found in lipids. Thus far, the primary species of chlorinated lipids studied include chlorinated sterols and fatty acids, phospholipid chlorohydrins, α-chloro fatty aldehydes, and the oxidation products of chlorinated fatty acids and aldehydes (8,12,14–19). Chlorinated lipids have been detected in human (15,16) and animal (8,18,19) lung samples, plasma (8,18), and abscessed post-mortem human tissue (17). Just like other published biomarkers for chlorine gas exposure, chlorinated lipids and fatty acids are also produced by e.g. medical conditions and in exposure to chlorine containing household chemicals. Though, the timescale in which the chlorinated lipids become detectable may vary for the chlorination sources (16).

Since chlorine is absorbed into the body via inhalation, it is beneficial to collect samples from the upper and lower airways, particularly from the nose to the bronchial level (3). Two suitable biomedical specimens for this are bronchoalveolar lavage fluid (BALF) and nasal lavage fluid (NLF). Collection of BALF samples involves the insertion of a bronchoscope through the mouth or nose into the lungs and is rarely performed on healthy humans. Sampling of NFL is a more realistic option; it is obtained by squirting fluid into the patient's nostrils, and the collected fluid is used for analysis (20). Both BALF and NLF contain lipids making them suitable samples for the analysis of chlorinated lipid biomarkers of chlorine exposure. Phospholipid chlorohydrins such as the chlorohydrin of 1-palmitoyl-2-oleoyl-sn-glycero-3-phosphocholine (POPC) have been detected in BALF (15,19) and NLF (16). Chlorinated fatty acids such as α-chlorofatty acid are also found in BALF (15). Other chlorinated lipids, namely 2-chloropalmitaldehyde, 2-chloro-stearaldehyde, and their oxidized products (free and esterified 2-chloropalmitic acid, and 2-chlorostearic acid) have been detected in the lungs of chlorine-exposed mice and rats (18).

For Peer Review

Phospholipids are the most prevalent lipid component in the exoplasmic (inner) and cytosolic (outer) leaflets of the plasma membrane in eukaryotic cells (21). Typically, phosphatidylcholines (PCs) comprise 40–50% of the total phospholipids in the plasma membrane and are the only phospholipids present in both leaflets. In the cytosolic leaflet, the PCs are generally fully saturated, while the exoplasmic leaflet is dominated by polyunsaturated PC species (22). PCs are a major component of the pulmonary surfactant of the lung, which comprises of 70–80% phospholipids (23,24). The presence of double bonds in unsaturated fatty acyl chains has a significant effect on the membrane structure fluidity (21). PCs and phosphatidylethanolamines (PEs) are the most abundant phospholipids in human nasal mucosa (16). It has been proposed that the formation of chlorinated lipids occurs when HOCl reacts with lipids via electrophilic attack on the double bonds in the unsaturated fatty acyl chains and via N-halogenation of the lipid headgroups including PC and PE, resulting in chlorohydrins and peroxidation products (14).

Here, POPC, a ubiquitous phospholipid belonging to the PC class, was chosen as the main target molecule for study. In addition, the corresponding saturated PC (1-palmitoyl-2-stearoyl-sn-glycero-3-phosphocholine, PSPC) and the monounsaturated PE (1-palmitoyl-2-oleoyl-sn-glycero-3-phosphoethanolamine, POPE), were studied. POPC, PSPC and POPE are commercially available and naturally found in eukaryotic cell membranes. The general structure of PC and PE lipids and the structures of the lipids of interest in this work are presented in **Fig. 1**. The aim of this work was to identify novel chlorinated phospholipids, using liquid chromatography (LC) coupled to mass spectrometry (MS), employing unit- (MS, MS/MS) and high-resolution (HRMS, MS/HRMS) tandem MS. The experiments were carried out using single-lipid model systems and pig lung samples.

For Peer Review

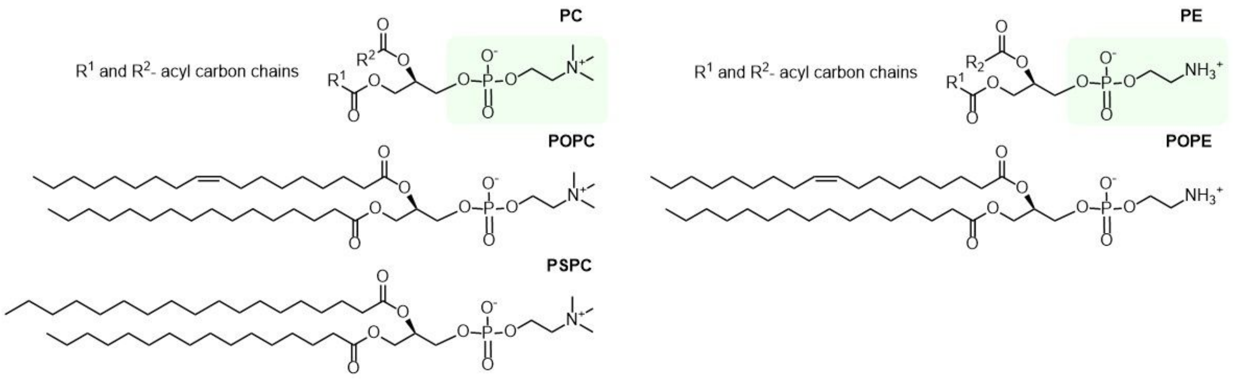

Fig. 1 Structures of lipid classes PC and PE, and the lipids POPC, PSPC, and POPE. The headgroups of PC and PE lipids are marked with a green box

2 EXPERIMENTAL SECTION

All described experiments were repeated at least once, with comparable or practically identical results.

2.1 Materials and reagents

POPC (> 99%), PSPC (> 99%) and POPE (> 99%) were obtained from Avanti Polar Lipids (Alabaster, AL, USA), chlorine gas (500 parts per million (ppm)) from Messer Austria GMH (Gumpoldskirchen, Austria), formic acid (FA) for LC-MS (98–100%) and Phospholipase C (PLC) from *Clostridium perfringens* from Merck (Burlington, MA, USA). Acetonitrile (ACN), dichloromethane (DCM), isopropanol (IPA) and methanol (MeOH) were obtained from Honeywell Fluka (Loughborough, Leicestershire, UK). Sodium hydroxide (NaOH) and chloroform were obtained from Thermo Fisher Scientific (Loughborough, Leicestershire, UK). Ammonium formate (NH_4HCO_2) was purchased from Sigma Aldrich (St. Louis, MO, USA). Concentrated HCl, sodium chloride (NaCl), calcium chloride ($CaCl_2$), Tris-base and bovine serum albumin were purchased from VWR International (Radnor, PA, USA). Deuterated MeOH (d_4-MeOH) and chloroform (d_1-chloroform) were obtained from Eurisotop (Saint-Aubin, France). All chemicals and reagents were of analytical grades or higher. The water used in this study was purified using a Direct-Q® 3 UV system from Millipore (Darmstadt, Germany). A lung from a freshly slaughtered pig was obtained from a local butcher (Tilateurastamo Kiven Säästöpossu, Karkkila, Finland), cut into smaller pieces, and stored at -80 °C until analysis.

2.2 Instrumentation

The LC-MS and LC-MS/MS analyses were performed with an Acquity I-Class UPLC® (Waters™; Milford, MA, USA) coupled to a Xevo® TQ-XS triple quadrupole mass spectrometer (Waters™). LC-HRMS and LC-MS/HRMS analyses were performed with a Thermo Scientific Dionex Ultimate 3000 UHPLC (Germering, Germany), coupled to a Thermo Scientific Orbitrap Fusion™ mass spectrometer (San Jose, CA, USA). LC separation was achieved using ACQUITY UPLC® BEH HILIC (2.1 x 100 mm, 1.7 µm; Waters™) and XBridge® BEH C18 (2.1 x 100 mm, 2.5 µm; Waters™) columns. For hydrophilic interaction liquid chromatography (HILIC) analysis, we used the following eluents: ACN (A), and 55 mM NH_4HCO_2 in 50% ACN in ultrapure water (B). The gradient was run from the starting point of 2% B for 1 min to 20% B at 10 min. The B eluent was linearly increased to 40% over 2 min and held for 3 min. The B ratio was

For Peer Review

linearly reduced to 2% B within 1 min, and the column was equilibrated for 4 min at 2% B, for a total run time of 20 min. The column oven was set to 45 °C. The flow rate was 0.5 mL/min and the injection volume was 5 µL. For analysis with the reverse-phase C18 column, these LC eluents were used: 0.1% FA and 5 mM of NH_4HCO_2 in 60% ACN (A); 0.1% FA and 5 mM of NH_4HCO_2 in 80% IPA and 20% ACN (B). The column oven was set to 60 °C. The chromatographic gradients consisted of initial conditions of 5% B at 0.6 min before linearly increasing to 100% B over 13 min. It was then held for 4 min before the B ratio was reduced to 5% B within 1 min, and the column was equilibrated for 2 min at 5% B. The flow rate was 0.6 mL/min, and the injection volume was 5 µL.

For the MS and MS/MS analyses, electrospray ionization (ESI) in positive mode was used. The MS parameters were set as follows: capillary voltage 3 kV, cone voltage 20 V, source temperature 150 °C, desolvation temperature 500 °C, cone gas flow 150 L/h, desolvation gas (nitrogen, N_2) flow 1000 L/h, collision gas (argon, Ar) flow 0.15 mL/min. MassLynx (Waters™, version 4.2) was used for data acquisition and analysis. For the precursor ion-scanning analyses (parent ion scanning of the mass-to-charge ratio (*m*/*z*) 184), a collision energy of 33 V was used. The collision gas was Ar. For the HRMS and MS/HRMS analyses, ionization was performed using heated electrospray ionization (HESI) in the positive ion mode, with N_2 as the spray gas and helium (He) as the collision gas. The instrument parameters were set as follows: spray voltage 4500 V, source temperature 350 °C, ion transfer tube temperature 230 °C, sheath gas 40 arbitrary units (Arb), auxiliary gas 15 Arb, and sweep gas 0 Arb. Exact mass measurements were carried out in the *m*/*z* range of 600–1000 with RF lens at 60% and a resolution of 60,000 in the full scan method and 120,000 in the product ion scan method. The instrument was calibrated to ≤ 5 ppm mass accuracy using external calibration. Stepped high-energy collisional dissociation (HCD) with 15%, 30% and 45% energies was used to fragment the ions. Xcalibur™ (Thermo Scientific, version 4.5) was applied for data acquisition and analysis.

For Peer Review

2.3 Chlorination of phospholipids

POPC (7.6 mg) in chloroform was transferred to a glass tube with a screw cap (Kimax®; Merck) and concentrated to dryness under a stream of N_2 in a TurboVap® LV II (Caliper Life Sciences, Hopkinton, MA, USA), at 7.5 psi and 30 ^{0}C. Next, 1 mL of water was added to the dried POPC, resulting in a 10 mM

sample. The aqueous 10 mM sample was bath-sonicated (Branson 3200; Branson Ultrasonics; Brookfield, CT, USA) for 1 h at room temperature to form a POPC vesicle solution. The vesicle solution was exposed to chlorine gas, with a 500 ppm concentration, for 5 min by submerging the gas stream (approximately 1 L/min) in the liquid to produce bubbling. The chlorinated POPC was extracted using a modified Folch's extraction method: 1.5 mL of MeOH and 3 mL of DCM were added, and the sample was vortexed for 10 seconds. The tube was then shaken for 1 min before being centrifuged for 5 min at 1000 revolutions per minute (rpm). The DCM (lower) layer was collected and dried in a TurboVap® LV II (as above). The dry extract was reconstituted with 1 mL of ACN. An aliquot of the reconstituted sample was further diluted with ACN to a concentration equivalent of 50 µM POPC starting material for analysis by LC-MS-based techniques. Unit-resolution LC-MS was used to screen for novel chlorinated PCs. LC-HRMS and LC-MS/HRMS were used to measure the accurate masses and fragmentation patterns of the novel compounds. For comparison, a chlorination of 10 mM POPC in chloroform was similarly performed. After the chlorination, the chloroform was evaporated (TurboVap® LV II, as above), and the sample was subsequently reconstituted in 1 mL of ACN. An aliquot of the sample was diluted and analyzed as described above. The chlorination experiments were repeated using PSPC (7.6 mg) and POPE (7.2 mg) dissolved in chloroform and in water. All prepared samples were stored at -20 ^{0}C until analysis. Un-treated control samples were prepared similarly.

2.4 Pig lung tissue chlorination

A surface piece of approximately 0.5 g was cut from pig lung, which had previously been frozen and stored at -80 ^{0}C. The thawed lung sample was placed in a 10 mL borosilicate glass tube with a screw cap (Kimax®). The lung sample was exposed to 500 ppm chlorine gas flow for 5 minutes, shaken and then exposed for an additional 5 min. The chlorine gas flow in the exposure was approximately 1 L/min. The surface lipids of the exposed lung tissue sample were collected by submerging the sample in 1 mL of water, followed by a modified Folch's extraction protocol (see section 2.3). The dried extract was reconstituted in 400 µL of ACN for analysis by LC-HRMS and LC-MS/HRMS. An un-exposed control lung sample was identically prepared.

For Peer Review

2.5 Mild alkaline hydrolysis

A 0.1 mL sample of a previously chlorinated 10 mM POPC solution in ACN (chlorinated in water, see section 2.3) was evaporated to dryness in a glass tube (Kimax®) under N_2 flow (TurboVap® LV II, see section 2.3). 0.5 mL MeOH and 0.5 mL chloroform were added to the evaporation residue before the tube was shaken. 167 µL of 0.3 M NaOH in MeOH/water (freshly prepared as 96:4 by volume) was added before vortexing thoroughly and incubating in the dark for 4 h. The mixture was neutralized by adding 100 µL of 0.3 M HCl in MeOH (prepared from concentrated HCl) before vortexing. Following this, 1.5 mL chloroform, 0.16 mL MeOH and 0.75 mL of water were added to the mixture before vortexing and centrifuging at 2000 rpm for 10 min. The upper phase was collected and analyzed directly by LC-MS. The lower phase was washed once with 2 mL of Folch's theoretical upper phase containing chloroform, MeOH and water (3:48:47 by volume). The mixture was shaken and centrifuged again before the upper phase was discarded. The lower phase was dried in a TurboVap® LV II (see section 2.3) and reconstituted with 1 mL of ACN. An aliquot of the reconstituted sample was further diluted with ACN to a concentration equivalent of 50 µM POPC starting material, and analyzed by LC-MS.

2.6 Enzymatic cleavage by phospholipase C

For PLC treatment, 0.1 mL of a previously chlorinated 10 mM POPC solution in ACN (chlorinated in water, see section 2.3) was evaporated to dryness in a glass tube (Kimax®) under N_2 flow (TurboVap® LV II, see section 2.3), and 0.5 mL of PLC buffer (140 mM NaCl, 10 mM Tris-HCl, 10 mM $CaCl_2$, 0.1% bovine serum albumin, pH 7.2) was added to form an approximate concentration of 2 mM chlorinated POPC solution. The lipids were emulsified by vortexing and PLC (solubilized in PLC buffer) was added to an approximate concentration of 5 units/mL. The sample was incubated overnight while shaking at 37 ^{0}C. A concentration matched, PLC-untreated sample was used as a control. The samples were extracted using a modified Folch's extraction protocol (see section 2.3). The dry extracts were reconstituted in 1 mL of ACN. An aliquot was further diluted with ACN to a concentration equivalent of 100 µM POPC starting material, prior to analysis by C18 LC-MS and LC-MS/MS.

For Peer Review

3 RESULTS AND DISCUSSION

3.1 POPC vesicles chlorinated in water

The POPC samples chlorinated as vesicles in water were first analyzed with LC-MS and LC-HRMS using a C18 column in positive polarity, before further analysis with HILIC separation. Chlorinated PCs were not detected in the un-treated control sample. Five chlorinated PCs were found in the chlorine-exposed sample: PC chlorohydrin (PC HOCl), PC dichloride (PC D), and analytes with theoretical accurate *m*/*z* values of 794.54611 (PC A), 777.56560 (PC B) and 803.55139 (PC C) (**Table 1**). Evaluation of the isotopic patterns of PC A–C revealed that PC A and B contain one chlorine atom and PC C two chlorine atoms (**Fig. S10–S12**). The ^{13}C peaks of PC B and C differed by approximately 0.5 mass units from the monoisotopic ion peak (**Fig. 2**, and **Fig. S11** and **Fig. S12** in the Electronic Supplementary Material (SM)), indicating that the analytes are doubly charged.

A chlorinated PC with an accurate mass of 794.54611 has previously been reported in POPC samples incubated with HOCl (17). Based on the fragmentation pattern, the authors suggested that the analyte was a POPC chlorinated in one of the acyl chains without opening the double bond. Here, LC-MS/HRMS with stepped HCD fragmentation was used to fragment PC A–C to obtain structural information. All three lipids produced typical phosphocholine fragments (*m*/*z* 86.09643, 98.98417, 104.10699, 124.99982, and 184.07332, proposed structures in **Fig. 3**) (25), indicating that the chlorine substituent is not located in the headgroup. This is also supported by the chlorinated fragments consisting of the glycerol backbone and one or two acyl chains (e.g. *m*/*z* 977.61184, 794.54611, 611.48006, 556.31644, and 373.25040) (**Tables S3**, **S5**, and **S6**, **Fig. S21–S23** in the SM). Interestingly, PC A and PC C produced a fragment ion with an *m*/*z* of 202.03943 (**Fig. 3**), corresponding to the phosphocholine headgroup in which one of the hydroxyls in the phosphate has been replaced with a chlorine atom (**Tables S3** and **S6**, **Fig. S31** and **S33** in the SM). This fragment is presumably produced by re-arrangement reactions during the collisional fragmentation. For the re-arrangement reaction to occur, the chlorine atom must be in close proximity to the phosphate, i.e. either in the headgroup or in the glycerol backbone. Since PC A and C produce the aforementioned phosphocholine fragments, it is unlikely that the chlorine substituent is in the headgroup. PC A also produced fragment ions with *m*/*z* 239.23694, 263.23696 and 199.03678. The first two mentioned are ions

For Peer Review

produced in the fragmentation of the un-chlorinated acyl chains. The last ion mentioned could potentially have the molecular formula $C_6H_{12}ClO_5^+$, which corresponds the chlorinated glycerol backbone (**Fig. 3**).

Based on the aforementioned and other fragments of PC A–C (and the other detected chlorinated PCs discussed in section 3.2), we propose that the chlorine substituent is located in the glycerol backbone, which differs from previously made conclusions on the structure of the chlorinated PC with the *m*/*z* value of 794.54611 (17). This hypothesis is supported by previous studies where chlorination of ethers resulted in addition of chlorine in the α- and β-positions. While the α-carbon of the carbonyl can also be chlorinated (26), the glycerol backbone might be a more available reaction site, hence leading to the observed products. Selected fragments of the chlorinated PCs are presented in **Fig. 3**. The exact position of the chlorine atom(s) in the glycerol backbone cannot be concluded from the data available. However, multiple peaks were detected for many of the analytes, suggesting that multiple structural isomers of the chlorinated PCs were formed during the chlorination.

Based on the two charges and the fragmentation (e.g. *m*/*z* 489.30972 = PC A with an additional phosphocholine headgroup), we propose that PC B and C are chlorinated, dimeric peroxy-diphospholipids in which one of the lipid monomers is PC A. The second lipid in the dimers is POPC or PC HOCl in PC B and PC C, respectively.

The structures of PC HOCl and PC A–D (and PC E–L, which are discussed in section 3.2) are presented in **Fig. 4**. The isotopic patterns of the analytes are presented in **Fig. S9–S13**, and the fragmentation patterns of the analytes can be found in **Tables S1**, **S3**, **S5**, **S6** and **S8** in the SM. The proposed structures for the detected fragments are presented in **Fig. S30–S34** in the SM.

For Peer Review

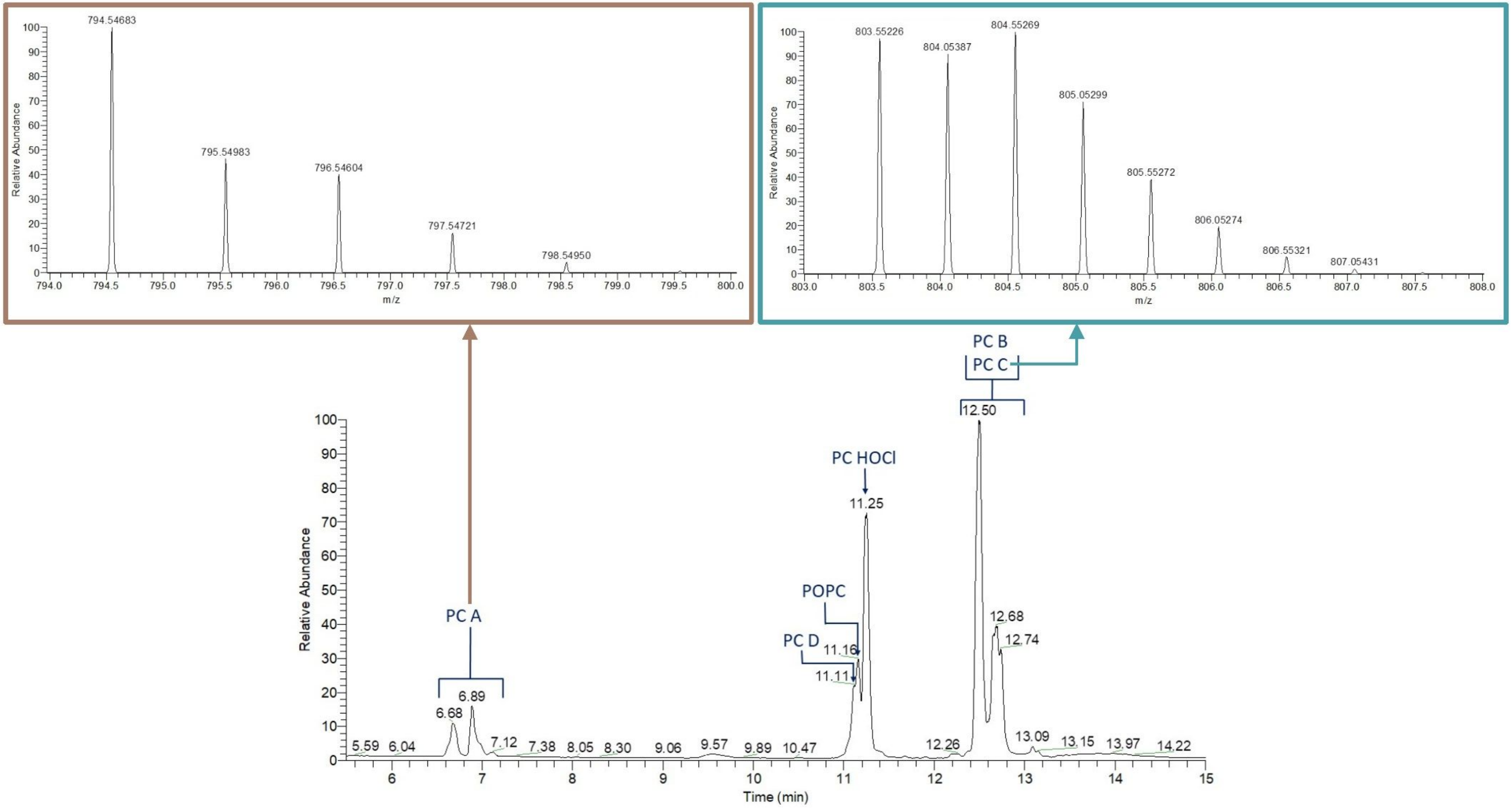

Fig. 2 TIC of a POPC sample chlorinated in water separated by HILIC and the isotopic patterns of PC A (left) and PC C (right). The chlorinated PCs elute in three structure-dependent groups: PCs chlorinated in the glycerol backbone (e.g. PC A), PCs chlorinated in the acyl chains (e.g. PC D), and dimeric chlorinated PCs (e.g. PC C). The isotope peaks in the isotopic pattern of PC C are 0.5 units apart, which is typical for doubly-charged ions

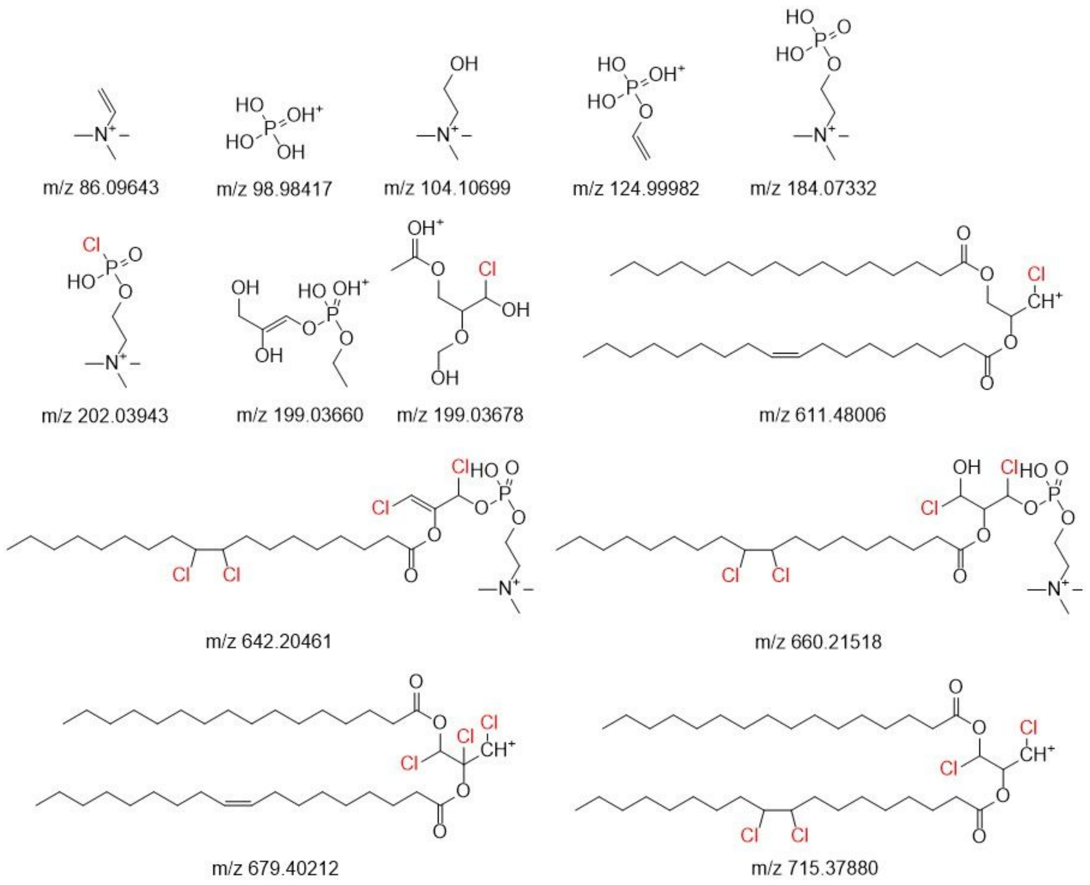

Fig. 3 Proposed structures of selected fragments of the chlorinated PCs

Review

Table 1 Abbreviations of the chlorinated lipids, their predicted formula and theoretical and measured *m*/*z*. Masses corresponding to the lipids marked with an asterisk (*) were detected in the chlorinated lung sample

Lipid	Predicted formula	z	Theoretical *m/z*	Measured *m/z*	Error (ppm)
			**Chlorinated PCs**		
PC HOCl*	$C_{42}H_{84}ClNO_9P^+$	1	812.55667	812.55767	1.22240
PC A*	$C_{42}H_{82}ClNO_8P^+$	1	794.54611	794.54683	0.90686
PC B	$C_{84}H_{165}ClN_2O_{16}P_2^{2+}$	2	777.56560	777.56672	1.44039
PC C*	$C_{84}H_{166}Cl_2N_2O_{17}P_2^{2+}$	2	803.55139	803.55226	1.08539
PC D*	$C_{42}H_{83}Cl_2NO_8P^+$	1	830.52279	830.52391	1.34962
PC E	$C_{42}H_{82}Cl_3NO_8P^+$	1	864.48381	864.48357	-0.27991
PC F*	$C_{84}H_{165}Cl_3N_2O_{16}P_2^{2+}$	2	812.53445	812.53392	1.81570
PC G	$C_{84}H_{164}Cl_4N_2O_{16}P_2^{2+}$	2	829.51496	829.51399	-1.17469
PC H	$C_{84}H_{163}Cl_5N_2O_{16}P_2^{2+}$	2	846.49548	846.49431	-1.37940
PC I	$C_{84}H_{162}Cl_6N_2O_{16}P_2^{2+}$	2	863.47599	863.47391	-2.40630

**Lipid**	**Predicted formula**	**z**	**Theoretical *m/z***	**Measured *m/z***	**Error (ppm)**
PC J*	$C_{42}H_{81}Cl_4NO_8P^+$	1	898.44484	898.44468	-0.18223
PC K	$C_{42}H_{81}Cl_2NO_8P^+$	1	828.50714	828.50808	1.13393
PC L	$C_{42}H_{80}Cl_3NO_8P^+$	1	862.46816	862.46855	0.44985
			**Chlorinated PEs**		
PE HOCl	$C_{39}H_{78}ClNO_9P^+$	1	770.50972	770.51004	0.41349
PE A	$C_{39}H_{77}Cl_2NO_8P^+$	1	788.47584	788.47738	1.95313
PE B	$C_{39}H_{77}Cl_2NO_9P^+$	1	804.47075	804.47082	0.08136
PE C	$C_{39}H_{76}Cl_3NO_8P^+$	1	822.43686	822.43597	-1.08806
PE D	$C_{39}H_{76}ClNO_8P^+$	1	752.49916	752.49996	1.06970
PE E	$C_{39}H_{75}Cl_2NO_8P^+$	1	786.46019	786.46131	1.42531
PE F	$C_{39}H_{74}Cl_3NO_8P^+$	1	820.42121	820.42299	2.16989

For Peer Review

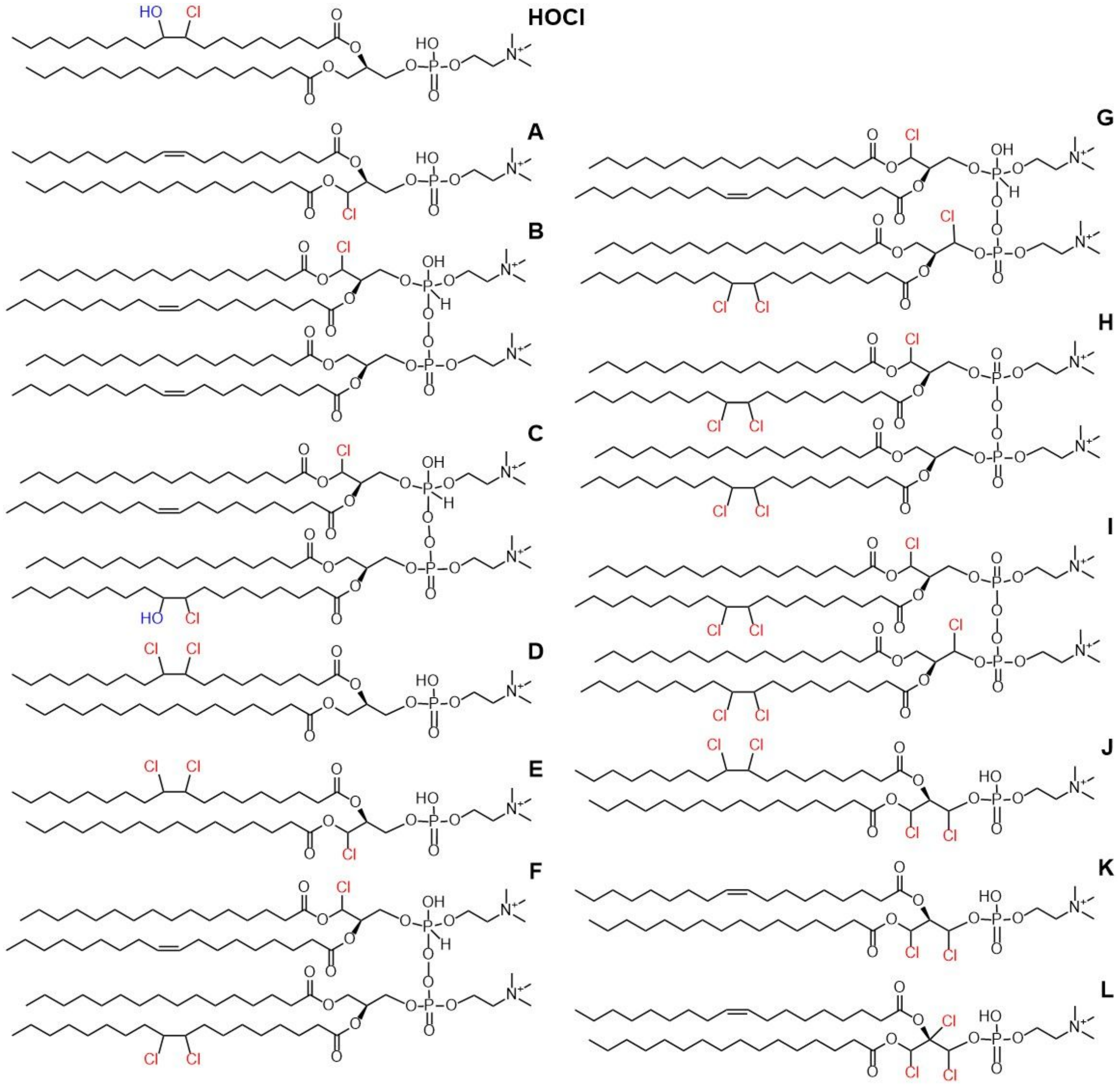

Fig. 4 Proposed structures of PC HOCl and PC A–L, as detected by LC-HRMS. The chlorine substituent(s) may be located in any of the carbons in the glycerol backbone

To obtain further structural information, the samples were also analyzed with HILIC LC-HRMS. In C18, retention is mainly based on hydrophobicity; hydrophobic analytes are retained more strongly than hydrophilic analytes. The hydrophobicity of PCs is determined by the lengths and degrees of unsaturation of the alkyl chains (27). The retention of analytes in HILIC columns is more complicated, but generally polar and ionic compounds are retained more effectively (28). The polarity of PCs is mainly determined by the phosphocholine headgroup (27). PC B and C show the strongest retention on HILIC, presumably because they both have two charged cholines which like to interact with the hydrated zone and charges of the stationary phase. The retention times suggests that PC A is the least polar/ionic of the four chlorinated

PCs. An electronegative chlorine atom in the glycerol backbone might decrease the net-polarity of PC A, e.g. by attracting the quaternary amine or by changing the charge distribution in the headgroup. This can, in turn, result in changes in the PCs' interactions with solvent molecules and the stationary phase and shifting of the retention times. Further research is needed to determine how the chlorine modification in the glycerol backbone affects the structure and properties of PCs. Nevertheless, the charge and chlorine substituent position-dependent retention in HILIC separation were useful in determining the structure of the chlorinated PCs and PEs discussed in sections 3.2 and 3.5.

Experiments using ESI negative polarity scanning were also performed with C18 LC-MS. Of the chlorinated analytes, only PC HOCl and PC D were detected as negative ions ($[M-CH_3]^-$, m/z 796.5 and m/z 814.5 respectively, data not shown). In the case of the glycerol-chlorinated lipids, the inability to produce negatively charged ions may have been due to changes in ionizability, caused by the very electronegative chlorine atom near the headgroup. Similarly to un-chlorinated PCs, PC HOCl and PC D (which contain chlorine atoms only in their acyl chains, see **Fig. 4**) were negatively ionized, through a loss of a methyl group and subsequent neutralization of the quaternary ammonium choline (25).

For further structural investigation of the position of the chlorine substituents and the dimeric lipids, a sample was prepared for ^1H nuclear magnetic resonance (NMR) analysis. A 1 mL sample containing 10 mM of POPC in water was chlorinated, after which the solvent was changed to a mixture of d_4-MeOH and d_1-chloroform (1:5, 600 µL) and analyzed by ^1H NMR. An untreated control sample of POPC was prepared and analyzed similarly. The results of the analysis were, however, inconclusive due to the complexity of the chlorinated sample. For easier interpretation, the various chlorinated molecular species would need to be isolated, which is planned for future experiments.

3.2 POPC chlorinated in chloroform

Samples prepared by chlorination of POPC dissolved in chloroform and untreated control samples were also analyzed with LC-HRMS. The samples were prepared in an attempt to determine whether an aqueous environment is necessary for the production of the novel lipids, as well as to study whether a water-free environment produces additional chlorinated PCs that could give more structural insight into the location of the chlorine substituents in the novel lipids.

For Peer Review

No chlorinated lipids were detected in the control sample. Most of the chlorinated lipids observed in the samples chlorinated in water were detected in the chloroform samples as well, however, eight additional chlorinated PCs were also identified (**Table 1**, **Fig. 4**). The isotopic patterns and fragmentation measured by LC-HRMS and LC-MS/HRMS indicated that four of the eight additional compounds were peroxy-diphospholipids (PC F, G, H and I) (**Fig. S15–S18** in the SM). The other four had chlorine atoms attached to their glycerol backbone (PC K and L), or to both the glycerol backbone and the acyl chain (PC E and J). They produce fragments containing the glycerol backbone with one or both acyl chains, which support the proposed chlorination site being located in the glycerol backbone (selected structures in **Fig. 3**). For example PC E and J produce fragments corresponding to ions resulting from the loss of the saturated acyl chain and the loss of the phosphocholine (*m*/*z* 681.41777, 626.25415, 715.37880 and 660.21518) (selected structures in **Fig. 3**). These fragments show that the chlorine atoms must reside in the oleoyl-chain and/or the glycerol backbone. Since the double bonds in both molecules have been saturated with chlorine, the only logical location for the remaining chlorine atoms is the glycerol backbone. The corresponding fragments (*m*/*z* 645.44109, 590.27747, 643.42544 and 624.23850) and the retention time of PC K and L support the conclusion. The fragmentation patterns and isotopic patterns of the analytes can be found in **Tables S10**, **S11**, **S13–S16**, **S18** and **S19** and **Fig. S14**, **S19–21**, and **S35–S42** in the SM. The proposed structures for all chlorinated PCs are presented in **Fig. 4**, and their masses are listed in **Table 1**. The measured accurate masses of all chlorinated PCs did not differ more than ±2.5 ppm from the theoretical value.

Chlorination of POPC dissolved in chloroform resulted in the production of individual lipids with higher degrees of chlorination than obtained in the chlorination of an aqueous POPC-sample. In water, the differences in hydrophobicity of the headgroup and the alkyl chains cause the lipids to arrange in bilayer vesicles, especially when energy is introduced into the system e.g. through sonication. Although chlorine is a small molecule, the ordered structure of the bilayer vesicles may limit access to the unsaturated sites, and electrostatic and other physical interactions with headgroups nearby may restrict reactivity at the glycerol backbone. In addition, the reaction of chlorine with water (producing HOCl and HCl (29)) competes with the chlorination of the lipids. In chloroform, the lipids are ordered as inverted micelles, in which the acyl chains are oriented towards the solvent. There are fewer competing reactions with the

For Peer Review

solvent, resulting in more polychlorinated mono- and dimeric-PCs. Due to the differing behavior of lipids and reactions of chlorine in chloroform and in aqueous systems, the chloroform experiments are poor simulations of chlorination reactions in biological systems. Therefore, the suitability of the polychlorinated PCs identified in the chloroform experiments as biomarkers should be critically evaluated. Some polychlorinated PCs may also be formed in tissues (aqueous systems) from chlorine produced in the spontaneous degradation of externally introduced or metabolically produced HOCl. Dichlorination of unsaturated sites through natural metabolism has been demonstrated by the detection of dichlorinated cholesterol in tissue (4,14). However, when chlorine is used as a CW, the concentrations are very high. Therefore, it is feasible that polychlorinated lipids, such as the PCs identified in the chloroform experiments, would be more prominent in biomedical samples of chlorine attack victims, than in samples from patients suffering from immunological stress conditions.

For Peer Review

Chromatograms of the POPC sample chlorinated in chloroform and analyzed with the different separation methods (C18 and HILIC) are presented in **Fig. S**1 and **Fig. S2** in the SM. From the chromatograms, one can note that the chlorinated PCs elute in three groups on both stationary phases. The first eluting group consists of the PCs chlorinated only at the glycerol backbone (PC A, K and L). The POPCs chlorinated at the acyl chain double bond (PC D, E and J) are eluted next. With the HILIC column, PC HOCl elutes in the second group. With the C18 column, the increased interactions with the mobile phase cause the PC HOCl to elute before the first group of chlorinated lipids. The peroxy-diphosphocholines (PC C, F, G, H, and I) are eluted last on both stationary phases. This elution behavior taking place in groups is useful when determining the possible location of the chlorine atom(s) in the lipids. Multiple chromatographic peaks can be detected for most of the chlorinated PCs. This may have resulted from the analytes having several isomers. No conclusions from the structural differences could be drawn from the mass-spectrometric fragmentation patterns of the isomers.

3.3 Mechanism of dimerization

As can be seen from the structures presented in **Fig. 4**, all chlorinated peroxy-diphosphocholines (PC B, C, F, G, H and I) have at least one chlorine substituent in the glycerol backbone. Our hypothesis is that the dimerization is initiated by the formation of a PC chlorinated at the glycerol, e.g. PC A. The chlorine

substituent changes the three dimensional structure and electron distribution in the headgroup, allowing dimerization with other lipids in its proximity. After dimerization, the lipids can be further chlorinated at the glycerol and remaining unsaturated sites in the acyl chains. The literature describes the production of peroxy-diphosphates by electrolysis of phosphate solutions containing added halide or pseudohalide anions (30). However, the exact mechanism of the chlorination and subsequent dimerization of phospholipids cannot be determined from our data. Further experiments are needed to elucidate the mechanisms involved.

3.4 Alkaline and enzymatic degradation of chlorinated POPC

In an attempt to verify the position of the chlorine substituent in PC A, chlorinated aqueous POPC samples were treated with mild alkaline hydrolysis or with PLC and analyzed with positive full-scan LC-MS and positive precursor ion scanning of *m*/*z* 184 (LC-MS/MS). Positive precursor ion scanning of *m*/*z* 184 is a PC-specific MS/MS-scanning mode (31). The rationale was to investigate whether alkaline or enzymatic degradation would result in degradation products, that would confirm the position of the chlorine in PC A (e.g. a chlorinated glycerophosphocholine or a chlorinated diacylglycerol (DAG)). Mild alkaline hydrolysis degrades PCs to fatty acids and a glycerophosphocholine (32). The free fatty acids were detected in alkaline treated chlorinated POPC samples, however, the chlorinated glycerophoshocholine was not detected (data not shown). No chlorinated species were found, which might be a result of the alkaline conditions causing elimination of the chlorine substituent.

PLC is an enzyme that is capable of releasing the phosphocholine headgroup of PCs, yielding a corresponding diacylglycerol lipid (33,34). PLC-treatment was able to liberate phosphocholine from POPC, PC HOCl and PC D, i.e. monomeric PCs without a chlorine substituent in the glycerol backbone (**Fig. 1** and **Fig. 4**). The liberation was evidenced by the loss of respective PC signals (**Fig. S50** in the SM) and the appearance of the corresponding DAG in the organic phase (**Fig. S52** in the SM). Interestingly, PC A, which putatively contains a chlorine substituent in the glycerol backbone, was seemingly resistant to PLC degradation, as evidenced by apparently no loss of signal intensity between PLC-treated and -untreated samples (**Fig. S50** and **Fig. S51** in the SM). Masses corresponding to a DAG released from PC A could not be observed in any of the PLC-treated samples, however, the signal may have been below the limit of detection of the instrument.

For Peer Review

The signals of PC B and PC C also disappeared after PLC-treatment (**Fig. S53** in SM), but no logical degradation products could be identified for these compounds. It is unlikely that the PLC-enzyme was able to cleave the dimers (PC B and C) back into individual lipids, because the signal for PC A in the sample did not increase compared to that of an untreated sample (**Fig. S51** in the SM). Nevertheless, the resistance of PC A to degradation by PLC further suggests that the chlorine is located in a position close to the lipid headgroup, possibly hindering PLC from accommodating the lipid headgroup at its active site. The resistance to degradation could also have biological implications. Hindering cellular PC catabolism could affect the generation of lipid signaling molecules (e.g. phosphatidic acid and DAG), that are involved in a range of cellular processes (35).

3.5 Chlorinated PSPC and POPE

To determine whether other phospholipids in addition to POPC react similarly to chlorination, PSPC and POPE were evaluated. They were dissolved in chloroform or sonicated in water, and subsequently chlorinated and analyzed with LC-HRMS and LC-MS/HRMS, using HILIC separation. Chlorinated lipids were not detected in the control samples that had not been exposed to chlorine gas. Interestingly, no chlorinated PSPC-related lipids were found in any of the samples. The saturated acyl chains of PSPC may have formed more rigid membrane structures, sterically obstructing chlorine from reaching the glycerol backbone. The presence of unsaturated acyl chains (such as the oleoyl in POPC) is seemingly required for sufficient micellar and vesicle bilayer fluidity, and subsequent chlorination of the glycerol backbone. Further experiments are needed to verify this assumption.

Seven chlorinated PEs were detected in the chlorine-exposed POPE samples (**Table 1**, **Fig. 5**). The isotopic patterns and fragmentation patterns of the chlorinated PEs are presented in **Fig. S23–S29** and **S43–S48** and **Tables S20–S28** in the SM. The seven chlorinated PEs included the chlorohydrin (PE HOCl), the chlorine saturated PE (PE A) and five PEs chlorinated at the glycerol backbone (PE B–F). The proposed structures of PE A–F are presented in **Fig. 5**. All seven chlorinated PEs were detected in the POPE sample chlorinated in chloroform. PE F was not produced, and only small amounts of PE D was formed when POPE was chlorinated in water. This difference is presumably caused by the solvent effects described in section 3.2. The accurate masses of some dimeric chlorinated PEs were detected, but their low quantity did not allow

For Peer Review

reliable identification using isotopic patterns and LC-MS/HRMS analysis. The experiment with POPE showed that other unsaturated phospholipid classes can also be chlorinated in the glycerol backbone, but the reactivity of different lipid classes varies.

The chromatograms of the chlorinated POPE samples are presented in **Fig. S5–S7** in the SM. The retention of the chlorinated PEs follow the trends observed for the chlorinated PCs (section 3.1). However, there is a notable difference in the retention of the backbone-chlorinated PEs (PE D–F) and the PCs (PC A, K and L) using HILIC separation (**Fig. S2**, **S4**, **S6** and **S7** in the SM). The backbone-chlorinated PCs eluted near 7 min, while their PE analogues eluted at 1.6 min (PE D) and 0.6 min (PE E and F). This demonstrates that the addition of chlorine atoms to the backbone of POPE reduces the polarity of the resulting PE more compared to the corresponding PCs. The methyl substituents on the amine makes the headgroup of PCs bulkier, which may hinder their interaction with the chlorine substituents in the glycerol backbone and the 3D-structure of the chlorinated PCs. The much smaller unsubstituted amine in PEs may allow the headgroup to interact more efficiently with the chlorine atoms in the glycerol backbone, making the molecule less polar (almost non-polar in the case of PE E and F).

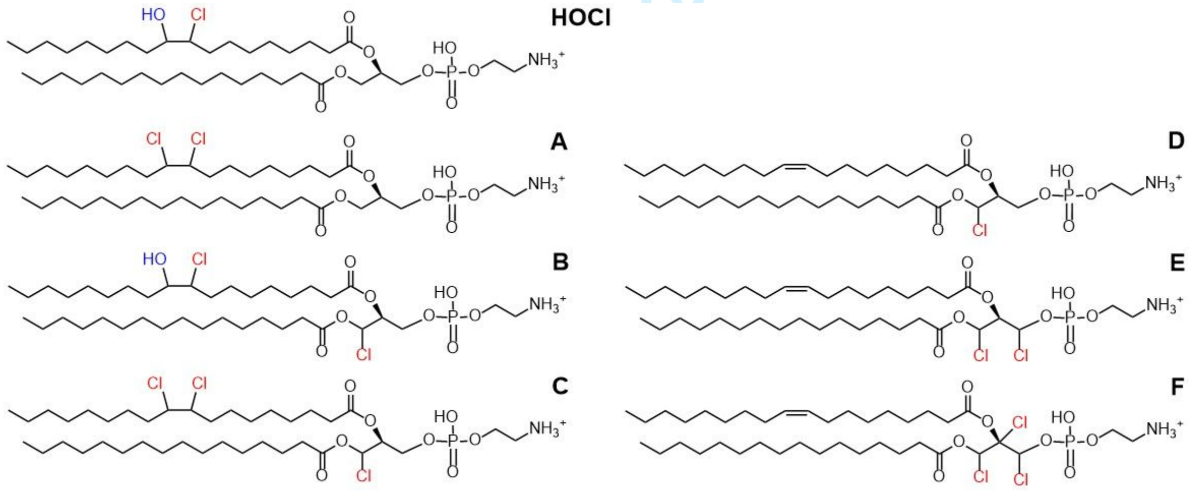

Fig. 5 Proposed structures of PE HOCl and PE A–F, as detected by LC-HRMS. The chlorine(s) may be located in any of the carbons in the glycerol backbone

3.6 Chlorinated pig lung sample

A sample of pig lung was chlorinated *in vitro* to determine whether the novel chlorinated lipids could be used as biomarkers for chlorine gas exposure. Lung tissue was selected as a chlorination target because inhalation is one of the main exposure routes for chlorine gas. The extracts of the chlorinated and untreated pig lung samples were analyzed using LC-HRMS for chlorinated PCs. Since both POPC and POPE were naturally present in the lung samples, we searched for the accurate mass of PC HOCl, PC A–L, PE HOCl and PE A–F in the total ion chromatograms (TICs) (**Table 1**). The TICs were also evaluated for the presence of other chlorinated PCs. The chlorinated PCs were distinguished from the unmodified PCs based on the accurate masses and their isotopic patterns; the patterns had to indicate that ^{37}Cl is present in the molecule, and the monoisotopic peak and the ^{13}C-peak of the dimeric compounds had to be approximately 0.5 mass units apart.

No chlorinated lipids were detected in the untreated control sample. As expected based on previous studies (14–18), PC HOCl and PC D were found in the chlorinated lung sample (**Tables S2** and **S9** in the SM). PC A and three of the novel chlorinated lipids (PC C, F and J) were also found in the chlorinated lung sample. The fragmentation patterns of the analytes measured with LC-MS/HRMS matched with the fragmentation of the reference chemicals (**Tables S4**, **S7**, **S12**, and **S17** in the SM), suggesting that the detected analytes are chlorinated PCs derived from POPC or structural isomers of POPC. Other chlorine-containing doubly charged PCs (based on the isotopic pattern) were also observed in the chlorinated lung sample at 12.4–12.8 min, which is a typical retention time for chlorinated dimeric PCs using HILIC separation. This indicates that chlorine exposure causes dimerization of other PC molecular species as well. Logically, the structures of the chlorinated mono-PCs and peroxy-diphospholipids formed are dependent on the lipids present in the tissue upon chlorine exposure. Identification of the exact structure of all the various mono- and dimeric chlorinated lipids detected in the lung sample is difficult, due to the vast number of structurally distinct PCs (and other lipids) originally present in the sample.

We propose that all chlorinated lipids identified by us here could potentially be used as biomarkers for chlorine gas exposure, alongside with the previously reported chlorohydrins and dichlorides. However, the compounds with more than two chlorine substituents may only be found in victims exposed to high levels

For Peer Review

of chlorine gas. The chlorinated derivatives of POPC may be particularly suitable as biomarkers of chlorine gas exposure, due to the ubiquitous nature of this lipid. Nevertheless, since the overall lipid composition is tissue-dependent, other suitable lipid alternatives should be explored. Further effort should be directed toward determining whether glycerol-chlorinated lipids and chlorinated peroxy-diphosholipids can be detected in biomedical samples (e.g. BALF and NLF) in concentrations that allow verification of chlorine gas exposure. The risks of false positives due to natural chlorination by metabolically produced HOCl should also be assessed.

4 CONCLUSION

Several biomarkers of chlorine gas exposure have previously been reported, including chlorinated tyrosine and phospholipid chlorohydrins, among others. These biomarkers may be also produced as a response to inflammation caused by disease and other stressful stimuli (4–6), making unambiguous verification of chlorine exposure problematic. As such, a more holistic approach to verification should be studied, where examination of the production of various biomarkers in response to chlorine gas and other stressful stimuli, such as disease, should be considered. As demonstrated by de Bruin-Hoegée et al. (12) and Jonasson et al. (13), machine learning would be a powerful tool for distinguishing chlorine gas exposure specific lipid-biomarkers from chlorinated species produced by other sources. In this work, we aimed on expanding upon the array of known biomarkers, in hopes that the combined knowledge may eventually lead to methods that can unambiguously prove cases of chlorine gas exposure in victims.

Here, we aimed at finding novel potential biomarkers for chlorine gas exposure, using POPC, PSPC, and POPE as study material. With LC-HRMS and LC-MS/HRMS techniques, 15 novel chlorinated lipids were detected and a new structure for the previously reported (17) chlorinated PC detected as *m*/*z* 794.54611 (PC A) was proposed. Five chlorinated PCs that had at least one chlorine atom attached to the glycerol backbone, and six peroxy-diphospholipids generated from the chlorinated PCs were found. Five PEs chlorinated at the glycerol backbone were also detected. No chlorinated PSPC-related PCs were detected, suggesting that at least one unsaturated site at the acyl chain of the phospholipid is needed for chlorination to occur. The comparatively smaller number of observed chlorinated PEs indicates that while other phospholipids can be chlorinated in a manner similar to that of POPC, the reactivity of various phospholipid

For Peer Review

classes may vary widely. To determine the relevance of the novel chlorinated lipids as biomarkers, chlorination experiments using pig lung were performed *in vitro*. In addition to the previously reported POPC chlorohydrin and dichloride, PC A and three of the novel chlorinated PCs (PC C, F and J), or their structural isomers, were found in the chlorinated lung sample, showing that the chlorinated lipids discovered here by us are potential biomarkers for chlorine gas exposure.

To the best of our knowledge, this is the first time putative peroxy-diphospholipids and lipids chlorinated at the glycerol backbone are described. In addition to being novel potential biomarkers for chlorine gas exposure, the compounds might have a negative impact on biological functions and produce a toxic responses in organisms. Although we were able to verify their formation in lung tissue upon chlorination *in vitro*, further effort is needed to verify their suitability for analysis from samples obtained by convenient lung sampling methods, such as nasal lavage. The risk of false positives caused by metabolically produced HOCl must also be assessed.

For Peer Review

5 APPENDIX

Chromatograms and interpretations of the fragmentation patterns are presented in the Electronic Supplementary Material. The Electronic Supplementary Material is available online.

6 FUNDING

This work was funded by the Ministry of Foreign Affairs (Government of Finland), the Department of Public Service (Government of Malaysia), and the Doctoral Program in Chemistry and Molecular Sciences (CHEMS) at the University of Helsinki.

7 ABBREVIATIONS

(H)ESI = (heated) electrospray ionization;

(HR)MS = (high-resolution) mass spectrometry;

ACN = acetonitrile;

BALF = bronchoalveolar lavage fluid;

CW = chemical weapon;

CWC = Chemical Weapons Convention;

DAG = diacylglycerol; DCM = dichloromethane;

FA = formic acid;

HCD = high-energy dissociation;

HILIC = hydrophilic interaction liquid chromatography;

IPA = isopropanol;

LC = liquid chromatography;

MeOH = methanol;

MS/(HR)MS = (high-resolution) tandem mass spectrometry;

NLF = nasal lavage fluid;

PC = phosphatidylcholine;

PE = phosphatidylethanolamine;

PLC = phospholipase C;

POPC = 1-palmitoyl-2-oleoyl-sn-glycero-3-phosphocholine;

POPE = 1-palmitoyl-2-oleoyl-sn-glycero-3-phosphoethanolamine;

PSPC = 1-palmitoyl-2-strearoyl-sn-glycero-3-phosphocholine;

TIC = total ion chromatogram;

UHPLC = ultrahigh performance liquid chromatography;

UPLC = ultra-performance liquid chromatography;

WWI = World War I

For Peer Review

8 DECLARATIONS

Data availability

The datasets generated and analysed during the current study are available from the corresponding author, upon reasonable request.

Credit roles

Conceptualisation: Matti A. Kjellberg; Data curation: Noora-Kaisa Rantanen, Nurhazlina Hamzah, Matti A. Kjellberg; Formal analysis: Noora-Kaisa Rantanen, Nurhazlina Hamzah, Matti A. Kjellberg; Funding acquisition: Noora-Kaisa Rantanen, Paula Vanninen, Hanna Hakulinen; Investigation: Noora-Kaisa Rantanen, Nurhazlina Hamzah, Matti A. Kjellberg, Solja Säde; Methodology: Noora-Kaisa Rantanen, Matti A. Kjellberg; Resources: Paula Vanninen, Hanna Hakulinen; Supervision: Matti A. Kjellberg; Paula Vanninen Visualisation: Noora-Kaisa Rantanen, Nurhazlina Hamzah, Matti A. Kjellberg; Writing – original draft: Noora-Kaisa Rantanen, Nurhazlina Hamzah, Matti A. Kjellberg; Writing – review and editing Noora-Kaisa Rantanen, Nurhazlina Hamzah, Matti A. Kjellberg, Solja Säde, Paula Vanninen, Hanna Hakulinen:

For Peer Review

Acknowledgment

The authors thank the Governments of Finland and Malaysia, and the University of Helsinki for their financial support. The authors also wish to acknowledge Tilateurastamo Kiven Säästöpossu (luomupossu.fi) for providing the pig lung used in this study and Dr. Ivana Sofrenic for her valuable support with comments and NMR analysis. The contribution of all VERIFIN staff is highly appreciated. We thank Dr. James Thompson of Language Services at the University of Helsinki for revising the English of this manuscript.

Declaration o competing interest

The authors have no competing interests that could influence the content of this article.

9 REFERENCES

1. The Organisation for the Prohibition of Chemical Weapons (OPCW). What is a Chemical Weapon? [Internet]. 2020 [cited 2023 Nov 1]. Available from: https://www.opcw.org/our-work/what-chemical-weapon

2. Schneider T, Lütkefend T. Nowhere to Hide the Logic of Chemical Weapons Use in Syria. Glob Public Policy Intitute. 2019;

3. Todd DG. Toxicological Profile for Chlorine [Internet]. Atlanta, GA: US Department of Health and Human Services, Public Health Service, Agency for Toxic Substances and Disease Registry; 2010. Available from: https://www.atsdr.cdc.gov/ToxProfiles/tp172.pdf

4. Schröter J, Schiller J. Chlorinated Phospholipids and Fatty Acids: (Patho)physiological Relevance, Potential Toxicity, and Analysis of Lipid Chlorohydrins. Oxid Med Cell Longev [Internet]. 2016;2016:1–26. Available from: https://www.hindawi.com/journals/omcl/2016/8386362/

5. Hazen SL, Crowley JR, Mueller DM, Heinecke JW. Mass Spectrometric Quantification of 3-Chlorotyrosine in Human Tissues with Attomole Sensitivity. Free Radic Biol Med [Internet]. 1997 Jan;23(6):909–16. Available from: https://linkinghub.elsevier.com/retrieve/pii/S0891584997000841

6. Kettle AJ. Neutrophils convert tyrosyl residues in albumin to chlorotyrosine. FEBS Lett [Internet]. 1996 Jan 22;379(1):103–6. Available from: https://febs.onlinelibrary.wiley.com/doi/10.1016/0014-5793%2895%2901494-2

7. de Bruin-Hoegée M, van Damme IM, van Groningen T, van der Riet-van Oeveren D, Noort D, van Asten AC. Elucidation of in Vitro Chlorinated Tyrosine Adducts in Blood Plasma as Selective Biomarkers of Chlorine Exposure. Chem Res Toxicol [Internet]. 2022 Jun 20;35(6):1070–9. Available from: https://pubs.acs.org/doi/10.1021/acs.chemrestox.2c00053

8. Achanta S, Gentile MA, Albert CJ, Schulte KA, Pantazides BG, Crow BS, et al. Recapitulation of human pathophysiology and identification of forensic biomarkers in a translational model of

For Peer Review

chlorine inhalation injury. Am J Physiol Cell Mol Physiol [Internet]. 2024 Apr 1;326(4):L482–95. Available from: https://journals.physiology.org/doi/10.1152/ajplung.00162.2023

9. Martz S V., Wittwer M, Tan-Lin CW, Bochet CG, Brackmann M, Curty C. Influence of Chlorinating Agents on the Formation of Stable Biomarkers in Hair for the Retrospective Verification of Exposure. Anal Chem [Internet]. 2022 Dec 6;94(48):16579–86. Available from: https://pubs.acs.org/doi/10.1021/acs.analchem.2c01867

10. Pantazides BG, Crow BS, Quiñones-González J, Perez JW, Harvilchuck JA, Wallery JJ, et al. Development of a clinical assay to measure chlorinated tyrosine in hair and tissue samples using a mouse chlorine inhalation exposure model. Anal Bioanal Chem [Internet]. 2021 Mar 28;413(6):1765–76. Available from: http://link.springer.com/10.1007/s00216-020-03146-x

11. Nishio T, Toukairin Y, Hoshi T, Arai T, Nogami M. Development of an LC–MS/MS method for quantification of 3-chloro-L-tyrosine as a candidate marker of chlorine poisoning. Leg Med [Internet]. 2021 Nov;53:101939. Available from: https://linkinghub.elsevier.com/retrieve/pii/S1344622321001036

12. de Bruin-Hoegée M, van der Schans MJ, Langenberg JP, van Asten AC. Biomarker profiling in plants to distinguish between exposure to chlorine gas and bleach using LC-HRMS/MS and chemometrics. Forensic Sci Int. 2024 May;358:112022.

13. Jonasson S, Magnusson R, Wingfors H, Gustafsson Å, Rankin G, Elfsmark L, et al. Potential exhaled breath biomarkers identified in chlorine-exposed mice. J Anal Toxicol. 2024 Mar 28;48(3):171–9.

14. Spickett CM. Chlorinated lipids and fatty acids: An emerging role in pathology. Pharmacol Ther [Internet]. 2007 Sep;115(3):400–9. Available from: https://linkinghub.elsevier.com/retrieve/pii/S0163725807001118

15. Hemström P, Larsson A, Elfsmark L, Åstot C. L-α-Phosphatidylglycerol Chlorohydrins as Potential Biomarkers for Chlorine Gas Exposure. Anal Chem [Internet]. 2016 Oct 18;88(20):9972–9. Available from: https://pubs.acs.org/doi/10.1021/acs.analchem.6b01896

For Peer Review

16. Lindén P, Jonasson S, Hemström P, Ålander L, Larsson A, Ågren L, et al. Nasal Lavage Fluid as a Biomedical Sample for Verification of Chlorine Exposure. J Anal Toxicol [Internet]. 2022 May 20;46(5):559–66. Available from: https://academic.oup.com/jat/article/46/5/559/6296464

17. Hoshioka Y, Abe H, Yajima D, Makino Y, Yamaguchi R, Saitoh H, et al. The composition of chlorinated or oxidized phosphatidylcholine products changes with hypochlorite concentration: Application to abscess lipid analysis. Leg Med [Internet]. 2020 Sep;46:101724. Available from: https://linkinghub.elsevier.com/retrieve/pii/S1344622320300584

18. Ford DA, Honavar J, Albert CJ, Duerr MA, Oh JY, Doran S, et al. Formation of chlorinated lipids post-chlorine gas exposure. J Lipid Res [Internet]. 2016 Aug;57(8):1529–40. Available from: https://linkinghub.elsevier.com/retrieve/pii/S0022227520352238

19. Hemström P, Jugg B, Watkins R, Jonasson S, Elfsmark L, Rutter S, et al. Phospholipid chlorohydrins as chlorine exposure biomarkers in a large animal model. Toxicol Lett. 2024;391.

20. Guthrie JW. General Considerations when Dealing with Biological Fluid Samples. In: Comprehensive Sampling and Sample Preparation [Internet]. Elsevier; 2012. p. 1–19. Available from: https://linkinghub.elsevier.com/retrieve/pii/B978012381373200065X

21. Chaurio R, Janko C, Muñoz L, Frey B, Herrmann M, Gaipl U. Phospholipids: Key Players in Apoptosis and Immune Regulation. Molecules [Internet]. 2009 Nov 30;14(12):4892–914. Available from: http://www.mdpi.com/1420-3049/14/12/4892

22. Lorent JH, Levental KR, Ganesan L, Rivera-Longsworth G, Sezgin E, Doktorova M, et al. Plasma membranes are asymmetric in lipid unsaturation, packing and protein shape. Nat Chem Biol [Internet]. 2020 Jun 1;16(6):644–52. Available from: https://www.nature.com/articles/s41589-020-0529-6

23. Nkadi PO, Merritt TA, Pillers DAM. An overview of pulmonary surfactant in the neonate: Genetics, metabolism, and the role of surfactant in health and disease. Mol Genet Metab [Internet]. 2009 Jun;97(2):95–101. Available from: https://linkinghub.elsevier.com/retrieve/pii/S1096719209000353

For Peer Review

24. Weaver TE, Nogee LM, Jobe AH. Surfactant During Lung Development. In: Fetal and Neonatal Lung Development. Cambridge University Press; 2016. p. 141–63.

25. Pulfer M, Murphy RC. Electrospray mass spectrometry of phospholipids. Mass Spectrom Rev [Internet]. 2003 Sep 26;22(5):332–64. Available from: https://analyticalsciencejournals.onlinelibrary.wiley.com/doi/10.1002/mas.10061

26. HALL GE, UBERTINI FM. THE CHLORINATION OF DIETHYL ETHER AT LOW TEMPERATURES 1,2. J Org Chem [Internet]. 1950 Jul 1;15(4):715–9. Available from: https://pubs.acs.org/doi/abs/10.1021/jo01150a002

27. Li A, Hines KM, Xu L. Lipidomics by HILIC-Ion Mobility-Mass Spectrometry. In 2020. p. 119–32. Available from: http://link.springer.com/10.1007/978-1-0716-0030-6_7

28. Buszewski B, Noga S. Hydrophilic interaction liquid chromatography (HILIC)—a powerful separation technique. Anal Bioanal Chem [Internet]. 2012 Jan 31;402(1):231–47. Available from: http://link.springer.com/10.1007/s00216-011-5308-5

29. Todd GD, Ruiz P, Cseh L, Tucker P, Doyle J, Llados FT, et al. Toxicological Profile for Chlorine. 2010. Report No.: 7782-50–5.

30. Jakob H, Leininger S, Lehmann T, Jacobi S, Gutewort S. Peroxo Compounds, Inorganic. In: Ullmann's Encyclopedia of Industrial Chemistry [Internet]. Weinheim, Germany: Wiley-VCH Verlag GmbH & Co. KGaA; 2007. Available from: https://onlinelibrary.wiley.com/doi/10.1002/14356007.a19_177.pub2

31. Brügger B, Erben G, Sandhoff R, Wieland FT, Lehmann WD. Quantitative analysis of biological membrane lipids at the low picomole level by nano-electrospray ionization tandem mass spectrometry. Proc Natl Acad Sci [Internet]. 1997 Mar 18;94(6):2339–44. Available from: https://pnas.org/doi/full/10.1073/pnas.94.6.2339

32. Kensil CR, Dennis EA. Alkaline hydrolysis of phospholipids in model membranes and the dependence on their state of aggregation. Biochemistry [Internet]. 1981 Oct 13;20(21):6079–85. Available from: https://pubs.acs.org/doi/abs/10.1021/bi00524a025

For Peer Review

33. Urbina P, Flores-Díaz M, Alape-Girón A, Alonso A, Goni FM. Phospholipase C and sphingomyelinase activities of the Clostridium perfringens α-toxin. Chem Phys Lipids [Internet]. 2009 May;159(1):51–7. Available from: https://linkinghub.elsevier.com/retrieve/pii/S0009308409000358

34. Monturiol-Gross L, Villalta-Romero F, Flores-Díaz M, Alape-Girón A. Bacterial phospholipases C with dual activity: phosphatidylcholinesterase and sphingomyelinase. FEBS Open Bio [Internet]. 2021 Dec 8;11(12):3262–75. Available from: https://febs.onlinelibrary.wiley.com/doi/10.1002/2211-5463.13320

35. Saito R de F, Andrade LN de S, Bustos SO, Chammas R. Phosphatidylcholine-Derived Lipid Mediators: The Crosstalk Between Cancer Cells and Immune Cells. Front Immunol [Internet]. 2022 Feb 15;13. Available from: https://www.frontiersin.org/articles/10.3389/fimmu.2022.768606/full

For Peer Review

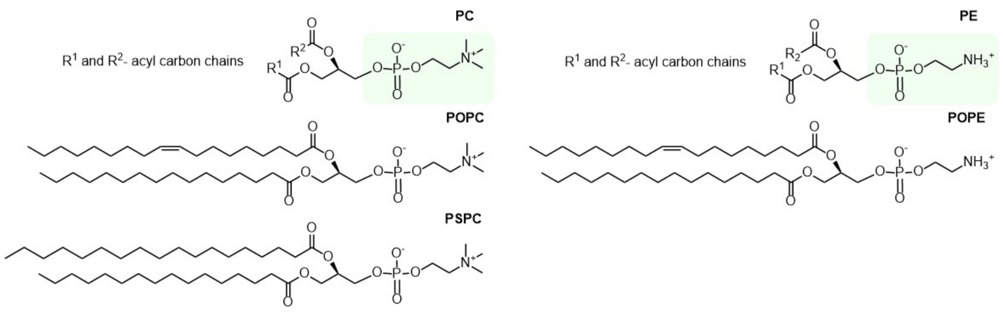

312x94mm (96 x 96 DPI)

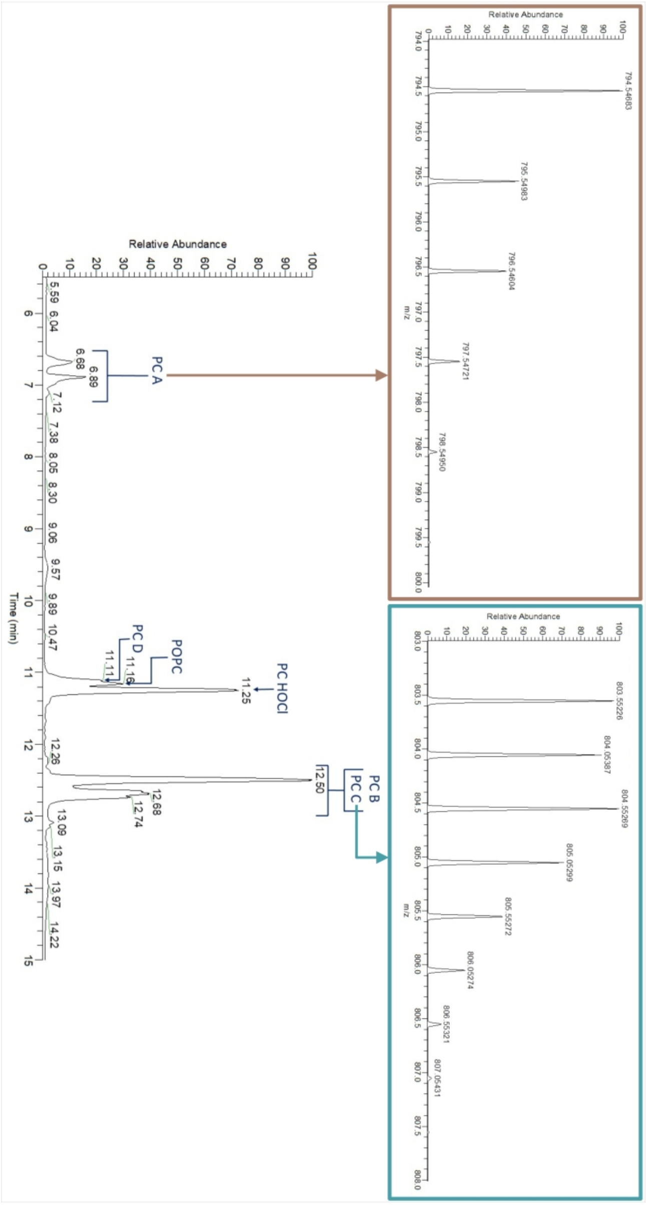

227x422mm (300 x 300 DPI)

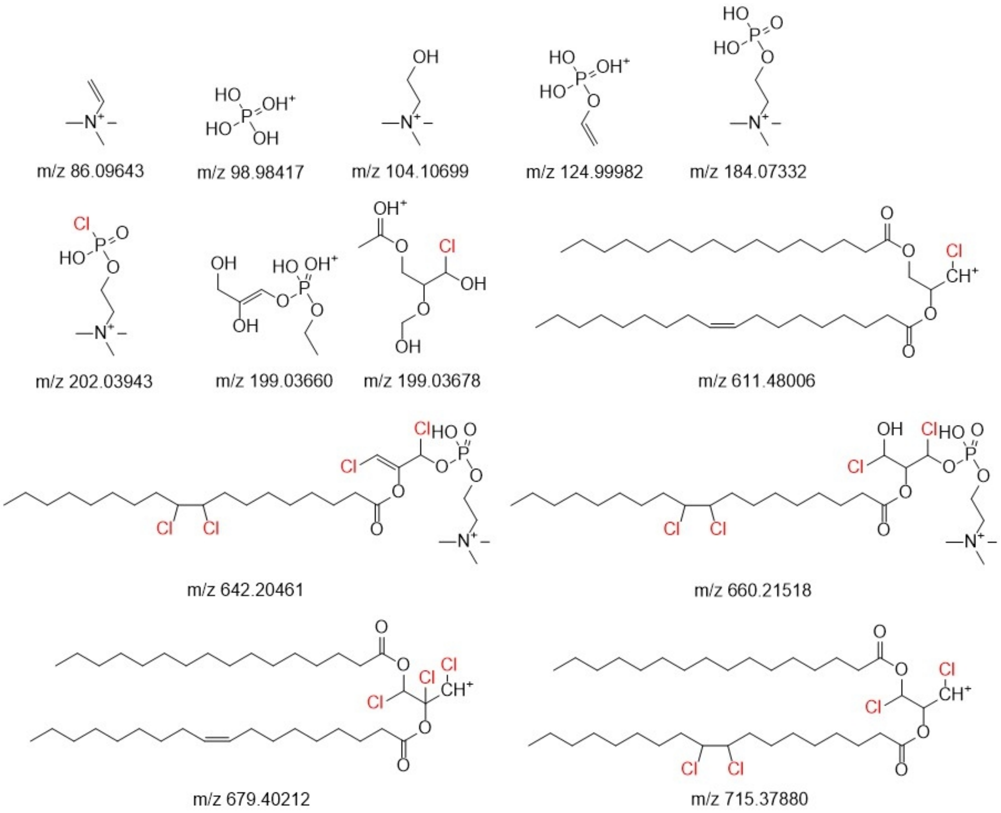

238x193mm (96 x 96 DPI)

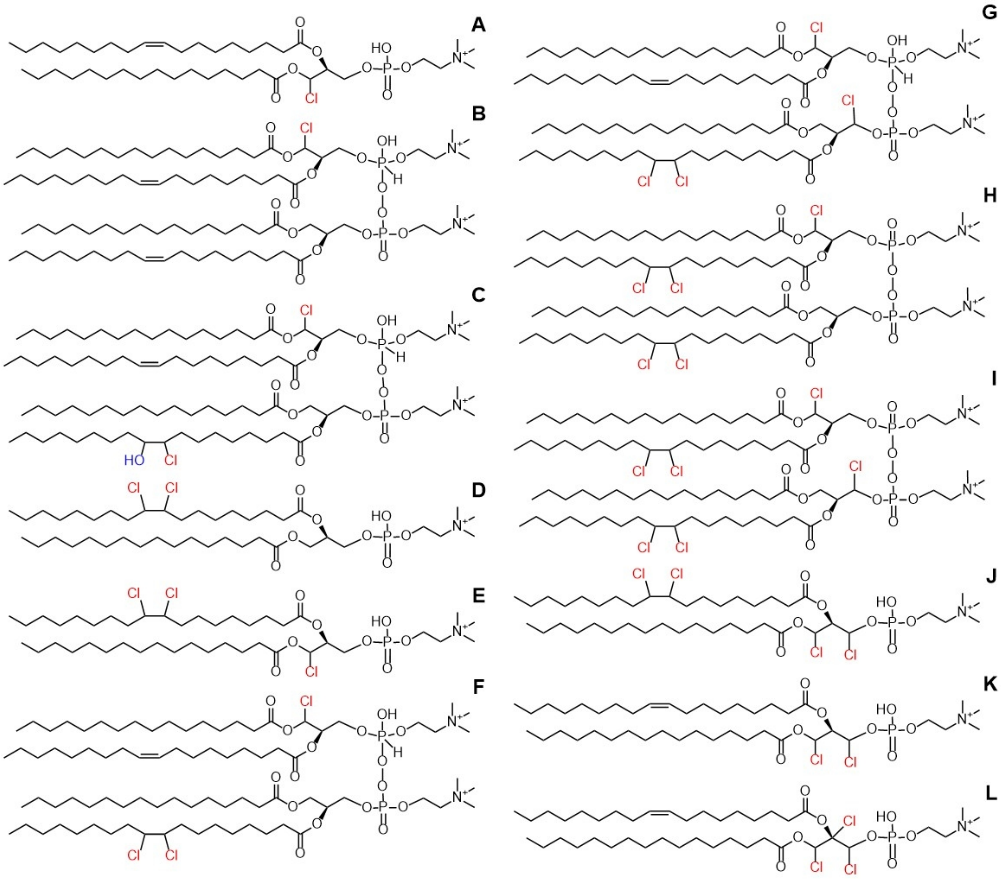

305x269mm (96 x 96 DPI)

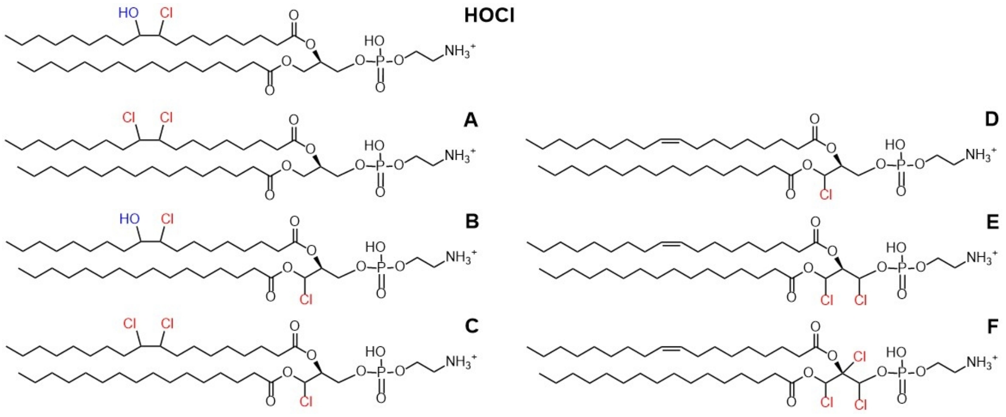

308x126mm (96 x 96 DPI)

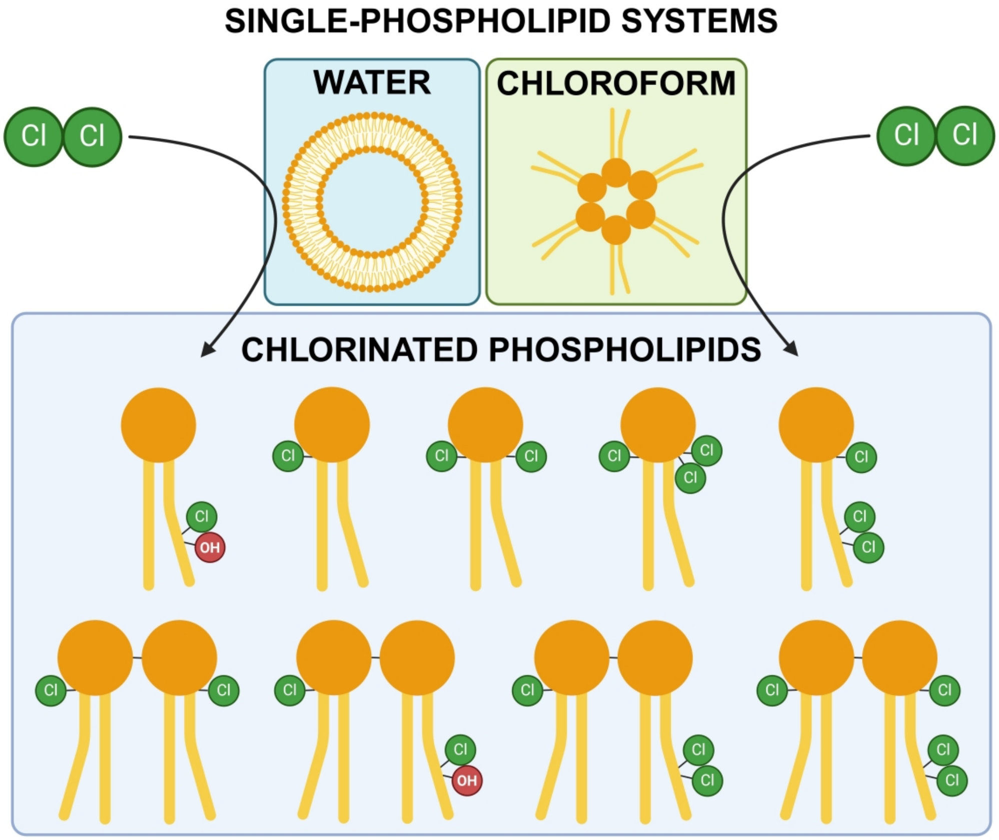

206x174mm (600 x 600 DPI)

Supplementary Material for

Novel chlorinated lipids, possible biomarkers of chlorine gas exposure

Noora-Kaisa Rantanen[a], Nurhazlina Hamzah[b], Matti A. Kjellberg[a,*], Solja Säde[a], Paula Vanninen[c] and Hanna Hakulinen[a]

a Finnish Institute for Verification of the Chemical Weapons Convention (VERIFIN), Department of Chemistry, University of Helsinki, P.O. Box 55, FI-00014 Helsinki, Finland.

b Toxicology Division, Forensic Science Analysis Centre, Department of Chemistry Malaysia, 46661 Petaling Jaya, Selangor, Malaysia

c Department of Chemistry, University of Helsinki, P.O. Box 55, FI-00014 Helsinki, Finland.

Corresponding Author: M. Kjellberg (matti.kjellberg@helsinki.fi), Finnish Institute for Verification of the Chemical Weapons Convention (VERIFIN), Department of Chemistry, University of Helsinki, P.O. Box 55, FI-00014 Helsinki, Finland

For Peer Review

Contents

1 Chromatograms of a chlorinated POPC sample 6
1.1 POPC chlorinated in chloroform 6
1.2 POPC chlorinated in water 7
1.3 POPE chlorinated in chloroform 8
1.4 POPE chlorinated in water 9
2 Isotopic patterns of analytes 9
2.1 POPC and chlorinated PCs 9
2.2 POPE and chlorinated PEs 14
3 Fragmentation patterns 18
3.1 Chlorinated PCs 18
3.1.1 Fragmentation of PC HOCl 18
3.1.2 Fragmentation of PC A 22
3.1.3 Fragmentation of PC B 27
3.1.4 Fragmentation of PC C 33
3.1.5 Fragmentation of PC D 40

3.1.6 Fragmentation of PC E 44
3.1.7 Fragmentation of PC F 47
3.1.8 Fragmentation of PC G 54
3.1.9 Fragmentation of PC H 61
3.1.10 Fragmentation of PC I 69
3.1.11 Fragmentation of PC J 77
3.1.12 Fragmentation of PC K 82
3.1.13 Fragmentation of PC L 86
3.2 Chlorinated PEs 90
3.2.1 Fragmentation of PE HOCl 90
3.2.2 Fragmentation of PE A 94
3.2.3 Fragmentation of PE B 97
3.2.4 Fragmentation of PE C 99
3.2.5 Fragmentation of PE D 103
3.2.6 Fragmentation of PE E 107
3.2.7 Fragmentation of PE F 111
4 Chromatograms of PLC-treatment 114

For Peer Review

Figures

Fig. S1 Total ion chromatogram (TIC) of a POPC sample chlorinated in chloroform separated by C18 6
Fig. S2 Total ion chromatogram (TIC) of a POPC sample chlorinated in chloroform separated by HILIC 6
Fig. S3 Total ion chromatogram (TIC) of a POPC sample chlorinated in water separated by C18 7
Fig. S4 Total ion chromatogram (TIC) of a POPC sample chlorinated in water separated by HILIC 7
Fig. S5 Total ion chromatogram (TIC) of a POPE sample chlorinated in chloroform separated by C18 8
Fig. S6 Total ion chromatogram (TIC) of a POPE sample chlorinated in chloroform separated by HILIC 8
Fig. S 7 Total ion chromatogram (TIC) of a POPE sample chlorinated in water separated by HILIC 9
Fig. S8 Isotopic pattern of POPC (from TIC of full scan) 9
Fig. S9 Isotopic pattern of PC HOCl (from TIC of full scan). 10
Fig. S10 Isotopic pattern of PC A (from TIC of full scan) 10
Fig. S11 Isotopic pattern of PC B (from TIC of full scan) 10
Fig. S12 Isotopic pattern of PC C (from TIC of full scan) 11
Fig. S13 Isotopic pattern of PC D (from TIC of full scan) 11
Fig. S14 Isotopic pattern of PC E (from TIC of full scan) 11

Fig. S15 Isotopic pattern of PC F (from TIC of full scan)....12
Fig. S16 Isotopic pattern of PC G (from TIC of full scan)....12
Fig. S17 Isotopic pattern of PC H (from TIC of full scan)....12
Fig. S18 Isotopic pattern of PC I (from TIC of full scan). The pattern has interferences from a co-eluting analyte with similar mass....13
Fig. S19 Isotopic pattern of PC J (from TIC of full scan)....13
Fig. S20 Isotopic pattern of PC K (from TIC of full scan)....13
Fig. S21 Isotopic pattern of PC L (from TIC of full scan)....14
Fig. S22 Isotopic pattern of POPE (from TIC of full scan)....14
Fig. S23 Isotopic pattern of PE HOCl (from TIC of full scan). The pattern has interferences from a co-eluting analyte....15
Fig. S24 Isotopic pattern of PE A (from TIC of full scan)....15
Fig. S25 Isotopic pattern of PE B (from TIC of full scan)....15
Fig. S26 Isotopic pattern of PE C (from TIC of full scan). The pattern has interferences from a co-eluting analyte....16
Fig. S27 Isotopic pattern of PE D (from TIC of full scan)....16
Fig. S28 Isotopic pattern of PE E (from TIC of full scan)....16
Fig. S29 Isotopic pattern of PE F (from TIC of full scan). The pattern has interferences from a co-eluting analyte....17
Fig. S30 Proposed structures of fragments PC HOCl-1–PC HOCl-15....21
Fig. S31 Proposed structures of fragments PC A-1–PC A-21....26
Fig. S32 Proposed structures of fragments PC B-1–PC B-28....32
Fig. S33 Proposed structures of fragments PC C-1–PC C-26....39
Fig. S34 Proposed structures of fragments PC D-1–PC D-11....43
Fig. S35 Proposed structures of fragments PC E-1–PC E-14....46
Fig. S36 Proposed structures of fragments PC F-1–PC F-28....53
Fig. S37 Proposed structures of fragments PC G-1–PC G-28....60
Fig. S38 Proposed structures of fragments PC H-1–PC H-31....68
Fig. S39 Proposed structures of fragments PC I-1–PC I-29....76
Fig. S40 Proposed structures of fragments PC J-1–PC J-18....81
Fig. S41 Proposed structures of fragments PC K-1–PC K-21....85
Fig. S42 Proposed structures of fragments PC L-1–PC L-24....89
Fig. S43 Proposed structures of fragments PE HOCl-1–PE HOCl-16....93
Fig. S44 Proposed structures of fragments PE A-1–PE A-12....96
Fig. S45 Proposed structures of fragments PE B-1–PE B-9....98
Fig. S46 Proposed structures of fragments PE C-1–PE C-16....102
Fig. S47 Proposed structures of fragments PE D-1–PE D-17....106
Fig. S48 Proposed structures of fragments PE E-1–PE E-20....110

For Peer Review

Fig. S49 Proposed structures of fragments PE F-1–PE F-12 113

Fig. S50 Positive precursor (parent) ion scans of *m/z* 184 (C18 LC-MS/MS) chromatograms of PLC-treated and -untreated, chlorinated aqueous POPC samples. 114

Fig. S 51 EICs (*m/z* 794.5) of positive precursor (parent) ion scans of *m/z* 184 (C18 LC-MS/MS) chromatograms of PLC-treated and -untreated, chlorinated aqueous POPC samples. 115

Fig. S52 Positive full scan (*m/z* 600-900) LC-MS EICs of *m/z* 612.5 (34:1-DAG), *m/z* 682.5 (34:1-DAG-Cl_2) and *m/z* 664.5 (34:1-DAG-OHCl) of PLC-untreated (upper chromatograms) and PLC-treated (lower chromatograms) chlorinated aqueous POPC samples. D. 117

Fig. S53 EICs (A and B = *m/z* 803.5, C and D = *m/z* 777.5) of positive precursor (parent) ion scans of *m/z* 184 (C18 LC-MS/MS) chromatograms of PLC-treated and -untreated, chlorinated aqueous POPC samples 118

Tables

Table S1 Fragments of PC HOCl (chlorinated POPC) 18

Table S2 Fragments of PC-HOCl or its isomer (chlorinated lung). Fragments that were detected in the single-lipid systems but not in the chlorinated lung sample are indicated with a hyphen (-). The ion having a m/z of 262.05064 is produced from a co-eluting matrix component with similar mass 19

Table S3 Fragments of PC A (chlorinated POPC) 22

Table S4 Fragments of PC A or its isomer (chlorinated lung) 23

Table S5 Fragments of PC B (chlorinated POPC) 27

Table S6 Fragments of PC C (chlorinated POPC) 33

Table S7 Fragments of PC C or its isomer (chlorinated lung). Fragments that were detected in the single-lipid systems but not in the chlorinated lung sample are indicated with a hyphen (-) 34

Table S8 Fragments of PC D (chlorinated POPC) 40

Table S9 Fragments of PC D or its isomer (chlorinated lung). Fragments that were detected in the single-lipid systems but not in the chlorinated lung sample are indicated with a hyphen (-) 41

Table S10 Fragments of PC E (chlorinated POPC) 44

Table S11 Fragments of PC F (chlorinated POPC) 47

Table S12 Fragments of PC F or its isomer (chlorinated lung). Fragments that were detected in the single-lipid systems but not in the chlorinated lung sample are indicated with a hyphen (-) 48

Table S13 Fragments of PC G (chlorinated POPC) 54

Table S14 Fragments of PC H (chlorinated POPC) 61

Table S15 Fragments of PC I (chlorinated POPC) 69

Table S16 Fragments of PC J (chlorinated POPC) 77

Table S17 Fragments of PC J or its isomer (chlorinated lung). Fragments that were detected in the single-lipid systems but not in the chlorinated lung sample are indicated with a hyphen (-) 78

Table S18 Fragments of PC K (chlorinated POPC) 82

Table S19 Fragments of PC L (chlorinated POPC) 86

Table S20 Fragments of PE HOCl (chlorinated POPE) 90

Table S23 Fragments of PE A (chlorinated POPE) 94

For Peer Review

Table S24 Fragments of PE B (chlorinated POPE) 97
Table S25 Fragments of PE C (chlorinated POPE) 99
Table S26 Fragments of PE D (chlorinated POPE) 103
Table S27 Fragments of PE E (chlorinated POPE) 107
Table S28 Fragments of PE F (chlorinated POPE)....................111

For Peer Review

1 Chromatograms of a chlorinated POPC sample

1.1 POPC chlorinated in chloroform

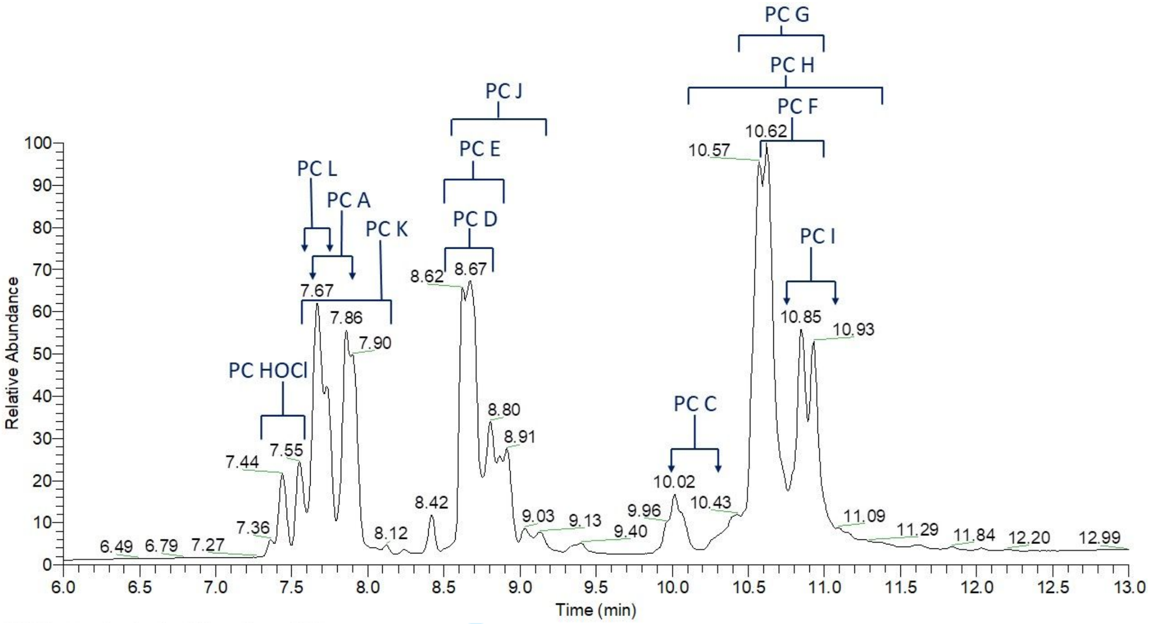

Fig. S1 Total ion chromatogram (TIC) of a POPC sample chlorinated in chloroform separated by C18

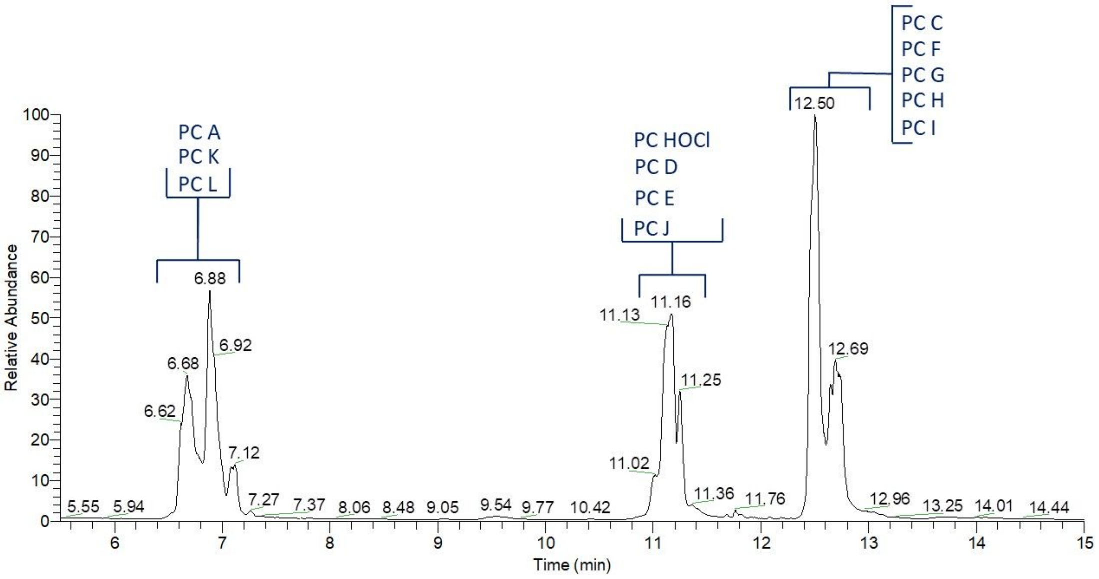

Fig. S2 Total ion chromatogram (TIC) of a POPC sample chlorinated in chloroform separated by HILIC

1.2 POPC chlorinated in water

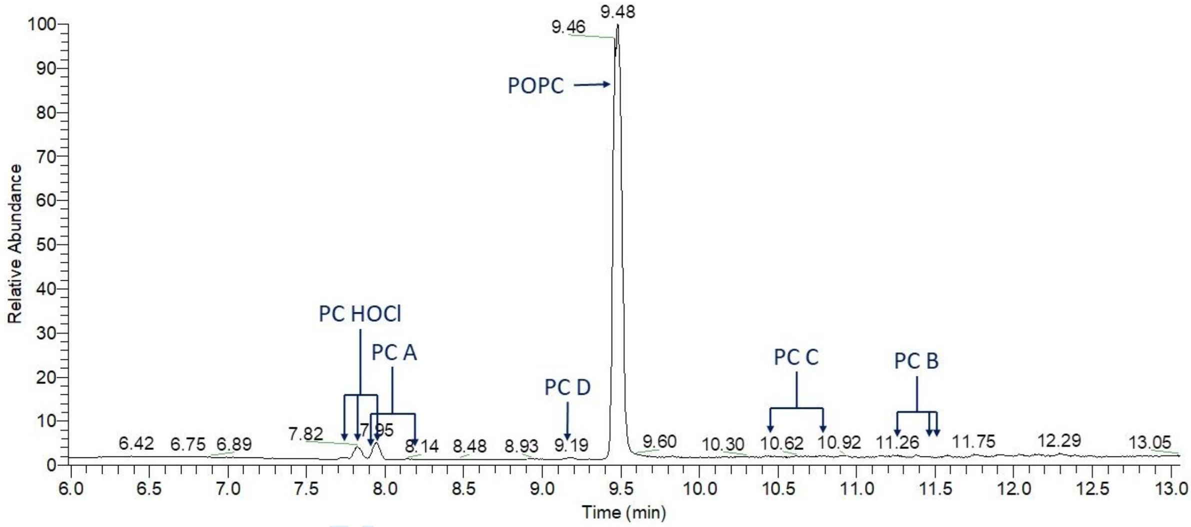

Fig. S3 Total ion chromatogram (TIC) of a POPC sample chlorinated in water separated by C18

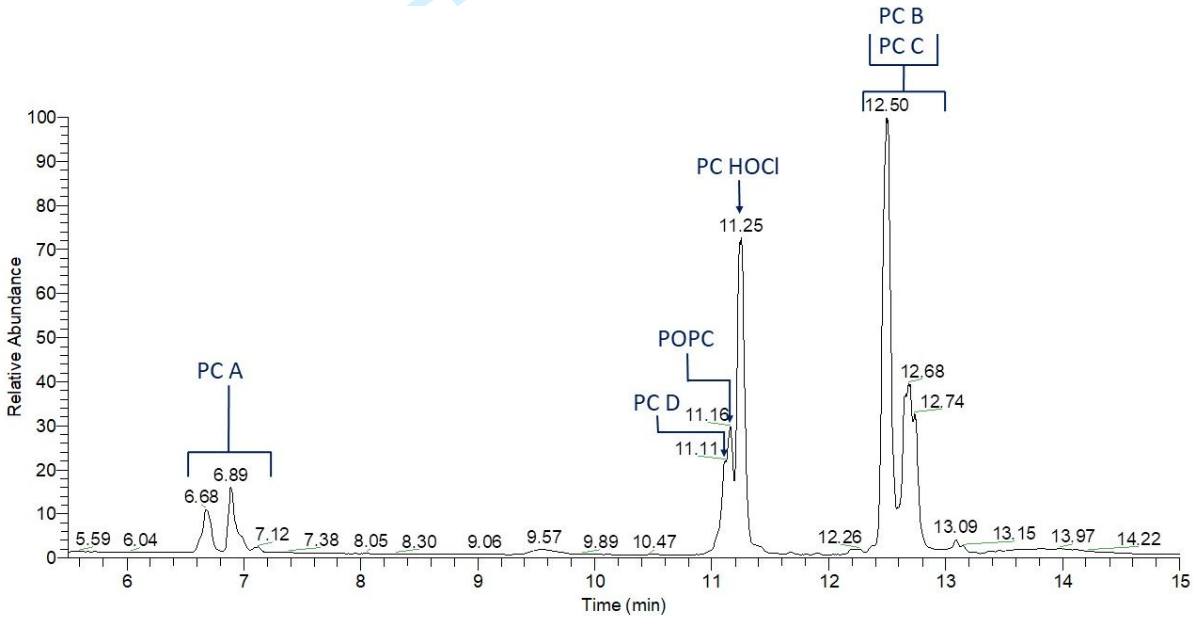

Fig. S4 Total ion chromatogram (TIC) of a POPC sample chlorinated in water separated by HILIC

1.3 POPE chlorinated in chloroform

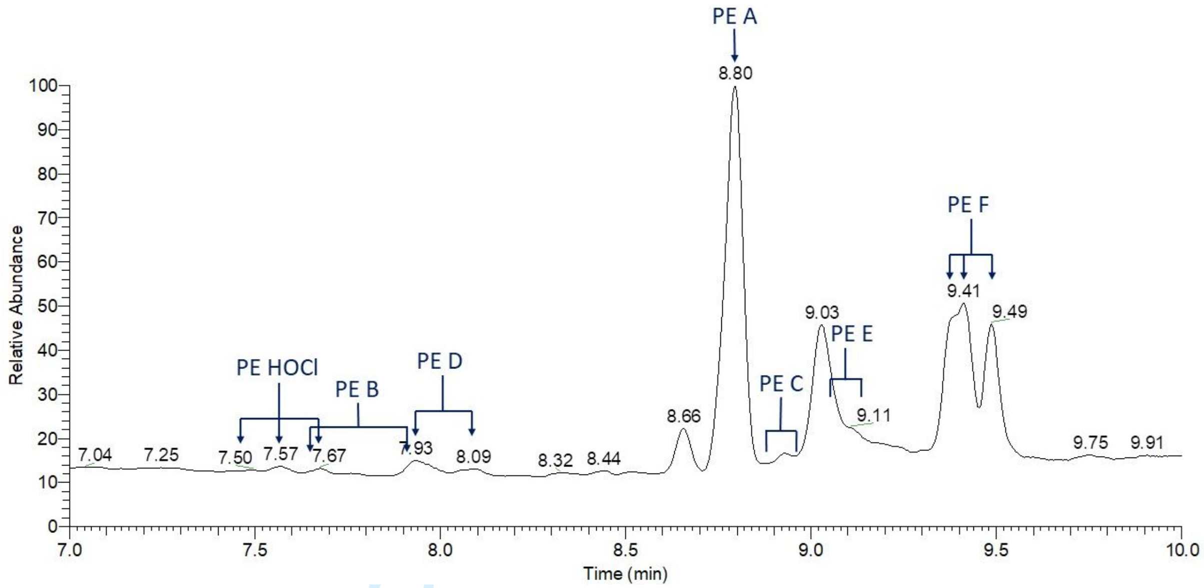

Fig. S5 Total ion chromatogram (TIC) of a POPE sample chlorinated in chloroform separated by C18

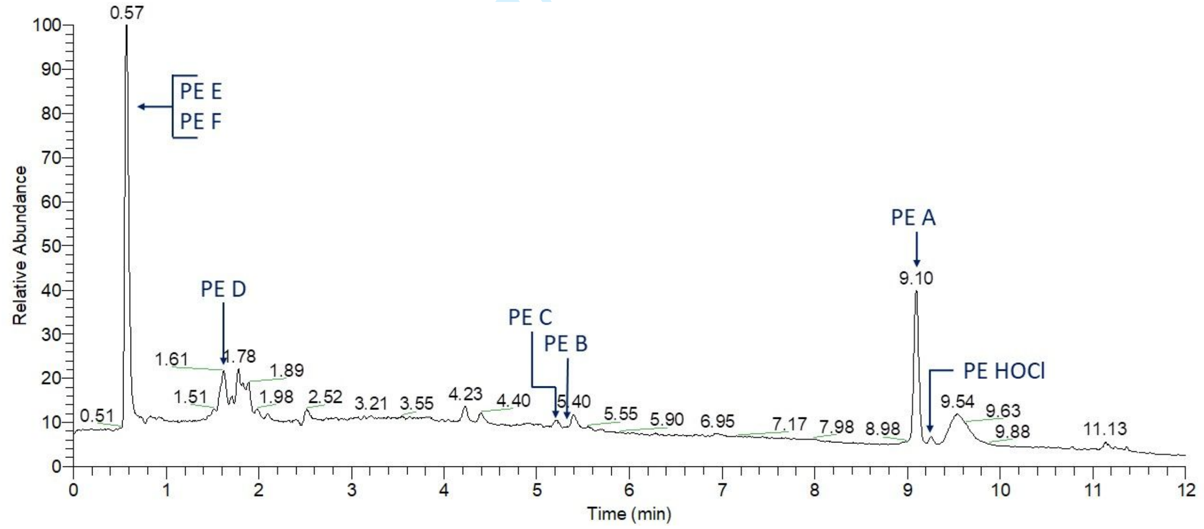

Fig. S6 Total ion chromatogram (TIC) of a POPE sample chlorinated in chloroform separated by HILIC

1.4 POPE chlorinated in water

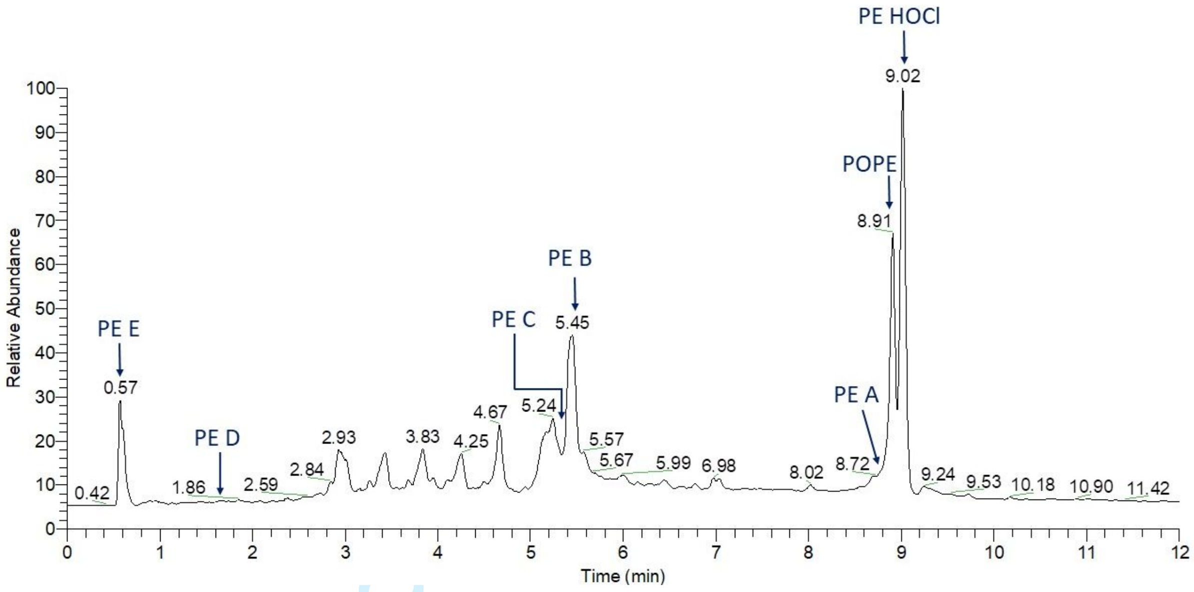

Fig. S 7 Total ion chromatogram (TIC) of a POPE sample chlorinated in water separated by HILIC

2 Isotopic patterns of analytes

2.1 POPC and chlorinated PCs

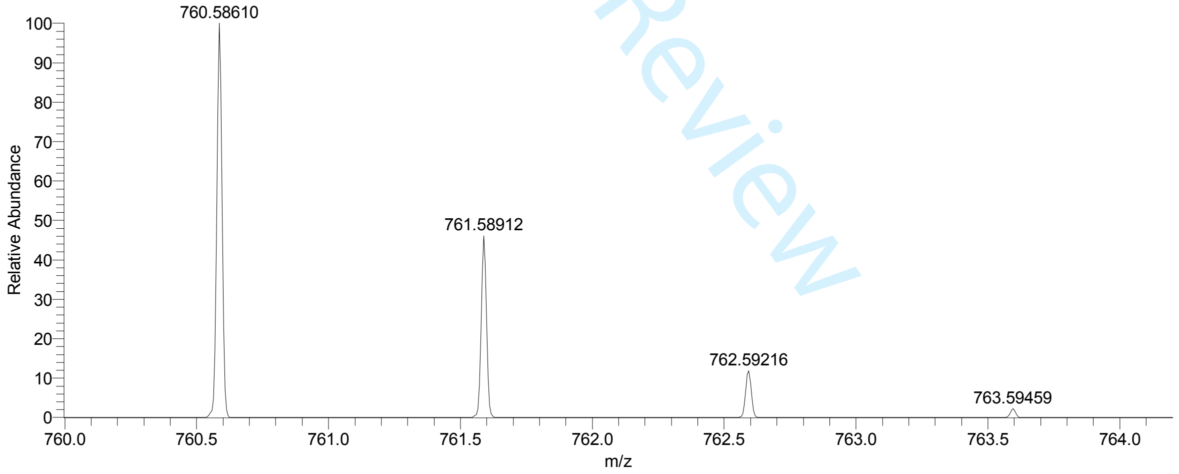

Fig. S8 Isotopic pattern of POPC (from TIC of full scan)

For Peer Review

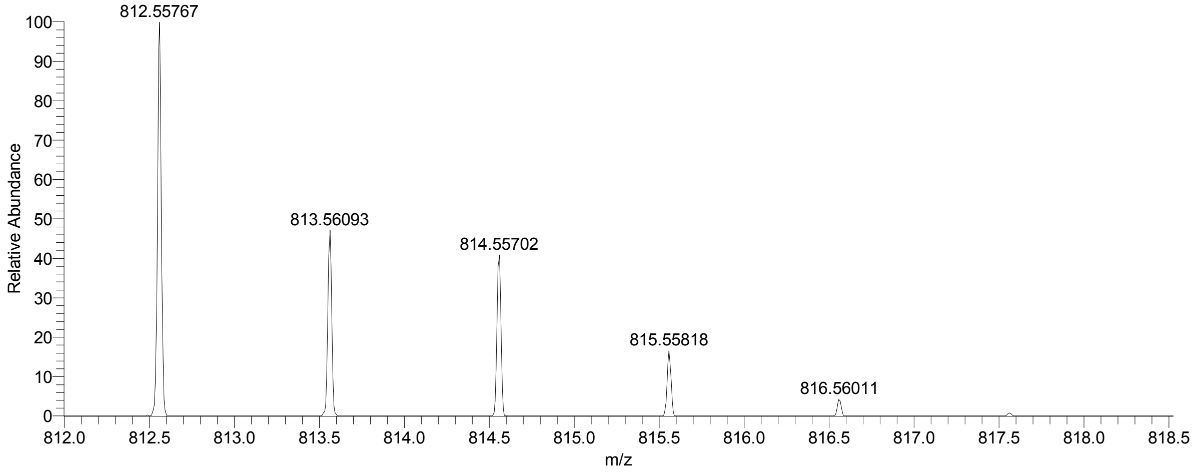

Fig. S9 Isotopic pattern of PC HOCl (from TIC of full scan)

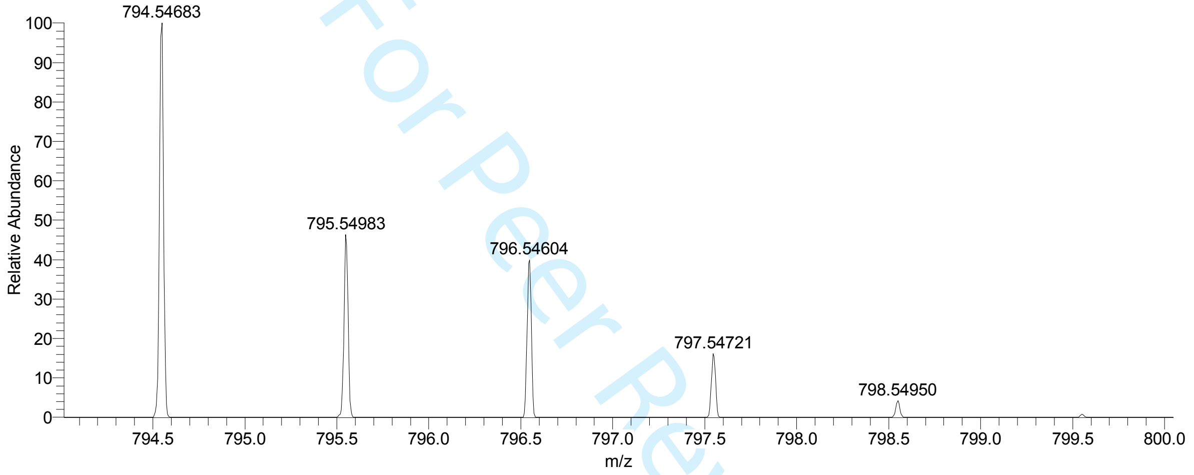

Fig. S10 Isotopic pattern of PC A (from TIC of full scan)

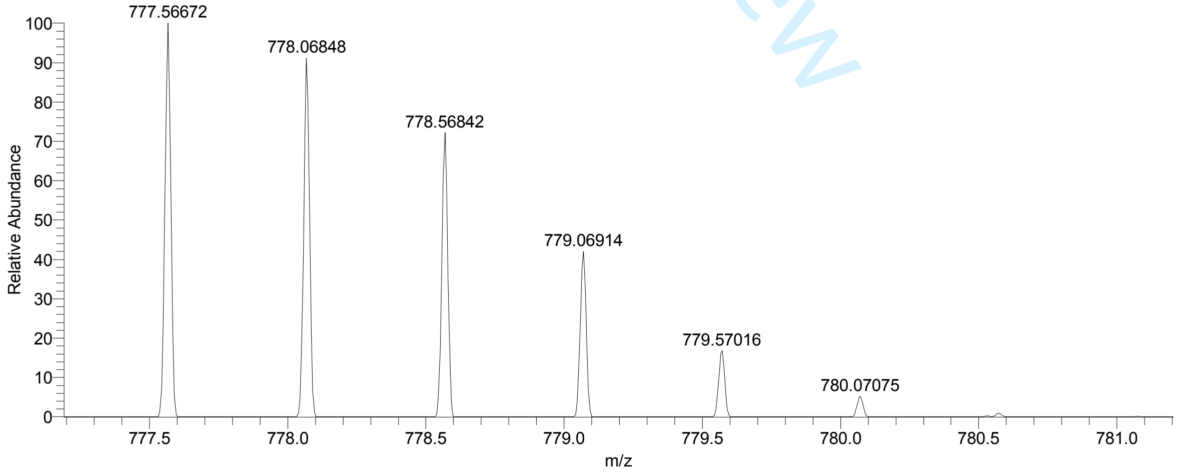

Fig. S11 Isotopic pattern of PC B (from TIC of full scan)

For Peer Review

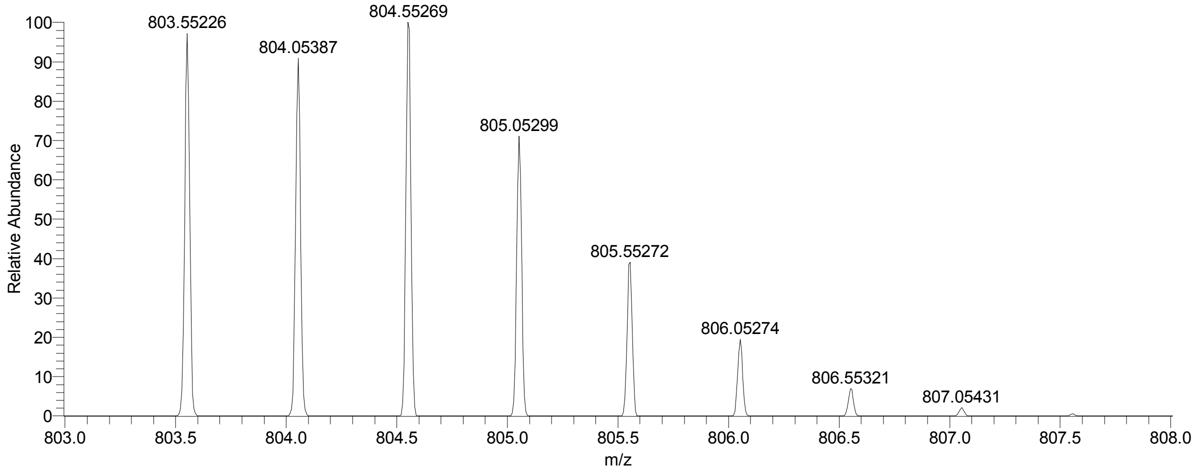

Fig. S12 Isotopic pattern of PC C (from TIC of full scan)

For Peer Review

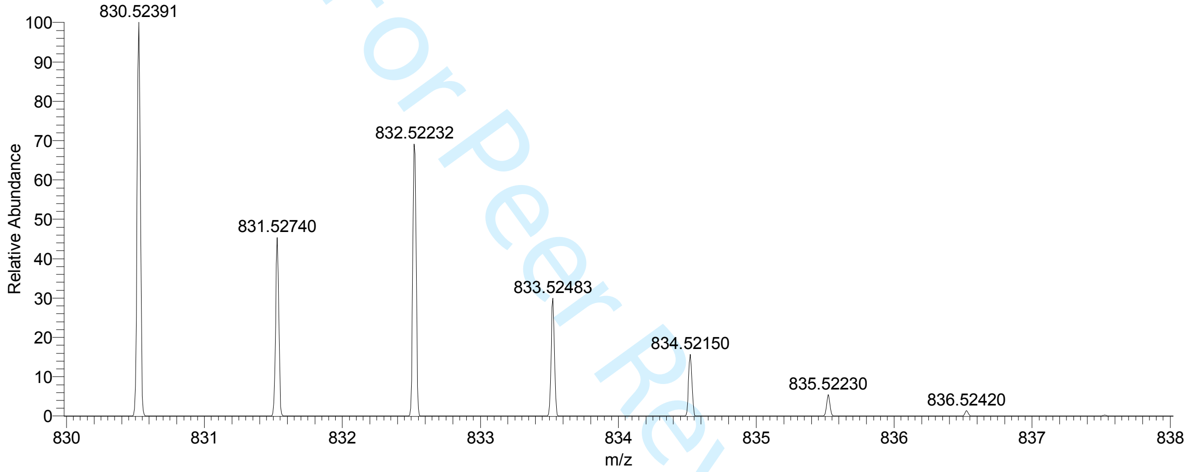

Fig. S13 Isotopic pattern of PC D (from TIC of full scan)

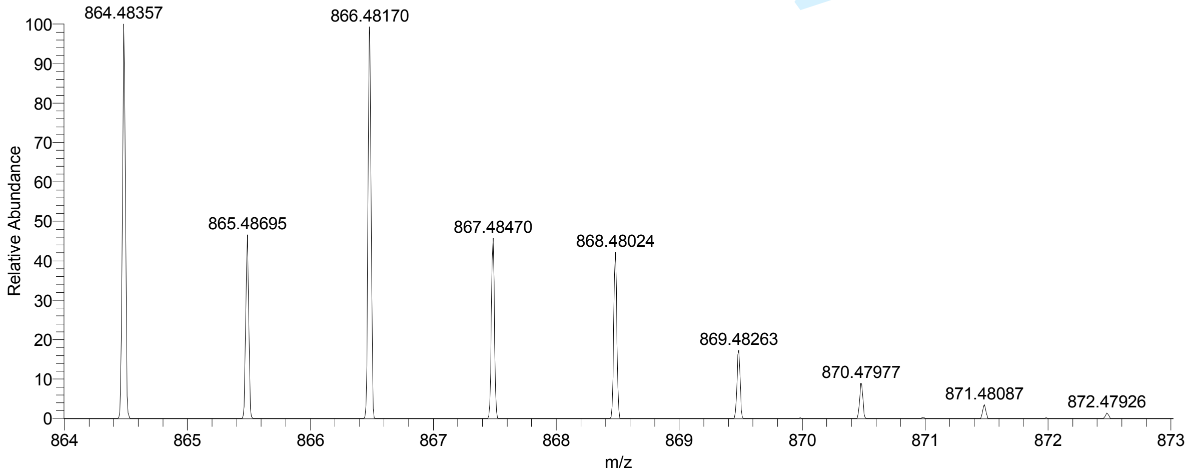

Fig. S14 Isotopic pattern of PC E (from TIC of full scan)

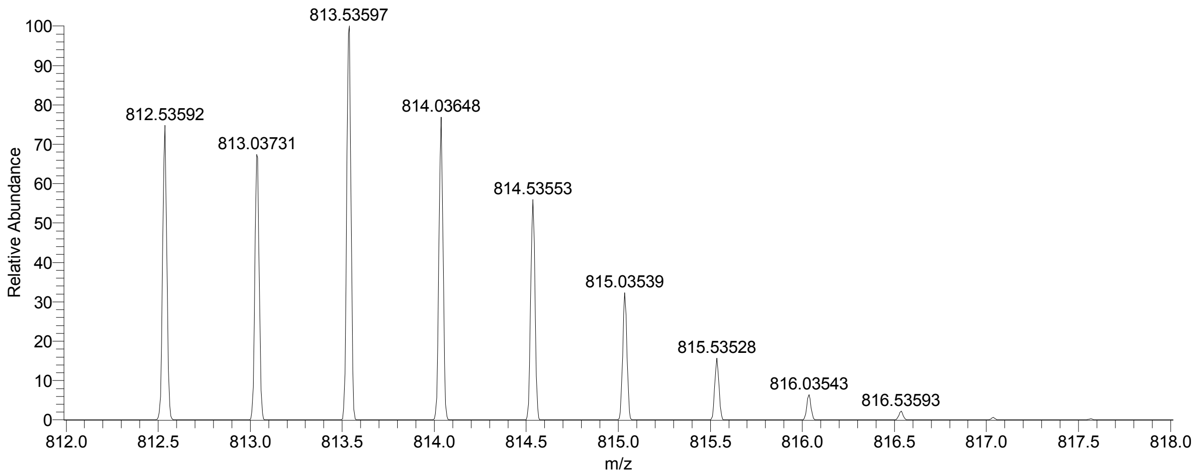

Fig. S15 Isotopic pattern of PC F (from TIC of full scan)

For Peer Review

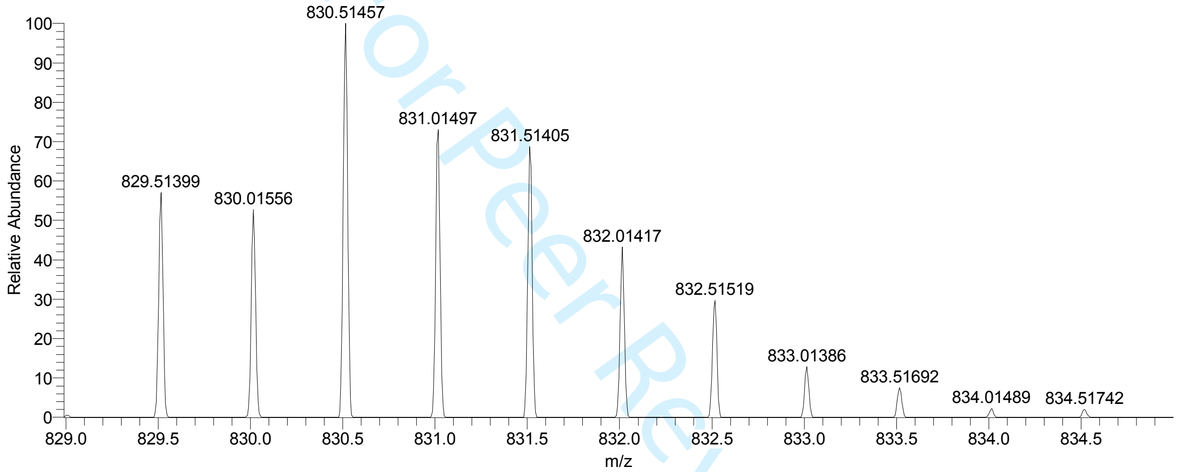

Fig. S16 Isotopic pattern of PC G (from TIC of full scan)

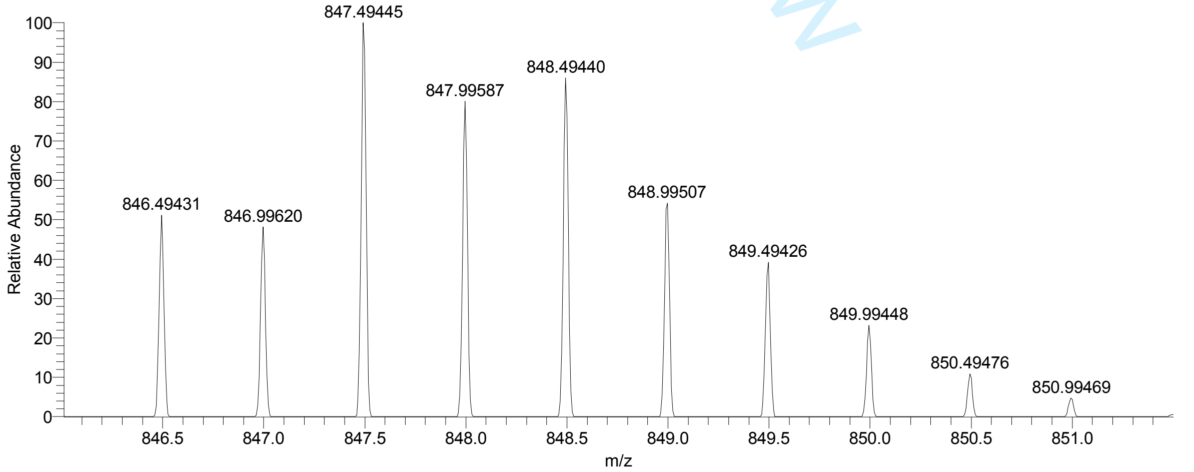

Fig. S17 Isotopic pattern of PC H (from TIC of full scan)

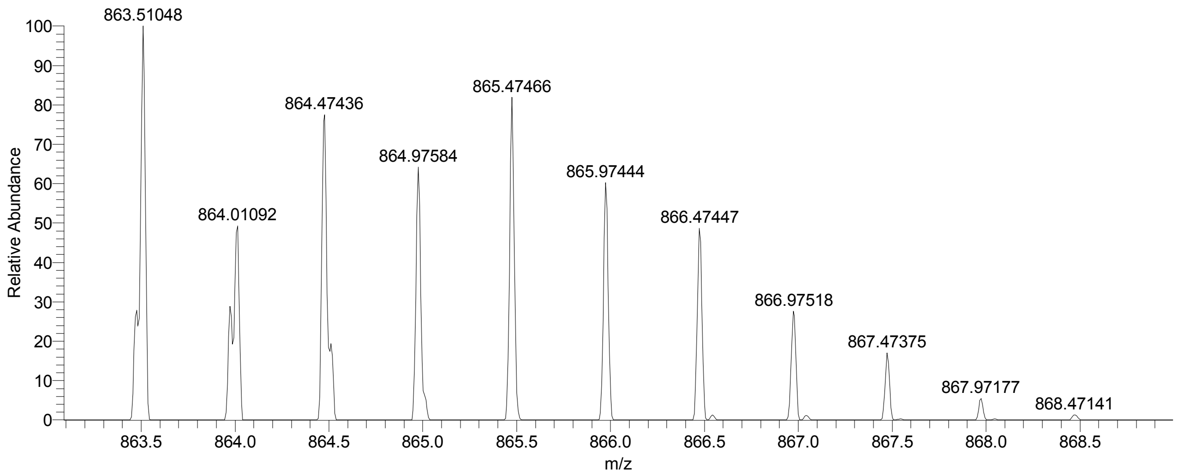

Fig. S18 Isotopic pattern of PC I (from TIC of full scan). The pattern has interferences from a co-eluting analyte with similar mass

For Peer Review

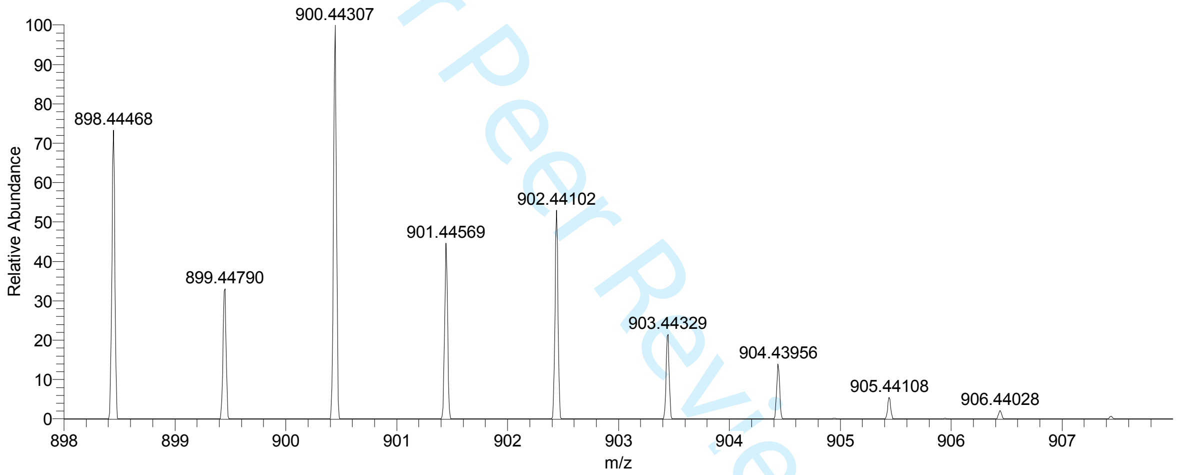

Fig. S19 Isotopic pattern of PC J (from TIC of full scan)

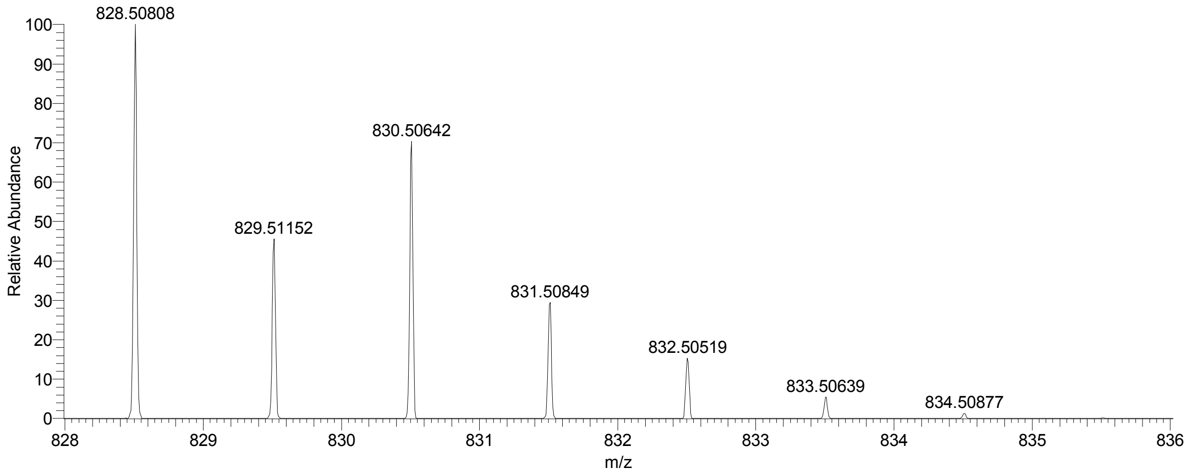

Fig. S20 Isotopic pattern of PC K (from TIC of full scan)

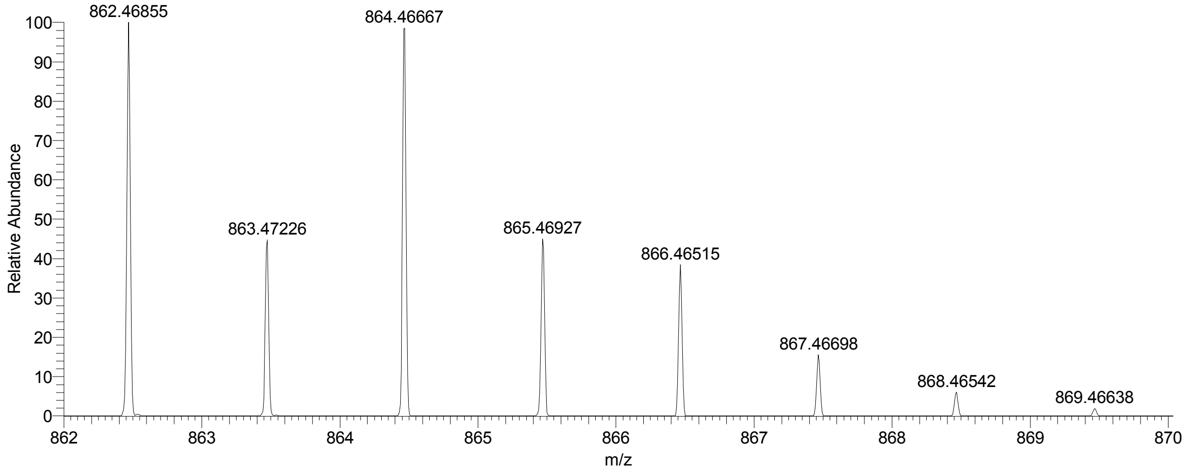

Fig. S21 Isotopic pattern of PC L (from TIC of full scan)

For Peer Review

2.2 POPE and chlorinated PEs

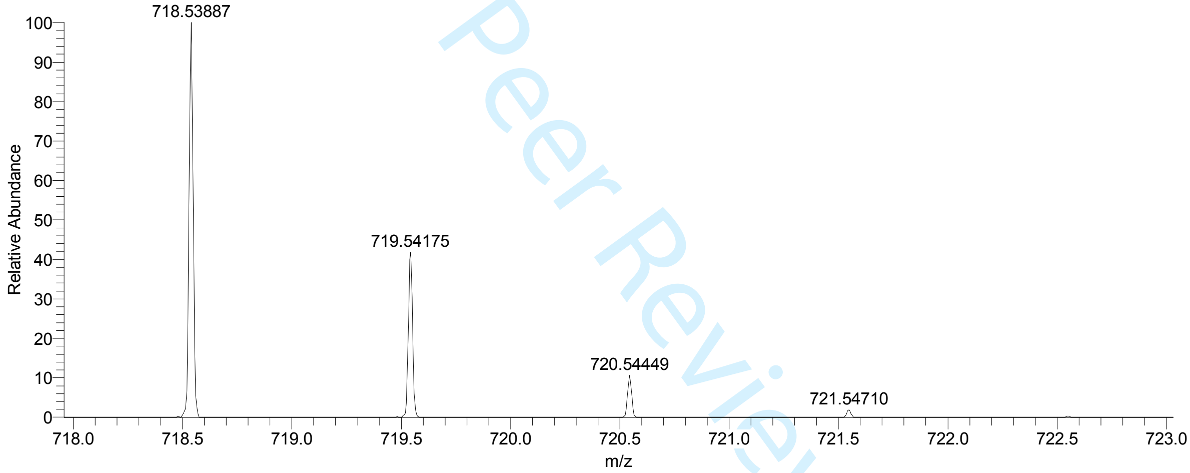

Fig. S22 Isotopic pattern of POPE (from TIC of full scan)

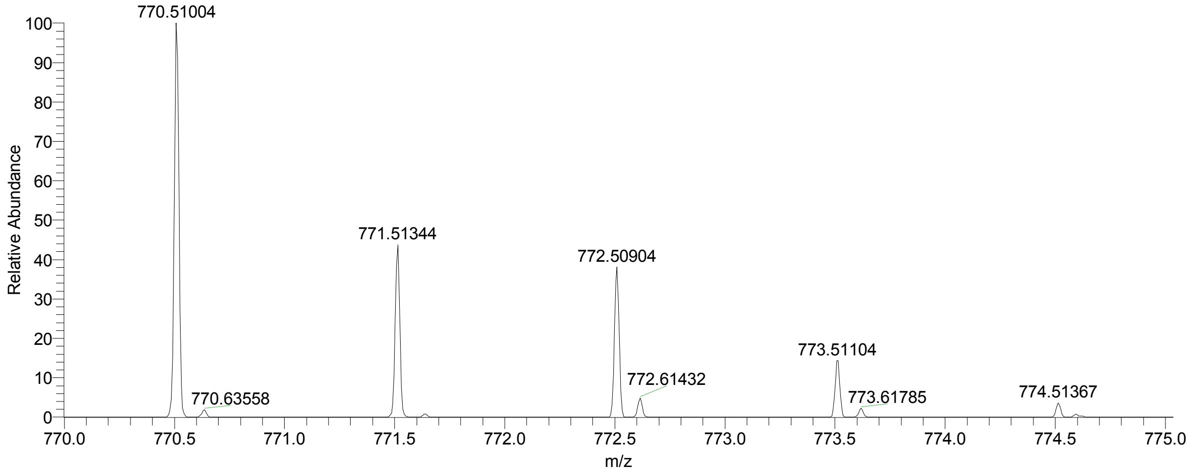

Fig. S23 Isotopic pattern of PE HOCl (from TIC of full scan). The pattern has interferences from a co-eluting analyte

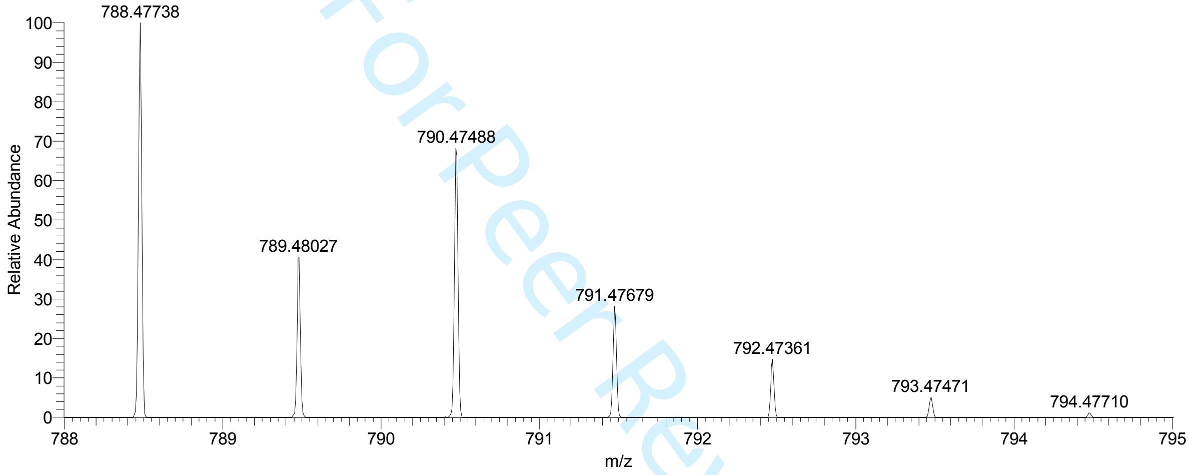

Fig. S24 Isotopic pattern of PE A (from TIC of full scan)

For Peer Review

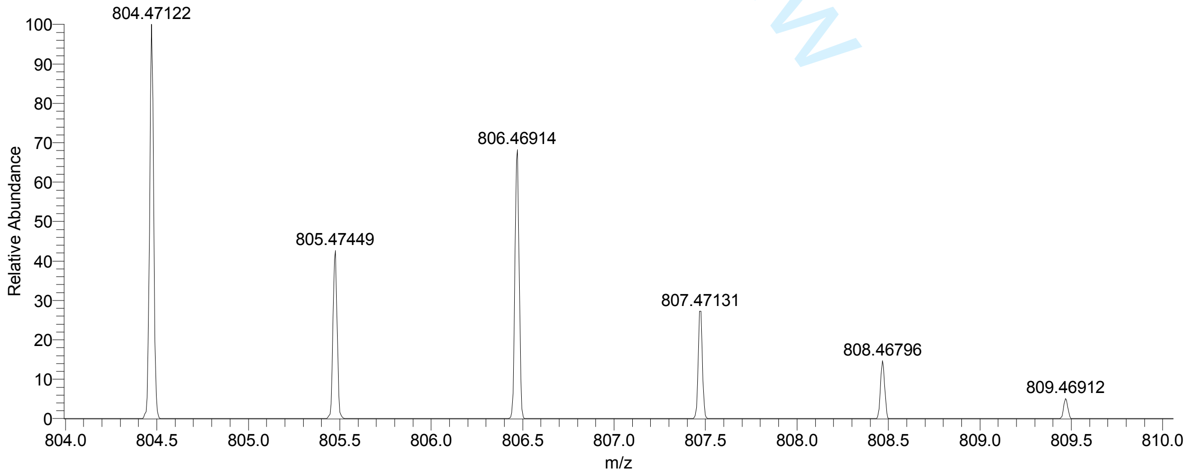

Fig. S25 Isotopic pattern of PE B (from TIC of full scan)

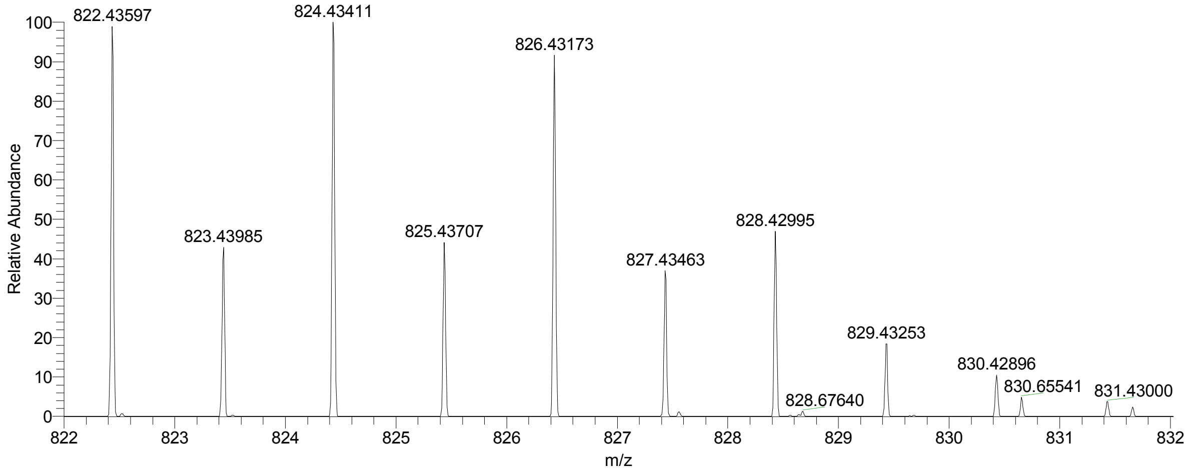

Fig. S26 Isotopic pattern of PE C (from TIC of full scan). The pattern has interferences from a co-eluting analyte

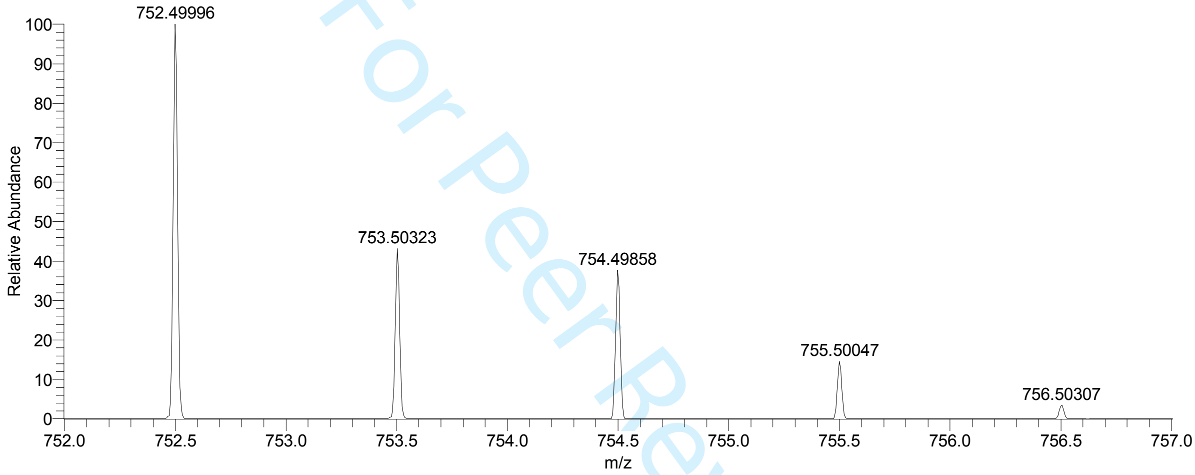

Fig. S27 Isotopic pattern of PE D (from TIC of full scan)

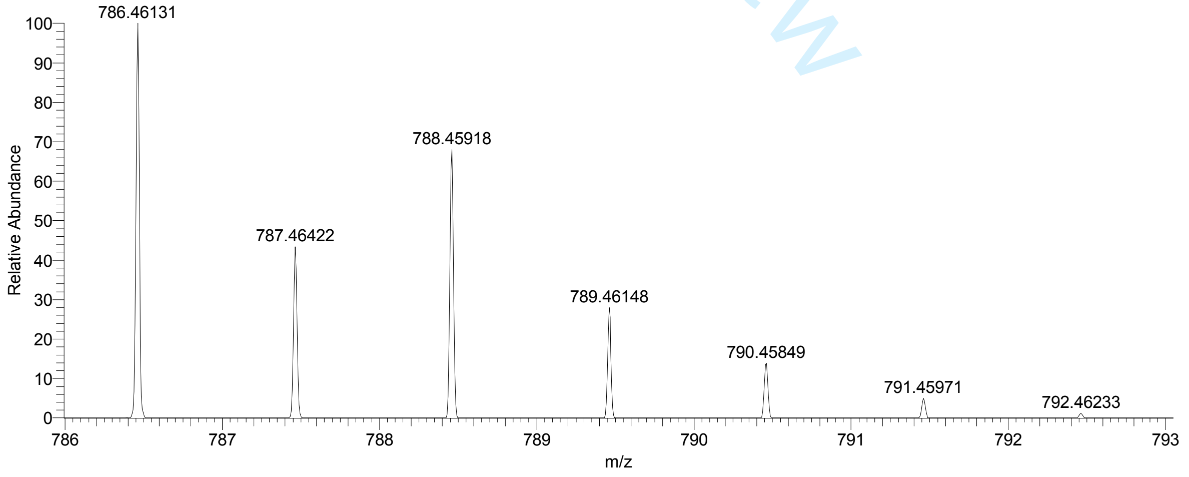

Fig. S28 Isotopic pattern of PE E (from TIC of full scan)

For Peer Review

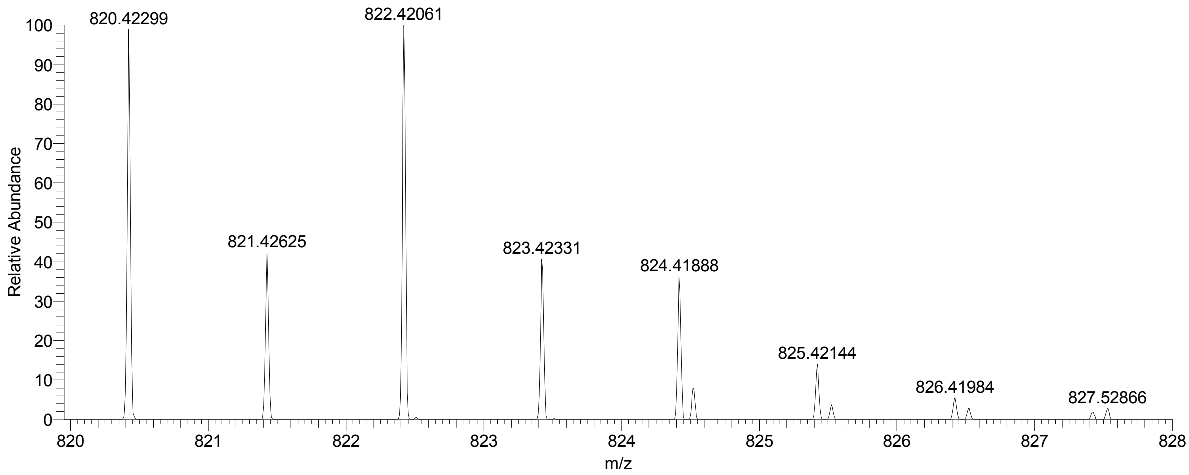

Fig. S29 Isotopic pattern of PE F (from TIC of full scan). The pattern has interferences from a co-eluting analyte

For Peer Review

3 Fragmentation patterns

3.1 Chlorinated PCs

3.1.1 Fragmentation of PC HOCl

Table S1 Fragments of PC HOCl (chlorinated POPC)

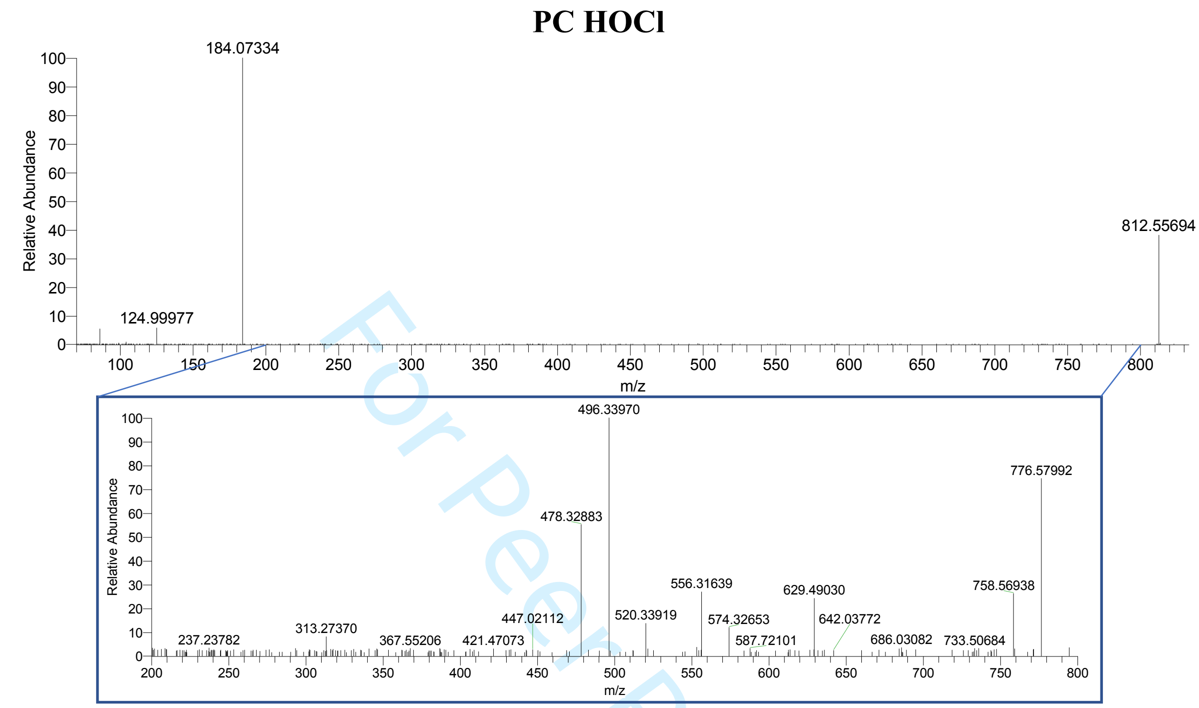

Fragment	Predicted formula	Measured *m/z*	Theoretical *m/z*	Mass difference [ppm]
PC HOCl-1	$C_{42}H_{84}O_9NClP^+$	812.55694	812.55667	0.32982
PC HOCl-2	$C_{42}H_{82}O_8NClP^+$	794.54608	794.54611	-0.03463
PC HOCl-3	$C_{42}H_{83}O_9NP^+$	776.57992	776.58000	-0.09698
PC HOCl-4	$C_{42}H_{81}O_8NP^+$	758.56938	758.56943	-0.07286
PC HOCl-5	$C_{37}H_{70}O_5Cl^+$	629.49030	629.49063	-0.52427
PC HOCl-6	$C_{26}H_{52}O_7NClP^+$	556.31639	556.31644	-0.09600
PC HOCl-7	$C_{26}H_{51}O_7NP^+$	520.33919	520.33977	-1.09901
PC HOCl-8	$C_{24}H_{51}O_7NP^+$	496.33979	496.33977	-0.12923
PC HOCl-9	$C_{24}H_{49}O_6NP^+$	478.32883	478.32920	-0.77245
PC HOCl-10	$C_{19}H_{37}O_3^+$	313.27370	313.27372	-0.05832
PC HOCl-11	$C_5H_{15}O_4NP^+$	184.07334	184.07332	0.09065
PC HOCl-12	$C_2H_6O_4P^+$	124.99977	124.99982	-0.45064
PC HOCl-13	$C_5H_{14}ON^+$	104.10691	104.10699	-0.79904
PC HOCl-14	$H_4O_4P^+$	98.98407	98.98417	-0.97996
PC HOCl-15	$C_5H_{12}N^+$	86.09637	86.09643	-0.67531

For Peer Review

Table S2 Fragments of PC-HOCl or its isomer (chlorinated lung). Fragments that were detected in the single-lipid systems but not in the chlorinated lung sample are indicated with a hyphen (-). The ion having a m/z of 262.05064 is produced from a co-eluting matrix component with similar mass

PC HOCl or its isomer

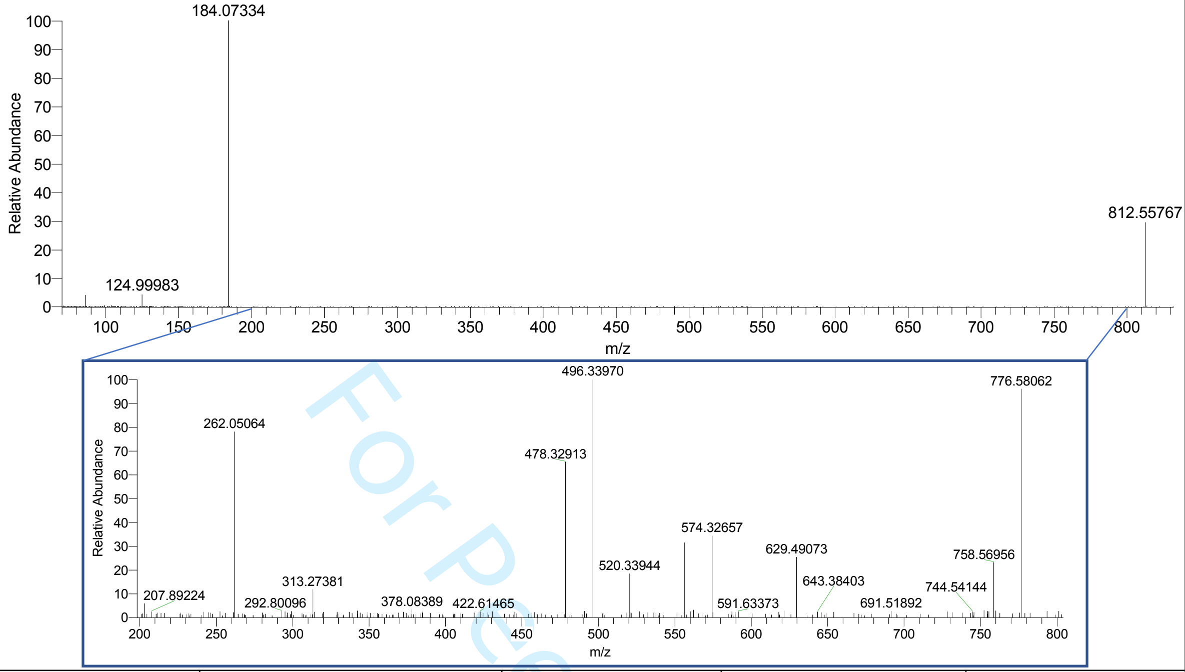

Fragment	Predicted formula	Measured *m/z*	Theoretical *m/z*	Mass difference [ppm]
PC HOCl-1	$C_{42}H_{84}O_9NClP^+$	812.55767	812.55667	1.22564
PC HOCl-2	$C_{42}H_{82}O_8NClP^+$	-	794.54611	-
PC HOCl-3	$C_{42}H_{83}O_9NP^+$	776.58062	776.58000	0.80740
PC HOCl-4	$C_{42}H_{81}O_8NP^+$	758.56956	758.56943	0.16634
PC HOCl-5	$C_{37}H_{70}O_5Cl^+$	629.49073	629.49063	0.15944
PC HOCl-6	$C_{26}H_{52}O_7NClP^+$	556.31662	556.31644	0.31716
PC HOCl-7	$C_{26}H_{51}O_7NP^+$	520.33944	520.33977	-0.63569
PC HOCl-8	$C_{24}H_{51}O_7NP^+$	496.33970	496.33977	-0.13974
PC HOCl-9	$C_{24}H_{49}O_6NP^+$	478.32913	478.32920	-0.14415
PC HOCl-10	$C_{19}H_{37}O_3^+$	313.27387	313.27372	0.29803
PC HOCl-11	$C_5H_{15}O_4NP^+$	184.07334	184.07332	0.10536
PC HOCl-12	$C_2H_6O_4P^+$	124.99983	124.99982	0.05619
PC HOCl-13	$C_5H_{14}ON^+$	104.10697	104.10699	-0.16860
PC HOCl-14	$H_4O_4P^+$	98.98417	98.98417	-0.05594
PC HOCl-15	$C_5H_{12}N^+$	86.09643	86.09643	0.03791

For Peer Review

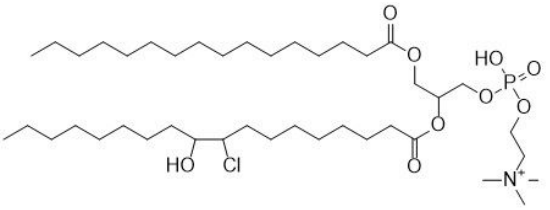

PC HOCl-1

m/z 812.55667

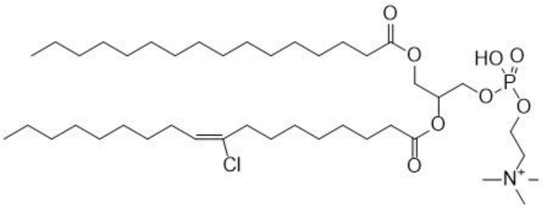

PC HOCl-2

m/z 794.54611

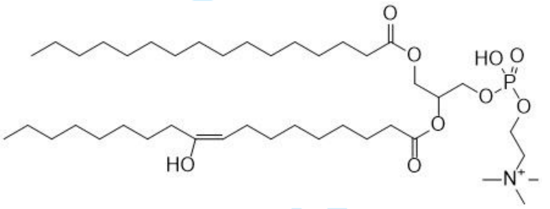

PC HOCl-3

m/z 776.58000

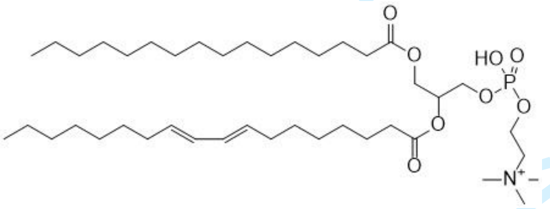

PC HOCl-4

m/z 758.56943

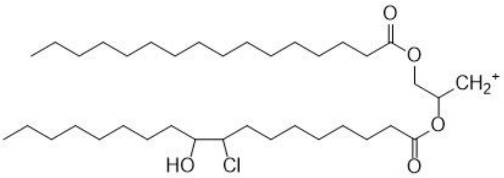

PC HOCl-5

m/z 629.49063

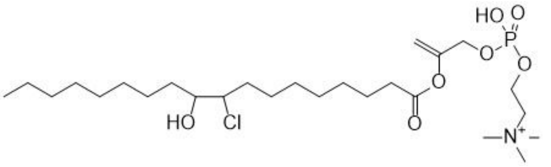

PC HOCl-6

m/z 556.31644

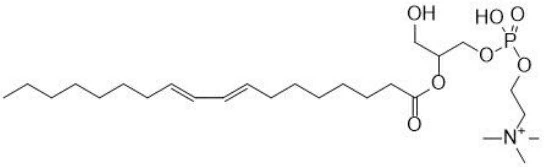

PC HOCl-7

m/*z* 520.33977

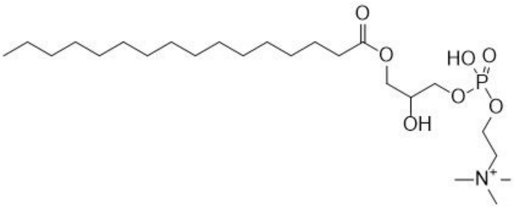

PC HOCl-8

m/*z* 496.33977

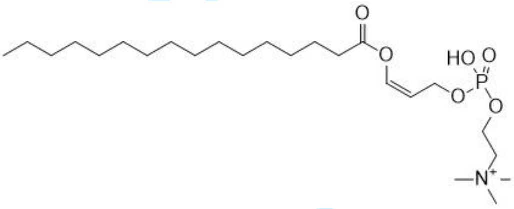

PC HOCl-9

m/*z* 478.32920

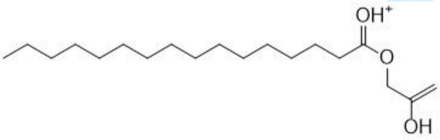

PC HOCl-10

m/*z* 313.27372

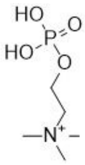

PC HOCl-11

m/*z* 184.07332

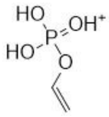

PC HOCl-12

m/*z* 124.99982

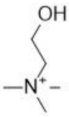

PC HOCl-13

m/*z* 104.10699

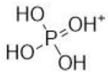

PC HOCl-14

m/*z* 98.98417

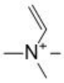

PC HOCl-15

m/*z* 86.09643

Fig. S30 Proposed structures of fragments PC HOCl-1–PC HOCl-15

3.1.2 Fragmentation of PC A

Table S3 Fragments of PC A (chlorinated POPC)

PC A

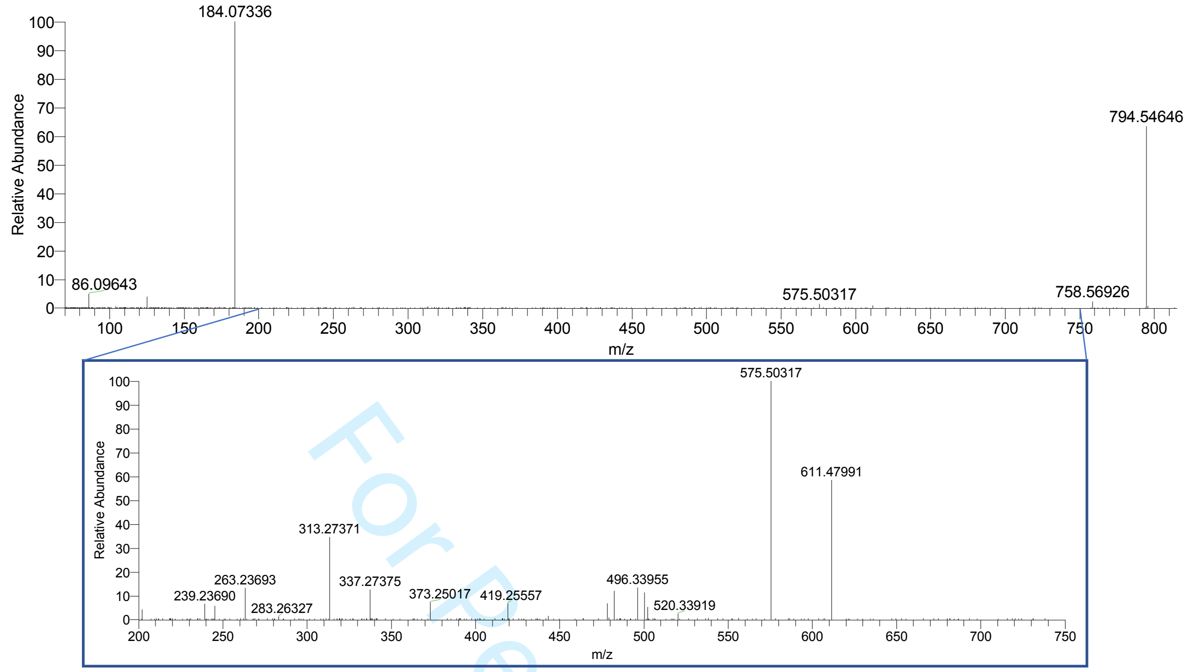

Fragment	Predicted formula	Measured *m/z*	Theoretical *m/z*	Mass difference [ppm]
PC A-1	$C_{42}H_{82}O_8NClP^+$	794.54646	794.54611	0.44387
PC A-2	$C_{42}H_{81}O_8NP^+$	758.56926	758.56943	-0.23133
PC A-3	$C_{37}H_{68}O_4Cl^+$	611.47991	611.48006	-0.25058
PC A-4	$C_{37}H_{67}O_4^+$	575.50317	575.50339	-0.37740
PC A-5	$C_{26}H_{52}O_7NClP^+$	556.31682	556.31644	0.67532
PC A-6	$C_{26}H_{50}O_6NClP^+$	538.30662	538.30588	1.38328
PC A-7	$C_{26}H_{51}O_7NP^+$	520.33919	520.33977	-1.09909
PC A-8	$C_{21}H_{40}O_6P^+$	419.25557	419.25570	-0.31520
PC A-9-1<br>PC A-9-2	$C_{21}H_{38}O_3Cl^+$<br>$C_{20}H_{38}O_4P^+$	373.25017	373.25040<br>373.25022	-0.61261<br>-0.13947
PC A-10	$C_{21}H_{37}O_3^+$	337.27375	337.27372	0.09572
PC A-11	$C_{19}H_{37}O_3^+$	313.27371	313.27372	-0.04657
PC A-12	$C_{18}H_{35}O_2^+$	283.26327	283.26316	0.40405
PC A-13	$C_{18}H_{31}O^+$	263.23693	263.23694	-0.04378
PC A-14	$C_{16}H_{31}O^+$	239.23690	239.23694	-0.15543
PC A-15	$C_5H_{14}O_3NClP^+$	202.03953	202.03943	0.46000
PC A-16-1<br>PC A-16-2	$C_6H_{12}ClO_5^+$<br>$C_5H_{12}O_6P^+$	199.03679	199.03678<br>199.03660	0.05668<br>0.94395
PC A-17	$C_5H_{15}O_4NP^+$	184.07336	184.07332	0.22552
PC A-18	$C_2H_6O_4P^+$	124.99982	124.99982	0.01891
PC A-19	$C_5H_{14}ON^+$	104.10700	104.10699	0.05873

For Peer Review

PC A-20	$H_4O_4P^+$	98.98418	98.98417	0.08355
PC A-21	$C_5H_{12}N^+$	86.09643	86.09643	0.09476

Table S4 Fragments of PC A or its isomer (chlorinated lung)

PC A or its isomer

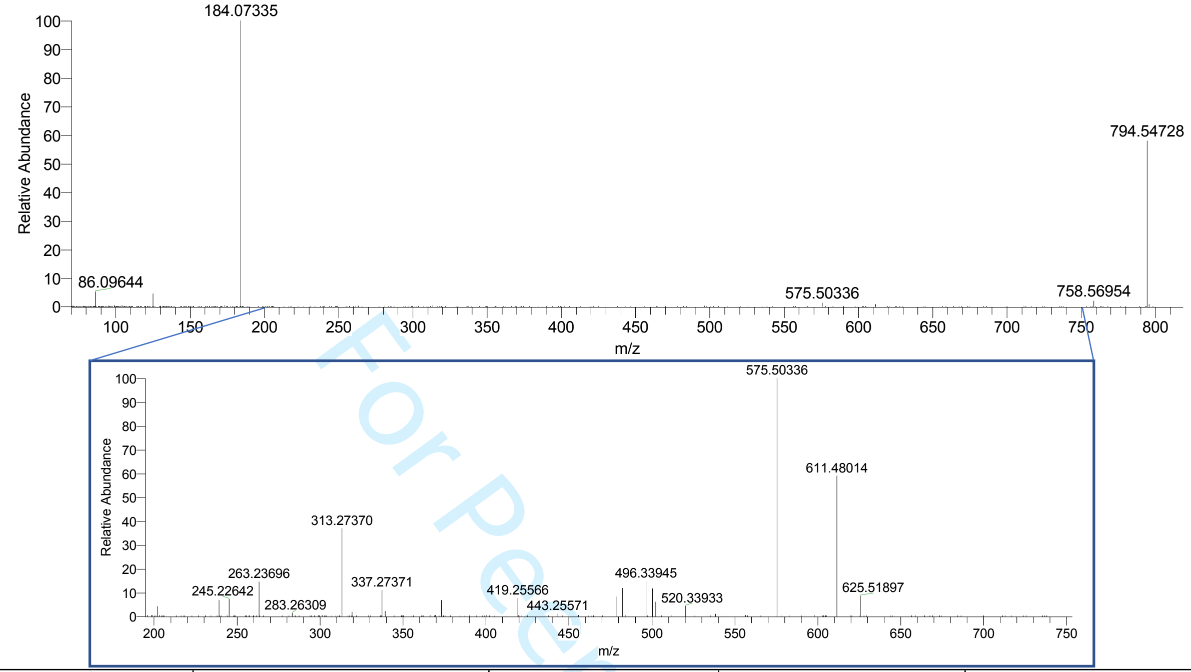

Fragment	Predicted formula	Measured *m/z*	Theoretical *m/z*	Mass difference [ppm]
PC A-1	$C_{42}H_{82}O_8NClP^+$	794.54728	794.54611	1.46960
PC A-2	$C_{42}H_{81}O_8NP^+$	758.56954	758.56943	0.14966
PC A-3	$C_{37}H_{68}O_4Cl^+$	611.48014	611.48006	0.12495
PC A-4	$C_{37}H_{67}O_4^+$	575.50336	575.50339	-0.05378
PC A-5	$C_{26}H_{52}O_7NClP^+$	556.31458	556.31644	-2.48272
PC A-6	$C_{26}H_{50}O_6NClP^+$	538.30492	538.30588	-1.78410
PC A-7	$C_{26}H_{51}O_7NP^+$	520.33933	520.33977	-0.83698
PC A-8	$C_{21}H_{40}O_6P^+$	419.25566	419.25570	-0.10375
PC A-9-1<br>PC A-9-2	$C_{21}H_{38}O_3Cl^+$<br>$C_{20}H_{38}O_4P^+$	373.25037	373.25040<br>373.25022	-0.11566<br>0.35749
PC A-10	$C_{21}H_{37}O_3^+$	337.27371	337.27372	-0.02008
PC A-11	$C_{19}H_{37}O_3^+$	313.27370	313.27372	-0.06781
PC A-12	$C_{18}H_{35}O_2^+$	283.26309	283.26316	-0.23502
PC A-13	$C_{18}H_{31}O^+$	263.23696	263.23694	0.07241
PC A-14	$C_{16}H_{31}O^+$	239.23694	239.23694	-0.00784
PC A-15	$C_5H_{14}O_3NClP^+$	202.03947	202.03943	0.19066
PC A-16-1<br>PC A-16-2	$C_6H_{12}ClO_5^+$<br>$C_5H_{12}O_6P^+$	199.03656	199.03678<br>199.03660	-1.09327<br>-0.20600
PC A-17	$C_5H_{15}O_4NP^+$	184.07335	184.07332	0.14356

For Peer Review

PC A-18	$C_2H_6O_4P^+$	124.99984	124.99982	0.12649
PC A-19	$C_5H_{14}ON^+$	104.10697	104.10699	-0.19729
PC A-20	$H_4O_4P^+$	98.98418	98.98417	0.04310
PC A-21	$C_5H_{12}N^+$	86.09644	86.09643	0.17362

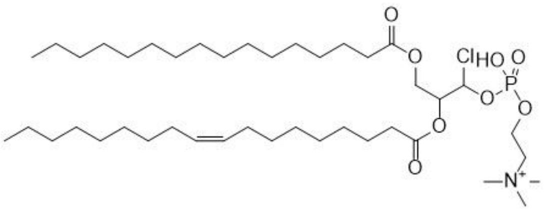

PC A-1

m/z 794.54611

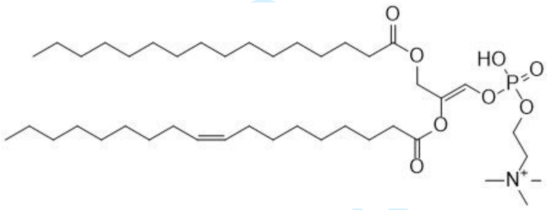

PC A-2

m/z 758.56943

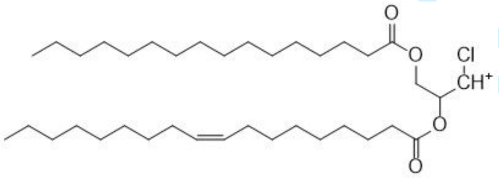

PC A-3

m/z 611.48006

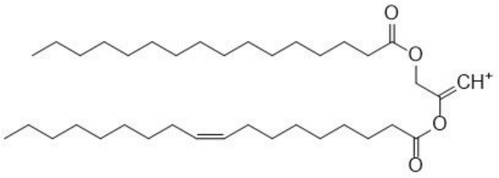

PC A-4

m/z 575.50339

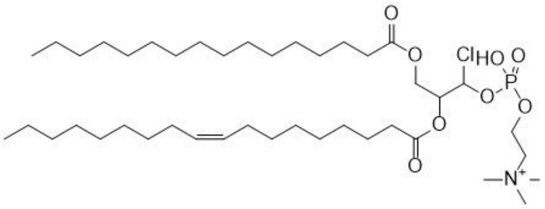

PC A-5

m/z 556.31644

For Peer Review

**PC A-6**<br>*m/z* 538.30588	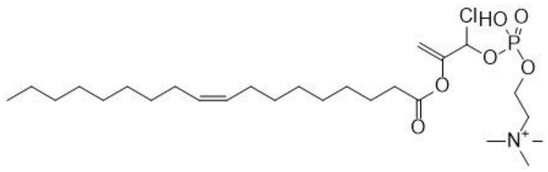
**PC A-7**<br>*m/z* 520.33977	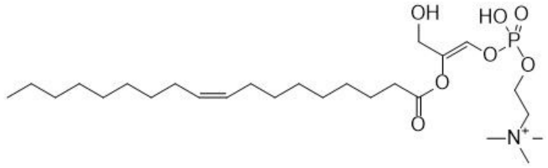
**PC A-8**<br>*m/z* 419.25570	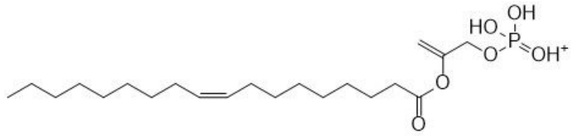
**PC A-9-1**<br>*m/z* 373.25040	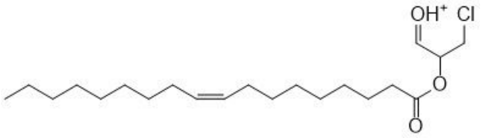
**PC A-9-2**<br>*m/z* 373.25022	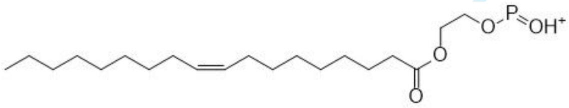
**PC A-10**<br>*m/z* 337.27372	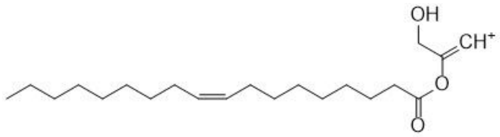
**PC A-11**<br>*m/z* 313.27372	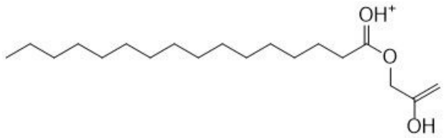
**PC A-12**<br>*m/z* 283.26316	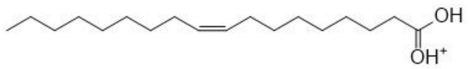

**PC A-13** 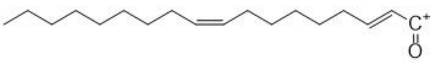<br>*m*/*z* 263.23694		
**PC A-14** 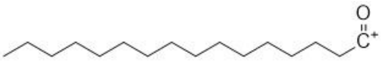<br>*m*/*z* 239.23694		
**PC A-15** 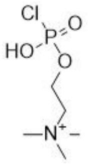<br>*m*/*z* 202.03943	**PC A-16-1** 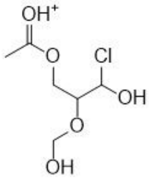<br>*m*/*z* 199.03678	**PC A-16-2** 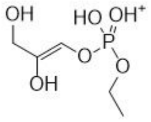<br>*m*/*z* 199.03660
**PC A-17** 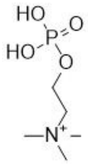<br>*m*/*z* 184.07332	**PC A-18** 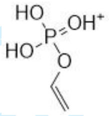<br>*m*/*z* 124.99982	**PC A-19** 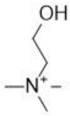<br>*m*/*z* 104.10699
**PC A-20** 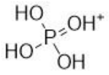<br>*m*/*z* 98.98417	**PC A-21** 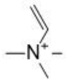<br>*m*/*z* 86.09643	

Fig. S31 Proposed structures of fragments PC A-1–PC A-21

For Peer Review

3.1.3 Fragmentation of PC B

Table S5 Fragments of PC B (chlorinated POPC)

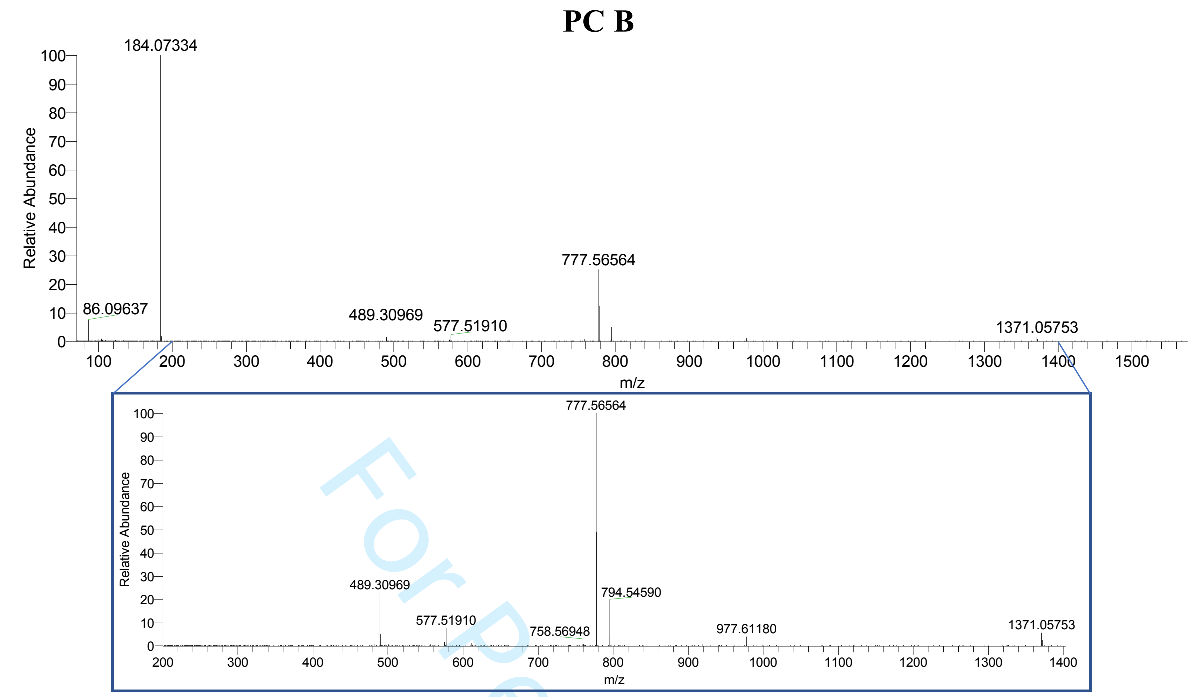

Fragment	Predicted formula	Measured *m/z*	Theoretical *m/z*	Mass difference [ppm]
PC B-1	$C_{79}H_{150}O_{12}NClP^{+}$	1371.05753	1371.05787	-0.24798
PC B-2	$C_{47}H_{96}O_{12}N_2ClP_2^{+}$	977.61180	977.61215	-0.04077
PC B-3	$C_{44}H_{87}O_{12}ClP_2^{+}$	918.53851	918.53865	0.18061
PC B-4	$C_{42}H_{82}O_8NClP^{+}$	794.54590	794.54611	-0.26576
PC B-5	$C_{84}H_{165}O_{16}N_2ClP_2^{2+}$	777.56564	777.56560	0.05144
PC B-6	$C_{42}H_{83}O_8NP^{+}$	760.58502	760.58508	-0.08727
PC B-7-1<br>PC B-7-2	$C_{42}H_{81}O_8NP^{+}$<br>$C_{84}H_{162}O_{16}N_2P_2^{2+}$	758.56948	758.56943	0.06373
PC B-8	$C_{37}H_{68}O_4Cl^{+}$	611.48002	611.48006	-0.07137
PC B-9	$C_{37}H_{69}O_4^{+}$	577.51910	577.51904	0.10831
PC B-10	$C_{37}H_{67}O_4^{+}$	575.50348	575.50339	0.15869
PC B-11	$C_{26}H_{52}O_7NClP^{+}$	556.31635	556.31644	-0.17562
PC B-12	$C_{26}H_{50}O_6NClP^{+}$	538.30762	538.30588	-0.26764
PC B-13	$C_{26}H_{51}O_7NP^{+}$	520.33991	520.33977	0.28252
PC B-14	$C_{23}H_{48}O_6NClP^{+}$	500.29016	500.29023	-0.13729
PC B-15	$C_{47}H_{97}O_{12}N_2ClP_2^{2+}$	489.30969	489.30972	-0.05687
PC B-16	$C_{21}H_{40}O_6P^{+}$	419.25544	419.25570	-0.62024
PC B-17-1<br>PC B-17-2	$C_{21}H_{38}O_3Cl^{+}$<br>$C_{20}H_{38}O_4P^{+}$	373.25029	373.25040<br>373.25022	-0.29448<br>0.17866
PC B-18	$C_{21}H_{37}O_3^{+}$	337.27358	337.27372	-0.43369
PC B-19	$C_{19}H_{37}O_3^{+}$	313.27372	313.27372	0.00543

For Peer Review

PC B-20	$C_{18}H_{35}O_2^+$	283.26336	283.26316	0.70832
PC B-21	$C_{18}H_{31}O^+$	263.23691	263.23694	-0.12802
PC B-22	$C_{16}H_{31}O^+$	239.23680	239.23694	-0.58702
PC B-23	$C_6H_{17}O_4NP^+$	198.08898	198.08897	0.04253
PC B-24	$C_5H_{15}O_4NP^+$	184.07334	184.07332	0.08713
PC B-25	$C_2H_6O_4P^+$	124.99979	124.99982	-0.26414
PC B-26	$C_5H_{14}ON^+$	104.10690	104.10699	-0.84018
PC B-27	$H_4O_4P^+$	98.98408	98.98417	-0.91466
PC B-28	$C_5H_{12}N^+$	86.09637	86.09643	-0.70404

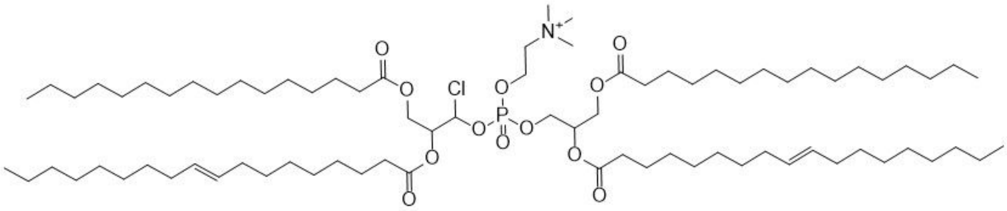

PC B-1

m/*z* 1371.05787

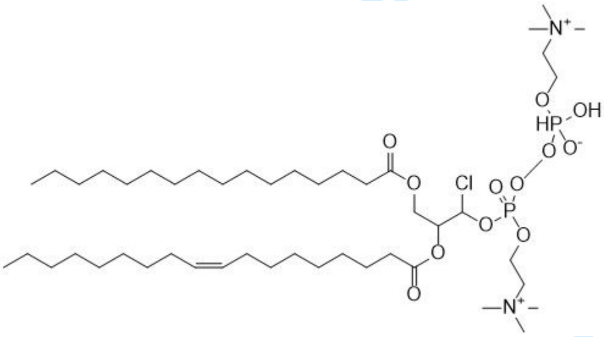

PC B-2

m/*z* 977.61215

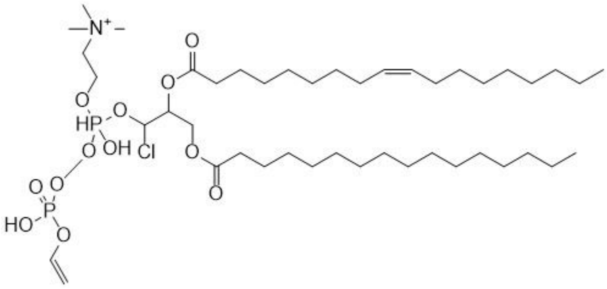

PC B-3

m/*z* 918.53865

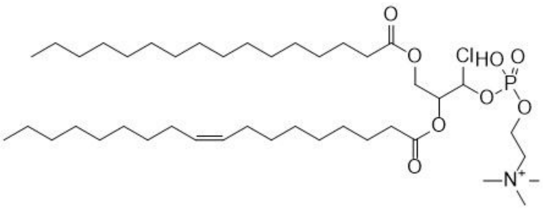

PC B-4

m/z 794.54611

PC B-5

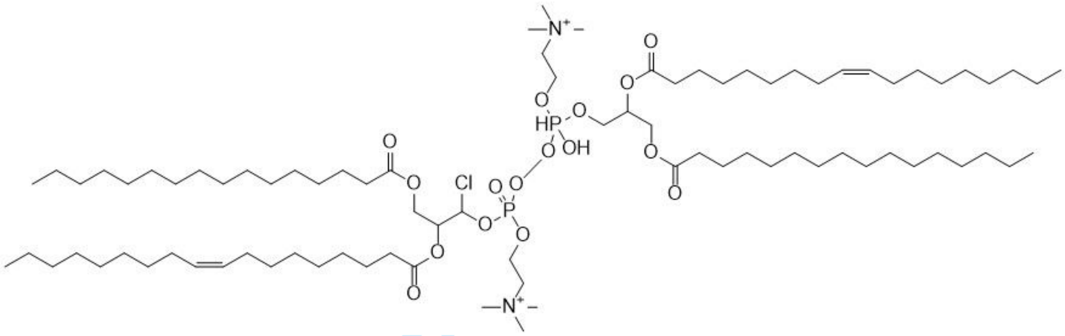

m/z 777.56560

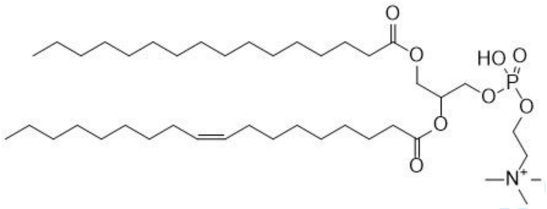

PC B-6

m/z 760.58508

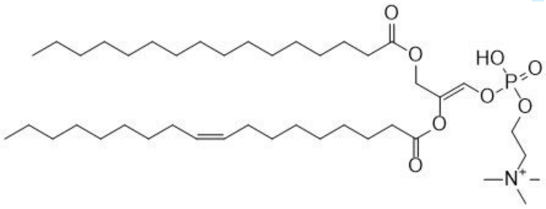

PC B-7-1

m/z 758.56943

PC B-7-2

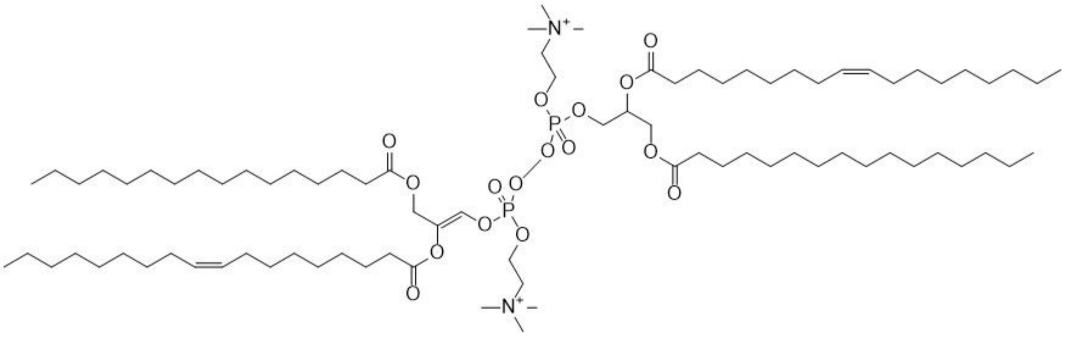

m/z 758.56943

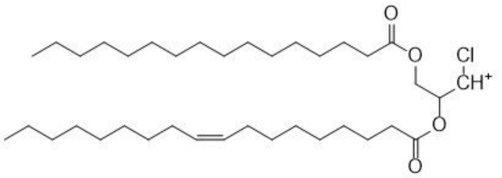

PC B-8

m/*z* 611.48006

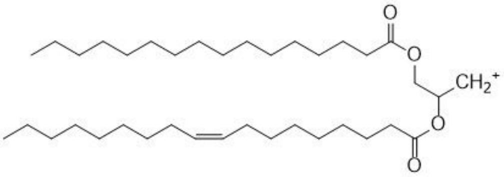

PC B-9

m/*z* 577.51904

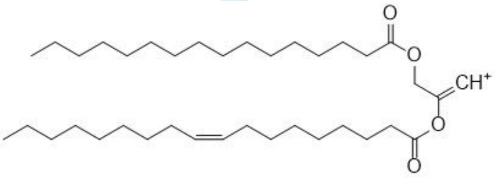

PC B-10

m/*z* 575.50339

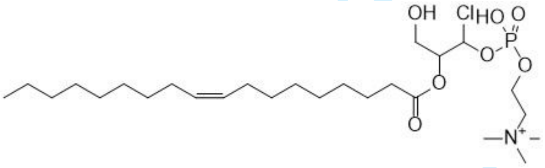

PC B-11

m/*z* 556.31644

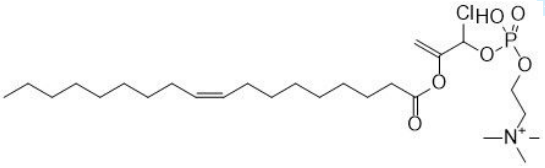

PC B-12

m/*z* 538.30588

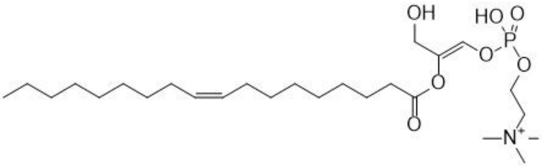

PC B-13

m/*z* 520.33977

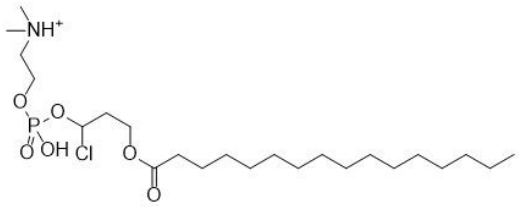

PC B-14

m/z 500.29023

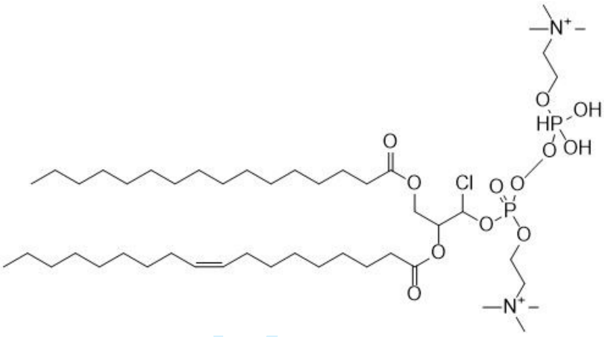

PC B-15

m/z 489.30972

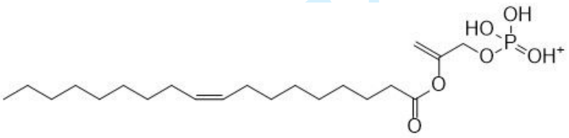

PC B-16

m/z 419.25570

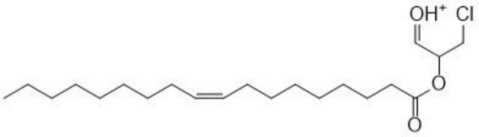

PC B-17-1

m/z 373.25040

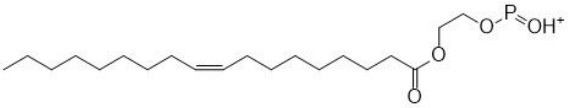

PC B-17-2

m/z 373.25022

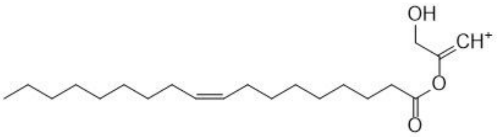

PC B-18

m/z 337.27372

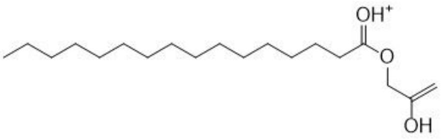

PC B-19

m/z 313.27372

For Peer Review

PC B-20 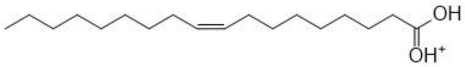

m/z 283.26316

PC B-21 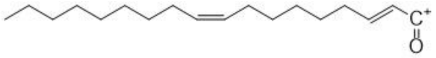

m/z 263.23694

PC B-22 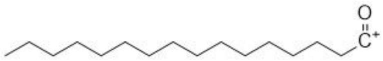

m/z 239.23694

PC B-23 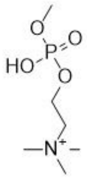

m/z 198.08897

PC B-24 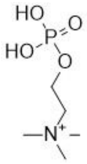

m/z 184.07332

PC B-25 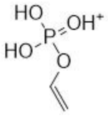

m/z 124.99982

PC B-26 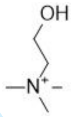

m/z 104.10699

PC B-27 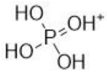

m/z 98.98417

PC B-28 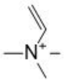

m/z 86.09643

Fig. S32 Proposed structures of fragments PC B-1–PC B-28

For Peer Review

3.1.4 Fragmentation of PC C

Table S6 Fragments of PC C (chlorinated POPC)

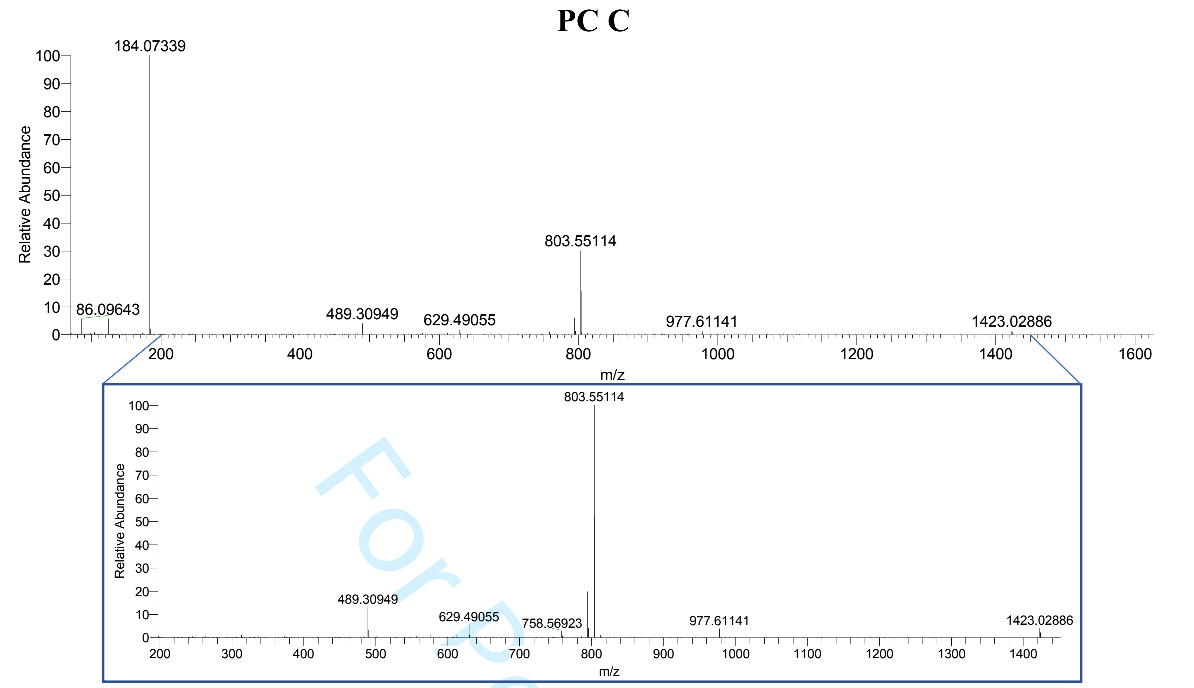

Fragment	Predicted formula	Measured *m/z*	Theoretical *m/z*	Mass difference [ppm]
PC C-1	$C_{79}H_{151}O_{13}NCl_2P^+$	1423.02886	1423.02946	-0.42163
PC C-2	$C_{47}H_{96}O_{12}N_2ClP_2^+$	977.61141	977.61215	-0.44037
PC C-3	$C_{44}H_{87}O_{12}ClP_2^+$	918.53842	918.53865	0.08025
PC C-4	$C_{42}H_{84}O_9NClP^+$	812.55632	812.55667	-0.43604
PC C-5	$C_{84}H_{166}O_{17}N_2Cl_2P_2^{2+}$	803.55114	803.55139	-0.31365
PC C-6	$C_{42}H_{82}O_8NClP^+$	794.54607	794.54611	-0.04594
PC C-7	$C_{42}H_{81}O_8NP^+$	758.56923	758.56943	-0.26297
PC C-8-1<br>PC C-8-2	$C_{37}H_{70}O_5Cl^+$	629.49055	629.49063	-0.13061
PC C-9	$C_{37}H_{68}O_4Cl^+$	611.48001	611.48006	-0.09200
PC C-10	$C_{37}H_{67}O_4^+$	575.50322	575.50339	-0.28249
PC C-11-1<br>PC C-11-2	$C_{26}H_{52}O_7NClP^+$	556.31618	556.31644	-0.47357
PC C-12	$C_{26}H_{51}O_7NP^+$	520.33882	520.33977	-1.81340
PC C-13	$C_{47}H_{97}O_{12}N_2ClP_2^{2+}$	489.30949	489.30972	-0.45999
PC C-14	$C_{21}H_{40}O_6P^+$	419.25552	419.25570	-0.42508
PC C-15-1<br>PC C-15-2	$C_{21}H_{38}O_3Cl^+$<br>$C_{20}H_{38}O_4P^+$	373.25013	373.25040<br>373.25022	-0.71068<br>-0.23754
PC C-16	$C_{21}H_{37}O_3^+$	337.27376	337.27372	0.10007
PC C-17	$C_{19}H_{37}O_3^+$	313.27374	313.27372	0.06135
PC C-18	$C_{18}H_{31}O^+$	263.23697	263.23694	0.11667

For Peer Review

PC C-19	$C_{16}H_{31}O^{+}$	239.23690	239.23694	-0.16491
PC C-20	$C_5H_{14}O_3NClP^{+}$	202.03940	202.03943	-0.17733
PC C-21	$C_6H_{17}O_4NP^{+}$	198.08900	198.08897	0.13456
PC C-22	$C_5H_{15}O_4NP^{+}$	184.07339	184.07332	0.36886
PC C-23	$C_2H_6O_4P^{+}$	124.99983	124.99982	0.06438
PC C-24	$C_5H_{14}ON^{+}$	104.10700	104.10699	0.10378
PC C-25	$H_4O_4P^{+}$	98.98419	98.98417	0.16739
PC C-26	$C_5H_{12}N^{+}$	86.09643	86.09643	0.08528

Table S7 Fragments of PC C or its isomer (chlorinated lung). Fragments that were detected in the single-lipid systems but not in the chlorinated lung sample are indicated with a hyphen (-)

PC C or its isomer

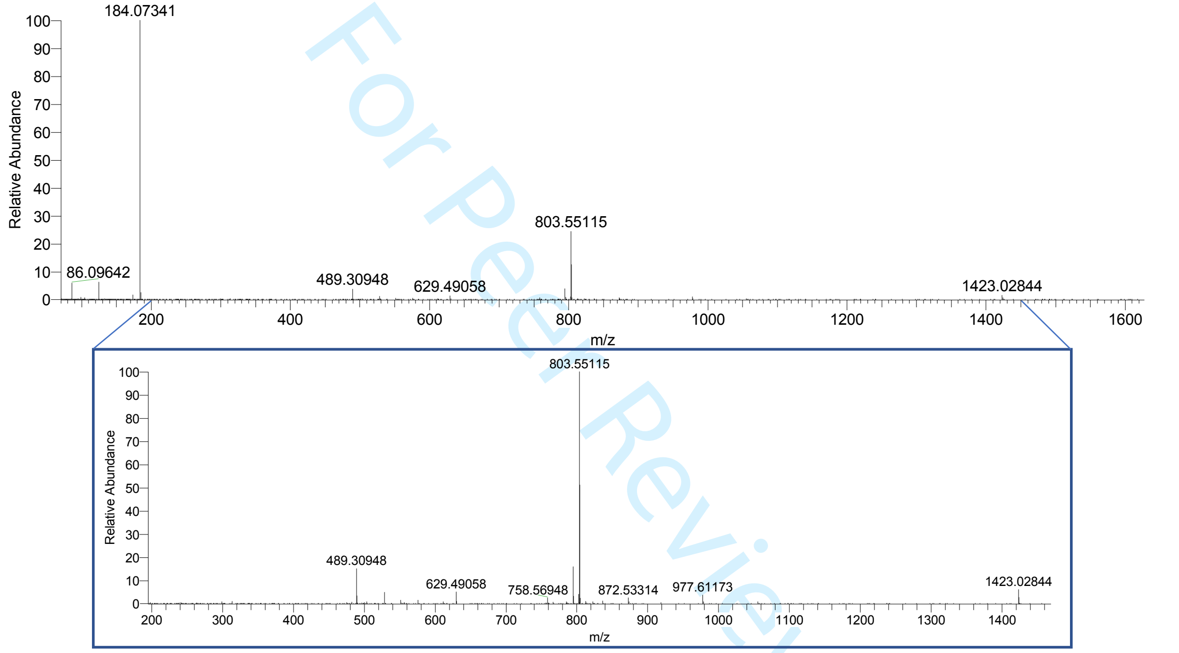

Fragment	Predicted formula	Measured *m/z*	Theoretical *m/z*	Mass difference [ppm]
PC C-1	$C_{79}H_{151}O_{13}NCl_2P^{+}$	1423.02844	1423.02946	-0.71678
PC C-2	$C_{47}H_{96}O_{12}N_2ClP_2^{+}$	977.61173	977.61215	-0.11995
PC C-3	$C_{44}H_{87}O_{12}ClP_2^{+}$	918.53869	918.53865	0.18645
PC C-4	$C_{42}H_{84}O_9NClP^{+}$	812.55646	812.55667	-0.25942
PC C-5	$C_{84}H_{166}O_{17}N_2Cl_2P_2^{2+}$	803.55115	803.55139	-0.30608
PC C-6	$C_{42}H_{82}O_8NClP^{+}$	794.54631	794.54611	0.25879
PC C-7	$C_{42}H_{81}O_8NP^{+}$	758.56948	758.56943	0.06046
PC C-8-1<br>PC C-8-2	$C_{37}H_{70}O_5Cl^{+}$	629.49058	629.49063	-0.08095
PC C-9	$C_{37}H_{68}O_4Cl^{+}$	611.48022	611.48006	0.25657
PC C-10	$C_{37}H_{67}O_4^{+}$	575.50329	575.50339	-0.16356
PC C-11-1<br>PC C-11-2	$C_{26}H_{52}O_7NClP^{+}$	556.31620	556.31644	-0.44276

For Peer Review

PC C-12	$C_{26}H_{51}O_7NP^+$	520.33967	520.33977	-0.17588
PC C-13	$C_{47}H_{97}O_{12}N_2ClP_2^{2+}$	489.30948	489.30972	-0.47710
PC C-14	$C_{21}H_{40}O_6P^+$	-	419.25570	-
PC C-15-1<br>PC C-15-2	$C_{21}H_{38}O_3Cl^+$<br>$C_{20}H_{38}O_4P^+$	373.25001	373.25040<br>373.25022	-1.03290<br>-0.55976
PC C-16	$C_{21}H_{37}O_3^+$	337.27375	337.27372	0.09522
PC C-17	$C_{19}H_{37}O_3^+$	313.27372	313.27372	0.00025
PC C-18	$C_{18}H_{31}O^+$	263.23693	263.23694	-0.05171
PC C-19	$C_{16}H_{31}O^+$	239.23697	239.23694	0.10571
PC C-20	$C_5H_{14}O_3NClP^+$	-	202.03943	-
PC C-21	$C_6H_{17}O_4NP^+$	198.08899	198.08897	0.08681
PC C-22	$C_5H_{15}O_4NP^+$	184.07341	184.07332	0.47658
PC C-23	$C_2H_6O_4P^+$	124.99981	124.99982	-0.06425
PC C-24	$C_5H_{14}ON^+$	104.10699	104.10699	-0.03547
PC C-25	$H_4O_4P^+$	98.98417	98.98417	0.01797
PC C-26	$C_5H_{12}N^+$	86.09642	86.09643	-0.01488

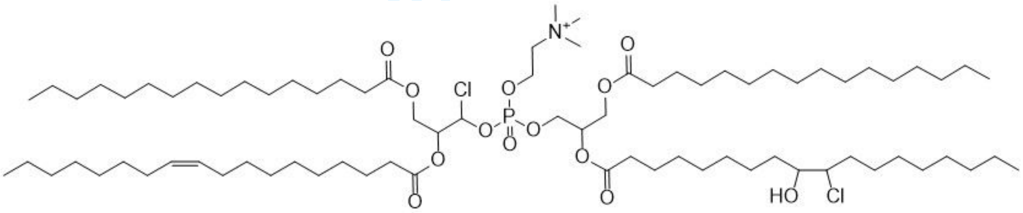

PC C-1

m/z 1423.02946

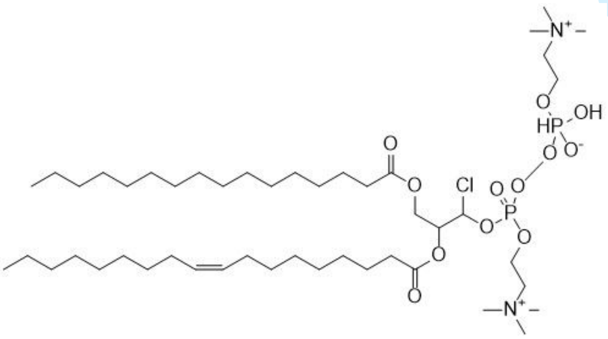

PC C-2

m/z 977.61215

For Peer Review

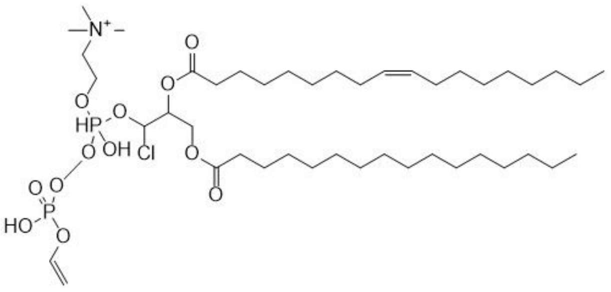

PC C-3

m/*z* 918.53865

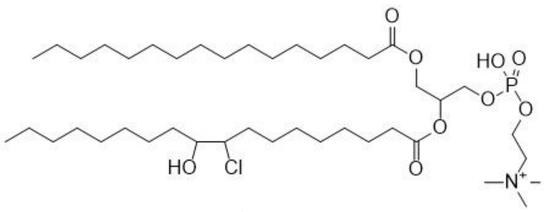

PC C-4

m/*z* 812.55667

PC C-5

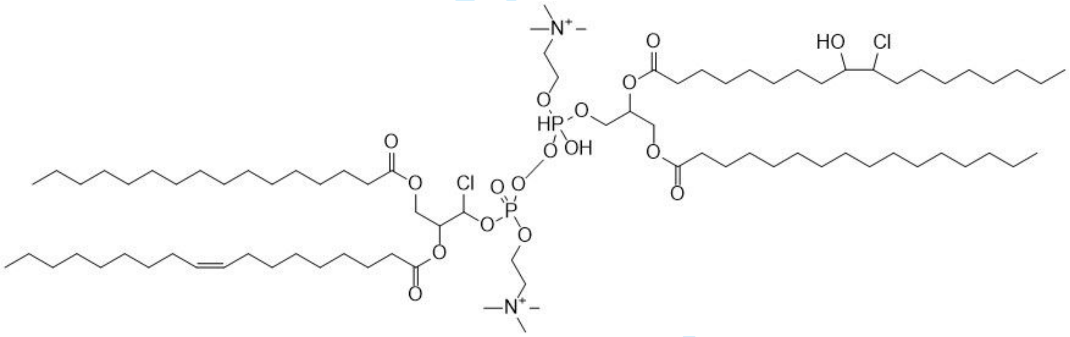

m/*z* 803.55139

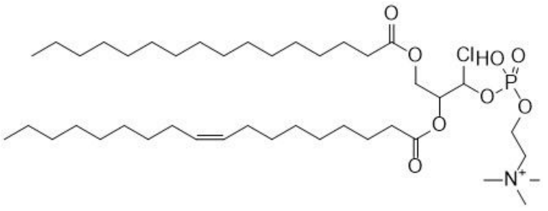

PC C-6

m/*z* 794.54611

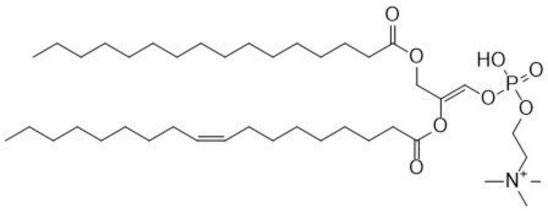

PC C-7

m/*z* 758.56943

**PC C-8-1**<br>*m/z* 629.4963	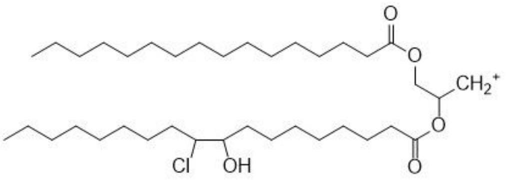
**PC C-8-2**<br>*m/z* 629.49063	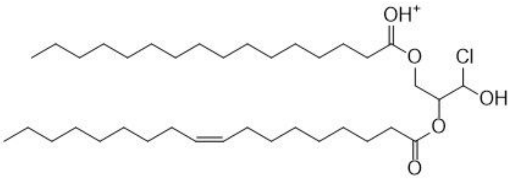
**PC C-9**<br>*m/z* 611.48006	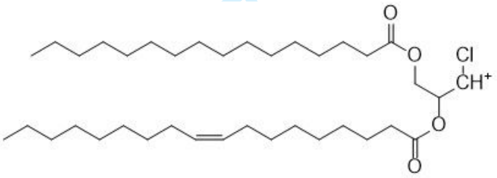
**PC C-10**<br>*m/z* 575.50339	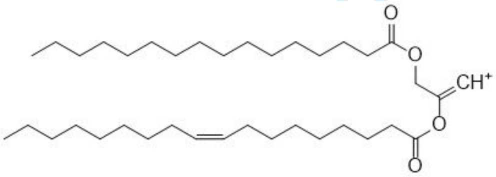
**PC C-11-1**<br>*m/z* 556.31644	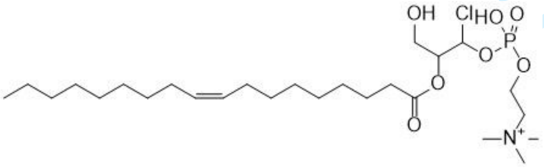
**PC C-11-2**<br>*m/z* 556.31644	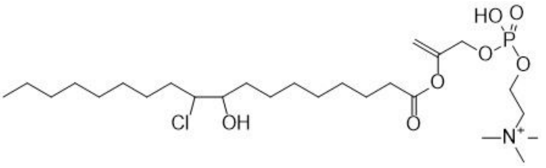
**PC C-12**<br>*m/z* 520.33977	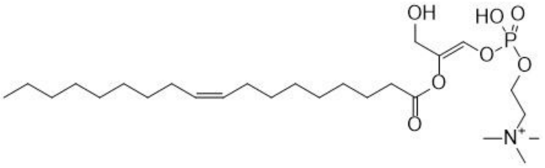

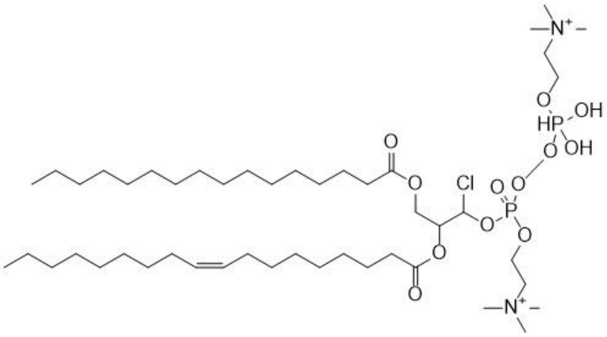

PC C-13

m/z 489.30499

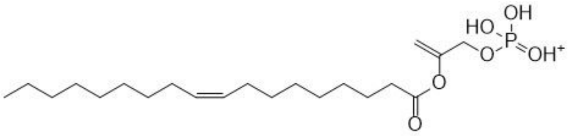

PC C-14

m/z 419.25570

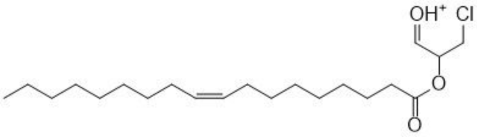

PC C-15-1

m/z 373.25040

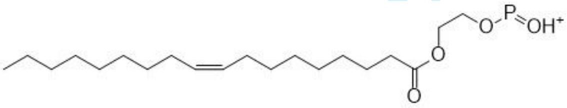

PC C-15-2

m/z 373.25022

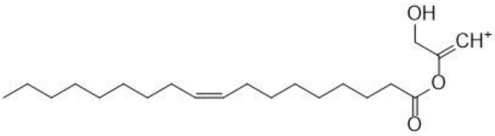

PC C-16

m/z 337.27372

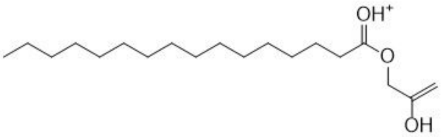

PC C-17

m/z 313.27372

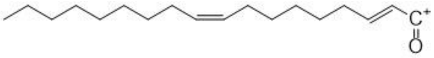

PC C-18

m/z 263.23694

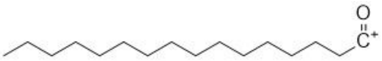

PC C-19

m/z 239.23694

**PC C-20** 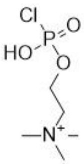 *m/z* 202.03943	**PC C-21** 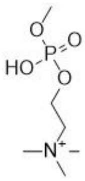 *m/z* 198.08897	**PC C-22** 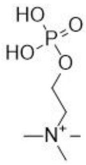 *m/z* 184.07332	**PC C-23** 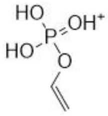 *m/z* 124.99982
**PC C-24** 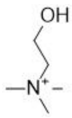 *m/z* 104.10699	**PC C-25** 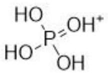 *m/z* 98.98417	**PC C-26** 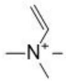 *m/z* 86.09643	

Fig. S33 Proposed structures of fragments PC C-1–PC C-26

For Peer Review

3.1.5 Fragmentation of PC D

Table S8 Fragments of PC D (chlorinated POPC)

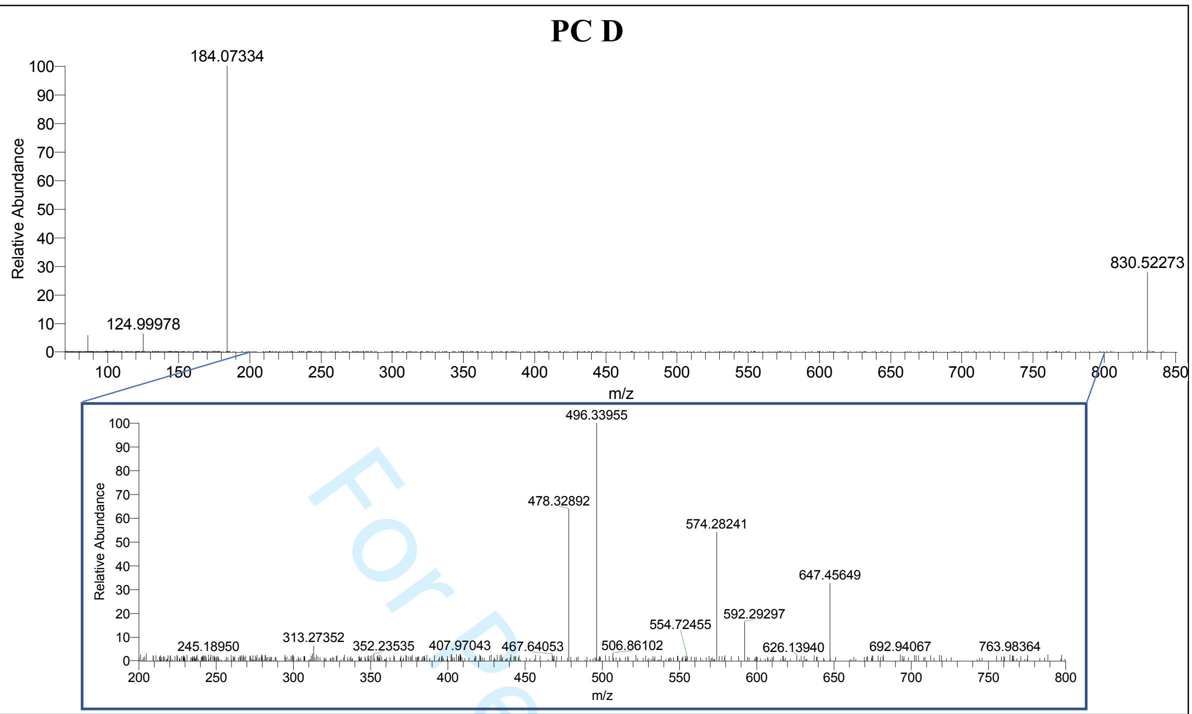

Fragment	Predicted formula	Measured *m/z*	Theoretical *m/z*	Mass difference [ppm]
PC D-1	$C_{42}H_{83}O_8NCl_2P^+$	830.52273	830.52279	-0.06594
PC D-2	$C_{26}H_{53}O_7NCl_2P^+$	592.29297	592.29312	-0.24927
PC D-3	$C_{26}H_{51}O_6NCl_2P^+$	574.28241	574.28256	-0.25783
PC D-4	$C_{24}H_{51}O_7NP^+$	496.33955	496.33977	-0.43811
PC D-5	$C_{24}H_{49}O_6NP^+$	478.32892	478.32920	-0.58323
PC D-6	$C_{19}H_{37}O_3^+$	313.27352	313.27372	-0.63389
PC D-7	$C_5H_{15}O_4NP^+$	184.07334	184.07332	0.10690
PC D-8	$C_2H_6O_4P^+$	124.99978	124.99982	-0.36088
PC D-9	$C_5H_{14}ON^+$	104.10691	104.10699	-0.73140
PC D-10	$H_4O_4P^+$	98.98409	98.98417	-0.84008
PC D-11	$C_5H_{12}N^+$	86.09638	86.09643	-0.55625

For Peer Review

Table S9 Fragments of PC D or its isomer (chlorinated lung). Fragments that were detected in the single-lipid systems but not in the chlorinated lung sample are indicated with a hyphen (-)

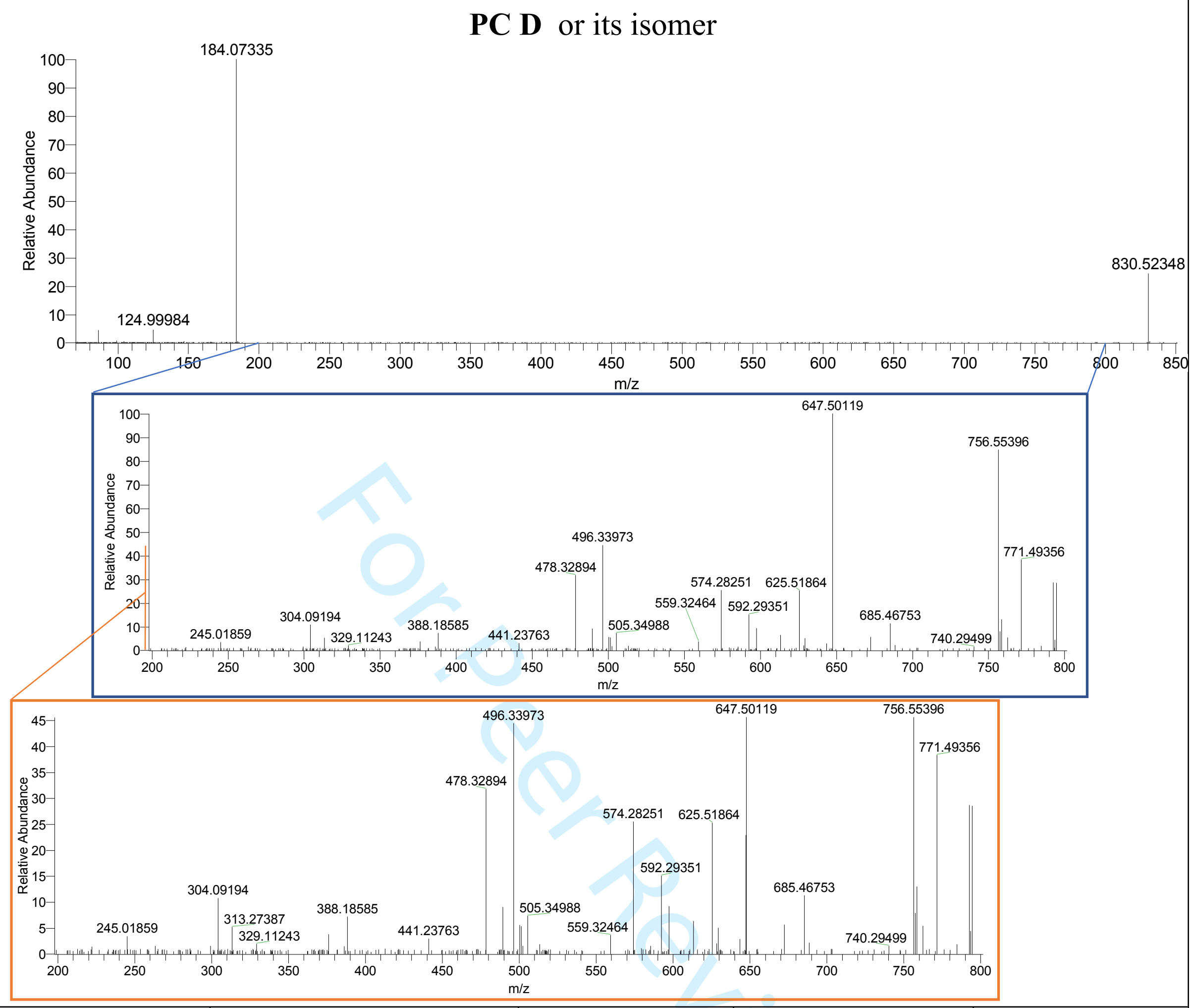

Fragment	Predicted formula	Measured *m/z*	Theoretical *m/z*	Mass difference [ppm]
PC D-1	$C_{42}H_{84}O_8NClP^+$	830.52348	830.52279	0.84046
PC D-2	$C_{26}H_{53}O_7NCl_2P^+$	-	592.29312	-
PC D-3	$C_{26}H_{51}O_6NCl_2P^+$	574.28251	574.28256	-0.07953
PC D-4	$C_{24}H_{51}O_7NP^+$	496.33973	496.33977	-0.07514
PC D-5	$C_{24}H_{49}O_6NP^+$	478.32894	478.32920	-0.53791
PC D-6	$C_{19}H_{37}O_3^+$	313.27387	313.27372	0.48829
PC D-7	$C_5H_{15}O_4NP^+$	184.07335	184.07332	0.13822
PC D-8	$C_2H_6O_4P^+$	124.99984	124.99982	0.11768
PC D-9	$C_5H_{14}ON^+$	104.10698	104.10699	-0.14965
PC D-10	$H_4O_4P^+$	98.98418	98.98417	0.06363
PC D-11	$C_5H_{12}N^+$	86.09643	86.09643	0.10188

For Peer Review

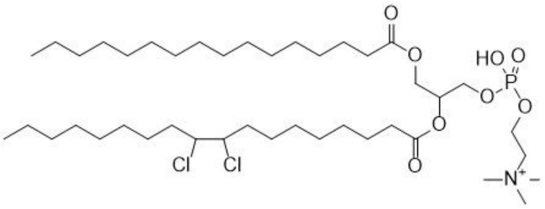

PC D-1

m/z 830.52279

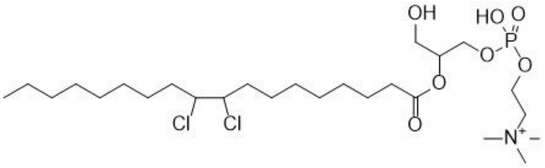

PC D-2

m/z 592.29312

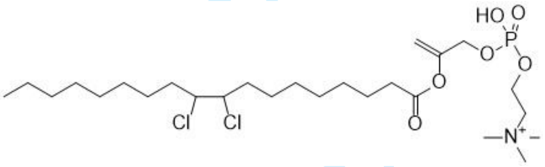

PC D-3

m/z 574.28256

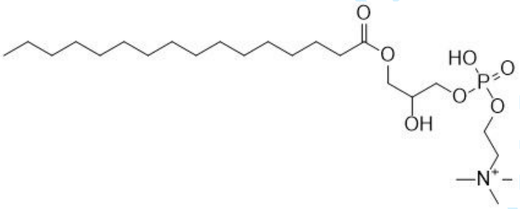

PC D-4

m/z 496.33977

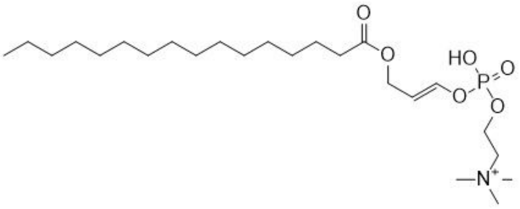

PC D-5

m/z 478.32920

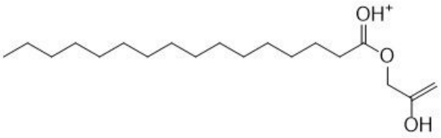

PC D-6

m/z 313.27372

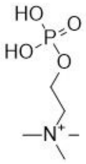

PC D-7

m/z 184.07332

**PC D-8** 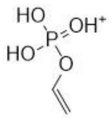	**PC D-9** 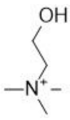	**PC D-10** 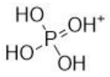	**PC D-11** 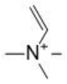
m/z 124.99982	*m/z* 104.10699	*m/z* 98.98417	*m/z* 86.09643

Fig. S34 Proposed structures of fragments PC D-1–PC D-11

For Peer Review

3.1.6 Fragmentation of PC E

Table S10 Fragments of PC E (chlorinated POPC)

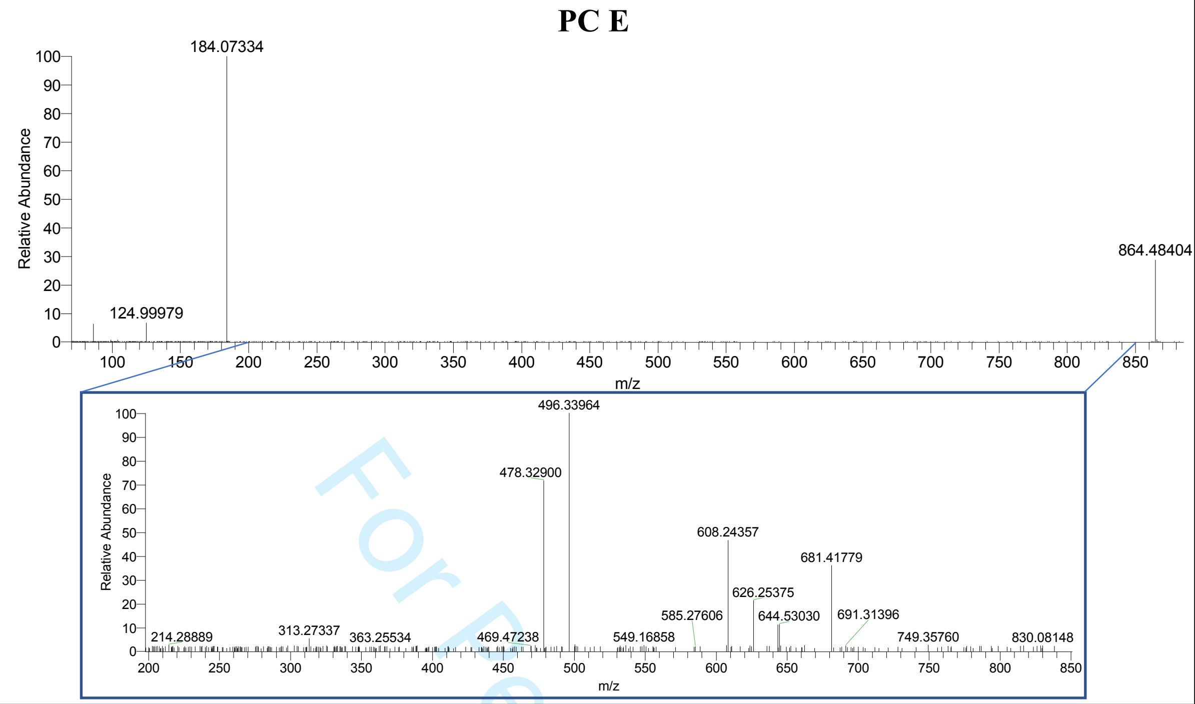

Fragment	Predicted formula	Measured *m/z*	Theoretical *m/z*	Mass difference [ppm]
PC E-1	$C_{42}H_{82}O_8NCl_3P^+$	864.48404	864.48381	0.26547
PC E-2	$C_{42}H_{81}O_8NCl_2P^+$	828.50696	828.50714	-0.21572
PC E-3	$C_{37}H_{68}O_4Cl_3^+$	681.41779	681.41777	0.02808
PC E-4	$C_{26}H_{52}O_7NCl_3P^+$	626.25375	626.25415	-0.64088
PC E-5	$C_{26}H_{50}O_6NCl_3P^+$	608.24357	608.24358	-0.01644
PC E-6	$C_{24}H_{51}O_7NP^+$	496.33964	496.33977	-0.26063
PC E-7	$C_{24}H_{49}O_6NP^+$	478.32900	478.32920	-0.42770
PC E-8	$C_{19}H_{37}O_3^+$	313.27337	313.27372	-1.13598
PC E-9	$C_{18}H_{31}O$+	263.23669	263.23694	-0.94134
PC E-10	$C_5H_{15}O_4NP^+$	184.07334	184.07332	0.10900
PC E-11	$C_2H_6O_4P^+$	124.99979	124.99982	-0.23917
PC E-12	$C_5H_{14}ON^+$	104.10691	104.10699	-0.73464
PC E-13	$H_4O_4P^+$	98.98408	98.98417	-0.94621
PC E-14	$C_5H_{12}N^+$	86.09639	86.09643	-0.40740

For Peer Review

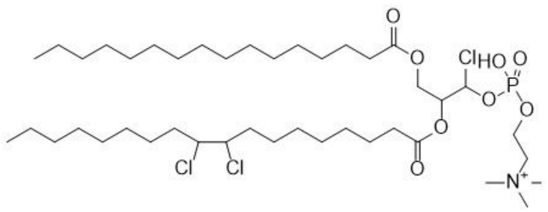

PC E-1

m/z 864.48381

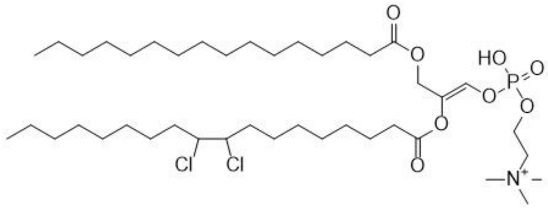

PC E-2

m/z 828.50714

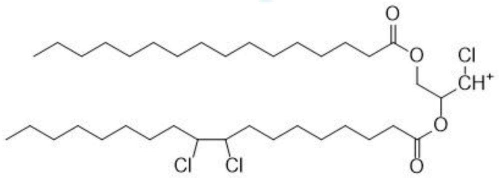

PC E-3

m/z 681.41777

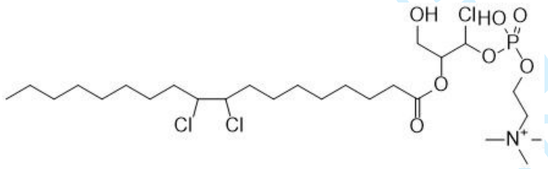

PC E-4

m/z 626.25415

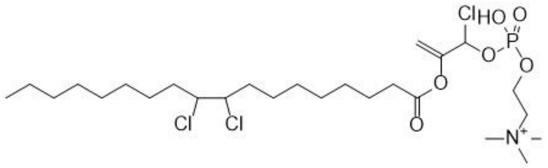

PC E-5

m/z 608.24358

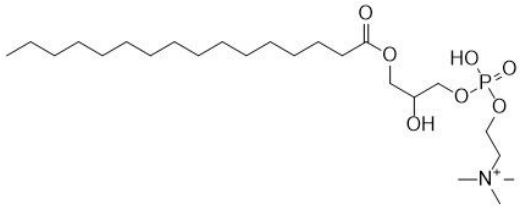

PC E-6

m/z 496.33977

For Review

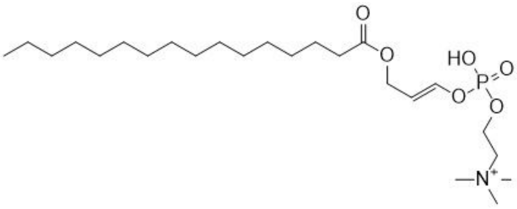

PC E-7

m/z 478.32920

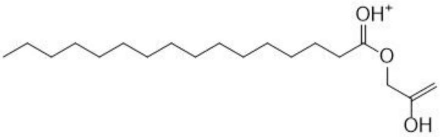

PC E-8

m/z 313.27372

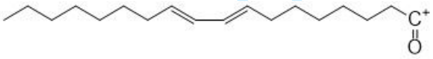

PC E-9

m/z 263.23694

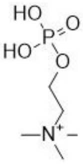

PC E-10

m/z 184.07332

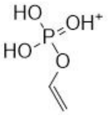

PC E-11

m/z 124.99982

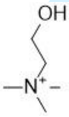

PC E-12

m/z 104.10699

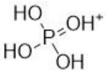

PC E-13

m/z 98.98417

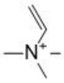

PC E-14

m/z 86.09643

Fig. S35 Proposed structures of fragments PC E-1–PC E-14

For Peer Review

3.1.7 Fragmentation of PC F

Table S11 Fragments of PC F (chlorinated POPC)

PC F

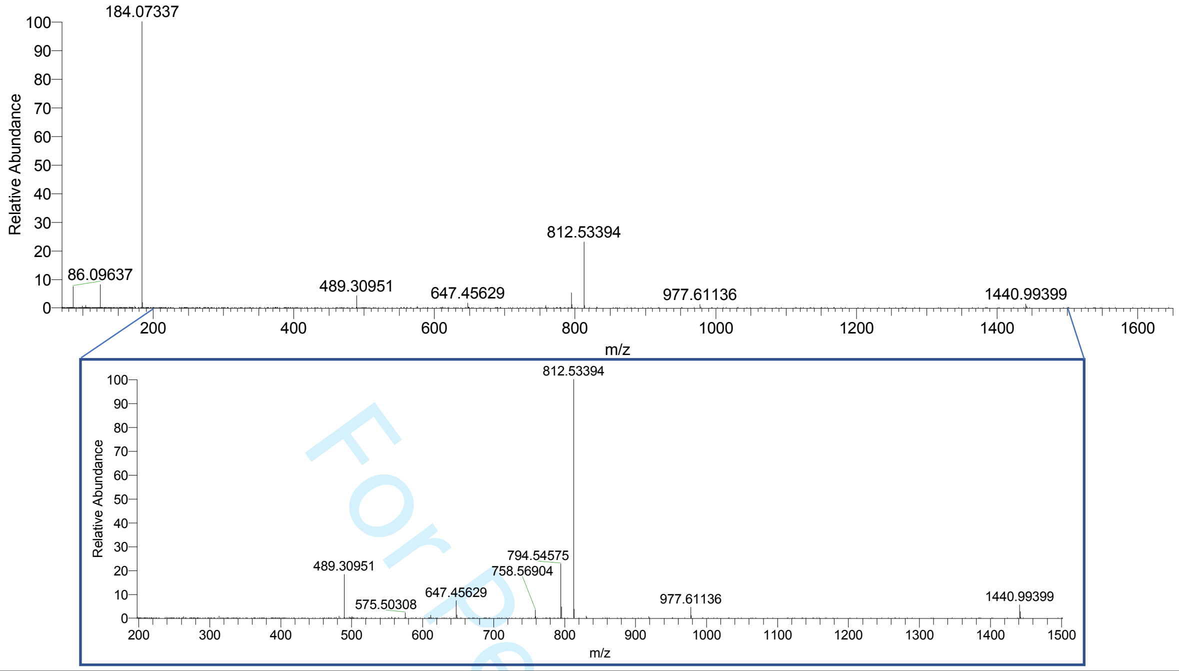

Fragment	Predicted formula	Measured *m/z*	Theoretical *m/z*	Mass difference [ppm]
PC F-1	$C_{79}H_{150}O_{12}NCl_3P^+$	1440.99399	1440.99558	-1.10340
PC F-2	$C_{47}H_{96}O_{12}N_2ClP_2^+$	977.61136	977.61215	-0.81186
PC F-3	$C_{44}H_{87}O_{12}NClP_2^+$	918.53778	918.53865	-0.94742
PC F-4	$C_{84}H_{165}O_{16}N_2Cl_3P_2^{2+}$	812.53394	812.53445	-0.62783
PC F-5-1<br>PC F-5-2	$C_{42}H_{82}O_8NClP^+$<br>$C_{84}H_{164}O_{16}N_2Cl_2P_2^{2+}$	794.54575	794.54611	-0.45673
PC F-6	$C_{42}H_{81}O_8NP^+$	758.56904	758.56943	-0.51122
PC F-7	$C_{37}H_{69}O_4Cl_2^+$	647.45629	647.45674	-0.69187
PC F-8	$C_{37}H_{68}O_4Cl^+$	611.47971	611.48006	-0.58099
PC F-9	$C_{37}H_{67}O_4^+$	575.50308	575.50339	-0.53909
PC F-10	$C_{26}H_{51}Cl_2NO_6P^+$	574.28229	574.28256	-0.46847
PC F-11	$C_{26}H_{52}O_7NClP^+$	556.31606	556.31644	-0.68471
PC F-12	$C_{26}H_{51}O_7NP^+$	520.33870	520.33977	-2.05233
PC F-13	$C_{23}H_{48}O_6NClP^+$	500.29005	500.29023	-0.35304
PC F-14	$C_{24}H_{51}O_7NP^+$	496.33966	496.33977	-0.20974
PC F-15	$C_{47}H_{97}O_{12}N_2ClP_2^{2+}$	489.30951	489.30972	-0.42582
PC F-16	$C_{23}H_{46}O_5NClP^+$	482.27939	482.27966	-0.56084
PC F-17	$C_{21}H_{40}O_6P^+$	419.25556	419.25570	-0.34476
PC F-18-1<br>PC F-18-2	$C_{21}H_{38}O_3Cl^+$<br>$C_{20}H_{38}O_4P^+$	373.25026	373.25040<br>373.25022	-0.36430<br>0.10884

For Peer Review

PC F-19	$C_{21}H_{37}O_3^+$	337.27352	337.27372	-0.59654
PC F-20	$C_{19}H_{37}O_3^+$	313.27352	313.27372	-0.62733
PC F-21	$C_{18}H_{31}O^+$	263.23685	263.23694	-0.33275
PC F-22	$C_{16}H_{31}O^+$	239.23681	239.23694	-0.54929
PC F-23	$C_6H_{17}O_4NP^+$	198.08900	198.08897	0.16862
PC F-24	$C_5H_{15}O_4NP^+$	184.07337	184.07332	0.29297
PC F-25	$C_2H_6O_4P^+$	124.99977	124.99982	-0.40240
PC F-26	$C_5H_{14}ON^+$	104.10690	104.10699	-0.82708
PC F-27	$H_4O_4P^+$	98.98409	98.98417	-0.80614
PC F-28	$C_5H_{12}N^+$	86.09637	86.09643	-0.60028

Table S12 Fragments of PC F or its isomer (chlorinated lung). Fragments that were detected in the single-lipid systems but not in the chlorinated lung sample are indicated with a hyphen (-)

For Peer Review

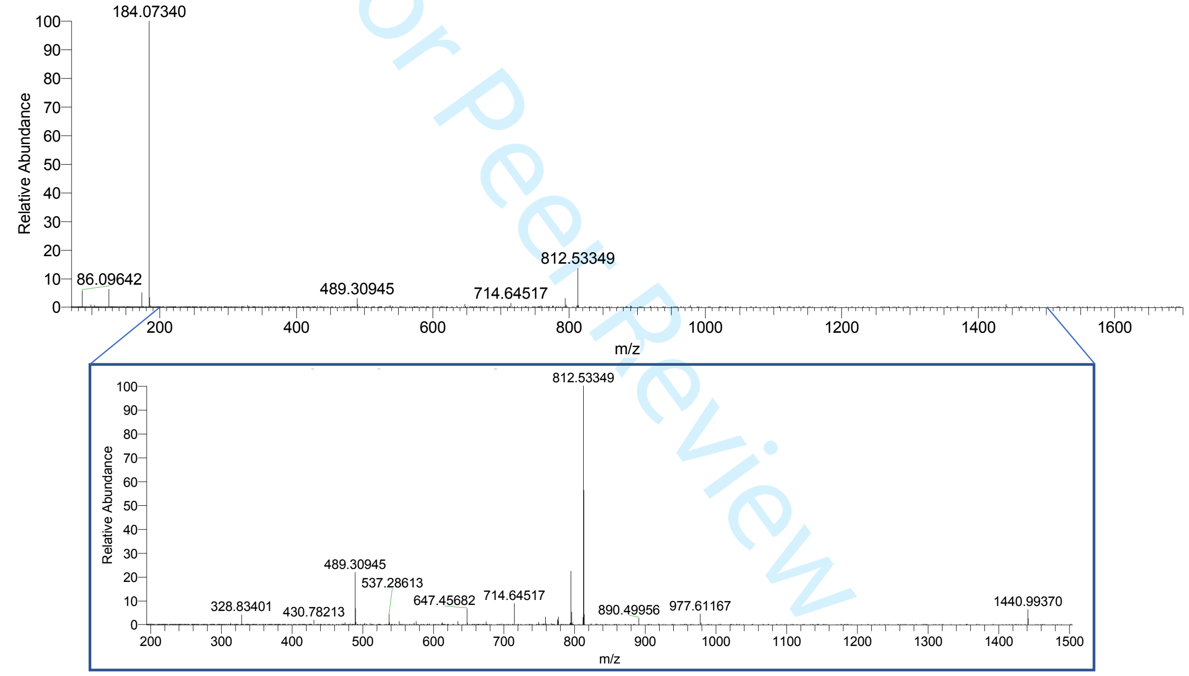

Fragment	Predicted formula	Measured *m/z*	Theoretical *m/z*	Mass difference [ppm]
PC F-1	$C_{79}H_{150}O_{12}NCl_3P^+$	1440.99370	1440.99558	-1.30465
PC F-2	$C_{47}H_{96}O_{12}N_2ClP_2^+$	977.61167	977.61215	-0.49278
PC F-3	$C_{44}H_{87}O_{12}NClP_2^+$	918.53751	918.53865	-1.24826
PC F-4	$C_{84}H_{165}O_{16}N_2Cl_3P_2^{2+}$	812.53349	812.53445	-1.17710
PC F-5-1<br>PC F-5-2	$C_{42}H_{82}O_8NClP^+$<br>$C_{84}H_{164}O_{16}N_2Cl_2P_2^{2+}$	794.54583	794.54611	-0.35210
PC F-6	$C_{42}H_{81}O_8NP^+$	758.56945	758.56943	0.02064
PC F-7	$C_{37}H_{69}O_4Cl_2^+$	647.45682	647.45674	0.11723
PC F-8	$C_{37}H_{68}O_4Cl^+$	611.48002	611.48006	-0.07815

PC F-9	$C_{37}H_{67}O_4^+$	575.50331	575.50339	-0.13418
PC F-10	$C_{26}H_{51}Cl_2NO_6P^+$	-	574.28256	-
PC F-11	$C_{26}H_{52}O_7NClP^+$	556.31580	556.31644	-1.16420
PC F-12	$C_{26}H_{51}O_7NP^+$	520.33942	520.33977	-0.67134
PC F-13	$C_{23}H_{48}O_6NClP^+$	500.29089	500.29023	1.32866
PC F-14	$C_{24}H_{51}O_7NP^+$	-	496.33977	-
PC F-15	$C_{47}H_{97}O_{12}N_2ClP_2^{2+}$	489.30945	489.30972	-0.54910
PC F-16	$C_{23}H_{46}O_5NClP^+$	482.27940	482.27966	0.45857
PC F-17	$C_{21}H_{40}O_6P^+$	-	419.25570	-
PC F-18-1<br>PC F-18-2	$C_{21}H_{38}O_3Cl^+$<br>$C_{20}H_{38}O_4P^+$	-	373.25040<br>373.25022	-
PC F-19	$C_{21}H_{37}O_3^+$	-	337.27372	-
PC F-20	$C_{19}H_{37}O_3^+$	313.27368	313.27372	-0.12720
PC F-21	$C_{18}H_{31}O^+$	263.23697	263.23694	0.10205
PC F-22	$C_{16}H_{31}O^+$	-	239.23694	-
PC F-23	$C_6H_{17}O_4NP^+$	198.08905	198.08897	0.39421
PC F-24	$C_5H_{15}O_4NP^+$	184.07340	184.07332	0.41983
PC F-25	$C_2H_6O_4P^+$	124.99981	124.99982	-0.09862
PC F-26	$C_5H_{14}ON^+$	104.10698	104.10699	-0.12297
PC F-27	$H_4O_4P^+$	98.98419	98.98417	0.15099
PC F-28	$C_5H_{12}N^+$	86.09642	86.09643	-0.04070

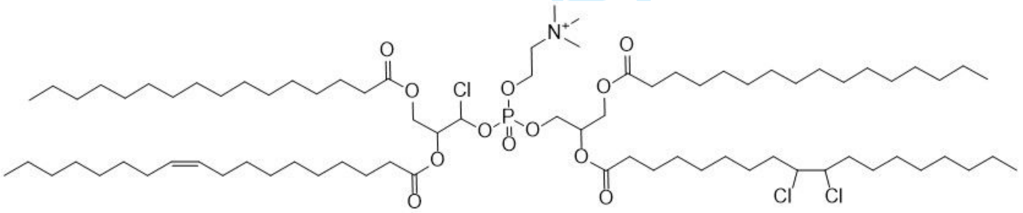

PC F-1

m/z 1440.99558

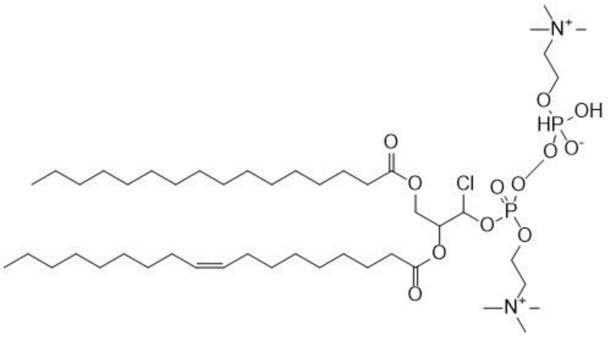

PC F-2

m/z 977.61215

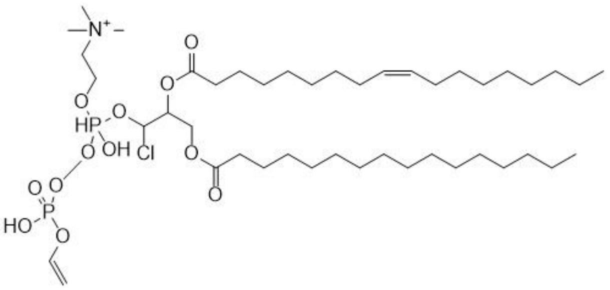

PC F-3

m/z 918.53865

PC F-4

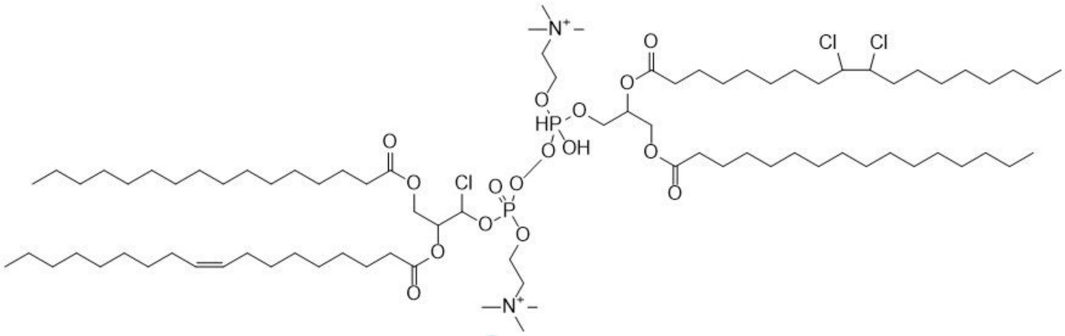

m/z 812.53445

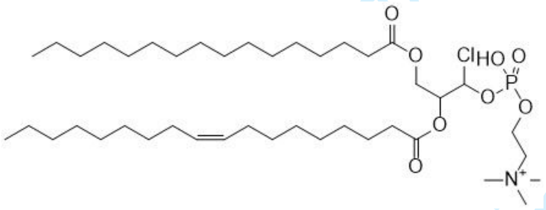

PC F-5-1

m/z 794.54611

PC F-5-2

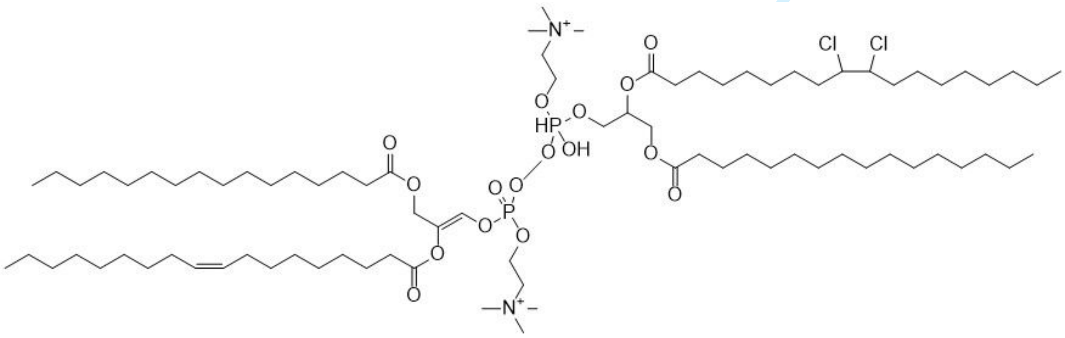

m/z 794.54611

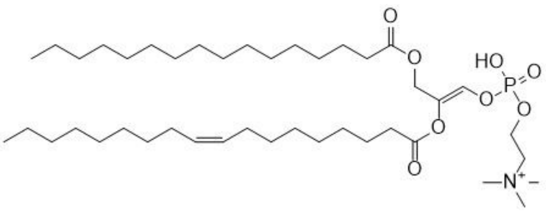

PC F-6

m/z 758.56943

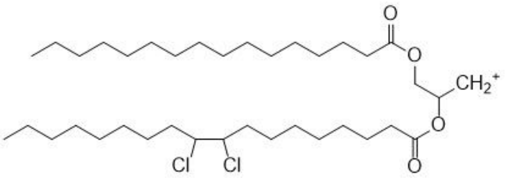

PC F-7

m/z 647.45674

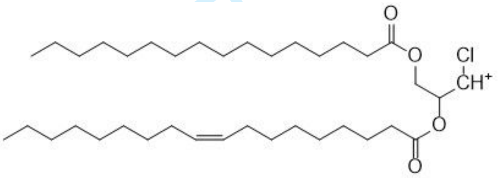

PC F-8

m/z 611.48006

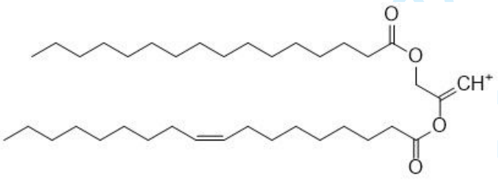

PC F-9

m/z 575.50339

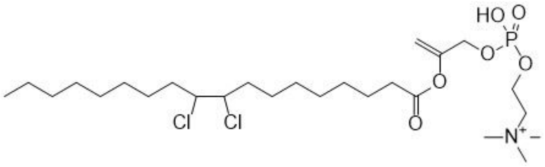

PC F-10

m/z 574.28256

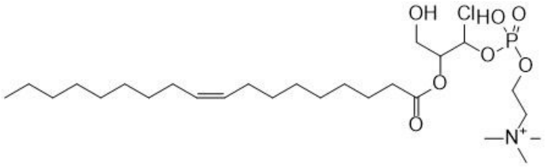

PC F-11

m/z 556.31644

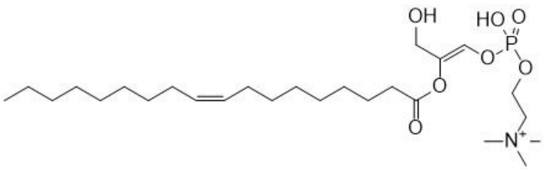

PC F-12

m/z 520.33977

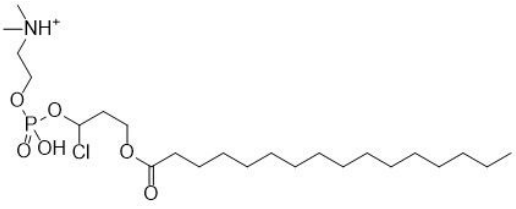

PC F-13

m/z 500.29023

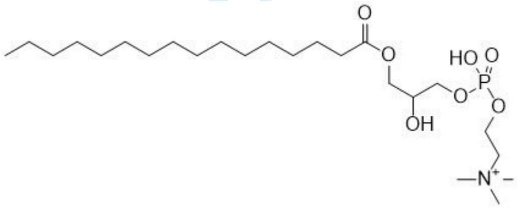

PC F-14

m/z 496.33977

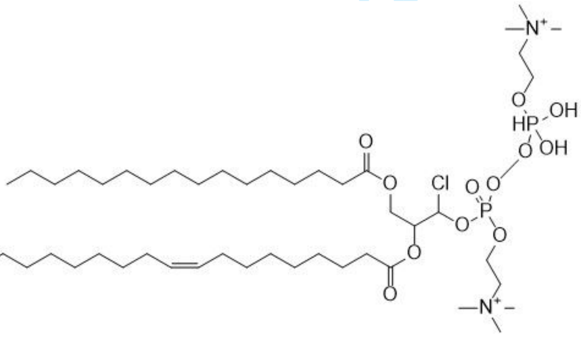

PC F-15

m/z 489.30972

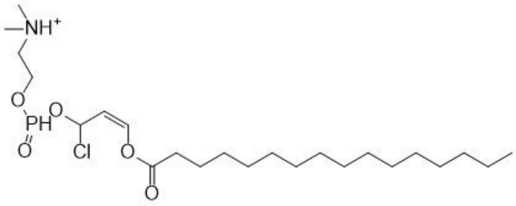

PC F-16

m/z 482.27966

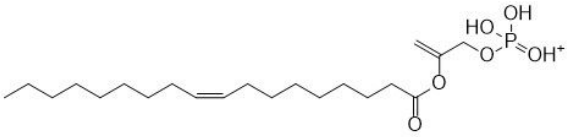

PC F-17

m/z 419.25570

PC F-18-1
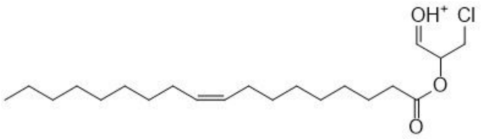
m/z 373.25040

PC F-18-2
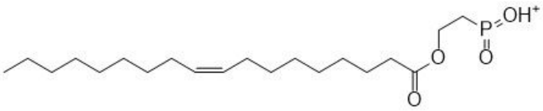
m/z 373.25022

PC F-19
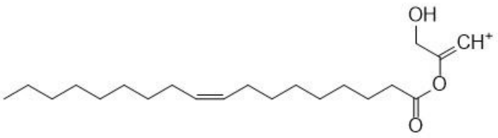
m/z 337.27372

PC F-20
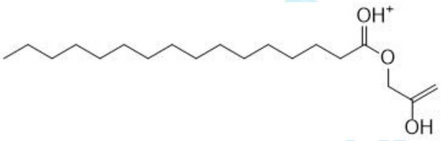
m/z 313.27372

PC F-21
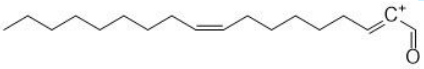
m/z 263.23694

PC F-22
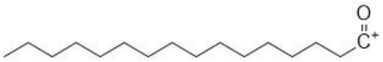
m/z 239.23694

PC F-23
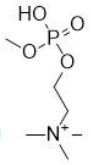
m/z 198.08897

PC F-24
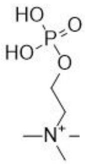
m/z 184.07332

PC F-25
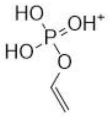
m/z 124.99982

PC F-26
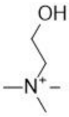
m/z 104.10699

PC F-27
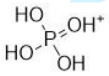
m/z 98.98417

PC F-28
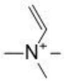
m/z 86.09643

Fig. S36 Proposed structures of fragments PC F-1–PC F-28

For Peer Review

3.1.8 Fragmentation of PC G

Table S13 Fragments of PC G (chlorinated POPC)

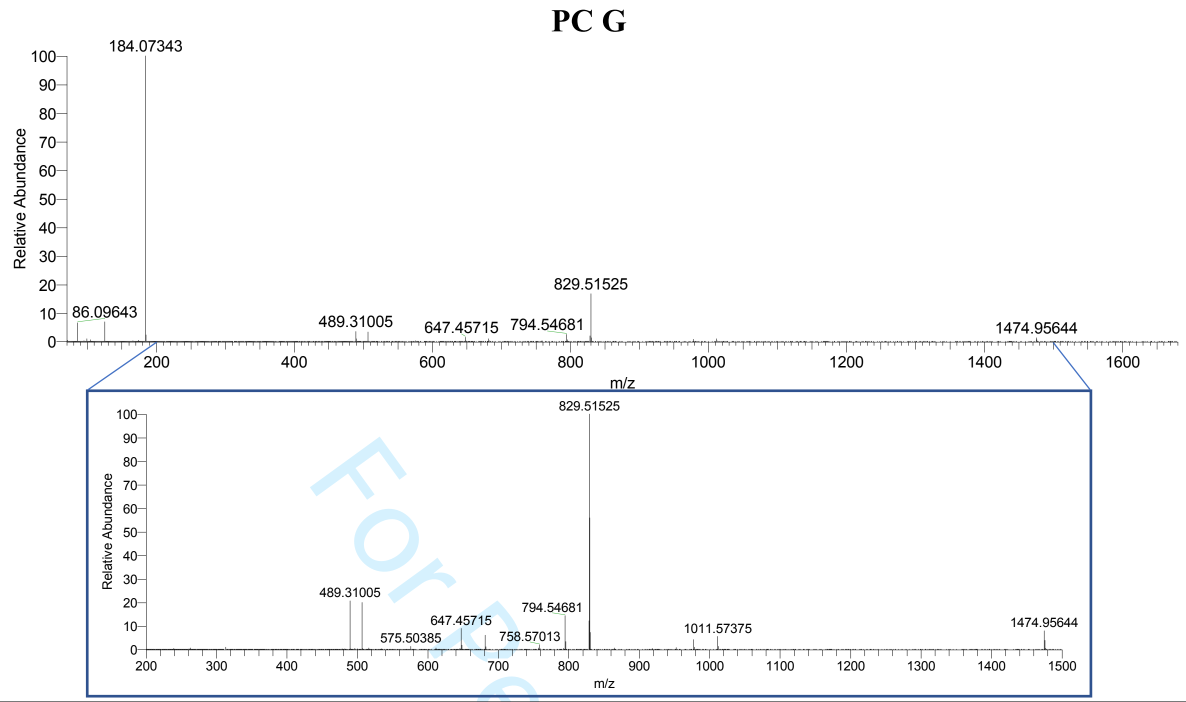

Fragment	Predicted formula	Measured *m/z*	Theoretical *m/z*	Mass difference [ppm]
PC G-1	$C_{79}H_{149}O_{12}NCl_4P^+$	1474.95644	1474.95660	0.00971
PC G-2	$C_{47}H_{95}O_{12}N_2Cl_2P_2^+$	1011.57375	1011.57318	0.56470
PC G-3	$C_{47}H_{96}O_{12}N_2Cl_2P_2^+$	977.61266	977.61215	0.52278
PC G-4	$C_{44}H_{86}O_{12}NCl_2P_2^{2+}$	952.50025	952.49968	0.60003
PC G-5-1<br>PC G-5-2	$C_{44}H_{87}O_{12}NClP_2^+$	918.53923	918.53865	0.63109
PC G-6	$C_{42}H_{82}O_8NCl_3P^+$	864.48483	864.48381	1.17815
PC G-7	$C_{84}H_{164}O_{16}N_2Cl_4P_2^{2+}$	829.51525	829.51496	0.34416
PC G-8-1<br>PC G-8-2	$C_{42}H_{82}O_8NClP^+$	794.54681	794.54611	0.88539
PC G-9	$C_{42}H_{81}O_8NP^+$	758.57013	758.56943	0.91663
PC G-10	$C_{37}H_{68}O_4Cl_3^+$	681.41829	681.41777	0.76201
PC G-11	$C_{37}H_{69}O_4Cl_2^+$	647.45715	647.45674	0.63656
PC G-12-1<br>PC G-12-2	$C_{37}H_{68}O_4Cl^+$	611.48048	611.48006	0.67150
PC G-13	$C_{26}H_{51}O_7NCl_2P^+$	590.27808	590.27747	1.02900
PC G-14-1<br>PC G-14-2	$C_{37}H_{67}O_4^+$	575.50385	575.50339	0.80746
PC G-15	$C_{47}H_{96}O_{12}N_2Cl_2P_2^{2+}$	506.29057	506.29023	0.66401
PC G-16-1<br>PC G-16-2	$C_{47}H_{97}O_{12}N_2ClP_2^{2+}$	489.31005	489.30972	0.68470

For Peer Review

PC G-17-1<br>PC G-17-2	$C_{21}H_{40}O_6P^+$	419.25570	419.25570	-0.00994
PC G-18	$C_{21}H_{37}O_3Cl_2^+$	407.21137	407.21143	-0.14200
PC G-19-1<br>PC G-19-2	$C_{21}H_{37}O_3^+$	337.27383	337.27372	0.33483
PC G-20	$C_{19}H_{37}O_3^+$	313.27384	313.27372	0.37148
PC G-21	$C_{18}H_{31}O^+$	263.23699	263.23694	0.19652
PC G-22	$C_{16}H_{31}O^+$	239.23694	239.23694	-0.00116
PC G-23	$C_6H_{17}O_4NP^+$	198.08900	198.08897	0.16822
PC G-24	$C_5H_{15}O_4NP^+$	184.07343	184.07332	0.57959
PC G-25	$C_2H_6O_4P^+$	124.99984	124.99982	0.17490
PC G-26	$C_5H_{14}ON^+$	104.10701	104.10699	0.17898
PC G-27	$H_4O_4P^+$	98.98420	98.98417	0.29268
PC G-28	$C_5H_{12}N^+$	86.09643	86.09643	0.06216

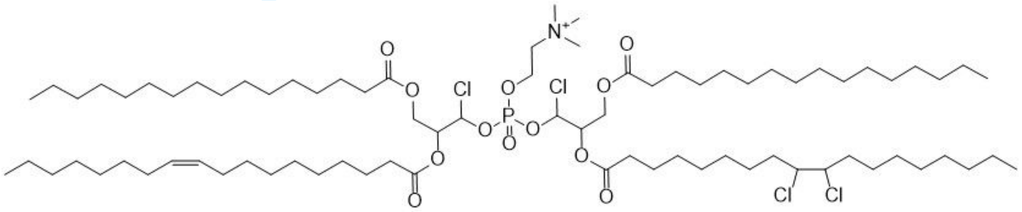

PC G-1

m/z 1474.95660

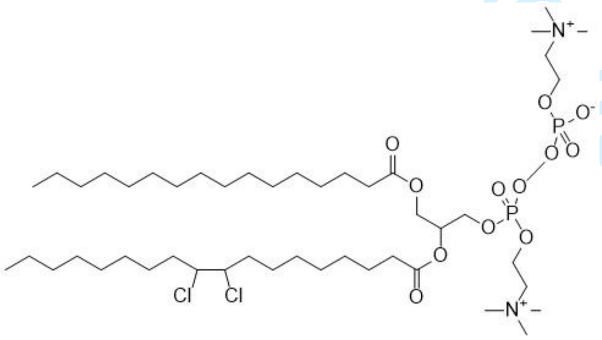

PC G-2

m/z 1011.57318

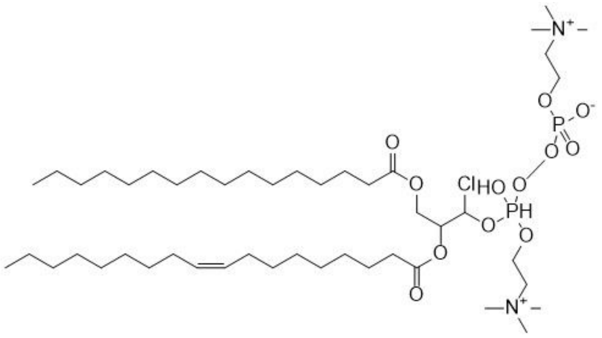

PC G-3

m/z 977.61215

For Review

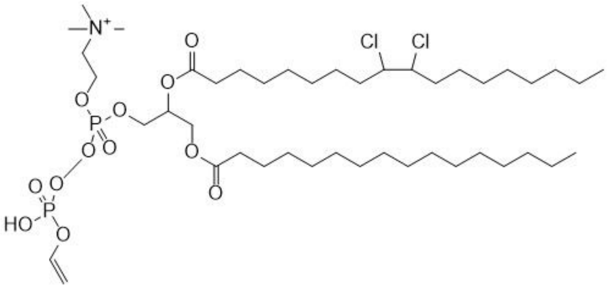

PC G-4

m/z 952.49968

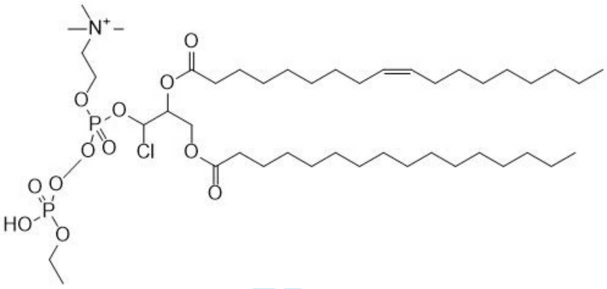

PC G-5-1

m/z 918.53865

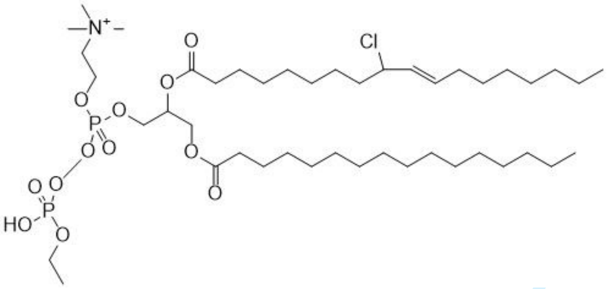

PC G-5-2

m/z 918.53865

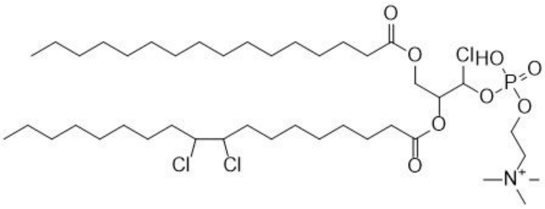

PC G-6

m/z 864.48381

PC G-7

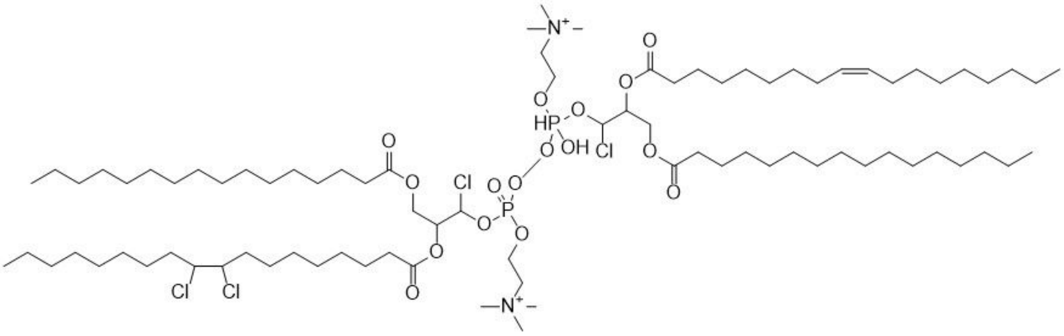

m/z 829.51496

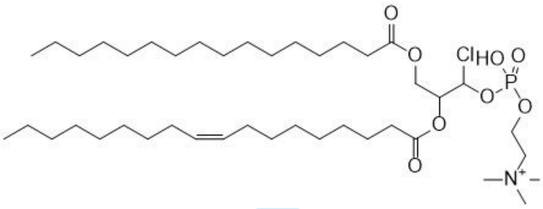

PC G-8-1

m/z 794.54611

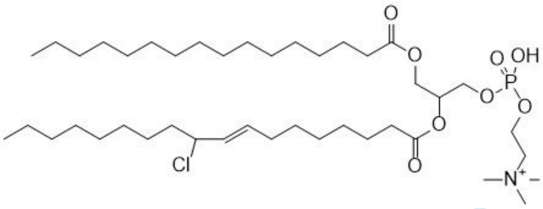

PC G-8-2

m/z 794.54611

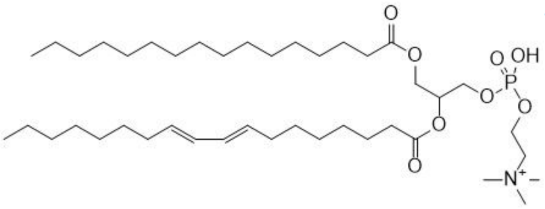

PC G-9

m/z 758.56943

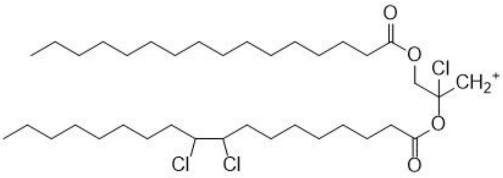

PC G-10

m/z 681.41777

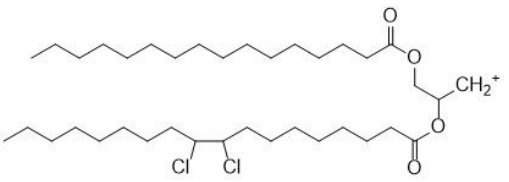

PC G-11

m/z 647.45674

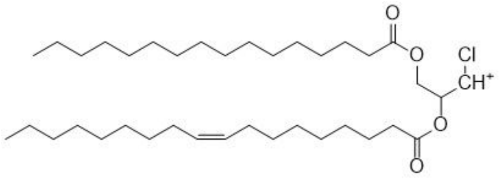

PC G-12-1

m/z 611.48006

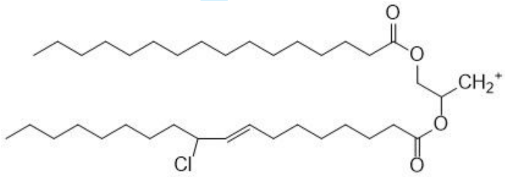

PC G-12-2

m/z 611.48006

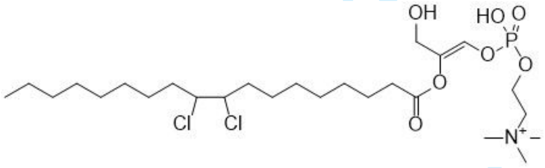

PC G-13

m/z 590.27747

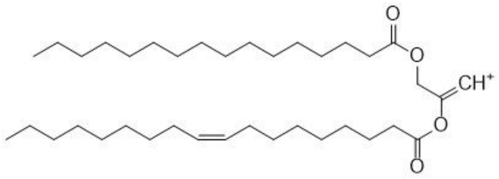

PC G-14-1

m/z 575.50339

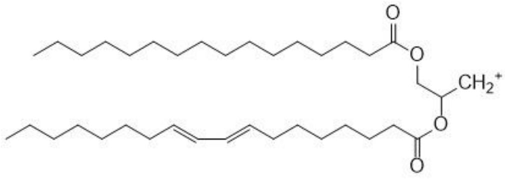

PC G-14-2

m/z 575.50339

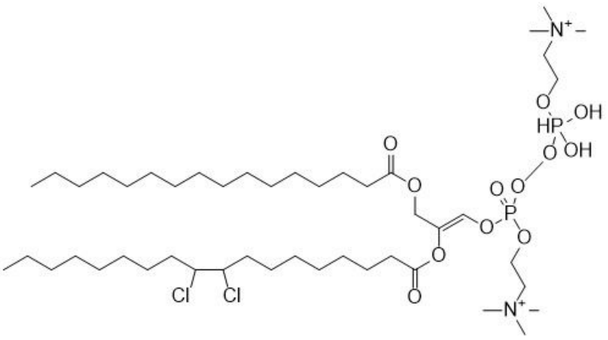

PC G-15

m/z 506.29023

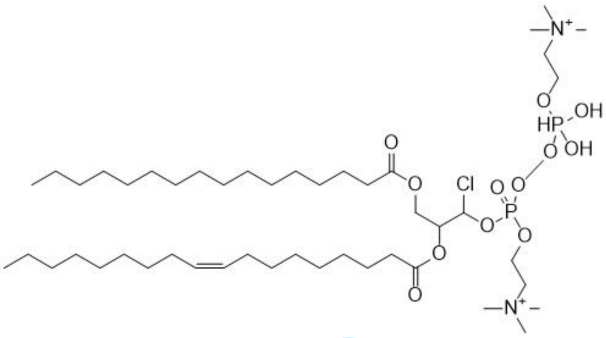

PC G-16-1

m/z 489.30972

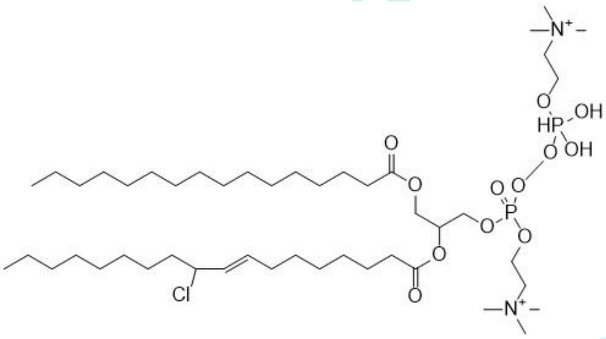

PC G-16-2

m/z 489.30972

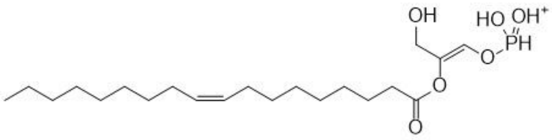

PC G-17-1

m/z 419.25570

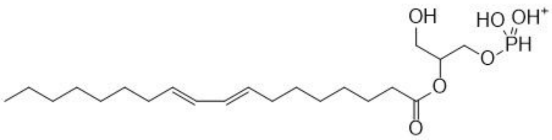

PC G-17-2

m/z 419.25570

PC G-18 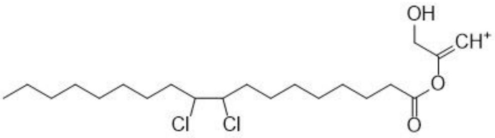

m/*z* 407.21143

PC G-19-1 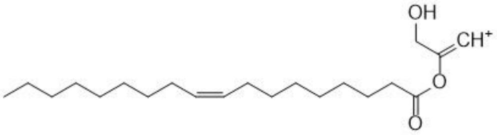

m/*z* 337.27372

PC G-19-2 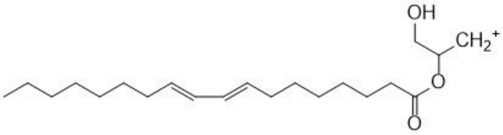

m/*z* 337.27372

PC G-20 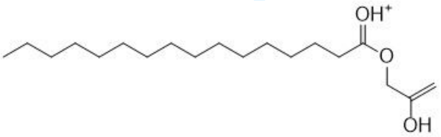

m/*z* 313.27372

PC G-21 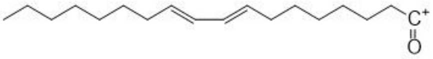

m/*z* 263.23694

PC G-22 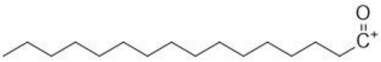

m/*z* 239.23694

PC G-23 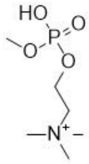

m/*z* 198.08897

PC G-24 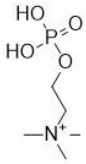

m/*z* 184.07332

PC G-25 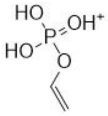

m/*z* 124.99982

PC G-26 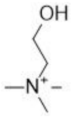

m/*z* 104.10699

PC G-27 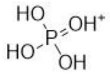

m/*z* 98.98417

PC G-28 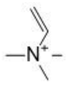

m/*z* 86.09643

Fig. S37 Proposed structures of fragments PC G-1–PC G-28

3.1.9 Fragmentation of PC H

Table S14 Fragments of PC H (chlorinated POPC)

PC H

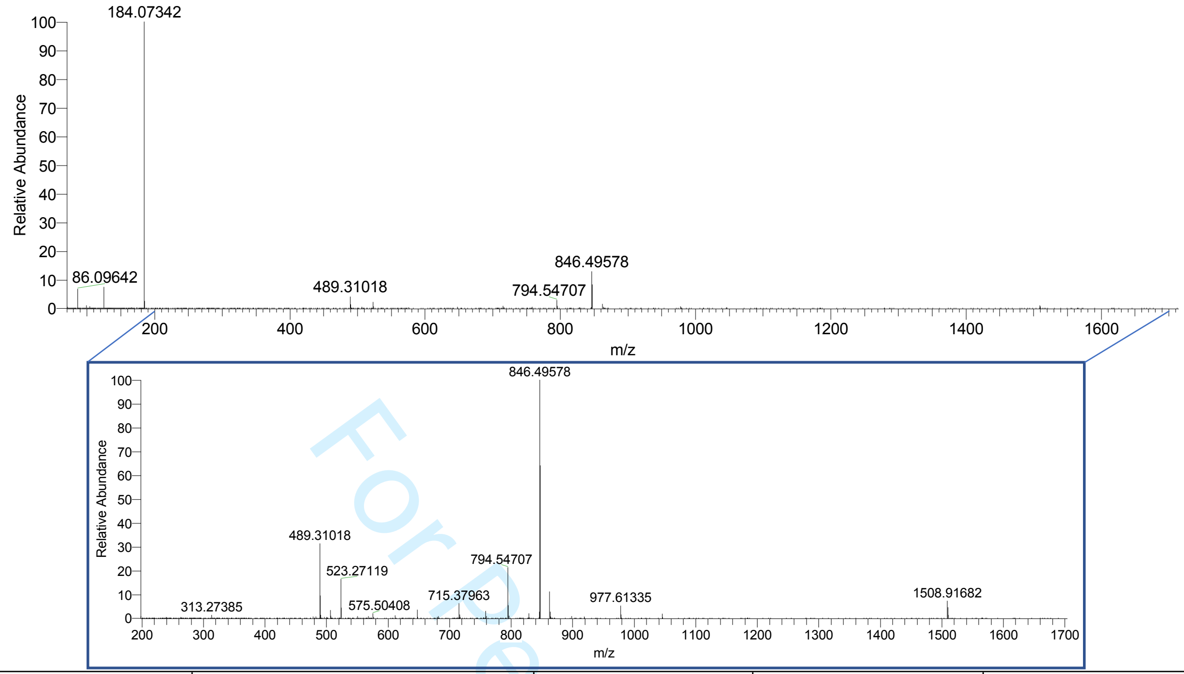

Fragment	Predicted formula	Measured *m/z*	Theoretical *m/z*	Mass difference [ppm]
PC H-1	$C_{79}H_{148}O_{12}NCl_5P^+$	1508.91682	1508.91763	-0.53681
PC H-2	$C_{47}H_{94}O_{12}N_2Cl_3P_2^+$	1045.53514	1045.53421	0.88951
PC H-3	$C_{47}H_{96}O_{12}N_2ClP_2^+$	977.61335	977.61215	1.22157
PC H-4	$C_{44}H_{87}O_{12}NClP_2^+$	918.53950	918.53865	0.92083
PC H-5	$C_{42}H_{81}O_8NCl_4P^+$	898.44567	898.44484	0.91973
PC H-6	$C_{42}H_{80}O_8NCl_3P^+$	862.46910	862.46816	1.08092
PC H-7	$C_{84}H_{163}O_{16}N_2Cl_5P_2^{2+}$	846.49578	846.49548	0.36288
PC H-8-1<br>PC H-8-2	$C_{42}H_{81}O_8NCl_2P^+$	828.50733	828.50714	0.23404
PC H-9-1<br>PC H-9-2	$C_{42}H_{82}O_8NClP^+$	794.54707	794.54611	1.20719
PC H-10	$C_{42}H_{81}O_8NP^+$	758.57047	758.56943	1.37152
PC H-11	$C_{37}H_{67}O_4Cl_4^+$	715.37963	715.37880	1.15740
PC H-12	$C_{37}H_{68}O_4Cl_3^+$	681.41813	681.41777	0.52652
PC H-13-1<br>PC H-13-2	$C_{37}H_{69}O_4Cl_2^+$	647.45731	647.45674	0.88326
PC H-14-1<br>PC H-14-2	$C_{37}H_{68}O_4Cl^+$	611.48049	611.48006	0.69511
PC H-15	$C_{37}H_{67}O_4^+$	575.50408	575.50339	1.19837
PC H-16-1<br>PC H-16-2	$C_{26}H_{52}O_7NClP^+$	556.31712	556.31644	1.20939

For Peer Review

PC H-17	$C_{47}H_{95}O_{12}N_2Cl_3P_2^{2+}$	523.27119	523.27074	0.85762
PC H-18	$C_{47}H_{96}O_{12}N_2Cl_2P_2^{2+}$	506.29059	506.29023	0.70700
PC H-19	$C_{23}H_{48}O_6NClP^+$	500.29065	500.29023	0.83717
PC H-20-1<br>PC H-20-2	$C_{47}H_{97}O_{12}N_2ClP_2^{2+}$	489.31018	489.30972	0.94642
PC H-21	$C_{21}H_{40}O_6P^+$	419.25598	419.25570	0.67144
PC H-22	$C_{21}H_{37}O_3^+$	337.27370	337.27372	-0.05336
PC H-23	$C_{19}H_{37}O_3^+$	313.27385	313.27372	0.40245
PC H-24	$C_{18}H_{31}O^+$	263.23695	263.23694	0.03579
PC H-25	$C_{16}H_{31}O^+$	239.23696	239.23694	0.08937
PC H-26	$C_6H_{17}O_4NP^+$	198.08903	198.08897	0.28389
PC H-27	$C_5H_{15}O_4NP^+$	184.07342	184.07332	-0.44451
PC H-28	$C_2H_6O_4P^+$	124.99983	124.99982	0.09204
PC H-29	$C_5H_{14}ON^+$	104.10700	104.10699	0.05997
PC H-30	$H_4O_4P^+$	98.98419	98.98417	0.20840
PC H-31	$C_5H_{12}N^+$	86.09642	86.09643	-0.04679

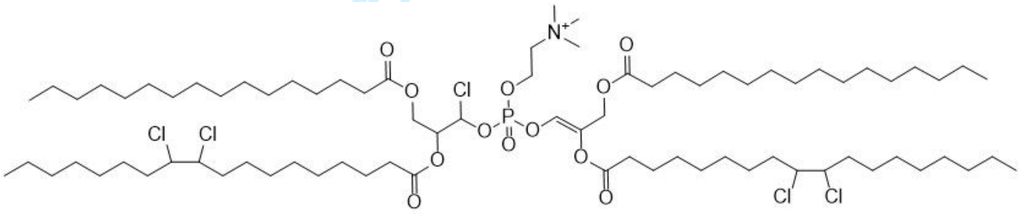

PC H-1

m/z 1508.91763

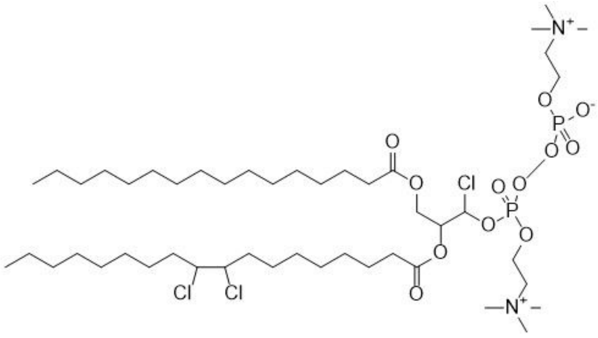

PC H-2

m/z 1045.53421

For Peer Review

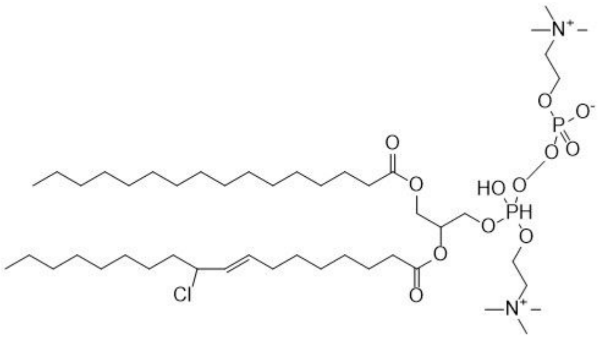

PC H-3

m/z 977.61215

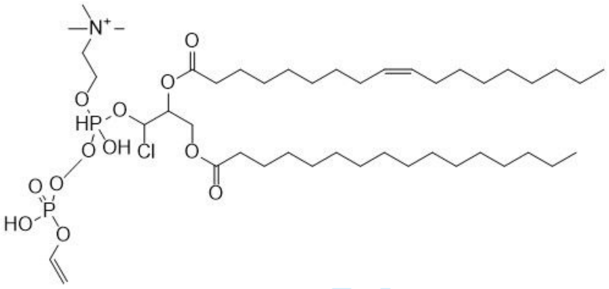

PC H-4

m/z 918.53865

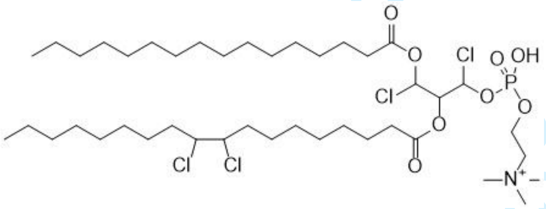

PC H-5

m/z 898.44484

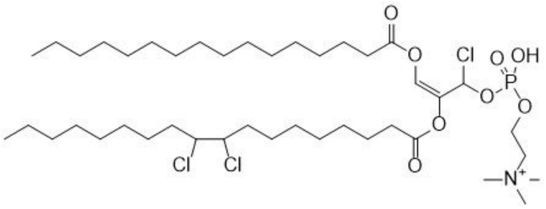

PC H-6

m/z 862.46816

PC H-7

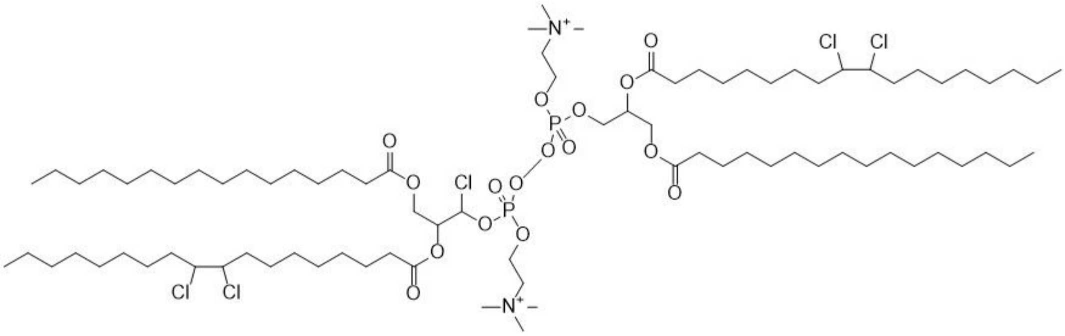

m/z 846.49548

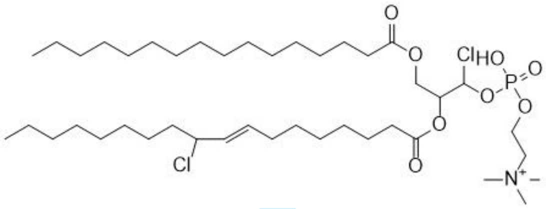

PC H-8-1

m/z 828.50714

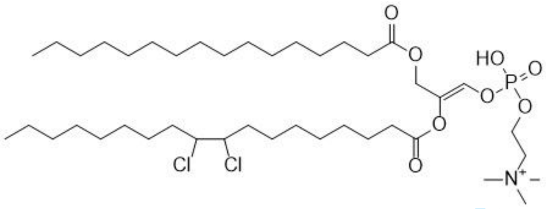

PC H-8-2

m/z 828.50714

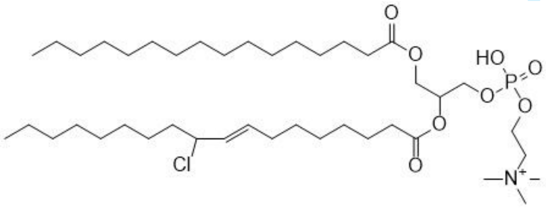

PC H-9-1

m/z 794.54611

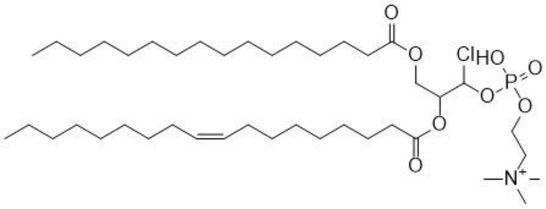

PC H-9-2

m/z 794.54611

**PC H-10**<br>*m/z* 758.56943	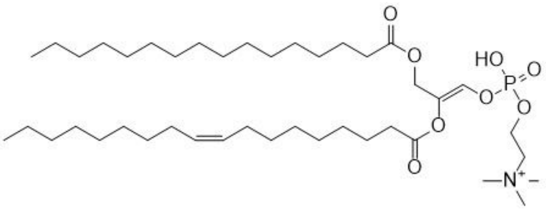
**PC H-11**<br>*m/z* 715.37880	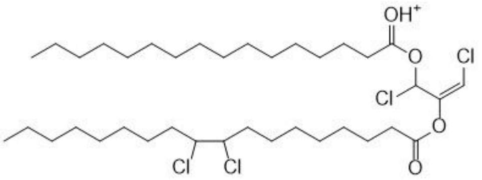
**PC H-12**<br>*m/z* 681.41777	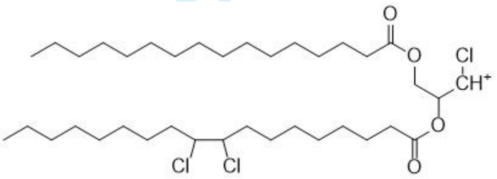
**PC H-13-1**<br>*m/z* 647.45674	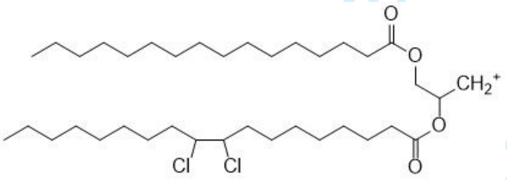
**PC H-13-2**<br>*m/z* 647.45674	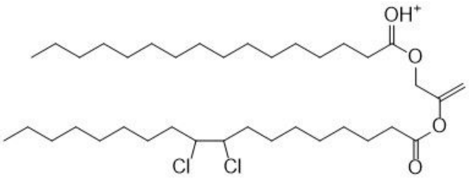
**PC H-14-1**<br>*m/z* 611.48006	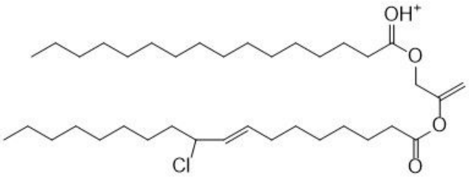

**PC H-14-2**<br>*m/z* 611.48006	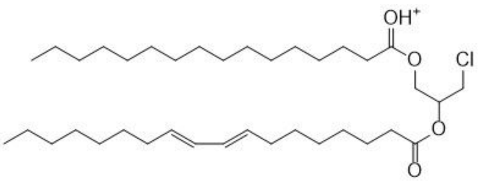
**PC H-15**<br>*m/z* 575.50339	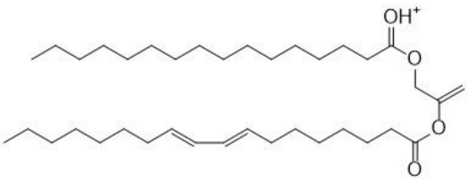
**PC H-16-1**<br>*m/z* 556.31644	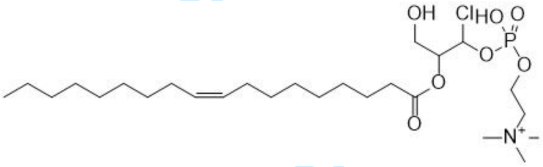
**PC H-16-2**<br>*m/z* 556.31644	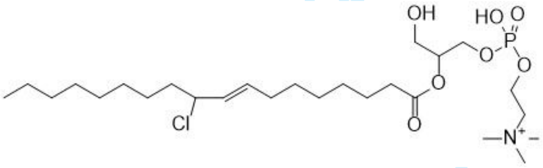
**PC H-17**<br>*m/z* 523.27074	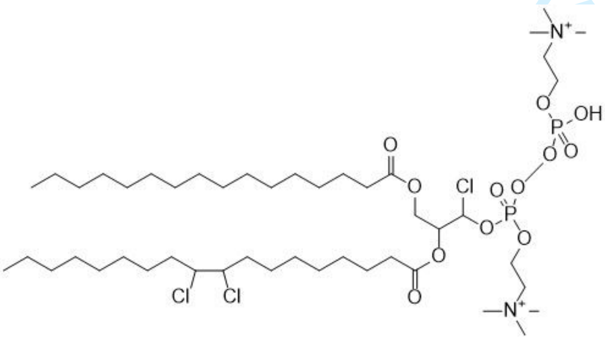

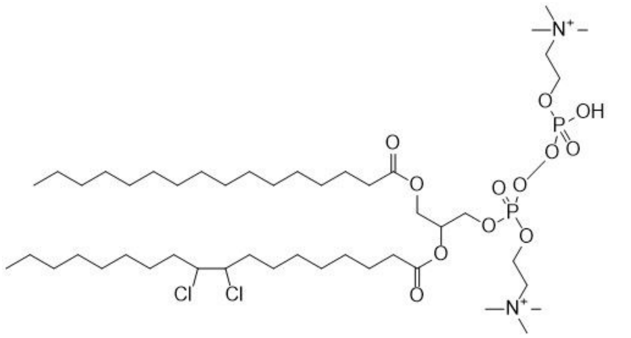

PC H-18

m/z 506.29023

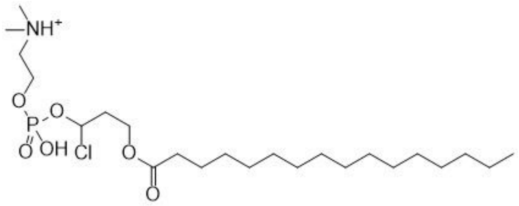

PC H-19

m/z 500.29023

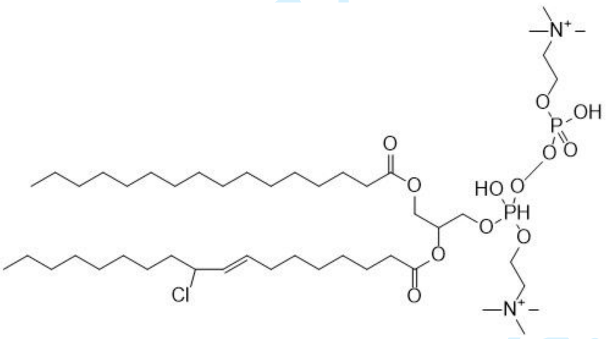

PC H-20-1

m/z 489.30972

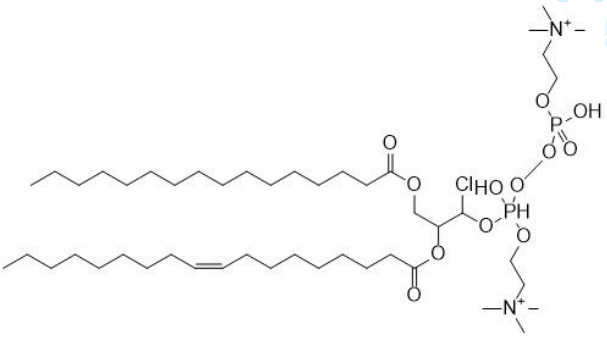

PC H-20-2

m/z 489.30972

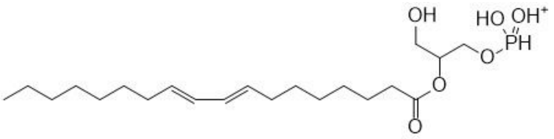

PC H-21-1

m/z 419.25570

PC H-21-2

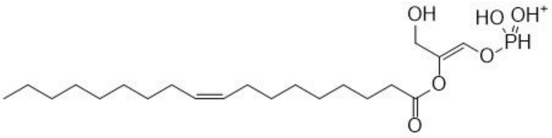

m/z 419.25570

PC H-22

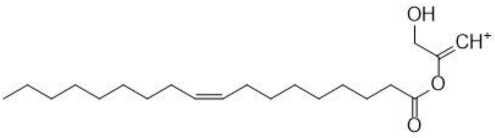

m/z 337.27372

PC H-23

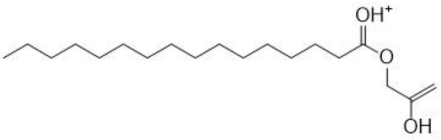

m/z 313.27372

PC H-24

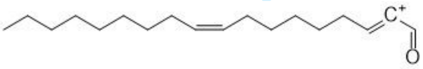

m/z 263.23694

PC H-25

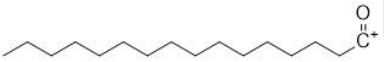

m/z 239.23694

PC H-26

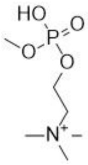

m/z 198.08897

PC H-27

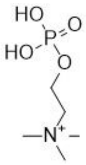

m/z 184.07332

PC H-28

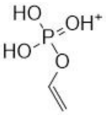

m/z 124.99982

PC H-29

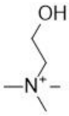

m/z 104.10699

PC H-30

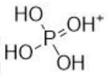

m/z 98.98417

PC H-31

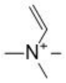

m/z 86.09643

Fig. S38 Proposed structures of fragments PC H-1–PC H-31

For Peer Review

3.1.10 Fragmentation of PC I

Table S15 Fragments of PC I (chlorinated POPC)

PC I

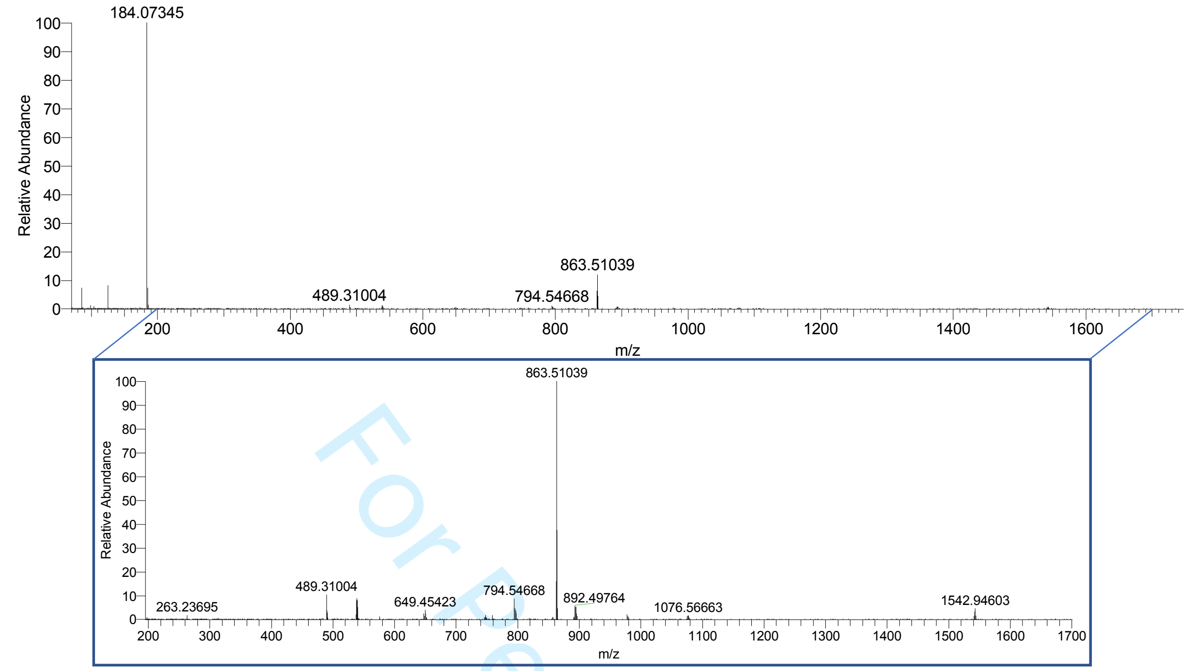

Fragment	Predicted formula	Measured *m/z*	Theoretical *m/z*	Mass difference [ppm]
PC I-1	$C_{79}H_{147}O_{12}N^{35}Cl_6P^+$	1542.87857	1542.87866	-0.05678
PC I-2	$C_{47}H_{93}O_{12}N_2Cl_4P_2^+$	1079.49772	1079.49524	2.30256
PC I-3-1<br>PC I-3-2<br>PC I-3-3	$C_{47}H_{96}O_{12}N_2ClP_2^+$	977.61255	977.61215	0.40389
PC I-4	$C_{44}H_{87}O_{12}NClP_2^+$	918.53657	918.53865	-2.26758
PC I-5	$C_{84}H_{162}O_{16}N_2^{35}Cl_6P_2^{2+}$	863.47371	863.47599	-2.63423
PC I-6-1<br>PC I-6-2	$C_{42}H_{81}O_8NCl_2P^+$	828.50720	828.50714	0.07896
PC I-7	$C_{42}H_{82}O_8NClP^+$	794.54668	794.54611	0.71885
PC I-8	$C_{42}H_{81}O_8NP^+$	758.56983	758.56943	0.52395
PC I-9	$C_{37}H_{71}O_6Cl_3P^+$	747.40495	747.40484	0.14686
PC I-10-1<br>PC I-10-2	$C_{37}H_{69}O_4Cl^{37}Cl^+$	649.45423	649.54379	0.66899
PC I-11-1<br>PC I-11-2	$C_{37}H_{69}O_4Cl_2^+$	647.45681	647.45674	0.10483
PC I-12-1<br>PC I-12-2	$C_{37}H_{68}O_4Cl^+$	611.48000	611.48006	-0.09834
PC I-13	$C_{37}H_{67}O_4^+$	575.50387	575.50339	0.84665
PC I-14-1<br>PC I-14-2	$C_{26}H_{52}O_7NClP^+$	556.31728	556.31644	1.49761

For Peer Review

PC I-15	$C_{47}H_{95}O_{12}N_2Cl_3P_2^{2+}$	523.27113	523.27074	0.73377
PC I-16	$C_{47}H_{96}O_{12}N_2Cl_2P_2^{2+}$	506.28996	506.29023	-0.53044
PC I-17	$C_{23}H_{48}O_6NClP^+$	500.29107	500.29023	1.67658
PC I-18-1<br>PC I-18-2	$C_{47}H_{97}O_{12}N_2ClP_2^{2+}$	489.31004	489.30972	0.67391
PC I-19	$C_{21}H_{40}O_6P^+$	419.25632	419.25570	1.46745
PC I-20	$C_{21}H_{37}O_3^+$	337.27403	337.27372	0.91413
PC I-21	$C_{19}H_{37}O_3^+$	313.27368	313.27372	-0.14752
PC I-22	$C_{18}H_{31}O^+$	263.23695	263.23694	0.03225
PC I-23	$C_{16}H_{31}O^+$	239.23694	239.23694	-0.01527
PC I-24	$C_6H_{17}O_4NP^+$	198.08901	198.08897	0.17405
PC I-25	$C_5H_{15}O_4NP^+$	184.07345	184.07332	-0.26864
PC I-26	$C_2H_6O_4P^+$	124.99985	124.99982	0.19990
PC I-27	$C_5H_{14}ON^+$	104.10701	104.10699	0.22765
PC I-28	$H_4O_4P^+$	98.98421	98.98417	0.38232
PC I-29	$C_5H_{12}N^+$	86.09643	86.09643	0.08213

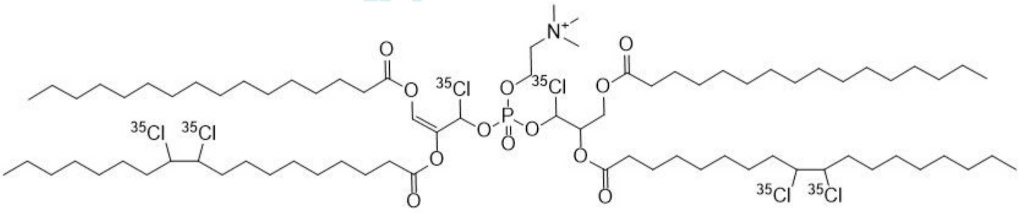

PC I-1

m/*z* 1542.87866

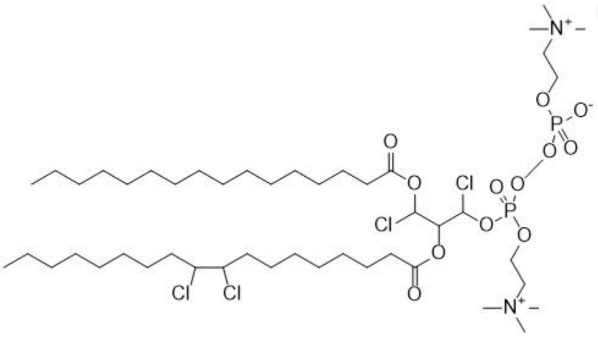

PC I-2

m/*z* 1079.49524

For Peer Review

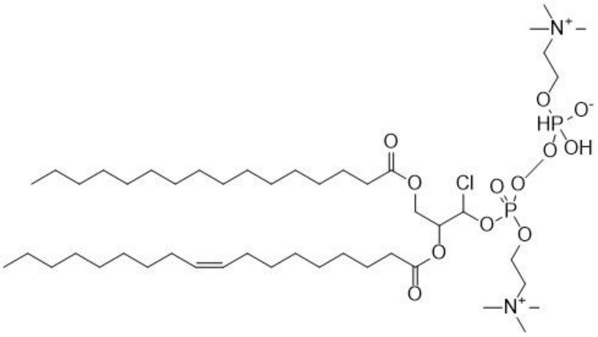

PC I-3-1

m/z 977.61215

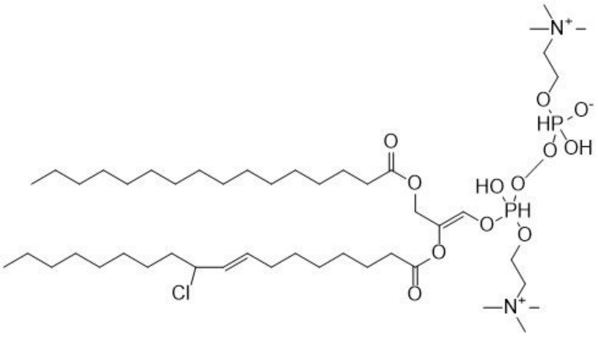

PC I-3-2

m/z 977.61215

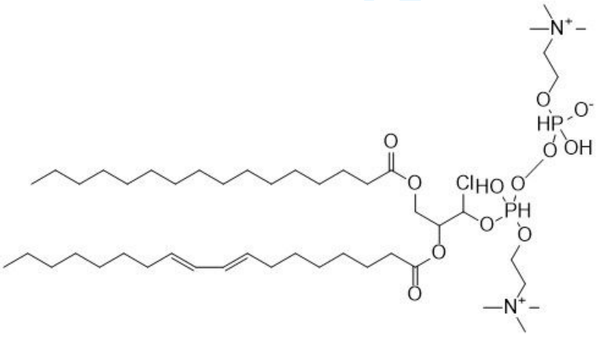

PC I-3-3

m/z 977.61215

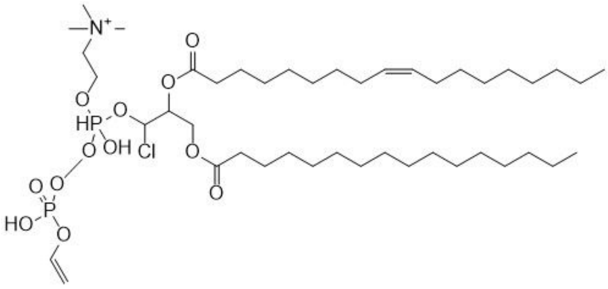

PC I-4

m/z 918.53865

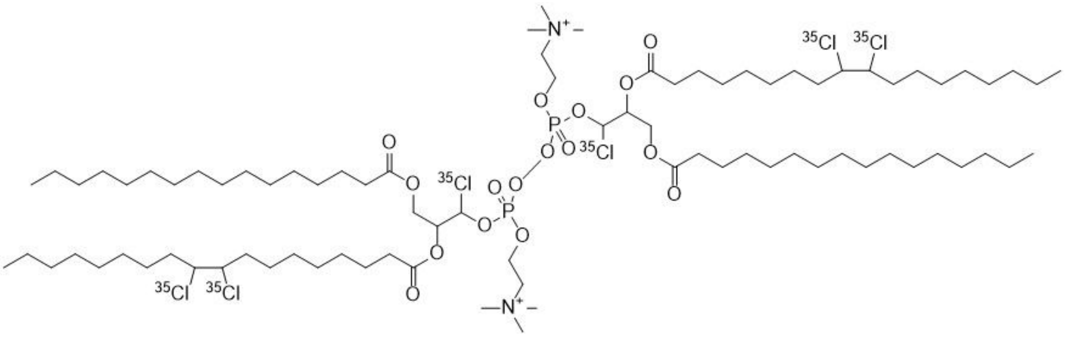

PC I-5

m/z 863.47599

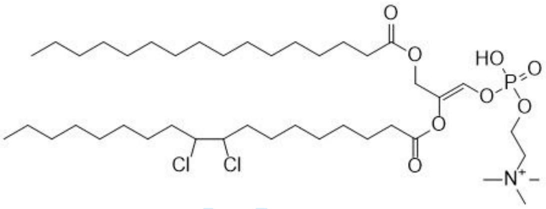

PC I-6-1

m/z 828.50714

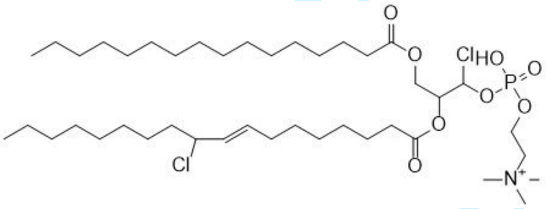

PC I-6-2

m/z 828.50714

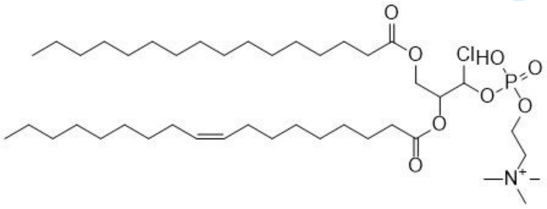

PC I-7

m/z 794.54611

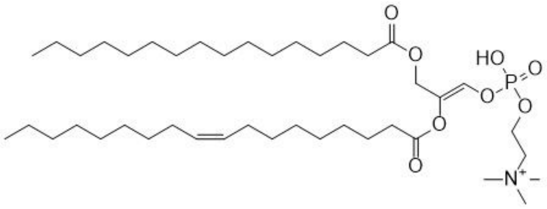

PC I-8

m/z 758.56943

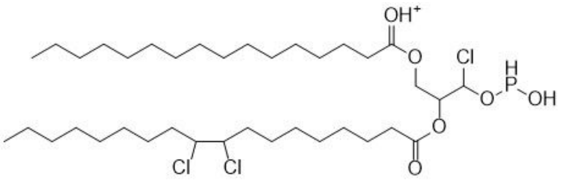

PC I-9

m/z 747.40484

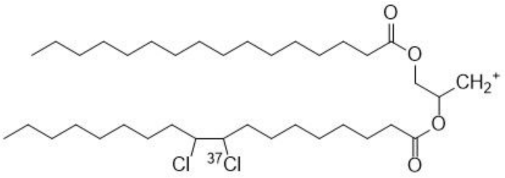

PC I-10-1

m/z 649.45379

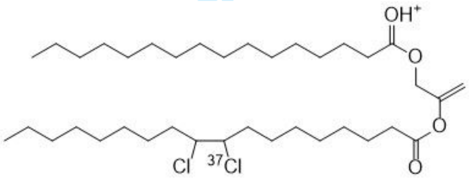

PC I-10-2

m/z 649.45379

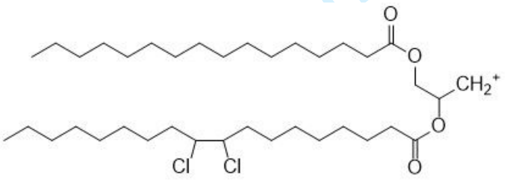

PC I-11-1

m/z 647.45674

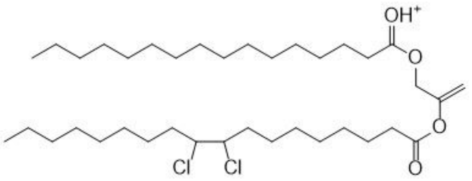

PC I-11-2

m/z 647.45674

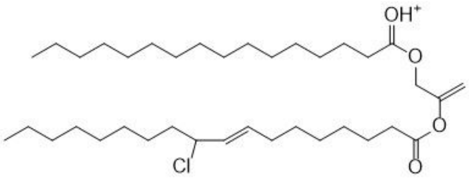

PC I-12-1

m/z 611.48006

**PC I-12-2**<br>*m/z* 611.48006	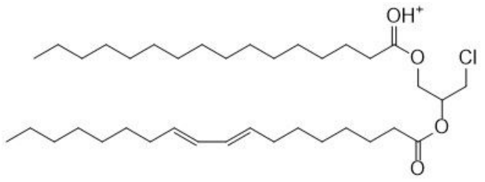
**PC I-13**<br>*m/z* 575.50339	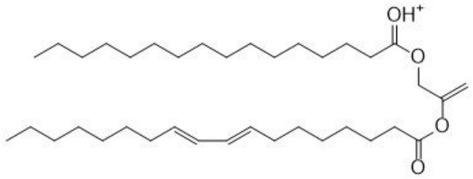
**PC I-14-1**<br>*m/z* 556.31644	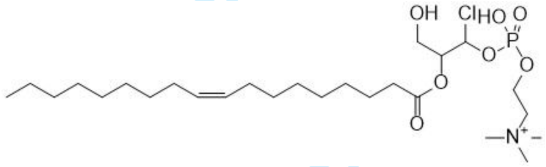
**PC I-14-2**<br>*m/z* 556.31644	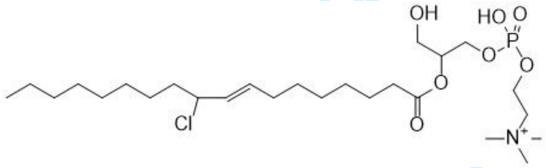
**PC I-15**<br>*m/z* 523.27074	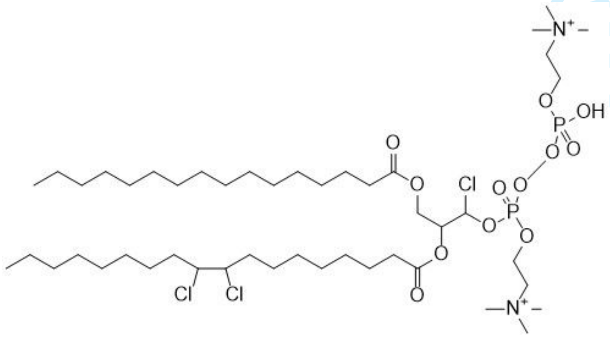

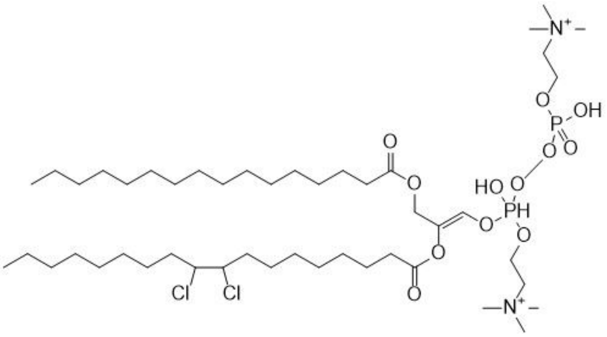

PC I-16

m/z 506.29023

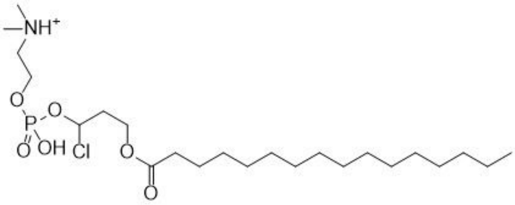

PC I-17

m/z 500.29023

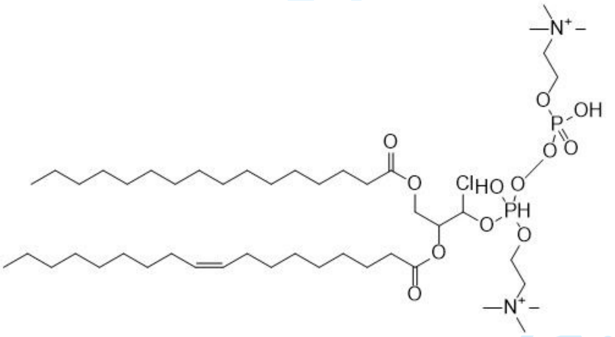

PC I-18-1

m/z 489.30972

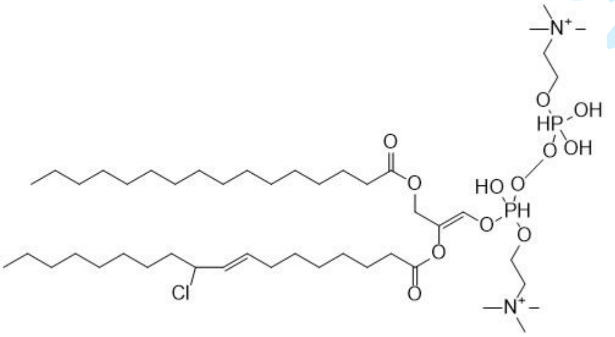

PC I-18-2

m/z 489.30972

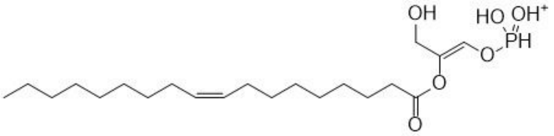

PC I-19

m/z 419.25570

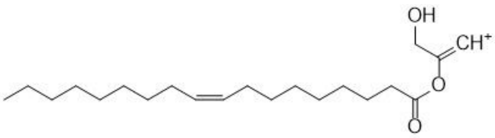

PC I-20

m/z 337.27372

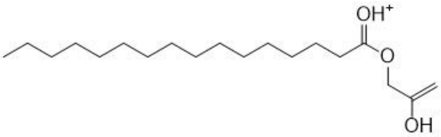

PC I-21

m/z 313.27372

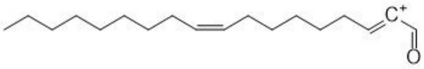

PC I-22

m/z 263.23694

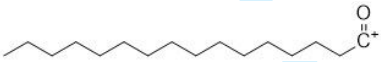

PC I-23

m/z 239.23694

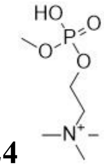

PC I-24

m/z 198.08897

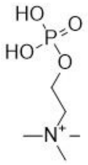

PC I-25

m/z 184.07332

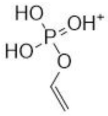

PC I-26

m/z 124.99982

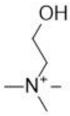

PC I-27

m/z 104.10699

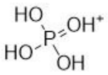

PC I-28

m/z 98.98417

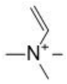

PC I-29

m/z 86.09643

Fig. S39 Proposed structures of fragments PC I-1–PC I-29

For Peer Review

3.1.11 Fragmentation of PC J

Table S16 Fragments of PC J (chlorinated POPC)

PC J

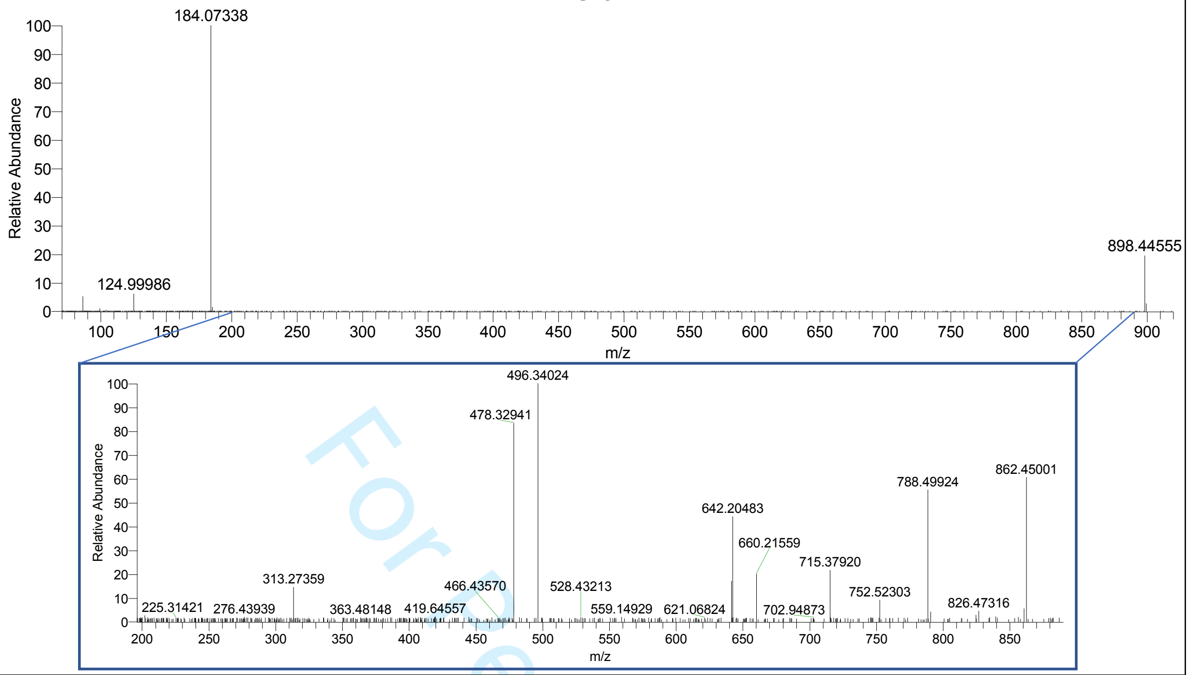

Fragment	Predicted formula	Measured *m/z*	Theoretical *m/z*	Mass difference [ppm]
PC J-1	$C_{42}H_{81}O_8NCl_4P^+$	898.44555	898.44484	0.79215
PC J-2	$C_{42}H_{78}O_8NCl_2{}^{37}ClP^+$	862.45001	862.44956	0.51172
PC J-3	$C_{42}H_{78}O_8NCl_3P^+$	860.45253	860.45251	0.01864
PC J-4	$C_{42}H_{76}O_8NClP^+$	788.49924	788.49916	0.10618
PC J-5	$C_{42}H_{75}O_8NP^+$	752.52303	752.52248	0.72579
PC J-6	$C_{37}H_{72}O_8Cl_2P^+$	745.43188	745.43364	-2.35125
PC J-7	$C_{37}H_{67}O_4Cl_4{}^+$	715.37920	715.37880	0.55595
PC J-8	$C_{26}H_{51}O_7NCl_4P^+$	660.21559	660.21518	0.62521
PC J-9	$C_{26}H_{49}O_6NCl_4P^+$	642.20483	642.20461	0.33578
PC J-10	$C_{24}H_{51}O_7NP^+$	496.34024	496.33977	0.60354
PC J-11	$C_{24}H_{49}O_6NP^+$	478.32941	478.32920	0.6999
PC J-12	$C_{21}H_{40}O_6P^+$	419.25497	419.25570	-1.73530
PC J-13	$C_{19}H_{37}O_3{}^+$	313.27359	313.27372	-0.41384
PC J-14	$C_5H_{15}O_4NP^+$	184.07338	184.07332	0.32327
PC J-15	$C_2H_6O_4P^+$	124.99986	124.99982	0.32506
PC J-16	$C_5H_{14}ON^+$	104.10702	104.10699	0.25134
PC J-17	$H_4O_4P^+$	98.98420	98.98417	0.33181
PC J-18	$C_5H_{12}N^+$	86.09645	86.09643	0.24434

For Peer Review

Table S17 Fragments of PC J or its isomer (chlorinated lung). Fragments that were detected in the single-lipid systems but not in the chlorinated lung sample are indicated with a hyphen (-)

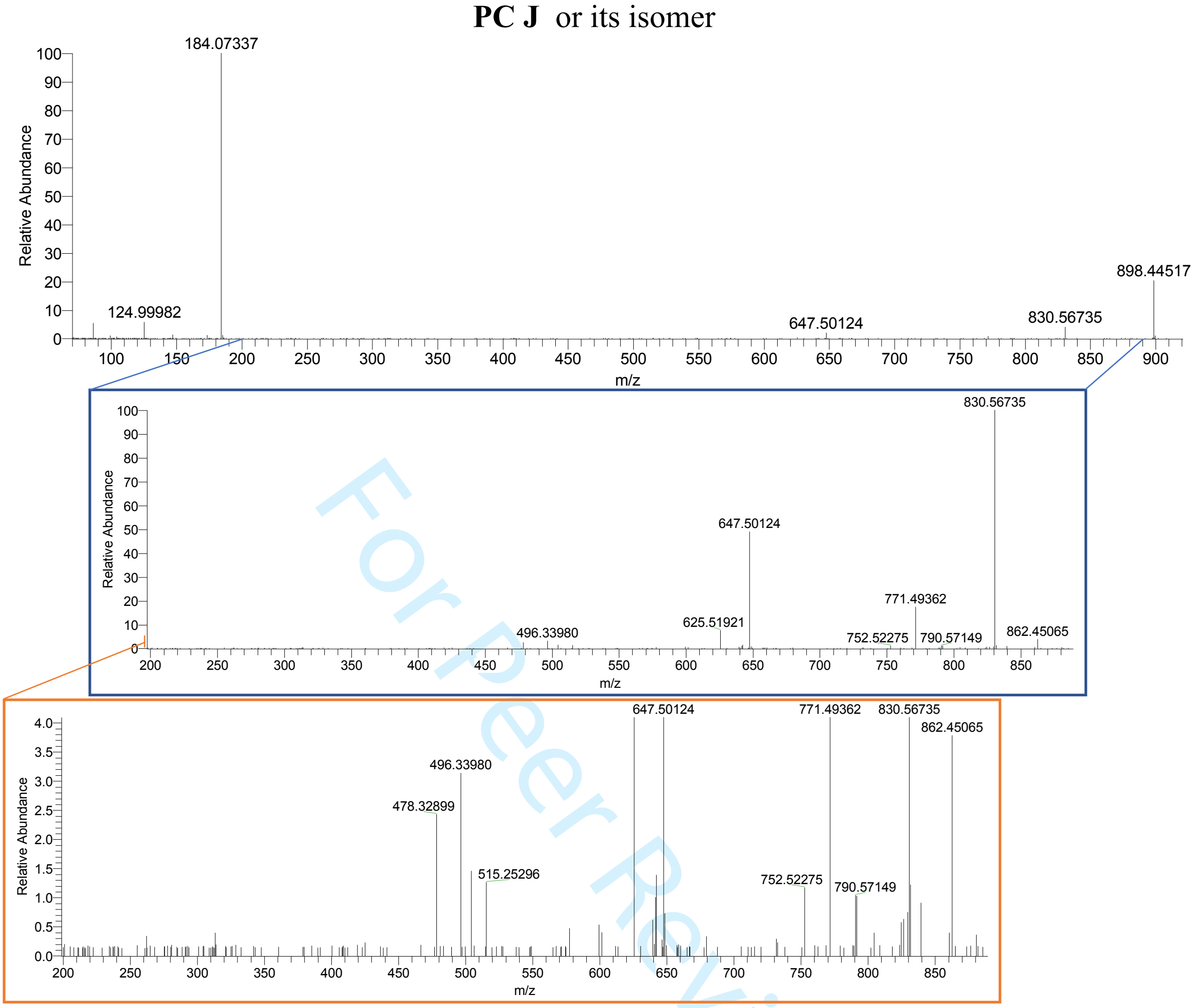

Fragment	Predicted formula	Measured *m/z*	Theoretical *m/z*	Mass difference [ppm]
PC J-1	$C_{42}H_{81}O_8NCl_4P^+$	898.44517	898.44484	0.36206
PC J-2	$C_{42}H_{78}O_8NCl_2{}^{37}ClP^+$	862.45049	862.44956	1.07472
PC J-3	$C_{42}H_{78}O_8NCl3P^+$	860.45187	860.45251	-0.74881
PC J-4	$C_{42}H_{76}O_8NClP^+$	788.49984	788.49916	0.83451
PC J-5	$C_{42}H_{75}O_8NP^+$	752.52275	752.52248	0.35877
PC J-6	$C_{37}H_{72}O_8Cl_2P^+$	-	745.43364	-
PC J-7	$C_{37}H_{67}O_4NCl4^+$	715.37872	715.37880	-0.10437
PC J-8	$C_{26}H_{51}O_7NCl_4P^+$	660.21387	660.21518	-1.98336
PC J-9	$C_{26}H_{49}O_6NCl_4P^+$	642.20494	642.20461	0.51812
PC J-10	$C_{24}H_{51}O_7NP^+$	496.33980	496.33977	0.07474
PC J-11	$C_{24}H_{49}O_6NP^+$	478.32899	478.32920	0.20823
PC J-12	$C_{21}H_{40}O_6P^+$	419.25525	419.25570	-1.08019
PC J-13	$C_{19}H_{37}O_3{}^+$	313.27425	313.27372	1.69437
PC J-14	$C_5H_{15}O_4NP^+$	184.07337	184.07332	0.24065

For Peer Review

PC J-15	$C_2H_6O_4P^+$	124.99982	124.99982	0.01673
PC J-16	$C_5H_{14}ON^+$	104.10698	104.10699	-0.11849
PC J-17	$H_4O_4P^+$	98.98554	98.98417	0.02534
PC J-18	$C_5H_{12}N^+$	86.09643	86.09643	0.08614

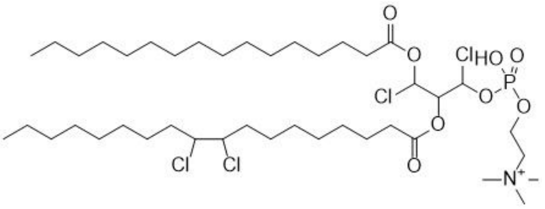

PC J-1

m/z 898.44484

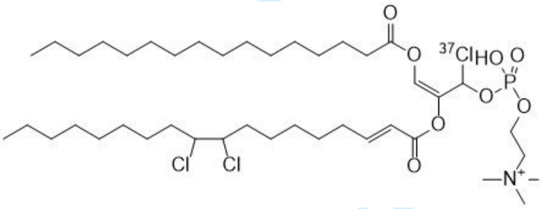

PC J-2

m/z 862.44956

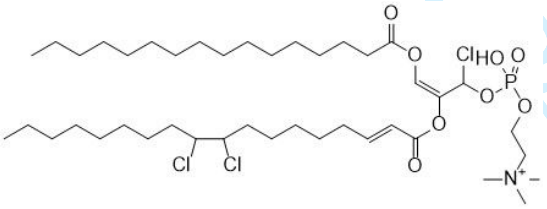

PC J-3

m/z 860.45251

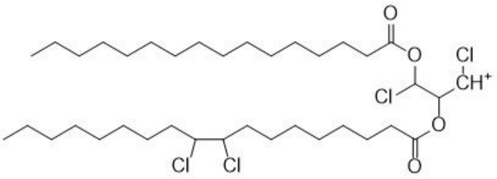

PC J-4

m/z 788.49916

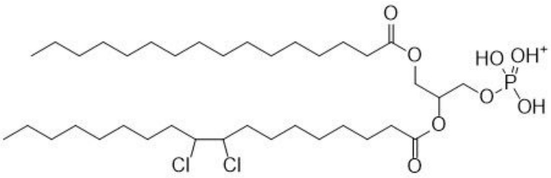

PC J-5

m/z 752.52248

For Peer Review

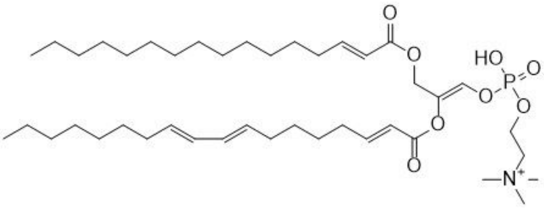

PC J-6

m/*z* 745.43364

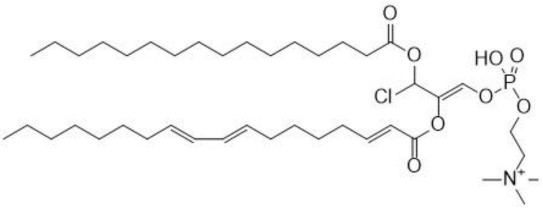

PC J-7

m/*z* 715.37880

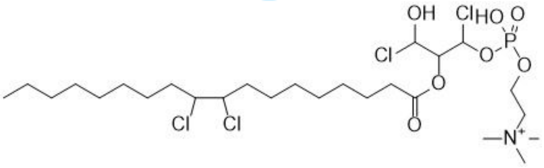

PC J-8

m/*z* 660.21518

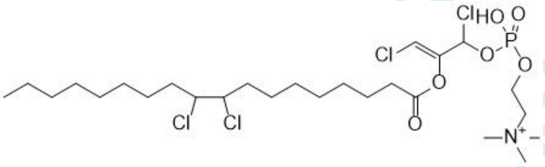

PC J-9

m/*z* 642.20494

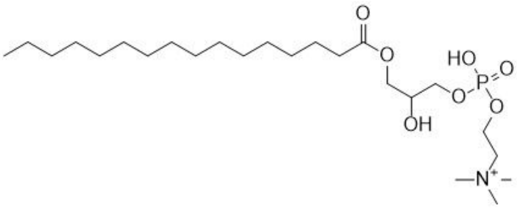

PC J-10

m/*z* 496.33977

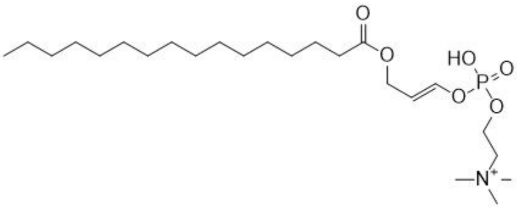

PC J-11

m/*z* 478.32920

For Review

**PC J-12** 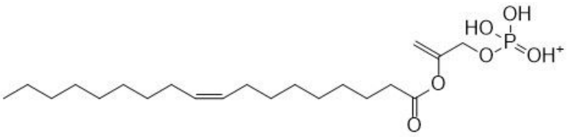<br>*m/z* 419.25570			
**PC J-13** 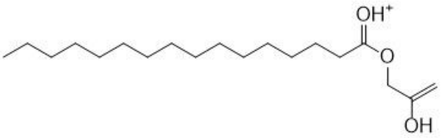<br>*m/z* 313.27372		**PC J-14** 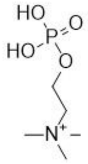<br>*m/z* 184.07332	
**PC J-15** 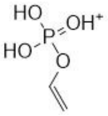<br>*m/z* 124.99982	**PC J-16** 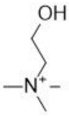<br>*m/z* 104.10699	**PC J-17** 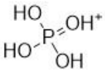<br>*m/z* 98.98417	**PC J-18** 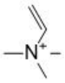<br>*m/z* 86.09643

Fig. S40 Proposed structures of fragments PC J-1–PC J-18

For Peer Review

3.1.12 Fragmentation of PC K

Table S18 Fragments of PC K (chlorinated POPC)

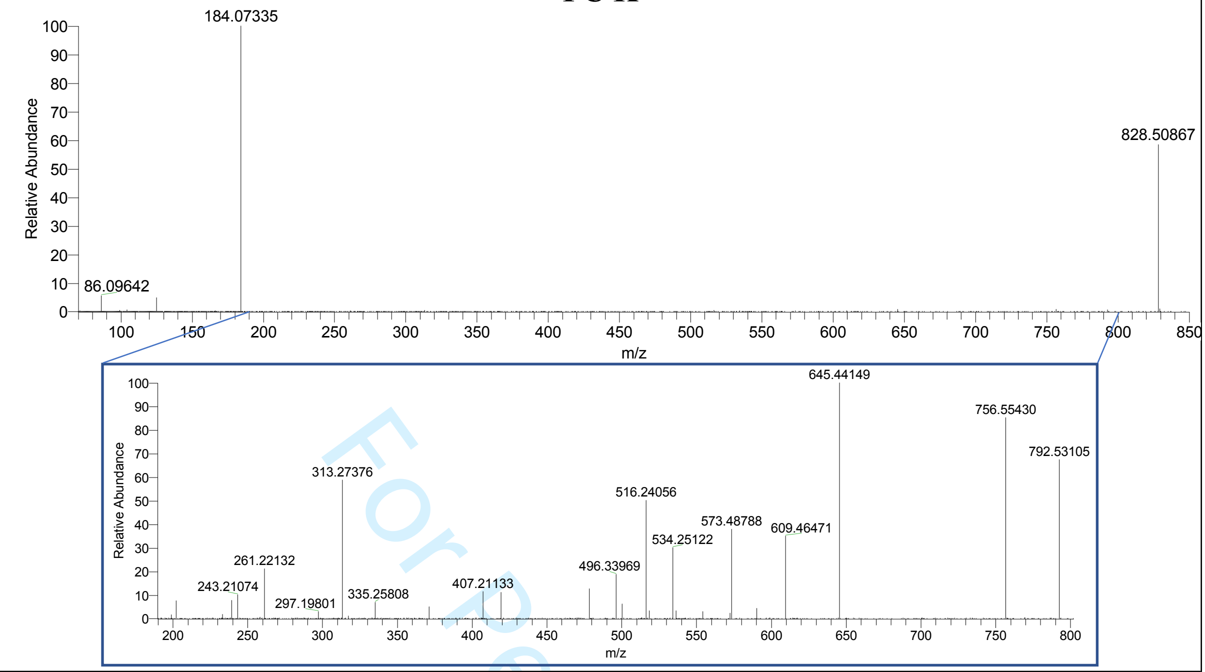

Fragment	Predicted formula	Measured *m/z*	Theoretical *m/z*	Mass difference [ppm]
PC K-1	$C_{42}H_{81}O_8NCl_2P^+$	828.50867	828.50714	1.85665
PC K-2	$C_{42}H_{80}O_8NClP^+$	792.53105	792.53046	0.74946
PC K-3	$C_{42}H_{79}O_8NP^+$	756.55430	756.55378	0.68070
PC K-4	$C_{37}H_{67}O_4Cl_2^+$	645.44149	645.44109	0.61244
PC K-5	$C_{37}H_{66}O_4Cl^+$	609.46471	609.46441	0.48685
PC K-6	$C_{26}H_{51}O_7NCl_2P^+$	590.27736	590.27747	-0.19601
PC K-7	$C_{37}H_{65}O_4^+$	573.48788	573.48774	0.24237
PC K-8	$C_{26}H_{50}O_7NClP^+$	554.30071	554.30079	-0.14966
PC K-9	$C_{23}H_{47}O_6NCl_2P^+$	534.25122	534.25126	-0.06894
PC K-10	$C_{23}H_{45}O_5NCl_2P^+$	516.24056	516.24069	-0.26195
PC K-11	$C_{24}H_{51}O_7NP^+$	496.33969	496.33977	-0.15130
PC K-12	$C_{24}H_{49}O_6NP^+$	478.32910	478.32920	-0.21757
PC K-13	$C_{21}H_{40}O_6P^+$	419.25574	419.25570	0.09321
PC K-14	$C_{19}H_{37}O_3^+$	313.27376	313.27372	0.13058
PC K-15	$C_5H_{14}O_3NClP^+$	202.03947	202.03943	0.20244
PC K-16	$C_5H_{12}O_5Cl^+$	199.03670	199.03660	-0.36645
PC K-17	$C_5H_{15}O_4NP^+$	184.07335	184.07332	0.17631
PC K-18	$C_2H_6O_4P^+$	124.99982	124.99982	-0.00608
PC K-19	$C_5H_{14}ON^+$	104.10697	104.10699	-0.17249
PC K-20	$H_4O_4P^+$	98.98416	98.98417	-0.08174

For Peer Review

PC K-21	$C_5H_{12}N^+$	86.09642	86.09643	-0.03334

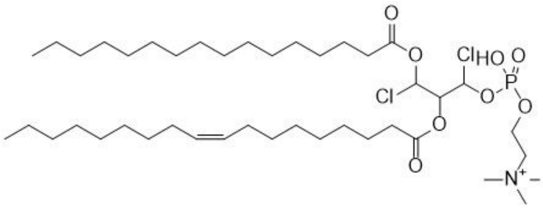

PC K-1

m/z 828.50714

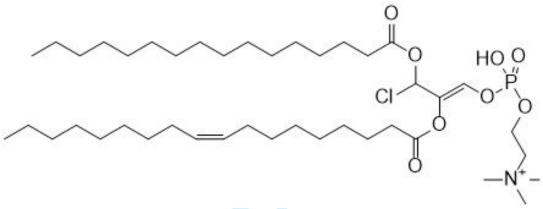

PC K-2

m/z 792.53046

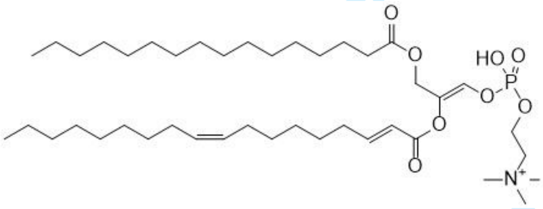

PC K-3

m/z 756.55378

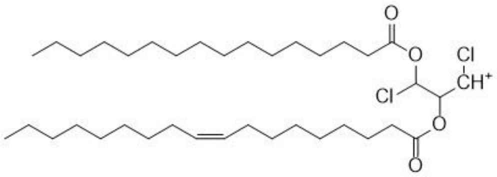

PC K-4

m/z 645.44109

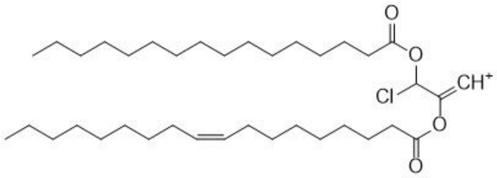

PC K-5

m/z 609.46441

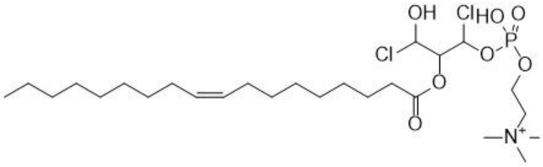

PC K-6

m/z 590.27747

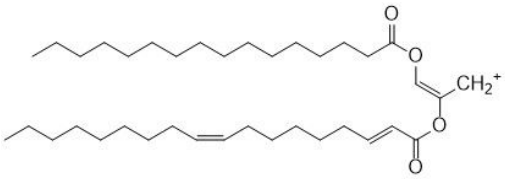

PC K-7

m/z 573.48774

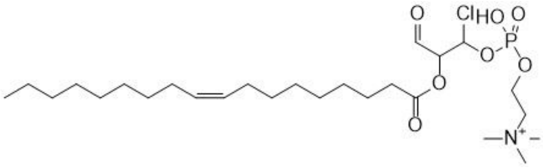

PC K-8

m/z 554.30079

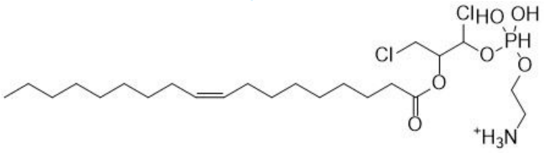

PC K-9

m/z 534.25126

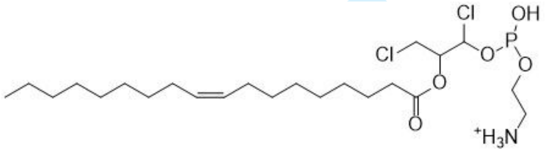

PC K-10

m/z 516.24069

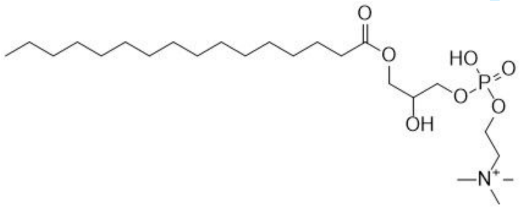

PC K-11

m/z 496.33977

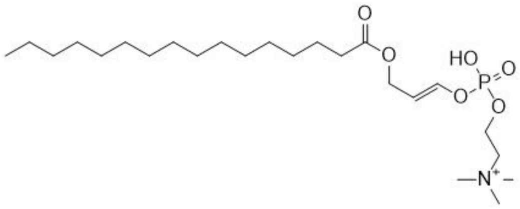

PC K-12

m/z 478.32920

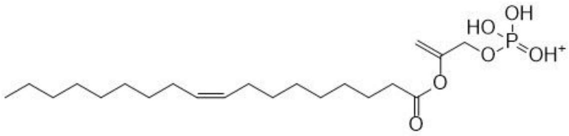

PC K-13

m/z 419.25570

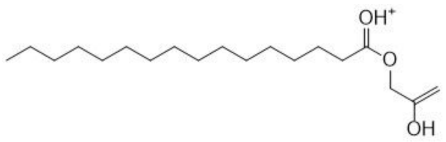

PC K-14
m/z 313.27372

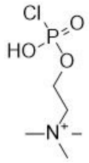

PC K-15
m/z 202.03943

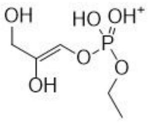

PC K-16
m/z 199.03660

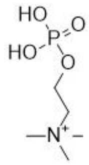

PC K-17
m/z 184.07332

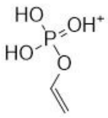

PC K-18
m/z 124.99982

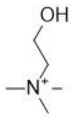

PC K-19
m/z 104.10699

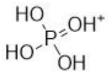

PC K-20
m/z 98.98417

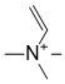

PC K-21
m/z 86.09643

Fig. S41 Proposed structures of fragments PC K-1–PC K-21

For Peer Review

3.1.13 Fragmentation of PC L

Table S19 Fragments of PC L (chlorinated POPC)

PC L

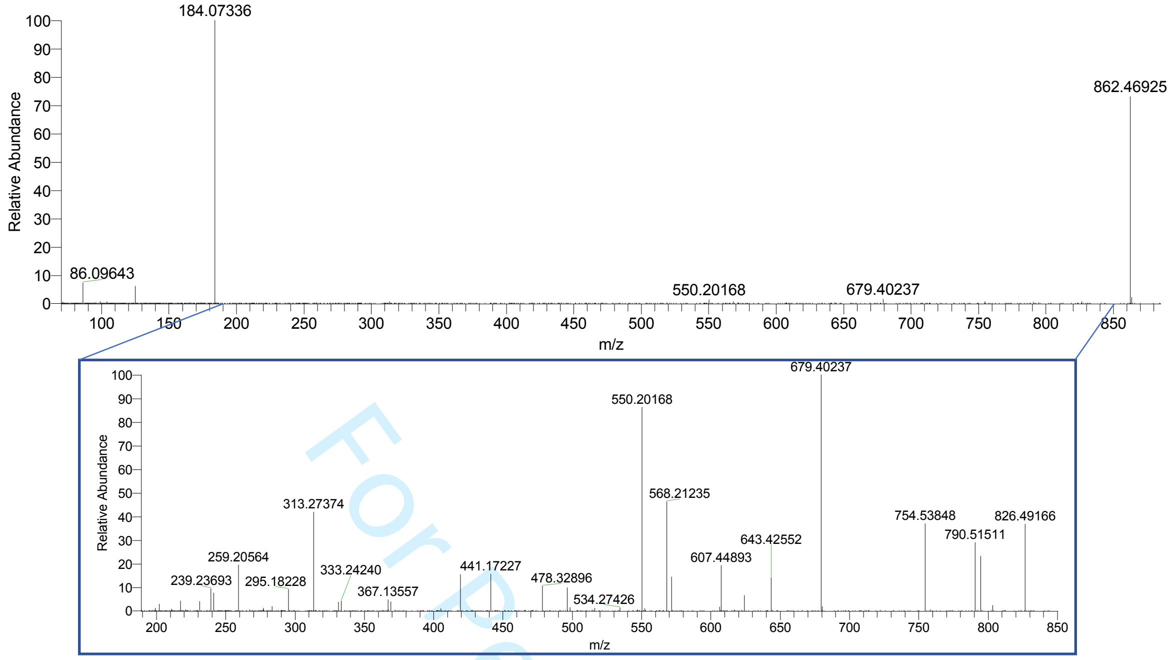

Fragment	Predicted formula	Measured *m/z*	Theoretical *m/z*	Mass difference [ppm]
PC L-1	$C_{42}H_{80}O_8NCl_3P^+$	862.46925	862.46816	1.25775
PC L-2	$C_{42}H_{79}O_8NCl_2P^+$	826.49166	826.49149	0.21141
PC L-3	$C_{42}H_{82}O_8NClP^+$	794.54647	794.54611	0.45360
PC L-4	$C_{42}H_{78}O_8NClP^+$	790.51511	790.51481	0.37653
PC L-5	$C_{42}H_{77}O_8NP^+$	754.53848	754.53813	0.45837
PC L-6	$C_{37}H_{66}O_4Cl_3^+$	679.40237	679.40212	0.36812
PC L-7	$C_{37}H_{65}O_4Cl_2^+$	643.42552	643.42544	0.11846
PC L-8	$C_{26}H_{50}O_7NCl_3P^+$	624.23885	624.23850	0.55622
PC L-9	$C_{37}H_{64}O_4Cl^+$	607.44893	607.44876	0.26698
PC L-10	$C_{37}H_{63}O_4^+$	571.47216	571.47209	0.12691
PC L-11	$C_{23}H_{46}O_6NCl_3P^+$	568.21235	568.21228	0.11902
PC L-12	$C_{23}H_{44}O_5NCl_3P^+$	550.20168	550.20172	-0.06559
PC L-13	$C_{24}H_{51}O_7NP^+$	496.33954	496.33977	-045206
PC L-14	$C_{24}H_{49}O_6NP^+$	478.32896	478.32920	-0.51209
PC L-15	$C_{20}H_{36}O_4Cl_2P^+$	441.17227	441.17228	-0.01778
PC L-16	$C_{21}H_{40}O_6P^+$	419.25565	419.25570	-0.13292
PC L-17	$C_{19}H_{37}O_3^+$	313.27374	313.27372	0.06461
PC L-18	$C_5H_{14}O_3NClP^+$	202.03945	202.03943	0.08051
PC L-19	$C_5H_{12}O_6P^+$	199.03671	199.03660	-0.33665
PC L-20	$C_5H_{15}O_4NP^+$	184.07336	184.07332	0.23763

For Peer Review

PC L-21	$C_2H_6O_4P^+$	124.99982	124.99982	0.00001
PC L-22	$C_5H_{14}ON^+$	104.10697	104.10699	-0.15339
PC L-23	$H_4O_4P^+$	98.98417	98.98417	0.01820
PC L-24	$C_5H_{12}N^+$	86.09643	86.09643	0.07431

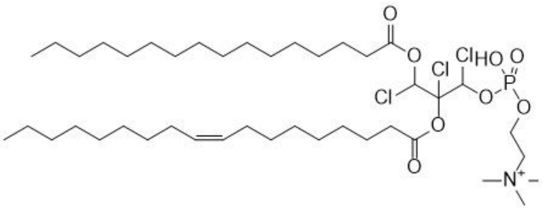

PC L-1

m/z 862.46816

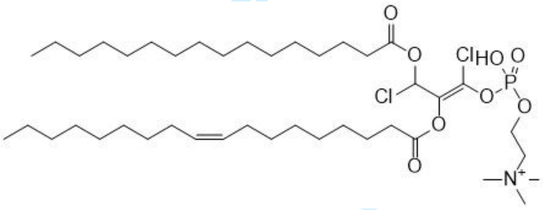

PC L-2

m/z 826.49149

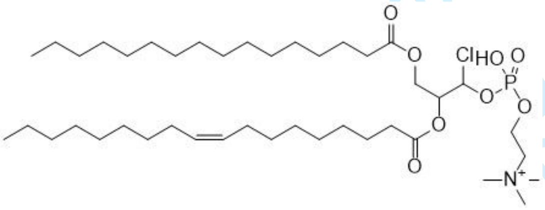

PC L-3

m/z 794.54611

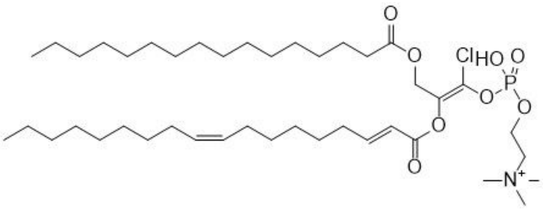

PC L-4

m/z 790.51481

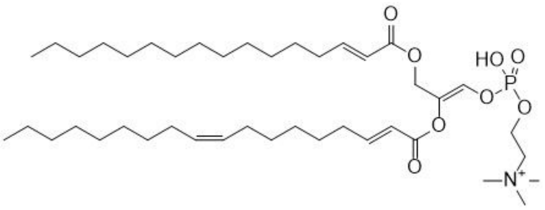

PC L-5

m/z 754.53813

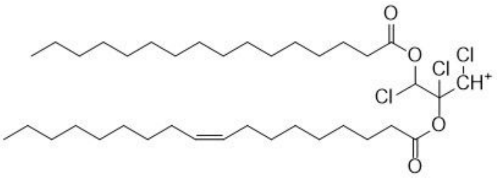

PC L-6

m/z 679.40212

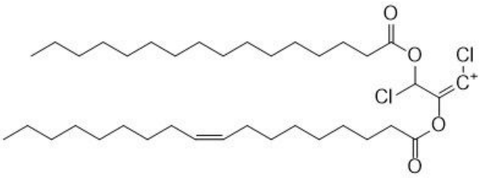

PC L-7

m/z 643.42544

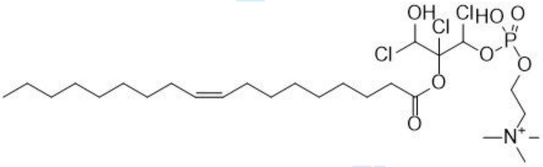

PC L-8

m/z 624.23850

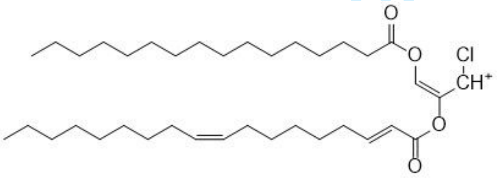

PC L-9

m/z 607.44876

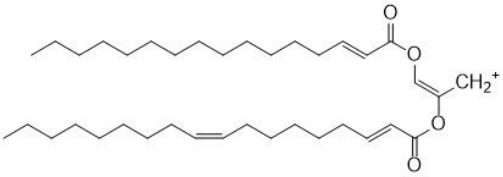

PC L-10

m/z 571.47209

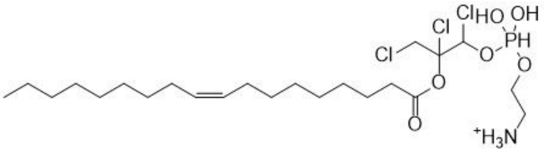

PC L-11

m/z 568.21228

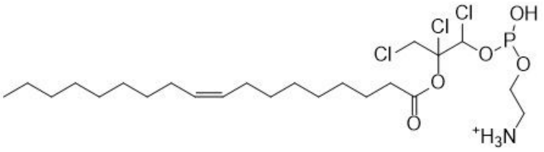

PC L-12

m/z 550.20172

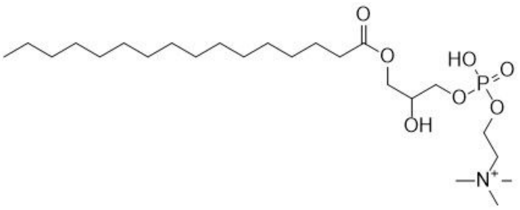

PC L-13

m/z 496.33977

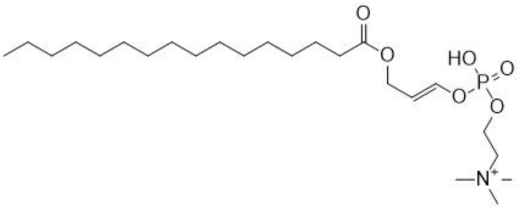

PC L-14

m/z 478.32920

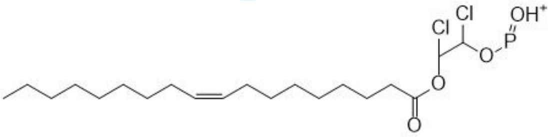

PC L-15

m/z 441.17228

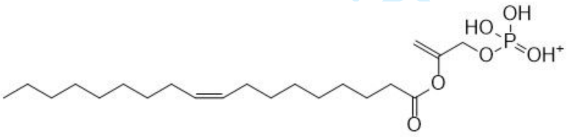

PC L-16

m/z 419.25570

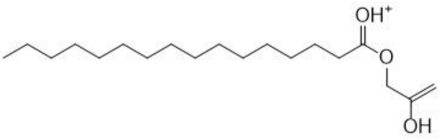

PC L-17

m/z 313.27372

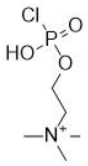

PC L-18

m/z 202.03943

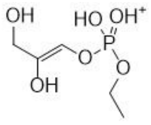

PC L-19

m/z 199.03660

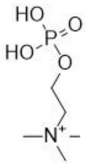

PC L-20

m/z 184.07332

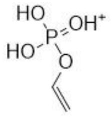

PC L-21

m/z 124.99982

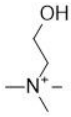

PC L-22

m/z 104.10699

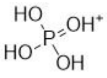

PC L-23

m/z 98.98417

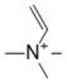

PC L-24

m/z 86.09643

Fig. S42 Proposed structures of fragments PC L-1–PC L-24

For Peer Review

3.2 Chlorinated PEs

3.2.1 Fragmentation of PE HOCl

Table S20 Fragments of PE HOCl (chlorinated POPE)

PE HOCl

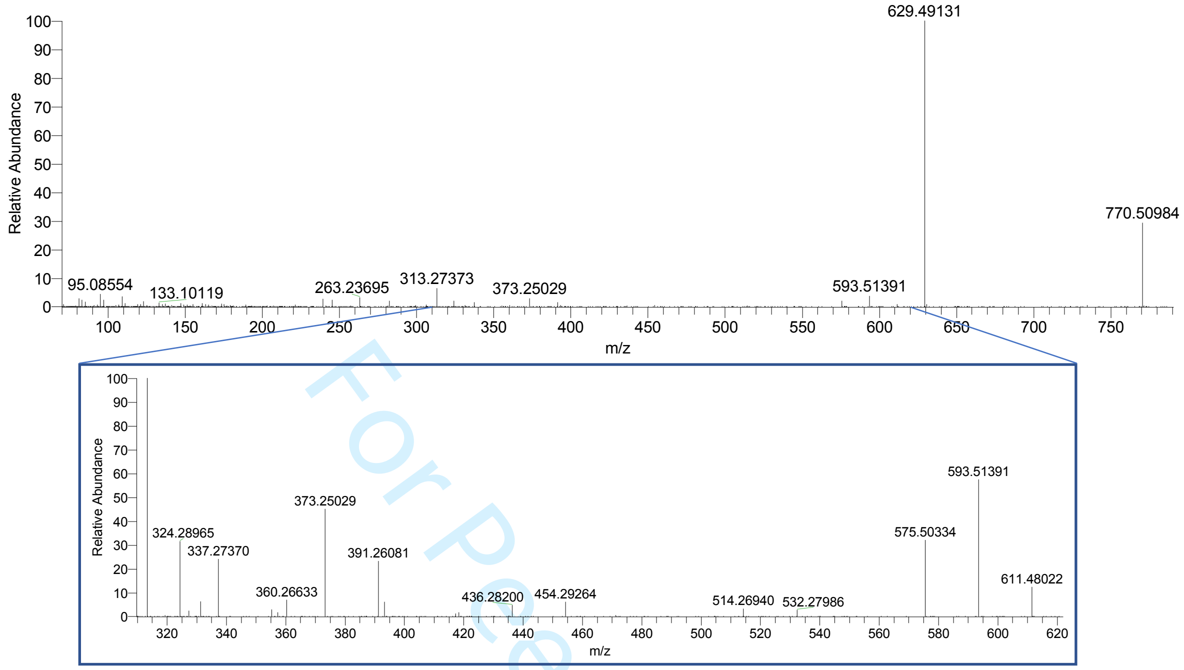

Fragment	Predicted formula	Measured *m/z*	Theoretical *m/z*	Mass difference [ppm]
PE HOCl-1	$C_{39}H_{78}O_9NClP^+$	770.50984	770.50972	0.15619
PE HOCl-2	$C_{39}H_{77}O_9NP^+$	734.53325	734.53305	0.27657
PE HOCl-3	$C_{37}H_{73}O_9ClP^+$	727.46743	727.46752	-0.12726
PE HOCl-4	$C_{39}H_{75}O_8NP^+$	716.52229	716.52248	-0.27005
PE HOCl-5	$C_{37}H_{70}O_5Cl^+$	629.49131	629.49063	1.08519
PE HOCl-6	$C_{37}H_{68}O_4Cl^+$	611.48022	611.48006	0.26142
PE HOCl-7	$C_{37}H_{69}O_5^+$	593.51391	593.51395	-0.07458
PE HOCl-8	$C_{37}H_{67}O4+$	575.50334	575.50339	-0.08993
PE HOCl-9	$C_{23}H_{46}O_7NClP^+$	514.26940	514.26949	-0.18811
PE HOCl-10	$C_{21}H_{45}O_7NP^+$	454.29264	454.29282	-0.38728
PE HOCl-11	$C_{21}H_{43}O_6NP^+$	436.28200	436.28225	-0.58564
PE HOCl-12	$C_{21}H_{41}O_5NP^+$	418.27163	418.27169	-0.12649
PE HOCl-13	$C_{21}H_{40}O_4Cl^+$	391.26081	391.26096	-0.38446
PE HOCl-14	$C_{21}H_{38}O_3Cl^+$	373.25029	373.25040	-0.30099
PE HOCl-15	$C_{21}H_{37}O_3^+$	337.27370	337.27372	-0.07472
PE HOCl-16	$C_{19}H_{37}O_3^+$	313.27373	313.27372	0.01219

For Peer Review

**PE HOCl-1**<br>*m/z* 770.50972	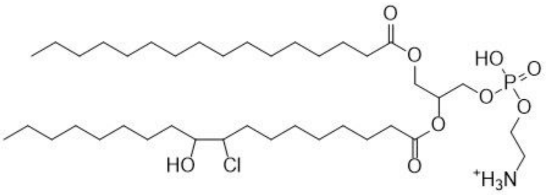
**PE HOCl-2**<br>*m/z* 734.53305	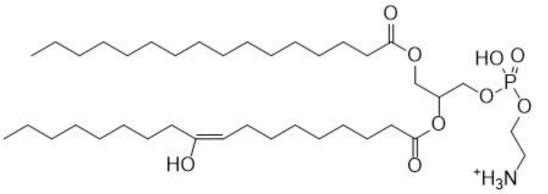
**PE HOCl-3**<br>*m/z* 727.46752	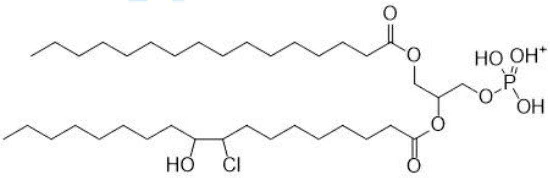
**PE HOCl-4**<br>*m/z* 716.52248	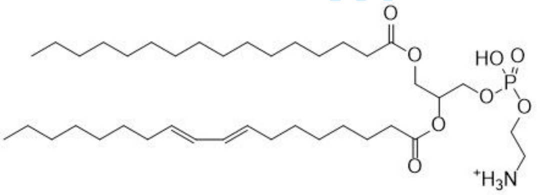
**PE HOCl-5**<br>*m/z* 629.49063	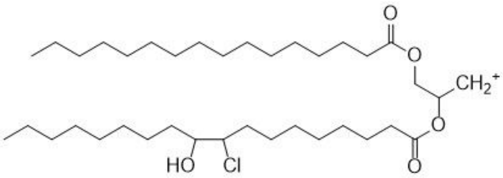
**PE HOCl-6**<br>*m/z* 611.48006	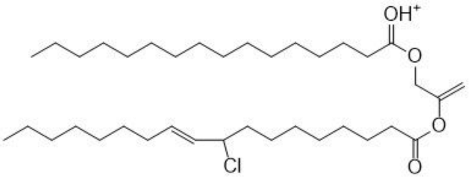

For Peer Review

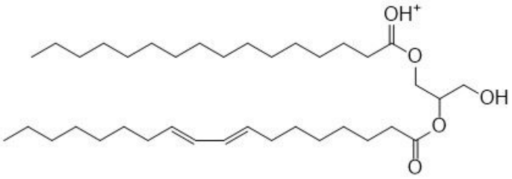

PE HOCl-7

m/*z* 593.51395

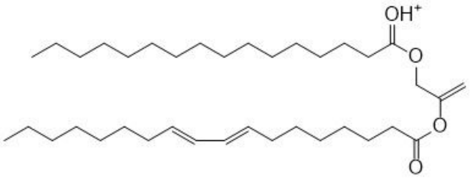

PE HOCl-8

m/*z* 575.50339

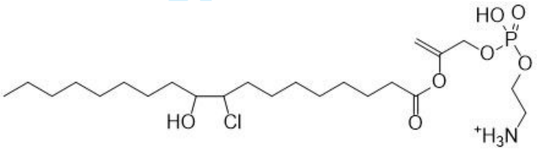

PE HOCl-9

m/*z* 514.26949

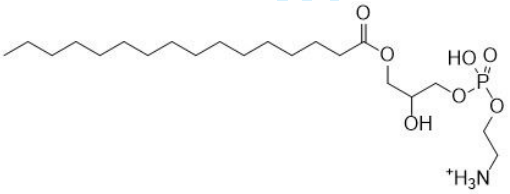

PE HOCl-10

m/*z* 454.29282

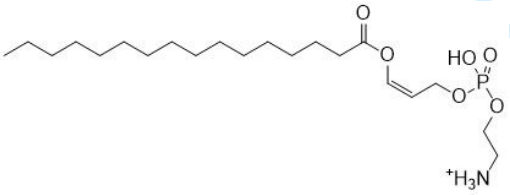

PE HOCl-11

m/*z* 436.28225

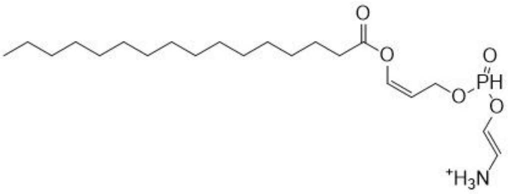

PE HOCl-12

m/*z* 418.27169

PE HOCl-13

m/z 391.26096

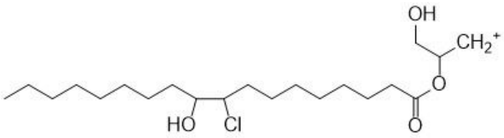

PE HOCl-14

m/z 373.25040

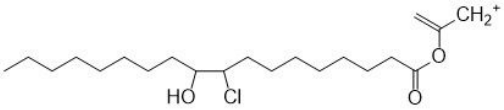

PE HOCl-15

m/z 337.27372

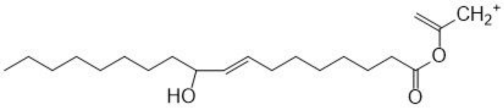

PE HOCl-16

m/z 313.27372

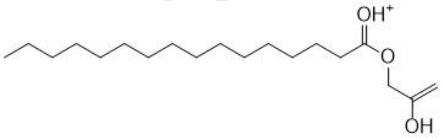

Fig. S43 Proposed structures of fragments PE HOCl-1–PE HOCl-16

For Peer Review

3.2.2 Fragmentation of PE A

Table S21 Fragments of PE A (chlorinated POPE)

PE A

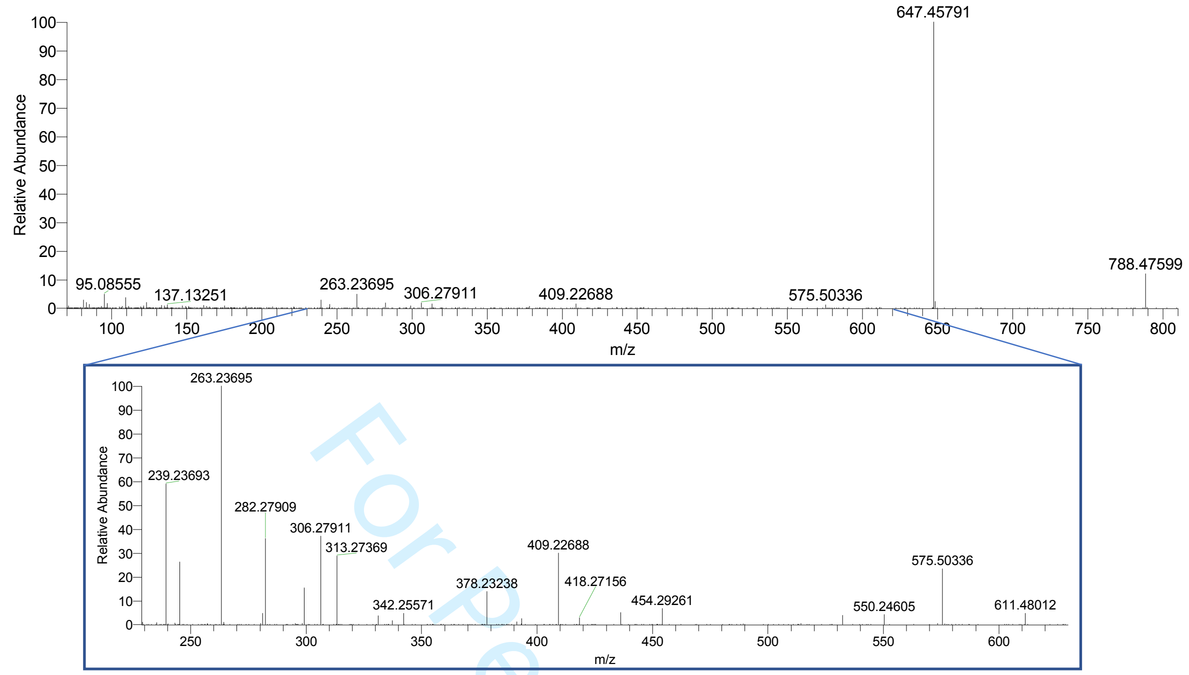

Fragment	Predicted formula	Measured *m/z*	Theoretical *m/z*	Mass difference [ppm]
PE A-1	$C_{39}H_{77}O_8NCl_2P^+$	788.47599	788.47584	-0.19483
PE A-2	$C_{37}H_{69}O_4Cl_2^+$	647.45791	647.45674	1.80579
PE A-3	$C_{37}H_{68}O_4Cl^+$	611.48022	611.48006	0.09811
PE A-4	$C_{37}H_{67}O_4^+$	575.50336	575.50339	-0.04597
PE A-5	$C_{23}H_{47}O_7NCl_2P^+$	550.24605	550.24617	-0.21449
PE A-6	$C_{23}H_{45}O_6NCl_2P^+$	532.23535	532.23561	-0.48065
PE A-7	$C_{21}H_{45}O_7NP^+$	454.29264	454.29282	-0.44274
PE A-8	$C_{21}H_{43}O_6NP^+$	436.28205	436.28225	-0.45668
PE A-9	$C_{21}H_{39}O_3Cl_2^+$	409.22688	409.22708	-0.47143
PE A-10	$C_{19}H_{37}O_3^+$	313.27369	313.27372	-0.09026
PE A-11	$C_{18}H_{31}O^+$	263.23695	263.23694	0.02725
PE A-12	$C_{16}H_{31}O^+$	239.23693	239.23694	-0.05187

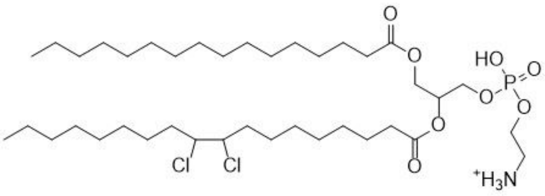

PE A-1

m/z 788.47584

For Peer Review

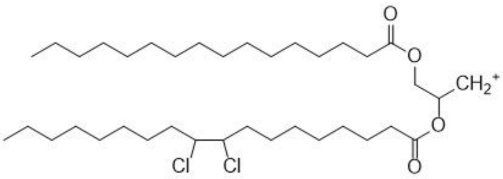

PE A-2

m/*z* 647.45674

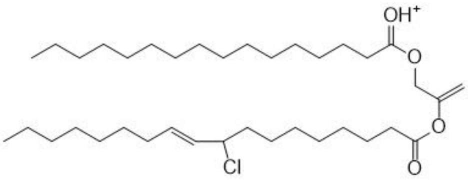

PE A-3

m/*z* 611.48006

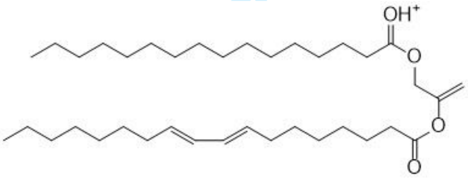

PE A-4

m/*z* 575.50339

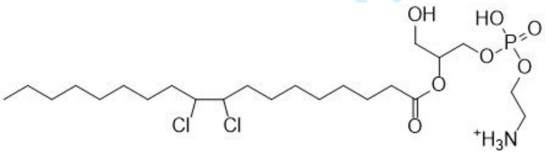

PE A-5

m/*z* 550.24617

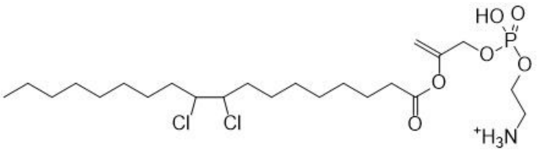

PE A-6

m/*z* 532.23561

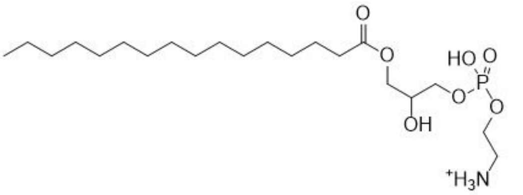

PE A-7

m/*z* 454.29282

PE A-8

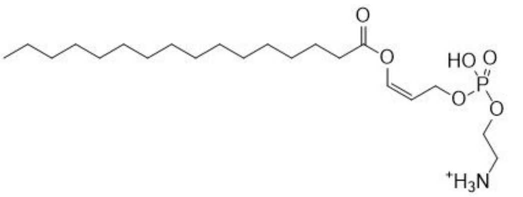

m/*z* 436.28225

PE A-9

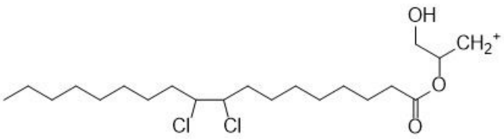

m/*z* 409.22708

PE A-10

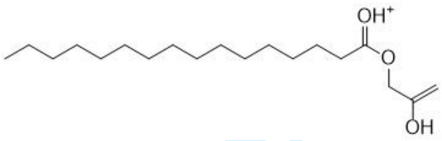

m/*z* 313.27372

PE A-11

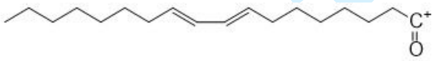

m/*z* 263.23694

PE A-12

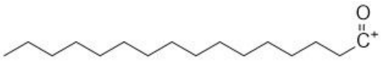

m/*z* 239.23694

Fig. S44 Proposed structures of fragments PE A-1–PE A-12

3.2.3 Fragmentation of PE B

Table S22 Fragments of PE B (chlorinated POPE)

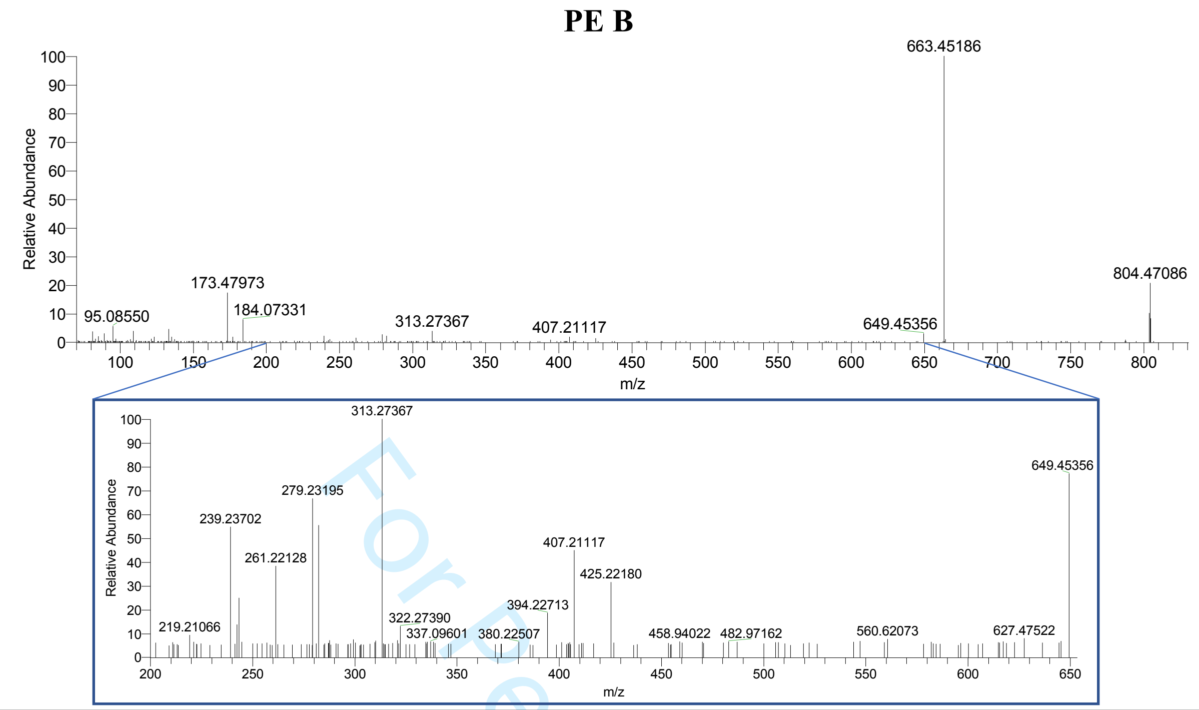

Fragment	Predicted formula	Measured *m/z*	Theoretical *m/z*	Mass difference [ppm]
PE B-1	$C_{39}H_{77}O_9NCl_2P^+$	804.47086	804.47075	0.13322
PE B-2	$C_{37}H_{69}O_5Cl_2^+$	663.45186	663.45166	0.31112
PE B-3	$C_{37}H_{67}O_4Cl_2^+$	645.44177	645.44109	1.05362
PE B-4	$C_{21}H_{39}O_4Cl_2^+$	425.22180	425.22199	-0.44596
PE B-5	$C_{21}H_{37}O_3Cl_2^+$	407.21117	407.21143	-0.61890
PE B-6	$C_{19}H_{37}O_3^+$	313.27367	313.27372	-0.16021
PE B-7	$C_{18}H_{31}O_2^+$	279.23195	279.23186	0.33282
PE B-8	$C_{18}H_{29}O^+$	261.22128	261.22129	-0.05309
PE B-9	$C_{16}H_{31}O^+$	239.23702	239.23694	0.32367

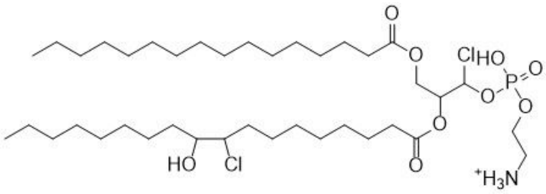

PE B-1

m/z 804.47075

For Peer Review

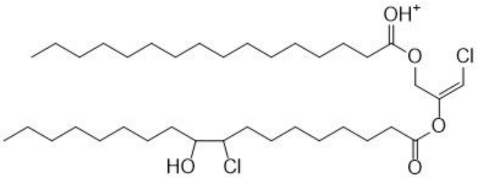

PE B-2

m/*z* 663.45166

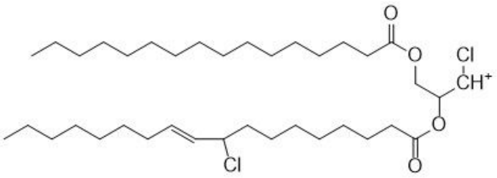

PE B-3

m/*z* 645.44109

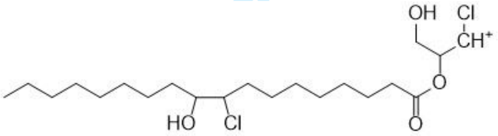

PE B-4

m/*z* 425.22199

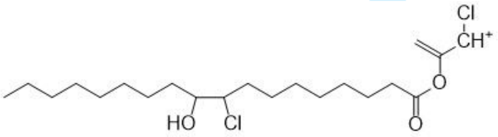

PE B-5

m/*z* 407.21143

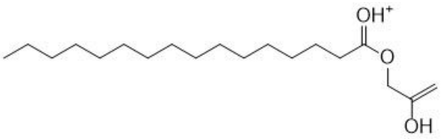

PE B-6

m/*z* 313.27372

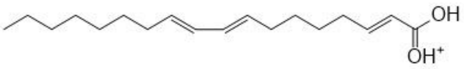

PE B-7

m/*z* 279.23186

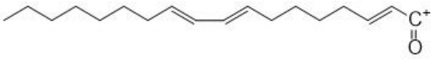

PE B-8

m/*z* 261.22129

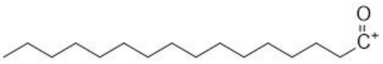

PE B-9

m/*z* 239.23694

Fig. S45 Proposed structures of fragments PE B-1–PE B-9

3.2.4 Fragmentation of PE C

Table S23 Fragments of PE C (chlorinated POPE)

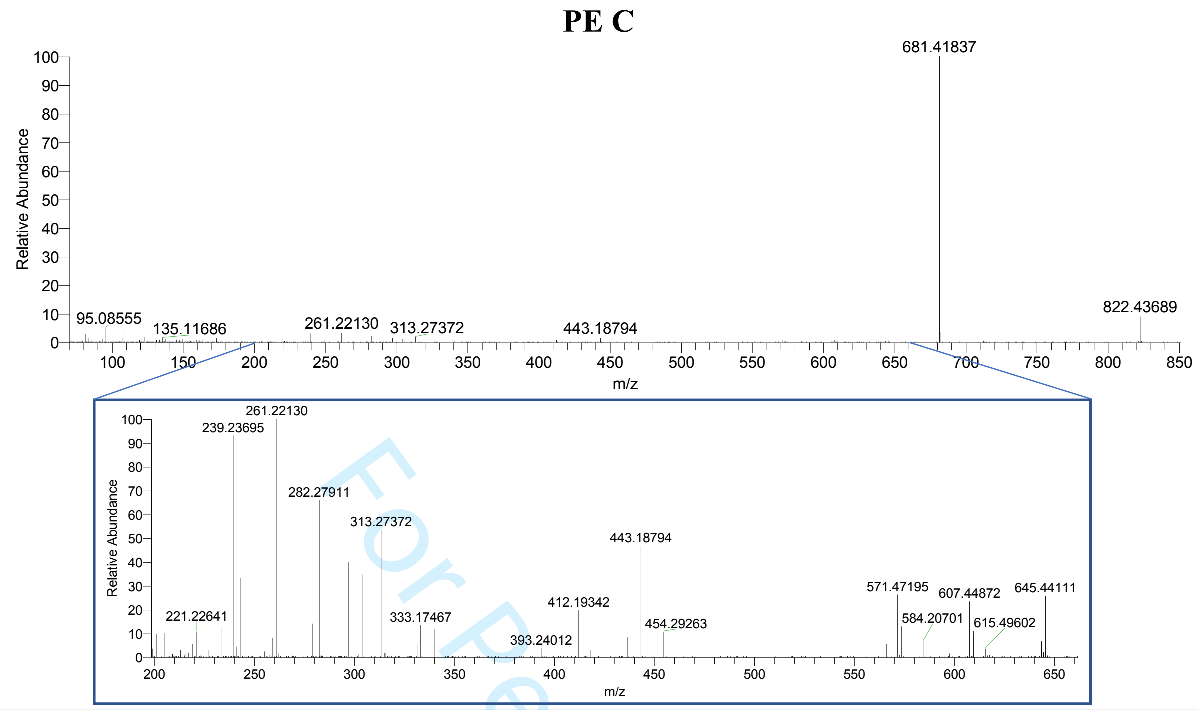

Fragment	Predicted formula	Measured *m/z*	Theoretical *m/z*	Mass difference [ppm]
PE C-1	$C_{39}H_{76}O_8NCl_3P^+$	822.43689	822.43686	0.02892
PE C-2	$C_{39}H_{72}O_8NClP^+$	748.46771	748.46786	-0.20487
PE C-3	$C_{39}H_{71}O_8NP^+$	712.49122	712.49118	0.05861
PE C-4	$C_{37}H_{68}O_4Cl_3^+$	681.41837	681.41777	0.88672
PE C-5	$C_{37}H_{67}O_4Cl_2^+$	645.44111	645.44109	0.02063
PE C-6	$C_{37}H_{66}O_4Cl^+$	609.46453	609.46441	0.18373
PE C-7	$C_{37}H_{64}O_4Cl^+$	607.44872	607.44876	-0.06574
PE C-8	$C_{23}H_{46}O_7NCl_3P^+$	584.20701	584.20720	-0.32809
PE C-9	$C_{37}H_{65}O_4^+$	573.48762	573.48774	-0.20880
PE C-10	$C_{37}H_{63}O_4^+$	571.47195	571.47209	-0.24529
PE C-11	$C_{23}H_{44}O_6NCl_3P^+$	566.19676	566.19663	0.23018
PE C-12	$C_{21}H_{45}O_7NP^+$	454.29263	454.29282	-0.41937
PE C-13	$C_{21}H_{43}O_6NP^+$	436.28198	436.28225	-0.62861
PE C-14	$C_{19}H_{37}O_3^+$	313.27372	313.27372	0.01010
PE C-15	$C_{18}H_{29}O^+$	261.22130	261.22129	0.03624
PE C-16	$C_{16}H_{31}O^+$	239.23695	239.23694	0.02258

For Peer Review

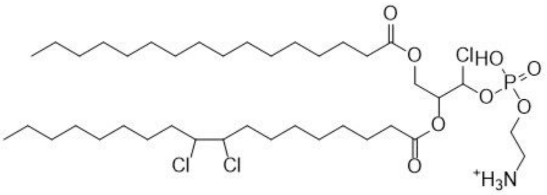

PE C-1

m/z 822.43686

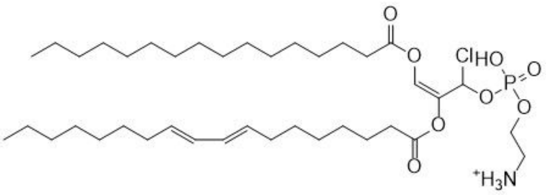

PE C-2

m/z 748.46786

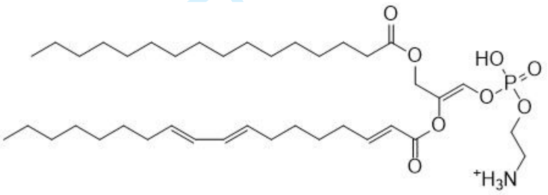

PE C-3

m/z 712.49118

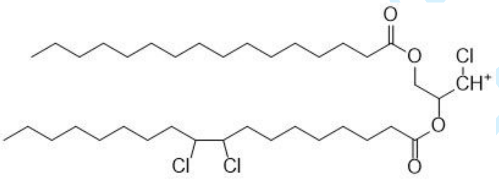

PE C-4

m/z 681.41777

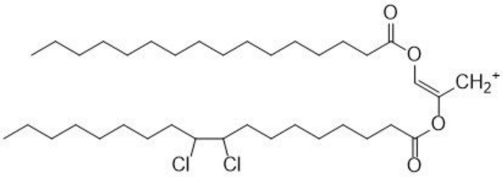

PE C-5

m/z 645.44109

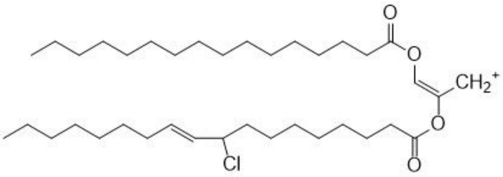

PE C-6

m/z 609.46441

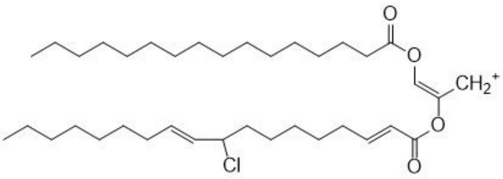

PE C-7

m/z 607.44876

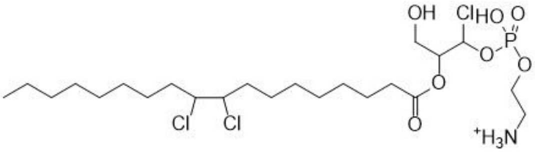

PE C-8

m/z 584.20720

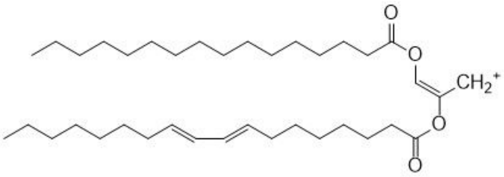

PE C-9

m/z 573.48774

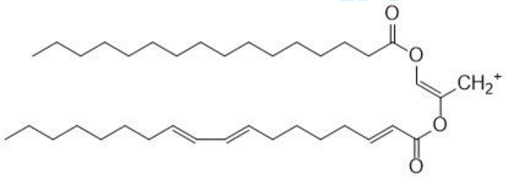

PE C-10

m/z 571.47209

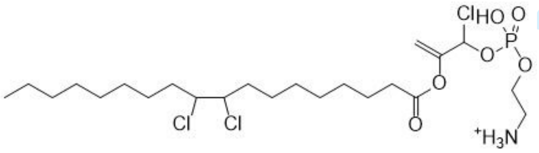

PE C-11

m/z 566.19663

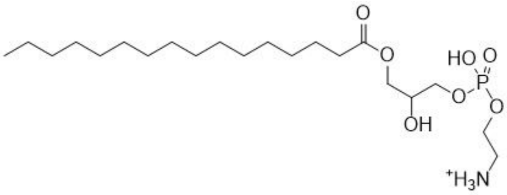

PE C-12

m/z 454.29282

**PE C-13**<br>*m/z* 436.28225	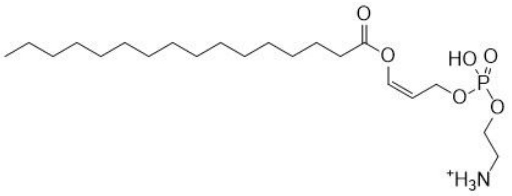
**PE C-14**<br>*m/z* 313.27372	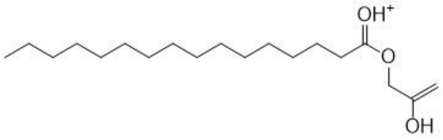
**PE C-15**<br>*m/z* 261.22129	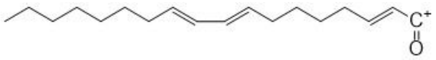
**PE C-16**<br>*m/z* 239.23694	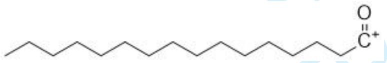

Fig. S46 Proposed structures of fragments PE C-1–PE C-16

For Peer Review

3.2.5 Fragmentation of PE D

Table S24 Fragments of PE D (chlorinated POPE)

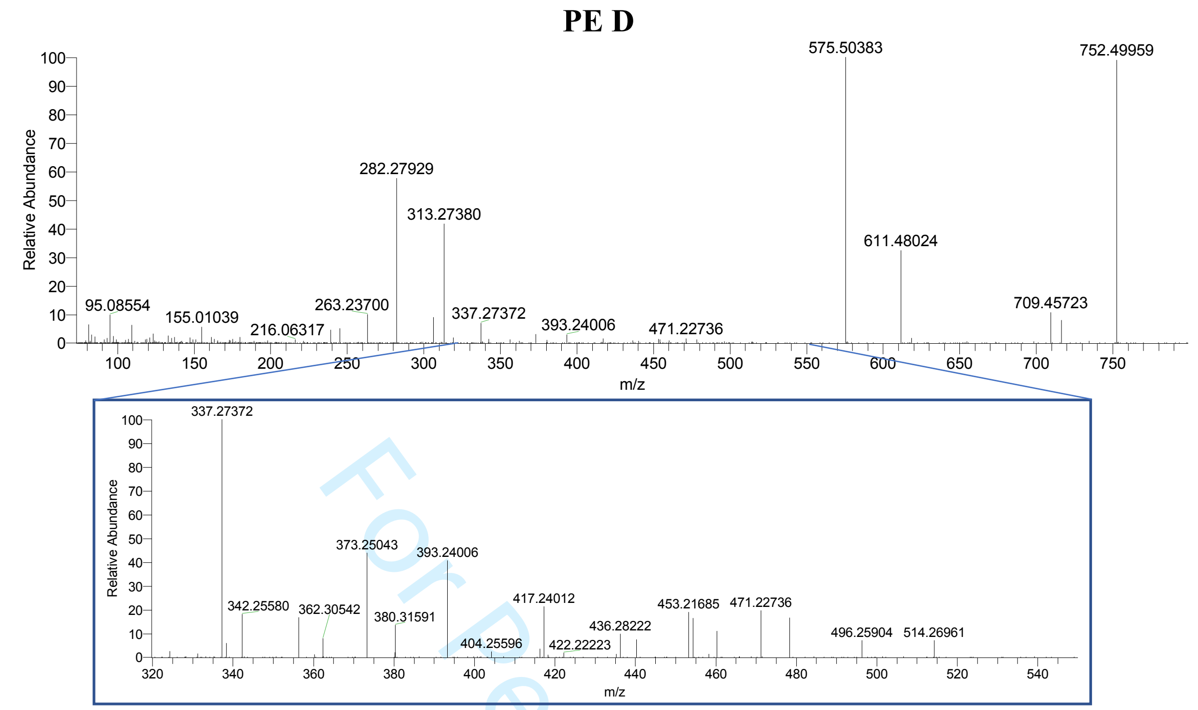

Fragment	Predicted formula	Measured *m/z*	Theoretical *m/z*	Mass difference [ppm]
PE D-1	$C_{39}H_{76}O_8NClP^+$	752.49959	752.49916	0.57852
PE D-2	$C_{39}H_{75}O_8NP^+$	716.52274	716.52248	0.36477
PE D-3	$C_{37}H_{71}O_8ClP^+$	709.45723	709.45696	0.37670
PE D-4	$C_{37}H_{68}O_4Cl^+$	611.48024	611.48006	0.29172
PE D-5	$C_{37}H_{67}O_4^+$	575.50383	575.50339	0.77651
PE D-6	$C_{23}H_{46}O_7NClP^+$	514.26961	514.26949	0.21802
PE D-7	$C_{23}H_{44}O_6NClP^+$	496.25904	496.25893	0.21975
PE D-8	$C_{23}H_{45}O_7NP^+$	478.29274	478.29282	-0.16183
PE D-9	$C_{21}H_{41}O_7ClP^+$	471.22736	471.22729	0.14506
PE D-10	$C_{21}H_{38}O_6P^+$	417.24012	417.24005	0.15665
PE D-11	$C_{19}H_{38}O_6P^+$	393.24006	393.24005	0.02156
PE D-12	$C_{21}H_{38}O_3Cl^+$	373.25043	373.25040	0.07417
PE D-13	$C_{21}H_{37}O_3^+$	337.27372	337.27372	0.00562
PE D-14	$C_{19}H_{37}O_3^+$	313.27380	313.27372	0.23586
PE D-15	$C_{18}H_{31}O^+$	263.23700	263.23694	0.21720
PE D-16	$C_{16}H_{33}O_2^+$	257.24756	257.24751	0.19611
PE D-17	$C_{16}H_{31}O^+$	239.23697	239.23694	0.13715

For Peer Review

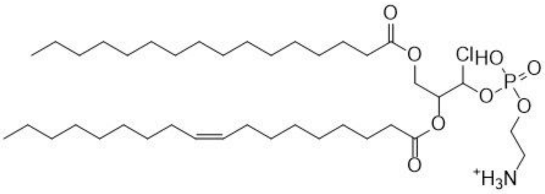

PE D-1

m/z 752.49916

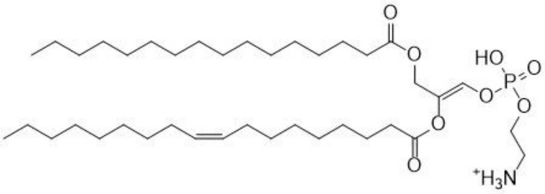

PE D-2

m/z 716.52248

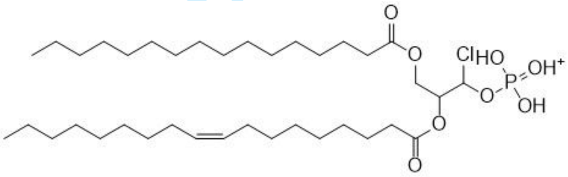

PE D-3

m/z 709.45696

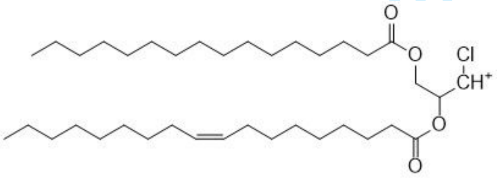

PE D-4

m/z 611.48006

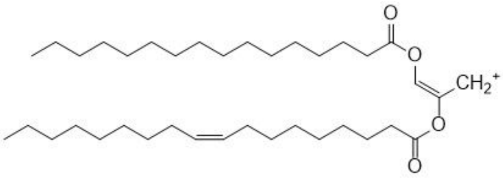

PE D-5

m/z 575.50339

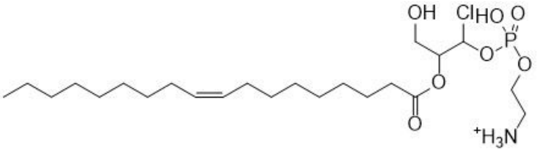

PE D-6

m/z 514.26949

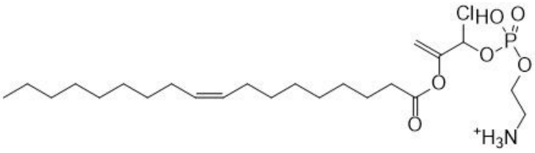

PE D-7

m/*z* 496..25893

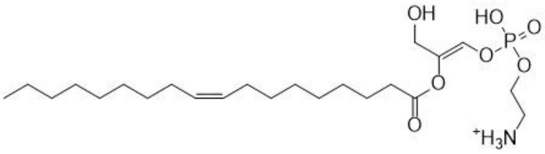

PE D-8

m/*z* 478.29282

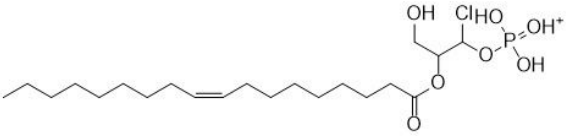

PE D-9

m/*z* 471.22729

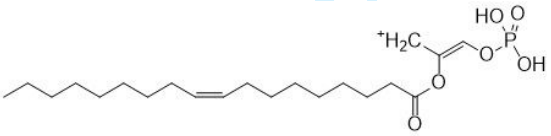

PE D-10

m/*z* 417.24005

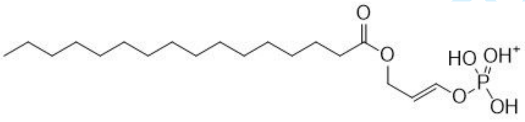

PE D-11

m/*z* 393.24005

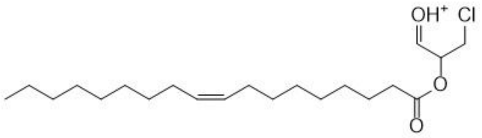

PE D-12

m/*z* 373.25040

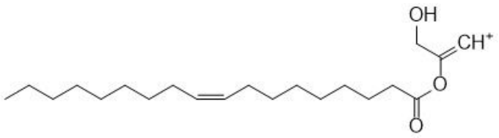

PE D-13

m/*z* 337.27372

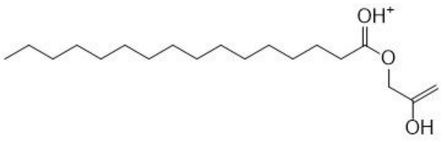

PE D-14

m/*z* 313.27372

**PE D-15** *m/z* 263.23694	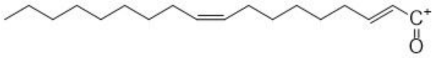
**PE D-16** *m/z* 257.24751	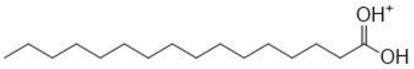
**PE D-17** *m/z* 239.23694	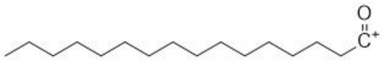

Fig. S47 Proposed structures of fragments PE D-1–PE D-17

For Peer Review

3.2.6 Fragmentation of PE E

Table S25 Fragments of PE E (chlorinated POPE)

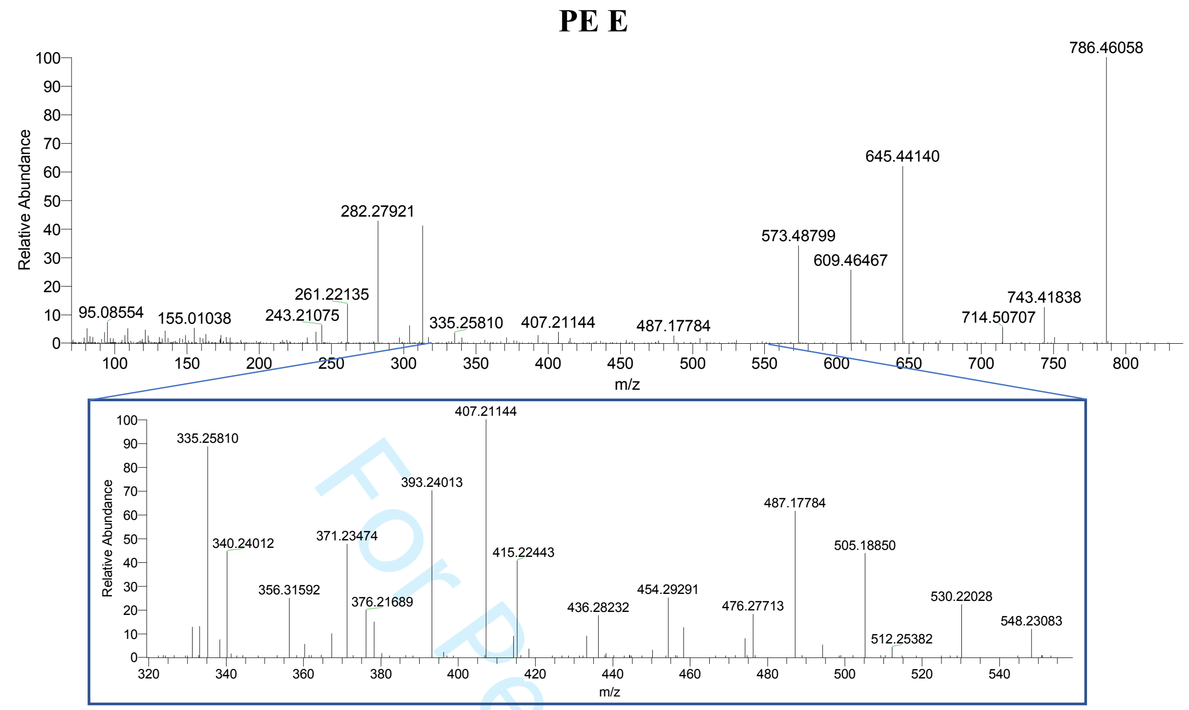

Fragment	Predicted formula	Measured *m/z*	Theoretical *m/z*	Mass difference [ppm]
PE E-1	$C_{39}H_{75}O_8NCl_2P^+$	786.46058	786.46019	0.49692
PE E-2	$C_{39}H_{74}O_8NClP^+$	750.48384	750.48351	0.44161
PE E-3	$C_{37}H_{70}O_8Cl_2P^+$	743.41838	743.41799	0.52986
PE E-4	$C_{39}H_{73}O_8NP^+$	714.50707	714.50683	0.33619
PE E-5	$C_{37}H_{67}O_4Cl_2{}^+$	645.44140	645.44109	0.47626
PE E-6	$C_{37}H_{66}O_4Cl^+$	609.46467	609.46441	0.42075
PE E-7	$C_{37}H_{65}O_4{}^+$	573.48799	573.48774	0.44134
PE E-8	$C_{23}H_{45}O_7NCl_2P^+$	548.23083	548.23052	1.12942
PE E-9	$C_{23}H_{43}O_6NCl_2P^+$	530.22028	530.21996	0.60137
PE E-10	$C_{21}H_{40}O_7Cl_2P^+$	505.18850	505.18832	0.35906
PE E-11	$C_{23}H_{42}O_6NClP^+$	494.24347	494.24328	0.37736
PE E-12	$C_{21}H_{38}O_6Cl_2P^+$	487.17784	487.17776	0.16040
PE E-13	$C_{23}H_{43}O_7NP^+$	476.27713	476.27717	-0.07976
PE E-14	$C_{21}H_{37}O_3Cl_2{}^+$	407.21144	407.21143	0.03059
PE E-15	$C_{21}H_{36}O_3Cl^+$	371.23474	371.23475	-0.02039
PE E-16	$C_{21}H_{35}O_3{}^+$	335.25810	335.25807	0.07951
PE E-17	$C_{19}H_{37}O_3{}^+$	313.27378	313.27372	0.19122
PE E-18	$C_{18}H_{29}O^+$	261.22135	261.22129	0.20549
PE E-19	$C_{16}H_{33}O_2{}^+$	257.24758	257.24751	0.27009
PE E-20	$C_{16}H_{31}O^+$	239.23697	239.23694	0.13013

For Peer Review

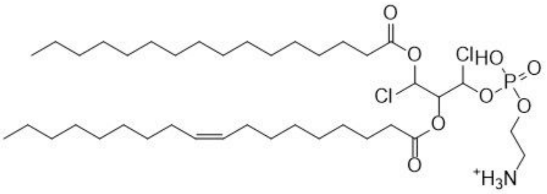

PE E-1

m/z 786.46019

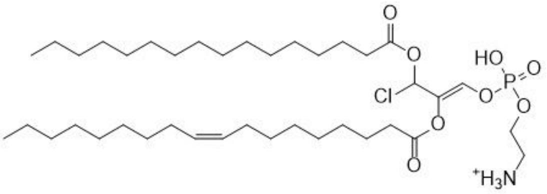

PE E-2

m/z 750.48351

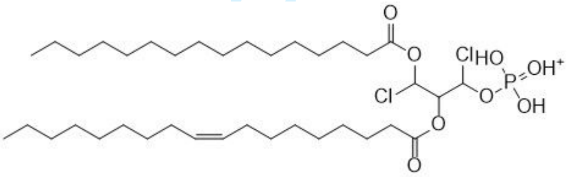

PE E-3

m/z 743.41799

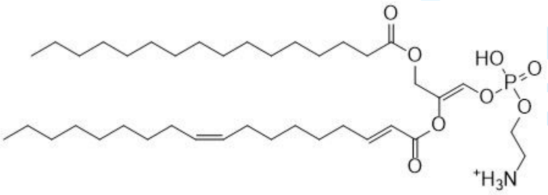

PE E-4

m/z 714.50683

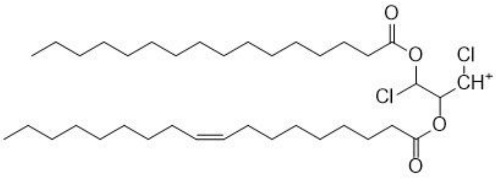

PE E-5

m/z 645.44109

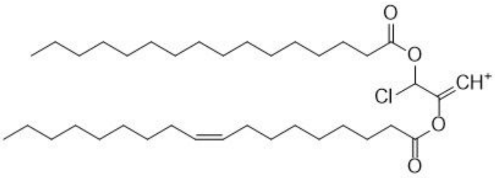

PE E-6

m/z 609.46441

For Peer Review

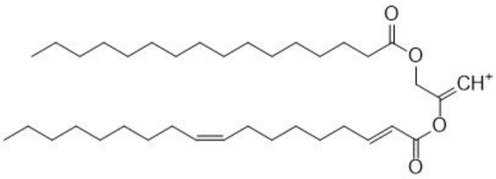

PE E-7

m/z 573.48774

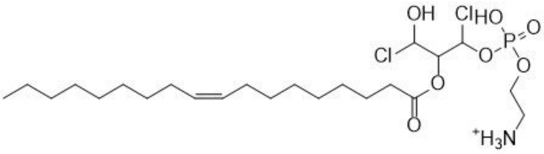

PE E-8

m/z 548.23052

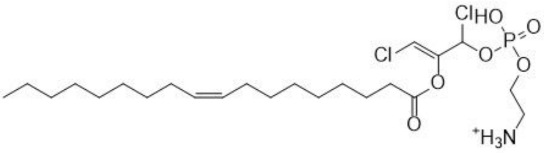

PE E-9

m/z 530.21996

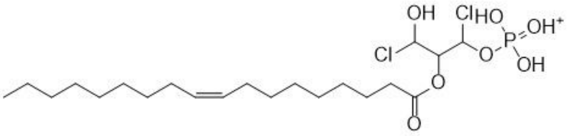

PE E-10

m/z 505.18832

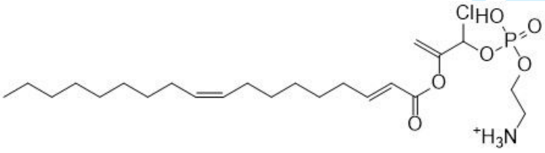

PE E-11

m/z 4.94.24328

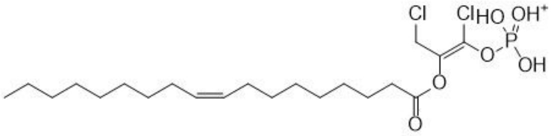

PE E-12

m/z 487.17776

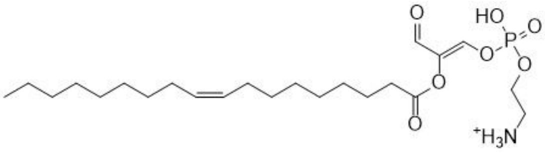

PE E-13

m/z 476.27717

PE E-14 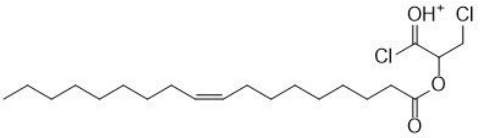

m/z 407.21143

PE E-15 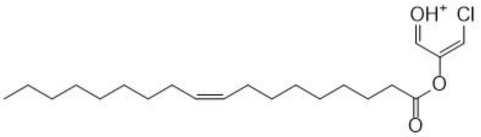

m/z 371.23475

PE E-16 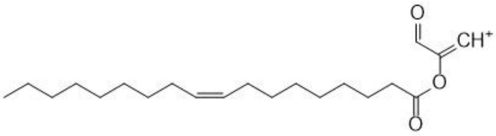

m/z 335.25807

PE E-17 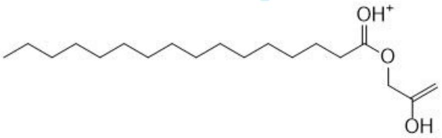

m/z 313.27372

PE E-18 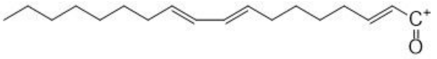

m/z 261.22129

PE E-19 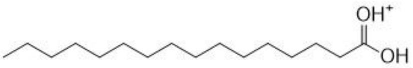

m/z 3257.24751

PE E-20 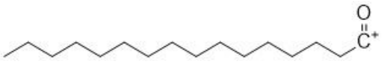

m/z 239.23694

Fig. S48 Proposed structures of fragments PE E-1–PE E-20

3.2.7 Fragmentation of PE F

Table S26 Fragments of PE F (chlorinated POPE)

PE F

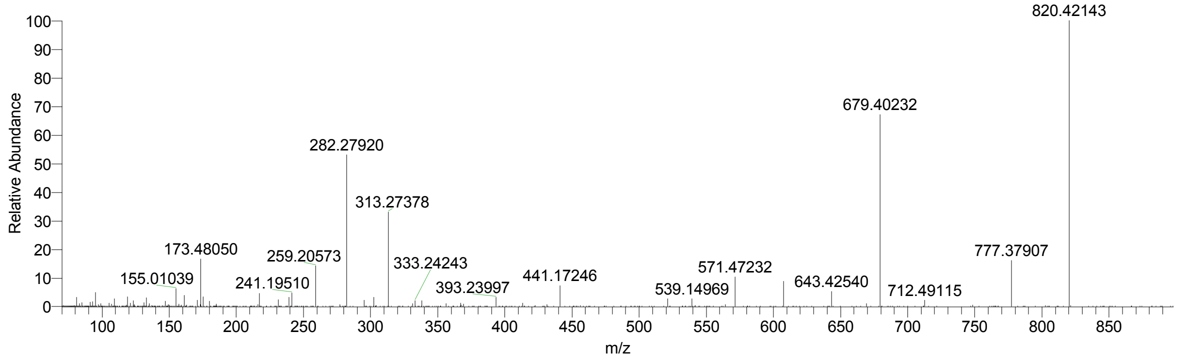

Fragment	Predicted formula	Measured *m/z*	Theoretical *m/z*	Mass difference [ppm]
PE F-1	$C_{39}H_{74}O_8NCl_3P^+$	820.42143	820.42121	0.26606
PE F-2	$C_{37}H_{69}O_8Cl_3P^+$	777.37907	777.37902	0.06855
PE F-3	$C_{39}H_{71}O_8NP^+$	712.49115	712.49118	-0.03701
PE F-4	$C_{37}H_{66}O_4Cl_3^+$	679.40232	679.40212	-0.16170
PE F-5	$C_{37}H_{65}O_4Cl_2^+$	643.42540	643.42544	-0.06250
PE F-6	$C_{37}H_{64}O_4Cl^+$	607.44899	607.44876	0.36675
PE F-7	$C_{37}H_{63}O_4^+$	571.47232	571.47209	0.50458
PE F-8	$C_{23}H_{42}O_6NCl_3P^+$	564.18182	564.18098	1.48775
PE F-9	$C_{21}H_{39}O_7Cl_3P^+$	539.14969	539.14935	0.63366
PE F-10	$C_{21}H_{37}O_6Cl_3P^+$	521.13898	521.13878	0.37250
PE F-11	$C_{21}H_{36}O_3Cl_3^+$	441.17246	441.17245	0.00125
PE F-12	$C_{19}H_{37}O_3^+$	313.27378	313.27372	0.18229

For Peer Review

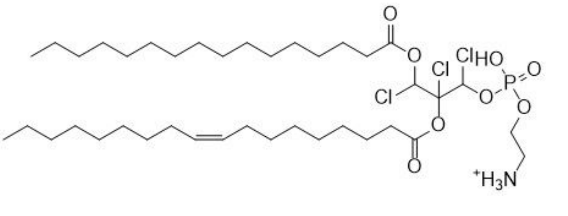

PE F-1

m/z 820.42121

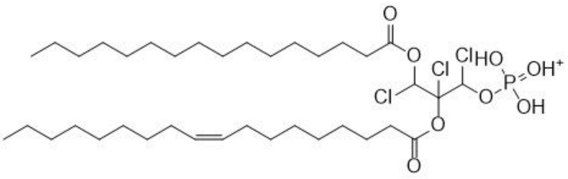

PE F-2

m/z 777.37902

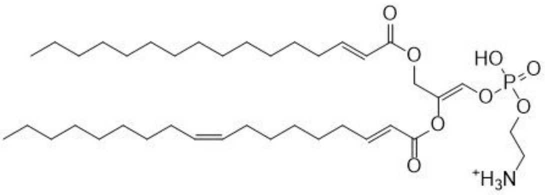

PE F-3

m/z 712.49118

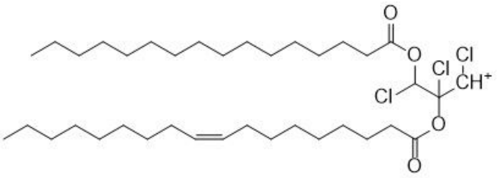

PE F-4

m/z 679.40212

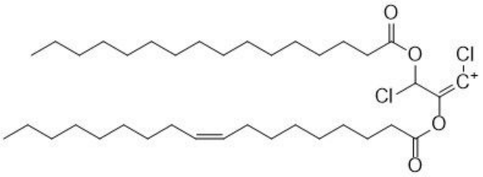

PE F-5

m/z 643.42544

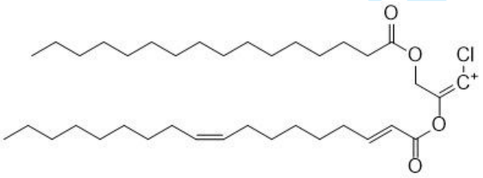

PE F-6

m/z 607.44876

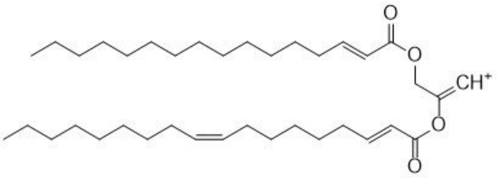

PE F-7

m/z 571.47209

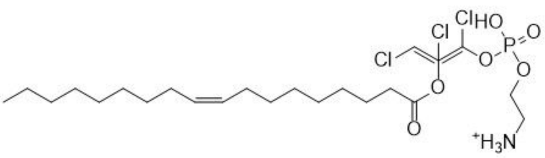

PE F-8

m/z 564.18098

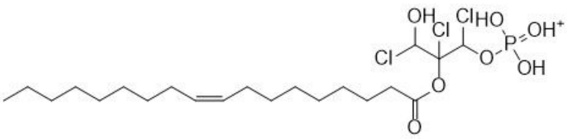

PE F-9

m/z 539.14935

For Peer Review

**PE F-10**<br>*m/z* 521.13879	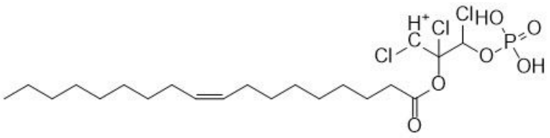
**PE F-11**<br>*m/z* 441.17245	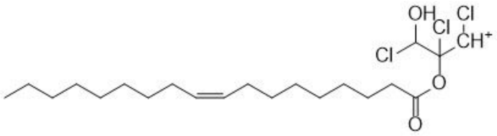
**PE F-12**<br>*m/z* 313.27372	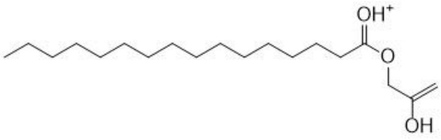

Fig. S49 Proposed structures of fragments PE F-1–PE F-12

For Peer Review

4 Chromatograms of PLC-treatment

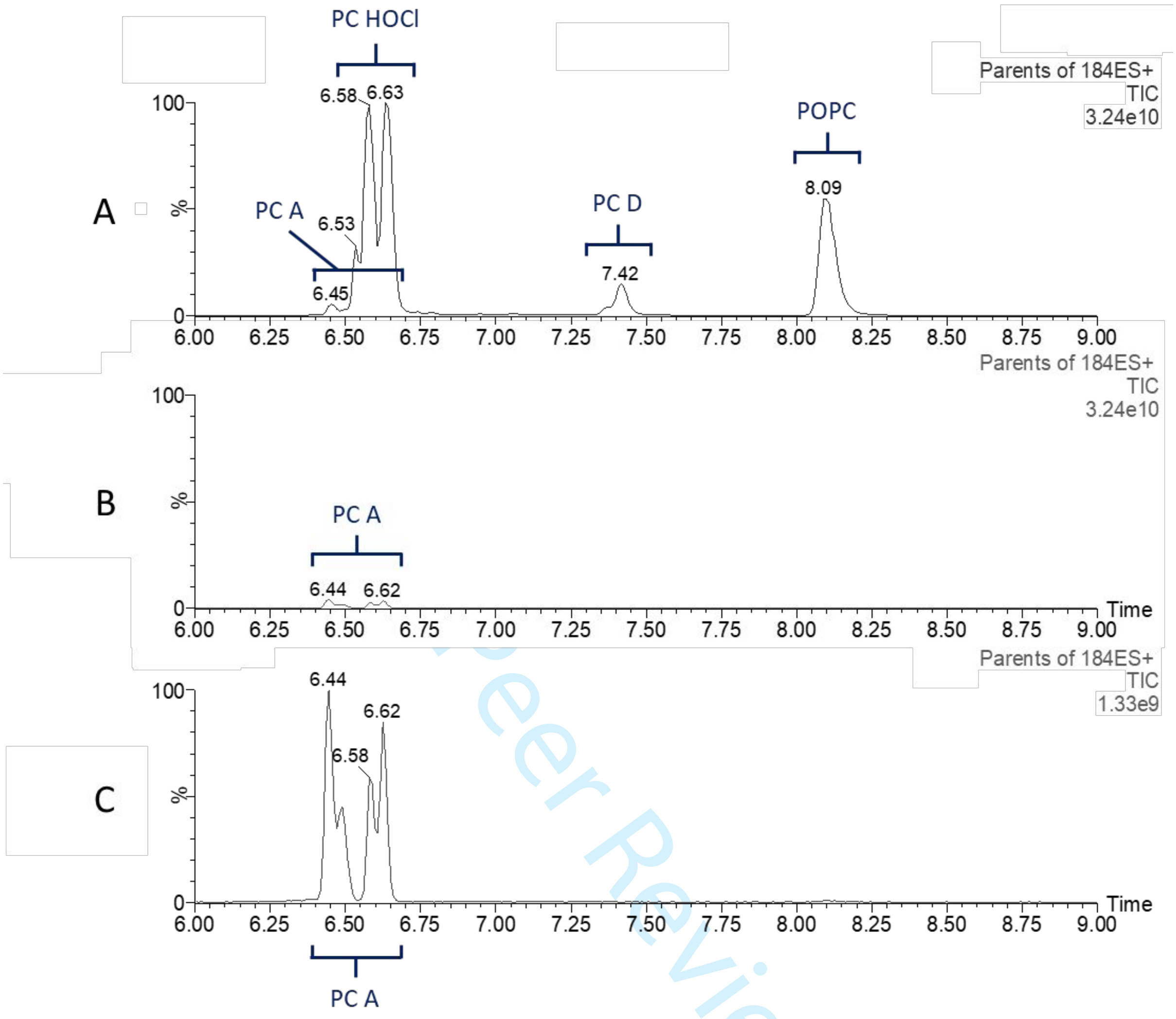

Fig. S50 Positive precursor (parent) ion scans of *m/z* 184 (C18 LC-MS/MS) chromatograms of PLC-treated and -untreated, chlorinated aqueous POPC samples. A. PLC-untreated sample. B. PLC-treated sample. C. Same as B, but the chromatogram is normalized to its most intense peak, to better visualize the PLC-catalysed degradation of PC HOCl, PC D and POPC. Y-axes (signal intensity) in A and B are normalized for comparison. Equal amounts of chlorinated POPC was used in the PLC-treated and -untreated samples

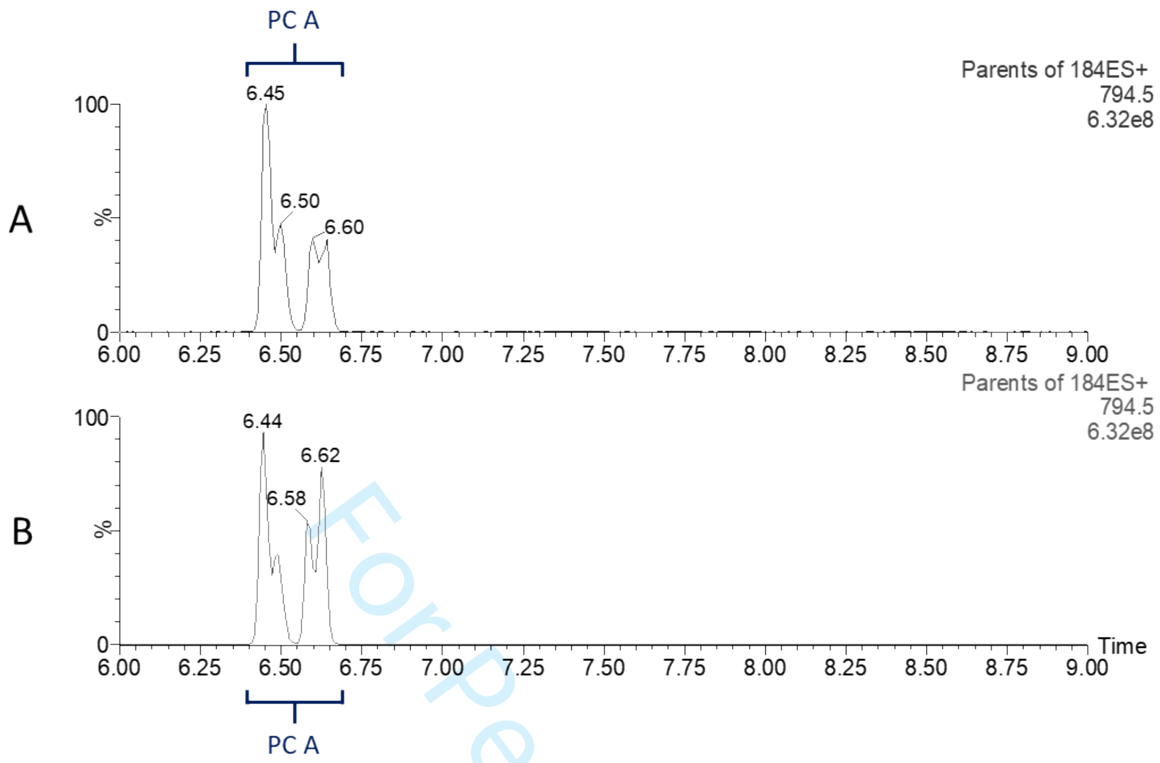

Fig. S 51 EICs (*m*/*z* 794.5) of positive precursor (parent) ion scans of *m*/*z* 184 (C18 LC-MS/MS) chromatograms of PLC-treated and -untreated, chlorinated aqueous POPC samples. A. PLC-untreated sample. B. PLC-treated sample. PC A is seen at RT 6.44 -6.62 min in both samples. Y-axes (signal intensity) are normalized for comparison. Equal amounts of chlorinated POPC was used in the PLC-treated and -untreated samples

For Peer Review

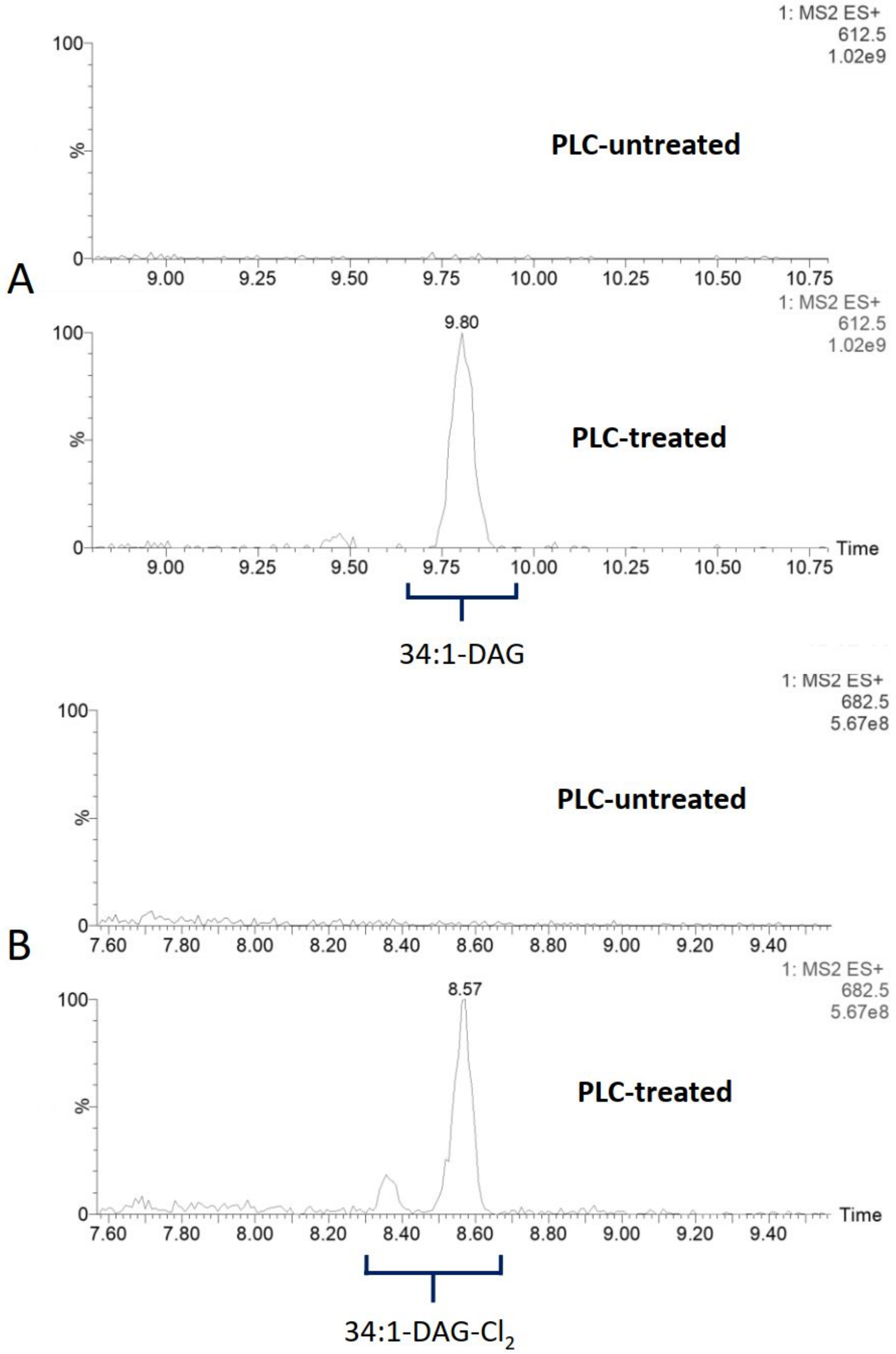
A
1: MS2 ES+
612.5
1.02e9
PLC-untreated
PLC-treated
9.80
Time
34:1-DAG
B
1: MS2 ES+
682.5
5.67e8
PLC-untreated
PLC-treated
8.57
Time
34:1-DAG-Cl2

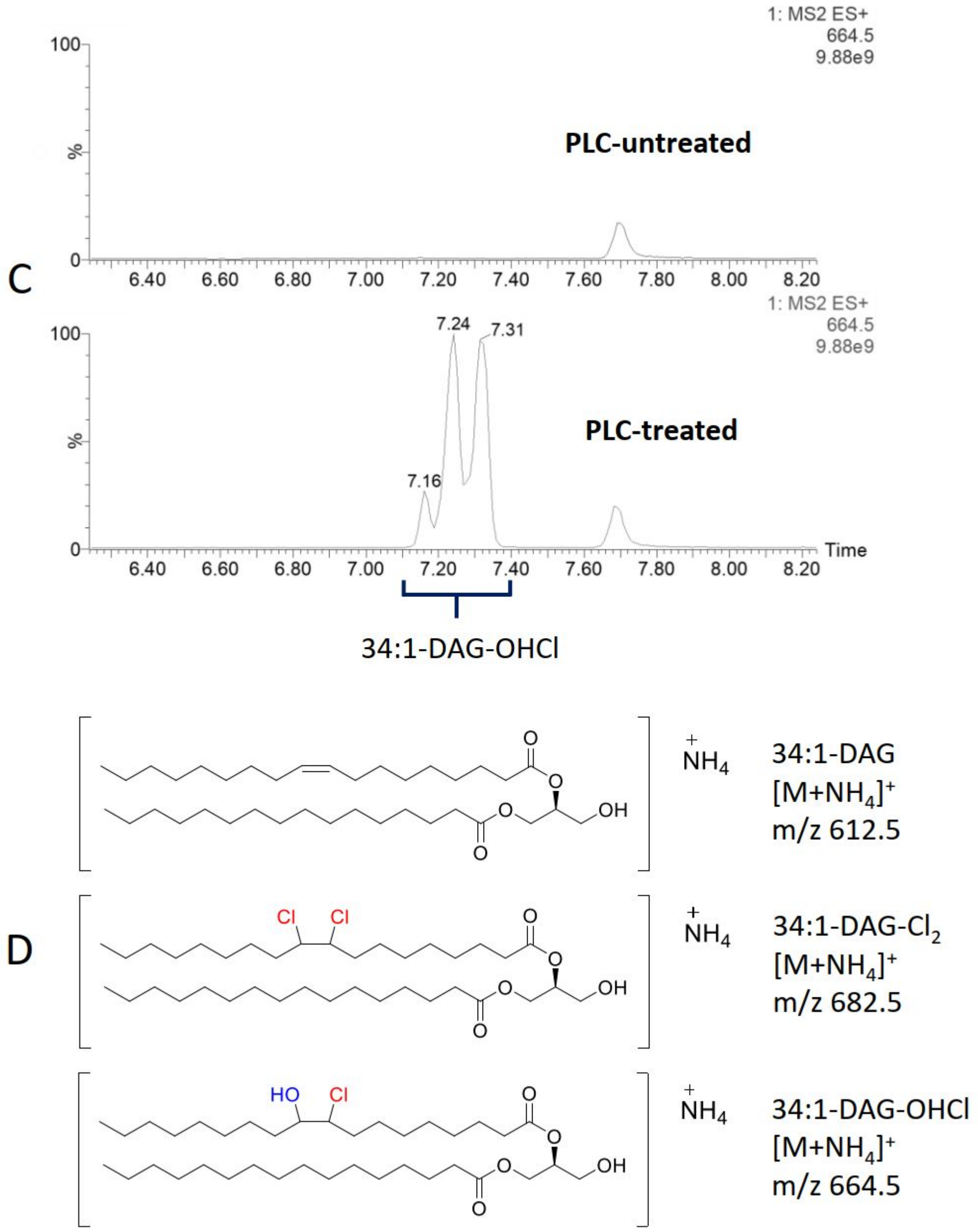

Fig. S52 Positive full scan (*m/z* 600-900) LC-MS EICs of *m/z* 612.5 (34:1-DAG), *m/z* 682.5 (34:1-DAG-Cl_2) and *m/z* 664.5 (34:1-DAG-OHCl) of PLC-untreated (upper chromatograms) and PLC-treated (lower chromatograms) chlorinated aqueous POPC samples. D. Likely structures of the diacylglycerols (DAGs) produced by PLC-treatment. The DAGs are ionized by adduction with ammonium ($[M+NH_4]^+$)

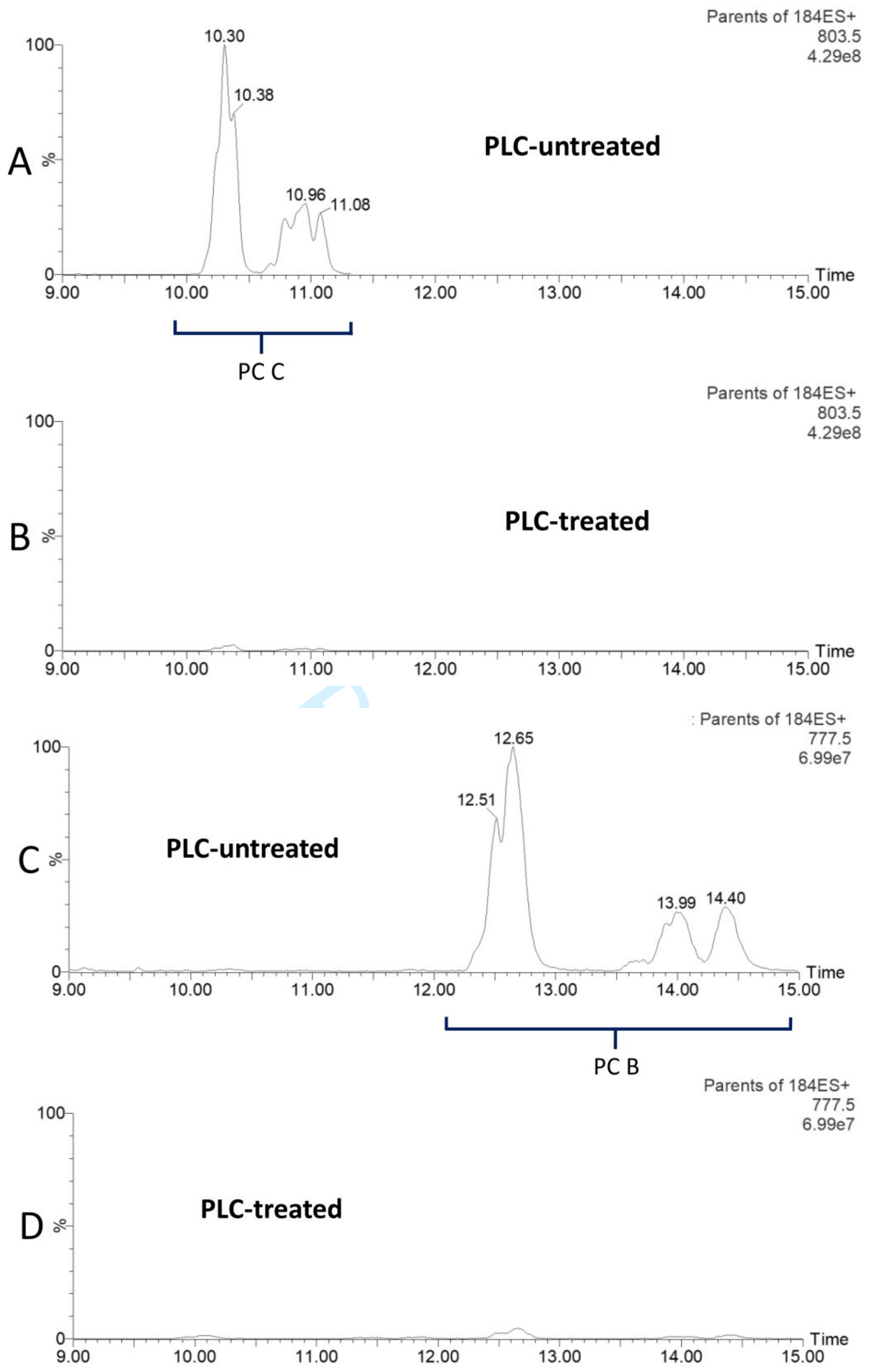

Fig. S53 EICs (A and B = *m/z* 803.5, C and D = *m/z* 777.5) of positive precursor (parent) ion scans of *m/z* 184 (C18 LC-MS/MS) chromatograms of PLC-treated and -untreated, chlorinated aqueous POPC samples
